# Novel Benzimidazol-2-Acetamide Derivatives as Submicromolar Casein Kinase 1δ Inhibitors with Favourable Predicted Drug-like Properties

**DOI:** 10.3390/ph19101666

**Published:** 2026-10-21

**Authors:** Costanza Ceni, Sara Calenda, Giulia Vagnoni, Elisa Marinari, Daniela Catarzi, Flavia Varano, Letizia Trevisan, Michelle Fornasier, Stephanie Federico, Giampiero Spalluto, Gianluca Novello, Veronica Salmaso, Stefano Moro, Martino Calamai, Vittoria Colotta

**Affiliations:** 1 Section of Pharmaceutical and Nutraceutical Sciences, Department of Neuroscience, Psychology, Drug Research and Child Health (NEUROFARBA), University of Florence, Via Ugo Schiff, 6, 50019 Sesto Fiorentino, Italy; costanza.ceni@unifi.it (C.C.); sara.calenda@unifi.it (S.C.); giulia.vagnoni@unifi.it (G.V.); elisa.marinari@unifi.it (E.M.); daniela.catarzi@unifi.it (D.C.); flavia.varano@unifi.it (F.V.); 2 Department of Chemical and Pharmaceutical Sciences, University of Trieste, Via Licio Giorgieri 1, 34127 Trieste, Italy; letizia.trevisan@phd.units.it (L.T.); michelle.fornasier@studenti.units.it (M.F.); sfederico@units.it (S.F.); spalluto@units.it (G.S.); 3 Molecular Modeling Section (MMS), Department of Pharmaceutical and Pharmacological Sciences, University of Padova, Via Marzolo 5, 35131 Padova, Italy; gianluca.novello@phd.unipd.it (G.N.); veronica.salmaso@unipd.it (V.S.); stefano.moro@unipd.it (S.M.); 4 European Laboratory for Non-Linear Spectroscopy (LENS), University of Florence, Via Nello Carrara 1, 50019 Sesto Fiorentino, Italy; calamai@lens.unifi.it; 5 National Institute of Optics-National Research Council (CNR-INO), Via Nello Carrara 1, 50019 Sesto Fiorentino, Italy

**Keywords:** CK1δ inhibitors, benzimidazole derivatives, molecular modelling

## Abstract

**Background/Objective:** Casein kinase 1δ (CK1δ) plays a central role in regulating circadian rhythms, DNA repair, apoptosis, and cell proliferation, and its dysregulation is associated with both cancer and neurodegenerative diseases, making CK1δ an attractive therapeutic target. This study aimed to develop novel CK1δ inhibitors with favourable drug-like properties through the synthesis and biological evaluation of benzimidazol-2-acetamide derivatives. **Methods:** A novel series of benzimidazol-2-acetamide derivatives was synthesized, incorporating different substituents on the benzimidazole core (5-chloro, 5,6-dichloro, 5-nitro, and 5-cyano) and various saturated cyclic amines on the acetamide moiety. Compounds were evaluated for CK1δ inhibitory activity, while molecular docking and molecular dynamics simulations were performed to investigate their binding mode. In silico ADME predictions assessed pharmacokinetic properties, and cytotoxicity was evaluated in SH-SY5Y neuroblastoma cells. **Results:** The 5,6-dichloro derivatives **20** and **24**, bearing piperidin-4-ylmethanamine and morpholine moieties, respectively, displayed the highest CK1δ inhibitory activity (IC_50_ = 0.172 and 0.223 μM). The 5-cyano analogue **22** also exhibited a good potency (IC_50_ = 0.224 μM). Computational studies confirmed an ATP-competitive binding mode and highlighted the dynamic behaviour of the solvent-exposed substituents within the CK1δ binding site. Predicted pharmacokinetic properties suggested favourable oral absorption and blood–brain barrier penetration. Compounds **20**, **22**, and **24** showed no cytotoxicity in SH-SY5Y cells at concentrations up to 10 μM. **Conclusions:** The new benzimidazol-2-acetamide derivatives exhibited good inhibitor activity against CK1δ, favourable predicted ADME properties, and low cellular toxicity. The combined experimental and computational findings provide valuable insights for the further optimization of this scaffold toward the development of therapeutics for CK1δ-related diseases.

## 1. Introduction

Casein kinase 1 delta (CK1δ) is a serine/threonine kinase and one of the six human members of the conserved CK1 family [1], alongside α, γ1–3, δ, and ε isoforms [2]. CK1δ is found in both the cytoplasm and nucleus of eukaryotic cells and can associate with membranes, cytoskeletal elements, and centrosomes [2,3]. Its abundance is not uniform; expression varies among tissues cell types and may change in response to physiological or pathological conditions [4]. By phosphorylating a broad range of substrates, CK1δ participates in processes such as circadian timing, DNA repair, cell growth, and apoptosis [5,6]. Aberrant CK1δ activity, often arising from overexpression or dysregulated signalling, has been implicated in the onset and progression of multiple pathological conditions [7]. In cancer, CK1δ can affect tumour behaviour through pathways that include Wnt/β-catenin, p53, Hedgehog, and Hippo signalling [8,9]. Altered CK1δ expression or activity has been described in glioblastoma, colorectal and hepatocellular carcinomas, melanoma, lung cancer, and breast and ovarian cancers, and has been associated with tumour progression and poorer clinical outcomes [7,10,11,12,13].

CK1δ has also been linked to neurodegenerative disease [14,15]. CK1δ mutations have been associated with familial advanced sleep-phase syndrome (FASPS), a circadian rhythm disorder characterized by a shift in circadian periodicity [16,17,18]. Aberrant overexpression of CK1δ has been observed in the hippocampus of patients affected by Alzheimer’s disease (AD), where the kinase colocalizes with neuritic plaques and neurofibrillary tangles [19,20,21]. In addition, CK1δ contributes to AD pathology by phosphorylating tau, a modification that promotes its assembly into paired helical filaments [21]. CK1δ also plays a key role in other proteinopathies. It phosphorylates TDP-43, a DNA/RNA-binding protein whose aberrant phosphorylation and aggregation in the cytoplasm are early events in amyotrophic lateral sclerosis (ALS) [22,23,24] and frontotemporal lobar degeneration (FTLD) [25,26]. CK1δ-dependent phosphorylation of α-synuclein likewise enhances the formation of insoluble aggregates that are the major component of Lewy bodies, a neuropathological hallmark of Parkinson’s disease (PD) [27,28].

**Figure 1:**
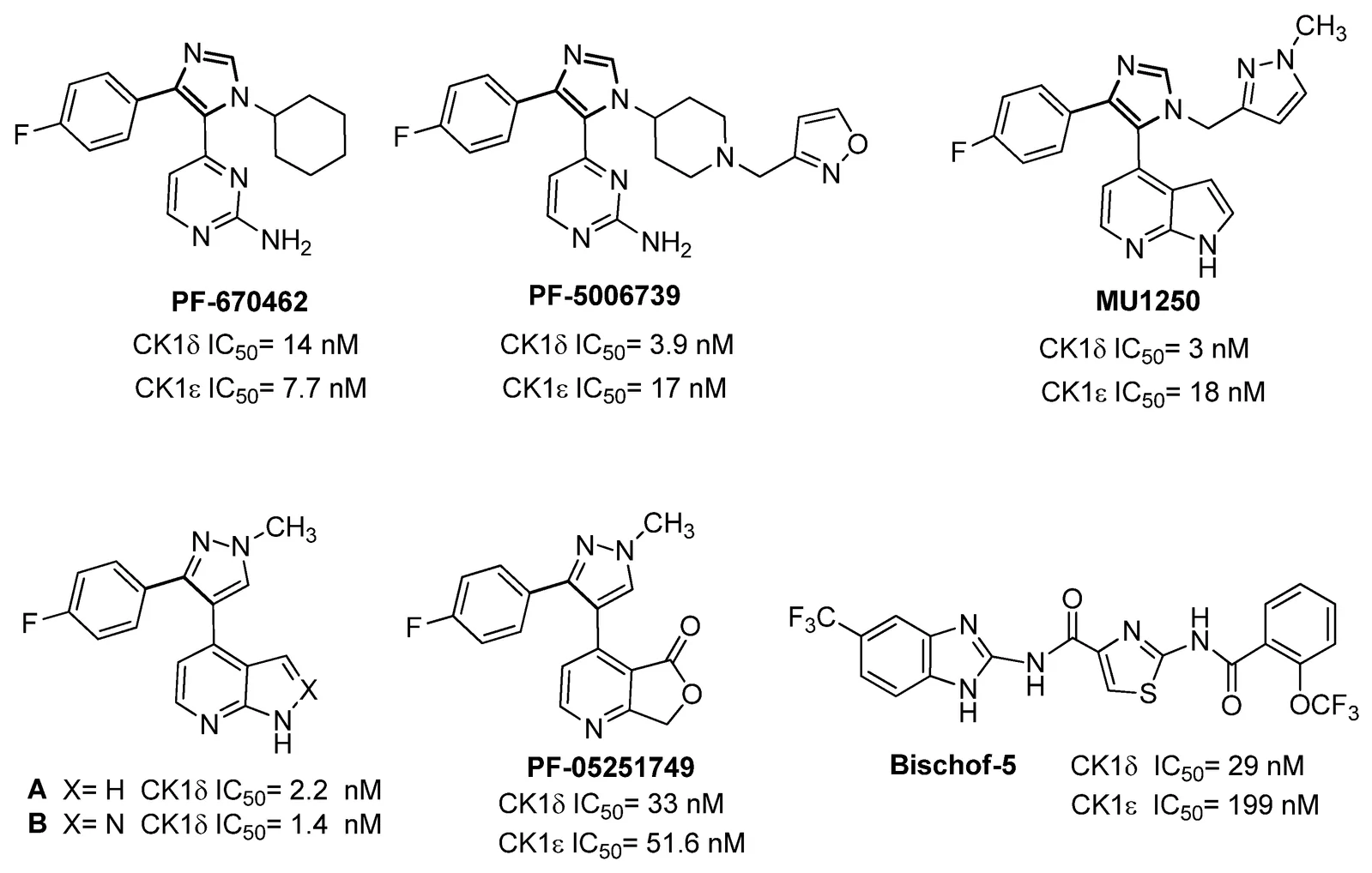
Examples of potent CK1 inhibitors.

The reported roles of CK1δ in cancer and neurodegeneration have prompted interest in inhibiting this kinase as a potential therapeutic strategy, in both oncogenic and neurodegenerative diseases. Consequently, the development of CK1δ inhibitors has garnered considerable interest to explore innovative therapeutics for cancer and CNS-related diseases [15]. Figure 1 shows the structures of selected potent CK1δ inhibitors featuring either monocyclic or bicyclic scaffolds [15]. Most of these compounds also exhibited nanomolar inhibitory activity against the CK1ε isoform, owing to the high degree of homology between the two kinases [1,2,15]. The imidazole derivatives **PF-670462** and **PF-5006739** share 2-aminopyrimidin-3-yl and *p*-fluorophenyl moieties as a structural feature. **PF-670462**, one of the first potent dual CK1δ/CK1ε inhibitors reported, reduced cognitive deficits in a mouse model of AD [29]; however, it has low oral bioavailability. Its analogue **PF-5006739** is more potent against CK1δ and has been shown to delay the circadian phase and attenuate opioid drug-seeking behaviour in in vivo models of addiction [30]. **MU1250** contains a 1*H*-pyrrolo[2,3-*b*]pyridine moiety in place of the 2-aminopyrimidine ring. Its adequate bioavailability made it a valuable chemical probe for studying CK1δ/ε in cellular and in vivo models [31].

The same 1*H*-pyrrolo[2,3-*b*]pyridine nucleus occurs in the pyrazole derivative **A**, which inhibits CK1δ at single-digit nanomolar concentration but has poor solubility. This limitation was overcome with its aza analogue **B**, containing a 1*H*-pyrazolo[3,4-*b*]pyridine core, which retained high inhibitory potency against CK1δ while displaying improved solubility and good oral bioavailability [32]. Among the pyrazole series, **PF-05251749** progressed to Phase I clinical trials, providing clinical evidence that CK1δ/ε inhibition delays the circadian phase [33,34].

**Bischof-5** is one of the most representative compounds of the 2-aminobenzimidazole series because of its CK1δ inhibitory activity and selectivity versus CK1ε [35]. Although it reduced proliferation of several cancer cell lines, poor aqueous solubility constrained its further development. To address this limitation, its structure was simplified to obtain analogues with more favourable physicochemical properties and improved cellular bioavailability [35].

**Figure 2:**
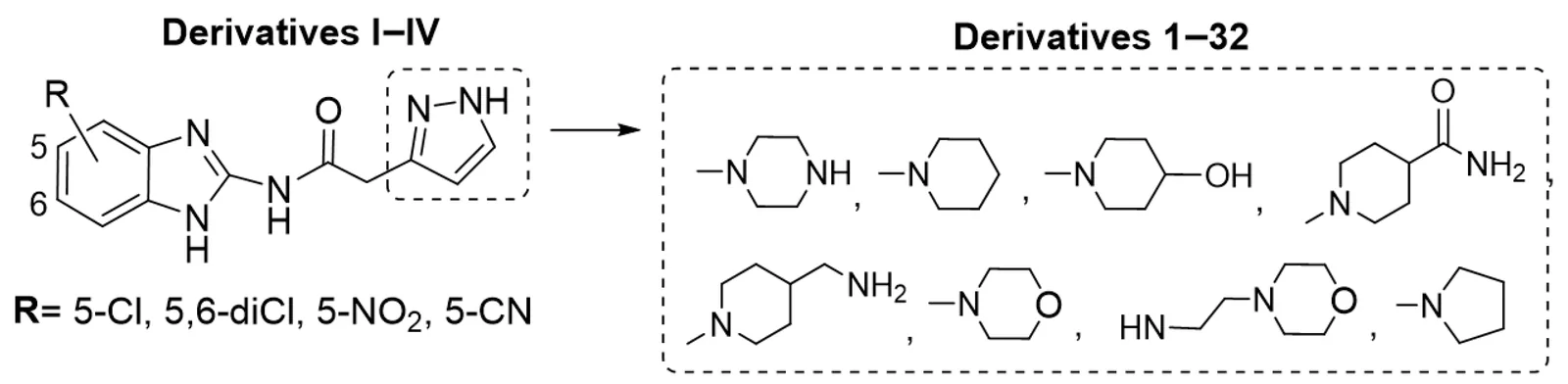
Previously (**I**–**IV**) and herein (**1**–**32**) reported benzimidazol-2-acetamide derivatives.

In this context, our research group recently developed a series of 2-amidobenzimidazole derivatives designed as simplified analogues of **Bischof-5** [36]. The compounds bear a pyrazole ring on the acetamide moiety at the 2-position and differ in their substituents (R) on the fused benzene ring (derivatives **I**–**IV**, Figure 2). Some of these compounds proved to be potent ATP-competitive CK1δ inhibitors, with potencies ranging from the low micromolar to the nanomolar range. Among the R substituents, the 5-chloro, 5,6-dichloro, 5-nitro, and 5-cyano groups were the most advantageous, yielding inhibitors with IC_50_ values ranging from 0.0986 µM (R = 5-CN) to 0.98 µM (R = 5,6-Cl_2_) [37].

Guided by these structure–activity relationships (SARs), we designed new derivatives that retain the benzimidazol-2-acetamide scaffold and the most favourable substitution patterns on the fused benzene ring, while the pyrazol-3-yl group is replaced with saturated nitrogen-containing heterocycles, such as piperazine, piperidine, morpholine, and pyrrolidine (Figure 2). This modification was aimed at investigating whether the introduction of basic saturated N-heterocycles, which are widely used in medicinal chemistry to modulate aqueous solubility, lipophilicity, and other drug-like properties [38,39,40,41] could improve the overall drug-like profile of this series while preserving CK1δ inhibitory activity. Accordingly, derivatives **1**–**32** (Figure 2) were synthesized.

These modifications were also suggested by computational studies, highlighting that these residues could project toward the solvent-exposed region of the kinase active site. Therefore, the introduction of polar or hydrogen-bond-donor/acceptor groups at this position was considered to be well tolerated and potentially advantageous for kinase-inhibitor interactions.

## 2. Results and Discussion

### 2.1. Chemistry

**Scheme 1:**
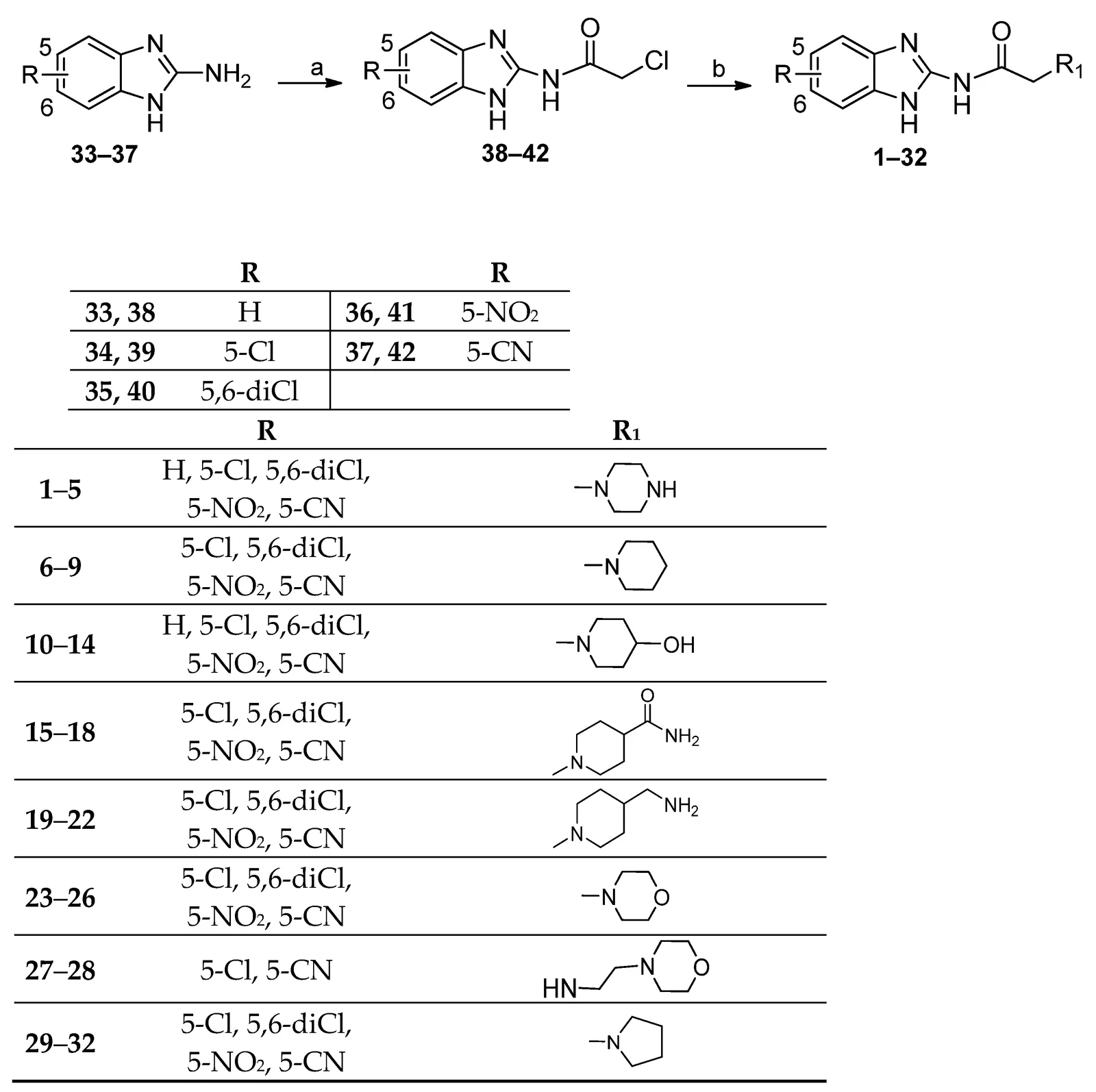
Reagents and conditions: (**a**) ClCH_2_COCl, NEt_3_, anhydrous THF; (**b**) (**i**) suitable amine, anhydrous K_2_CO_3_, anhydrous CH_3_CN; (**ii**) H_2_O/HCl 1M, pH = 8.

The synthesis of 2-acetamidobenzimidazole derivatives **1**–**32** was carried out as depicted in Scheme 1.

The 2-aminobenzimidazoles **33**–**37** were prepared by cyclizing the corresponding *o*-phenylenediamines with cyanogen bromide, as previously reported [37]. Reaction with chloroacetyl chloride afforded the 2-chloroacetamide intermediates **38**–**42**, which were converted into the final benzimidazole derivatives **1**–**32** by nucleophilic substitution with the appropriate amines.

The structures of the benzimidazole derivatives, including both intermediates and final compounds, are depicted as single tautomers, without accounting for prototropic tautomerism involving the benzimidazole NH proton. Accordingly, the structures shown in the figures and schemes correspond to the tautomers in which the substituents are assigned the lowest possible locants.

### 2.2. Structure–Activity Relationship (SAR) Studies

The newly synthesized derivatives **1**–**32** were evaluated on truncated CK1δ using the luminescent kinase assay Kinase-Glo^®^ Kit (Promega Italy S.r.l., Milan, Italy). The results are summarized in Table 1, where inhibitory activity of the lead compounds **I**–**IV** [37] are also reported for comparison.

All derivatives were initially tested at a fixed concentration of 40 μM. For compounds displaying less than 50% residual kinase activity, IC_50_ values were determined.

The obtained data indicated that the new benzimidazol-2-acetamido derivatives **1**–**32** inhibited CK1δ with potencies ranging from the submicromolar to the low micromolar range. Among the compounds with measurable IC_50_ values, the potency ranged from 0.172 to 7.96 μM, while derivatives **1**, **3**, **5**, **10** and **21** showed negligible activity (IC_50_ > 40 μM). The SAR analysis confirmed the importance of substitution on the fused benzene ring for CK1δ inhibition, as previously observed for this series [37]. In fact, the unsubstituted derivatives **1** and **10** were significantly less active against CK1δ (IC_50_ > 40 μM) than the corresponding compounds bearing R substituents (derivatives **2**, **4** and **11**–**14**, respectively). The replacement of the pyrazol-3-yl group of the lead compounds **I**–**IV** with a basic moiety, aimed at improving the physicochemical profile, produced different effects, depending on the substituent on the benzo ring.

Overall, the novel compounds showed slightly lower inhibitory activity than lead compounds **I**–**IV**, except for the 5,6-dichloro-substituted derivatives **12**, **16**, **20**, and **24**, which exhibited comparable or, in some cases, slightly greater potency than the corresponding lead compound **II**. The generally reduced potency of the new derivatives may result from replacing the relatively rigid and conformationally constrained pyrazole ring with more flexible and protonatable substituents, which may incur greater entropic and desolvation penalties upon binding. However, the impact of this modification was dependent on the substitution pattern of the benzimidazole ring. Indeed, the additional hydrophobic contribution provided by the 5,6-dichloro substitution appears to compensate for, and in some cases, overcome, the energetic cost associated with the more flexible basic R_1_ moieties.

In contrast to the lead compounds, among which the most active was the 5-cyano derivative **IV** [37], four out of six 5,6-dichloro substituted derivatives in the new series (**12**, **20**, **24** and **30**) exhibited higher activity than the corresponding 5-cyano analogues. The only exceptions were derivatives **3** and **7**, bearing piperazine and piperidine residues, respectively, which were the least active in their respective subset. The influence of the solvent-exposed R_1_ region was particularly evident within the 5,6-dichloro-substituted set. The unsubstituted piperidine derivative **7** displayed an IC_50_ value of 1.66 μM, whereas functionalization of the 4-position of the piperidine ring with hydroxy, carboxamide, and aminomethyl groups afforded compounds **12**, **16**, and **20**, which were approximately 2.7-, 4.7-, and 9.7-fold more potent, respectively. This trend suggests that the introduction of a suitably oriented hydrogen-bond-donating or -accepting group at this position enables additional interactions in the solvent-exposed region. The good potency of **20** (IC_50_ = 0.172 μM) may be related to the terminal primary amine, which combines hydrogen-bond-donating ability and potential electrostatic interactions with sufficient conformational flexibility to reach a complementary interaction site, as evidenced by molecular docking studies predicting a hydrogen bond between the protonated amine and Asp91 (Section 2.4). The shorter and more conformationally constrained hydroxy and carboxamide groups of **12** and **16**, respectively, may not achieve the same optimal interaction geometry. The potency of the morpholine derivative **24** (IC_50_ = 0.223 μM) further supports the importance of an appropriate balance between polarity, basicity, and conformational organization in the solvent-exposed region. The morpholine ring introduces an additional hydrogen-bond acceptor while retaining a relatively well-defined orientation and lower basicity than piperazine or piperidine.

**Figure 3:**
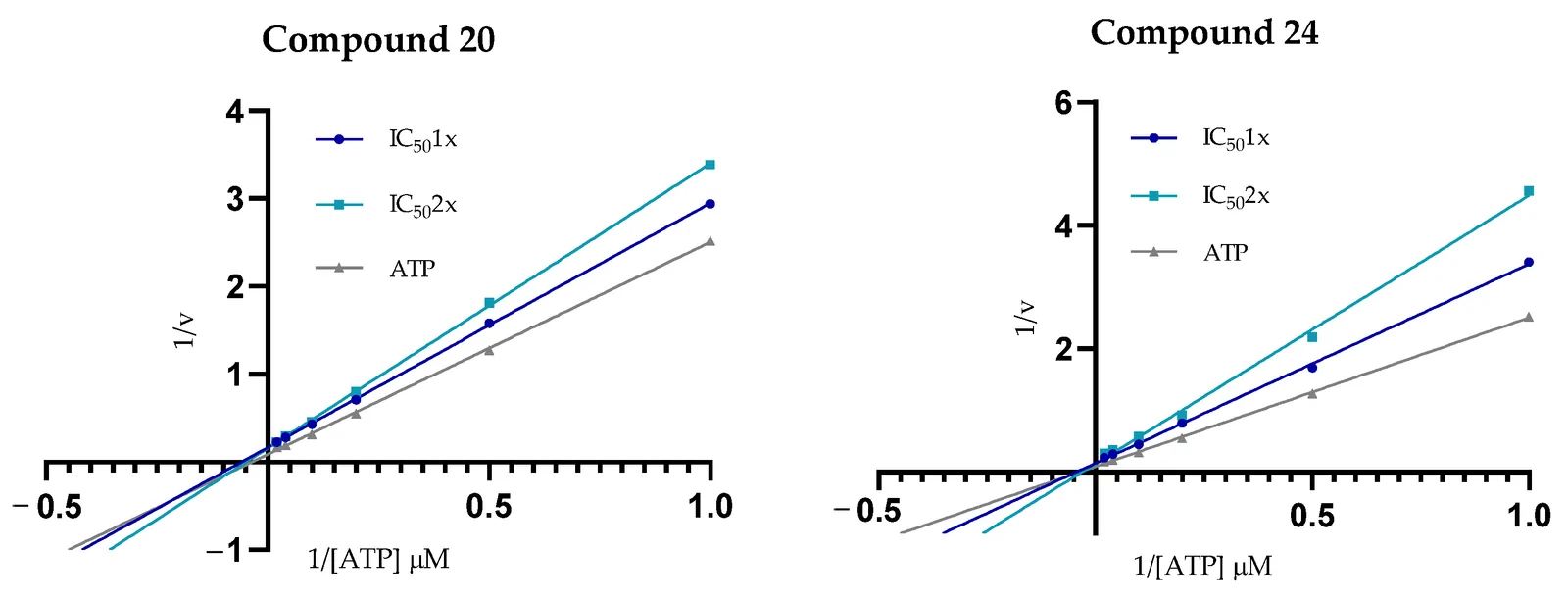
Lineweaver–Burk plots of compounds **20** and **24** for the evaluation of ATP-competitive inhibition of CK1δ. The reciprocal reaction velocity (1/V) was plotted against the reciprocal ATP concentration (1/[ATP]) in the absence of inhibitor (ATP) and in the presence of compounds **20** and **24** at 1× IC_50_ and 2× IC_50_. Data are representative of two independent experiments, with each condition tested in technical duplicate.

For compounds **20** and **24**, ATP-competition experiments were performed confirming their ATP-competitive behaviour (Figure 3). Following the 5,6-dichloro substitution, the 5-cyano group generally proved to be the most advantageous. Indeed, with the exception of compounds **5** and **14**, bearing a piperazine and a 4-hydroxypiperidine moiety, respectively, the 5-cyano derivatives **9**, **18**, **22**, **28** and **32** displayed higher inhibitory activity than the corresponding 5-chloro (**6**, **15**, **19**, **27** and **29**) and 5 nitro (**8**, **17**, **21** and **31**) analogues. It is worth noting that, as observed for the 5,6-dichloro-substituted derivatives, the piperidin-4-ylmethanamine residue also afforded the highest potency among the novel 5-cyano derivatives (compound **22**, IC_50_ = 0.224 μM). Conversely, within the 5-chloro- and 5-nitro-substituted analogues, the piperazine moiety emerged as the most favourable R_1_ substituent, as derivatives **2** (IC_50_ = 1.11 μM) and **4** (IC_50_ = 0.647 μM) were the most potent compounds in the respective set.

Finally, insertion of an aminoethyl spacer between the morpholine ring and the benzimidazole-2-acetamido scaffold reduced potency. Compounds **27** and **28** were approximately 3.7- and 1.4-fold less potent than the corresponding directly linked morpholine derivatives **23** and **26**, respectively. The additional linker may increase conformational flexibility, thereby increasing the entropic cost of binding and displacing the morpholine heteroatoms from their optimal interaction region.

### 2.3. In Silico ADME Prediction

Lead compounds **I**–**IV** and derivatives exhibiting a favourable CK1δ inhibition profile (**2**, **4**, **6**–**9**, **11**–**20** and **22**–**30**) were assessed through in silico analysis to evaluate their drug-likeness profiles based on absorption, distribution, metabolism, and excretion (ADME) properties which were predicted using the web program SwissADME (http://www.swissadme.ch, accessed on 11 November 2025) [42]. The predicted data for the selected compounds are summarized in the Supplementary Materials.

Overall, the investigated derivatives displayed favourable ADME profiles. Except for derivative **9**, all compounds obeyed Lipinski’s rule of five, supporting their drug-likeness. Furthermore, they showed a good likelihood of achieving at least 10% oral bioavailability in rats or exhibiting measurable Caco-2 permeability. SwissADME flags molecular fragments associated with potential false-positive biological responses (PAINS, Pan Assay Interference Structures); notably, none of the evaluated compounds triggered PAINS alerts. The topological polar surface area (TPSA) was below 140 Å^2^ for all derivatives, indicating favourable membrane permeability, with the exception of compounds **9** and **17**.

**Figure 4:**
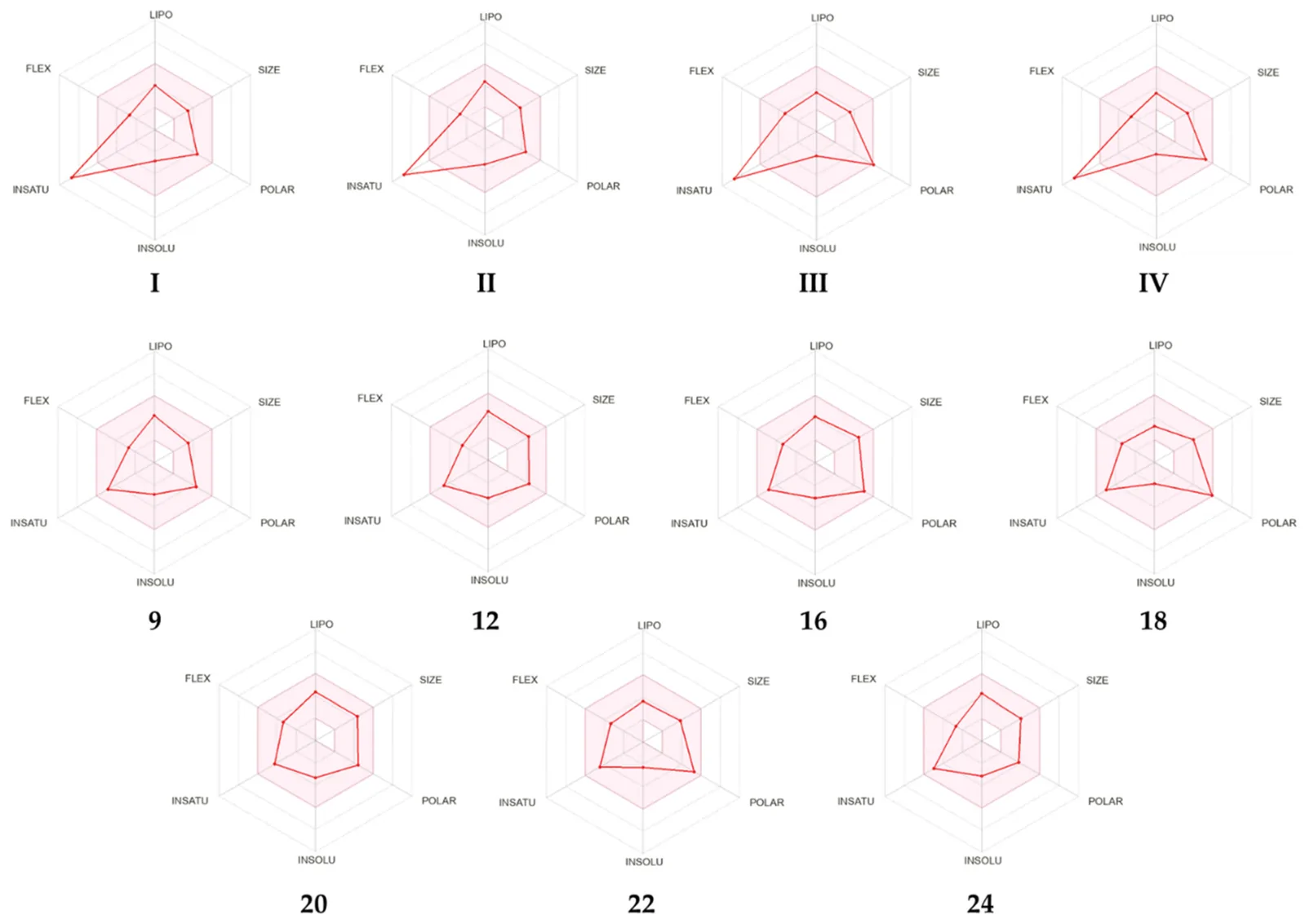
Bioavailability radars. LIPO = lipophilicity (XLOGP3 between −0.7 and 5.0); SIZE (molecular weight between 150 and 500 g/mol); POLAR = polarity (TPSA between 20 and 130 Å^2^); INSOLU = solubility (log S not higher than 6); INSATU = saturation (fraction of carbons in the sp^3^ hybridization not less than 0.25); FLEX = flexibility (no more than nine rotatable bonds).

Figure 4 shows bioavailability radars of the most potent CK1δ inhibitors of the series (derivatives **9**, **12**, **16**, **18**, **20**, **22** and **24**) and of the lead compounds **I**–**IV**. Bioavailability radars depict the drug-likeness of the analysed compounds, with each vertex representing a key physicochemical parameter (lipophilicity, size, polarity, solubility, flexibility, and saturation) relevant to oral bioavailability. The pink area indicates the optimal space for drug-like properties. This analysis highlights a clear difference between the lead compounds **I**–**IV** and the newly synthesized derivatives (Figure 4), indicating that the applied structural modifications successfully improved the overall drug-likeness profile of the new derivatives. In particular, compounds **I**–**IV** fall outside the optimal pink region in the saturation domain, indicating an excessive level of unsaturation. In contrast, all newly synthesized compounds fall entirely within the optimal region, reflecting a favourable balance of physicochemical descriptors relevant to oral bioavailability, with the exception of derivative **17** (see Supplementary Materials), whose increased polarity places it outside the recommended range.

**Figure 5:**
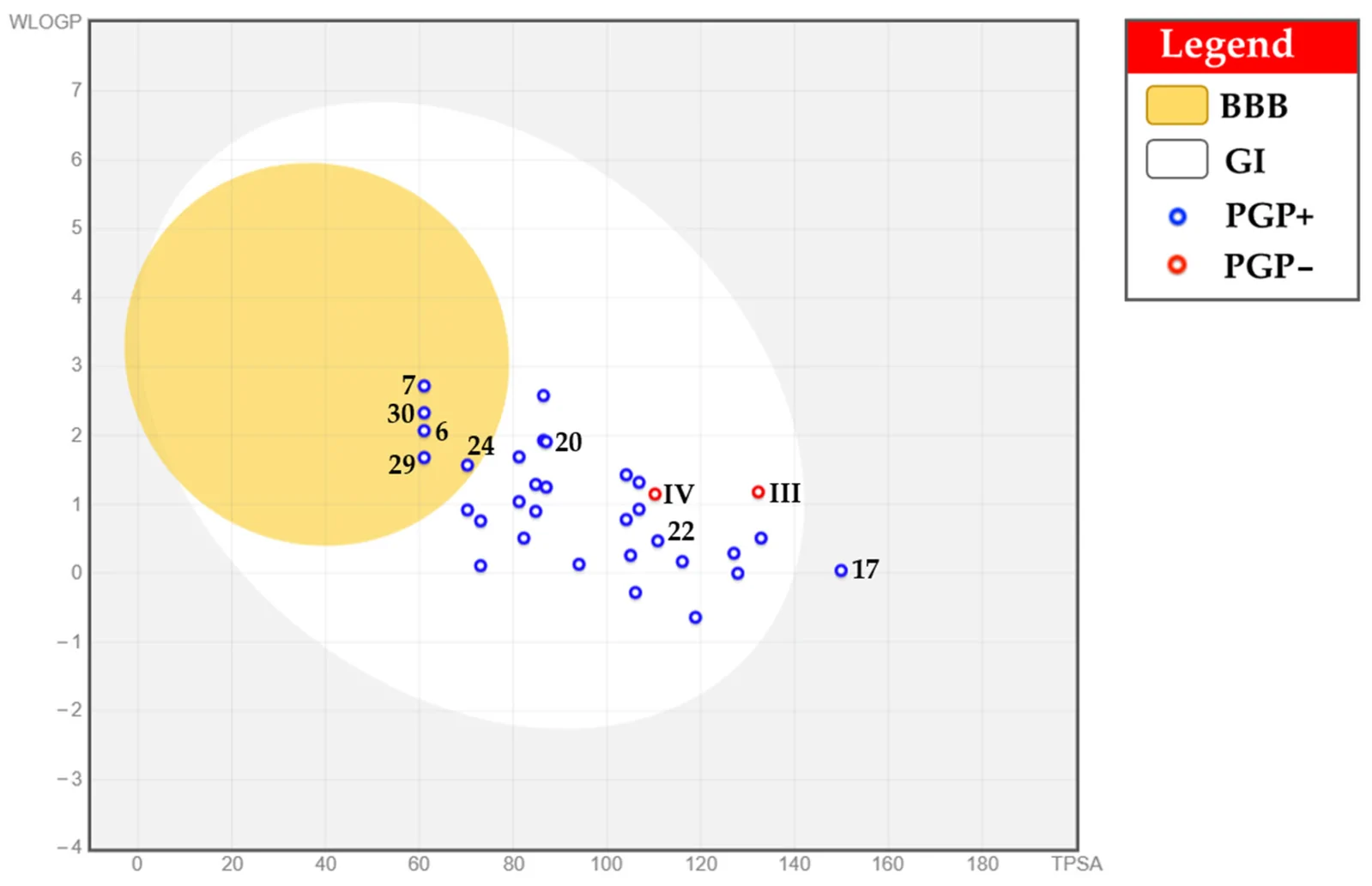
BOILED-Egg model representation.

Finally, the BOILED-Egg (Brain Or IntestinaL EstimateD) method (Figure 5) enables the simultaneous prediction of two key in vivo ADME parameters: passive gastrointestinal (GI) absorption and blood–brain barrier penetration (BBB). In agreement with the previously discussed physicochemical and ADME descriptors, all investigated derivatives, except compound **17**, were predicted to have good passive GI absorption (Figure 5), supporting a favourable oral bioavailability profile consistent with their TPSA values.

The poor GI permeation for derivative **17** is likely due to its higher polar surface area, which may hinder passive diffusion. Moreover, derivatives **6**, **7**, **24**, **29** and **30** were predicted to cross the blood–brain barrier, a desirable property for compounds targeting neurodegenerative diseases. However, except for lead compounds **III** and **IV**, derivatives were predicted to be P-glycoprotein (P-gp) substrates, which may potentially limit their effective gastrointestinal absorption and brain penetration despite the favourable passive permeability profile.

### 2.4. Molecular Modelling Studies

To rationalize the biological profile of the newly synthesized benzimidazol-2-acetamide derivatives, all compounds were docked into the ATP-binding site of CK1δ using the same structure-based workflow previously validated for the original series [37]. The crystallographic structure 5OKT was used because of its established suitability for this scaffold class and its ability to reproduce the canonical binding mode of benzimidazole-based CK1δ inhibitors. As observed in previously reported studies on benzimidazole-derivative series [37], the selected docking poses consistently preserved the characteristic bidentate hydrogen bond with the hinge residue Leu85, while maintaining the benzimidazole core deeply anchored within the hydrophobic ATP-binding cleft, in proximity of residues Ile15, Ile23, Ala36, Lys38, Tyr56, Pro66, Met82, Glu83, Leu135 and Ile148 (as reported in Video S1). Conserved crystallographic water molecules located near Tyr56 and Lys38 were also retained during the docking protocol because of their known role in mediating ligand recognition within the CK1δ binding site.

The newly designed derivatives were obtained by functionalizing the solvent-exposed region of the scaffold, with the aim of extending the interaction pattern beyond the canonical ATP-binding cavity while preserving the conserved hinge-binding motif. In particular, the introduction of additional polar and conformationally flexible substituents was expected to improve target engagement in the outer region of the pocket. However, despite maintaining the conserved recognition pattern observed for the previously reported inhibitors, the new compounds did not surpass the potency of the most active compound (compound **IV**) from the original series. This observation suggested that the newly introduced solvent-oriented moieties might not establish sufficiently stable interactions to provide a significant energetic advantage.

**Figure 6:**
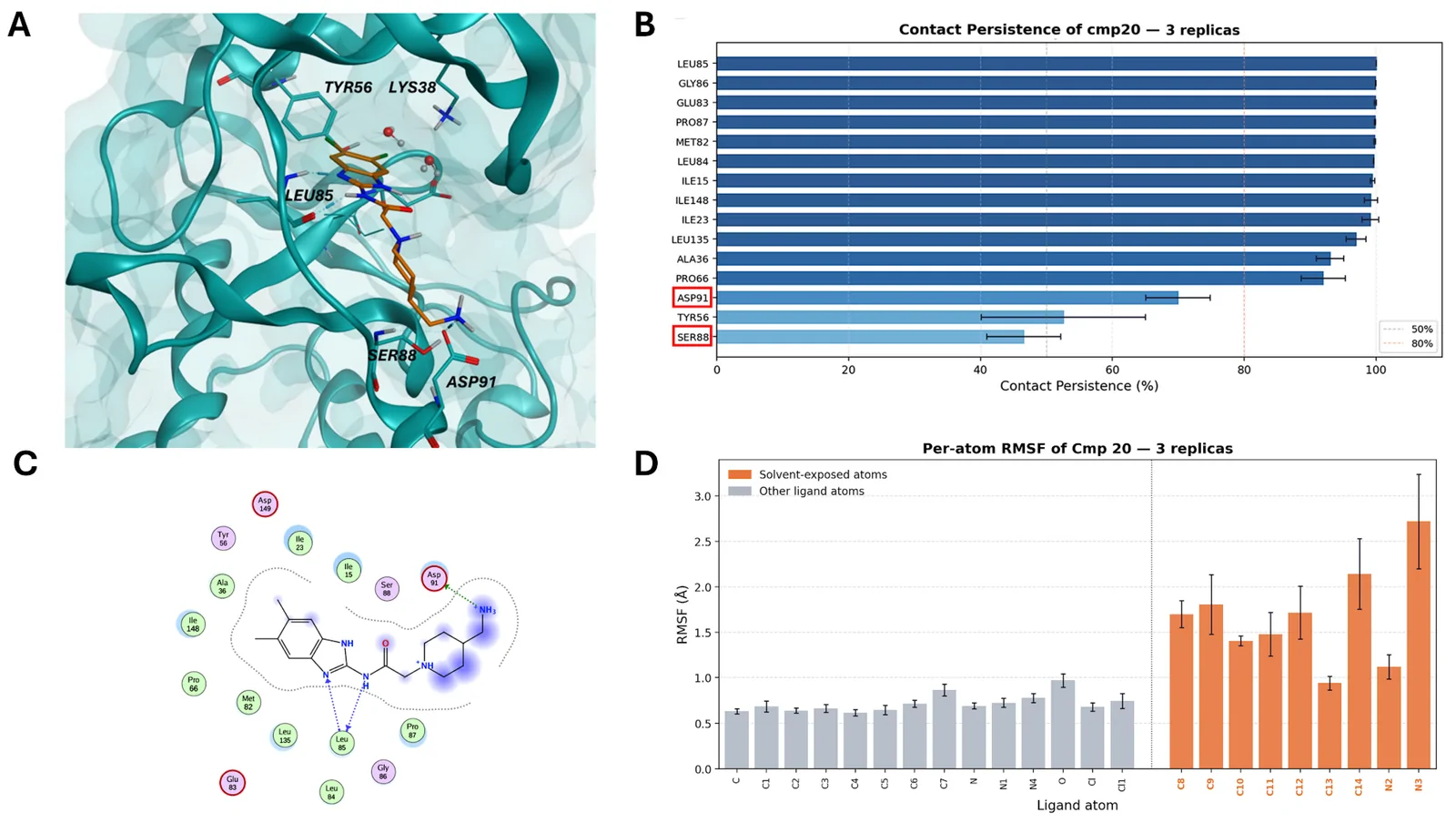
(**A**) Representative docking pose of compound **20** in the ATP-binding site, showing the conserved hinge interactions and the orientation of the solvent-exposed piperidin-4-ylmethanamine substituent toward Ser88 and Asp91. (**B**) Average ligand–protein contact persistence calculated over three independent 100 ns MD replicas. Residues with contact persistence >80% are shown in dark blue; those falling below this threshold (Asp91, 69.9%; Ser88, 46.5%), highlighted in red, correspond to the solvent-exposed region of the binding site. Error bars represent the standard deviation across replicas. (**C**) Two-dimensional ligand interaction diagram of compound **20** in the CK1δ binding site (docking pose): hydrogen bonds are drawn as dashed arrows, the residues lining the pocket are coloured according to their hydrophobic (green) or polar (violet) character, and the dotted contour delimits the solvent-accessible surface of the ligand. (**D**) Per-atom RMSF of compound **20**, computed over the three 100 ns MD replicas and reported as the mean over the replicas; error bars represent ±SD. Atoms are ordered from the benzimidazole core to the solvent-exposed piperidin-4-ylmethanamine substituent, whose atoms display the largest fluctuations and the largest inter-replica dispersion.

To investigate this hypothesis, compound **20**, representing the most potent derivative of the new series and bearing the piperidin-4-ylmethanamine substituent as the key structural modification introduced at the solvent-exposed edge of the binding site, was selected as a representative case for molecular dynamics (MD) simulations. As shown in Figure 6A, the docking pose of compound **20** within the CK1δ ATP-binding site (PDB: 5OKT) highlighted the canonical binding mode conserved across the series, with the benzimidazole core anchored in the hinge region through a bidentate hydrogen bond with Leu85 and additional hydrophobic contacts with the surrounding residues. The newly introduced piperidin-4-ylmethanamine substituent was oriented toward the solvent-exposed edge of the binding cleft, in proximity to Ser88 and Asp91, consistent with the design rationale of extending interactions beyond the canonical ATP pocket. The nature of the contacts established by compound **20** is summarized in the two-dimensional ligand interaction diagram reported in Figure 6C. The benzimidazole core is engaged in the bidentate hydrogen bond with the backbone of Leu85 and is enclosed in a markedly hydrophobic envelope contributed by Ile15, Ile23, Ala36, Pro66, Met80, Met82, Glu83, Leu84, Leu135 and Ile148, while the only polar contact detected beyond the ATP cleft is a single hydrogen bond between the protonated primary amine of the piperidin-4-ylmethanamine substituent and the side chain of Asp91. The solvent-accessibility contour further shows that this substituent lies almost entirely outside the pocket.

The docking pose of compound **20** was subjected to 100 ns molecular dynamics (MD) simulations performed in triplicate to assess the stability of the two structurally distinct regions of the ligand: the benzimidazole core, which establishes the conserved hinge interactions within the ATP-binding cleft, and the solvent-exposed portion, which was specifically designed to extend interactions beyond the canonical binding pocket.

The geometrical fluctuations of the ligand atoms were investigated by computing the per-atom root-mean-square fluctuation (RMSF) profile of the compound (Figure 6D). The atoms of the benzimidazole core, buried within the ATP cleft, fluctuate within a narrow range, whereas those belonging to the solvent-exposed piperidin-4-ylmethanamine substituent display markedly higher values, the terminal aminomethyl group being the most mobile portion of the molecule. The inter-replica average RMSF over the piperidin-4-ylmethanamine atoms amounts to 1.67 ± 0.20 Å, against an average value of 0.71 ± 0.04 Å for the rest of the molecule. The inter-replica average RMSD profile of the solvent-exposed moiety as compared to the initial docking conformation is reported in Figure S2, with an average RMSD of 2.14 ± 0.69 Å (Table 2).

This geometrical analysis was complemented by ligand–protein contact persistence analysis across the three replicas, which allowed us to directly evaluate whether the newly introduced substituent was capable of establishing a stable and reproducible interaction network at the outer boundary of the binding site.

Contact persistence analysis across the three replicas (average percentage of contacts) confirmed that the benzimidazole core maintained highly stable interactions with the hinge region and the surrounding hydrophobic residues throughout the simulations. In particular, Leu85 exhibited a contact persistence of ~100%, alongside other residues lining the ATP-binding cleft such as Gly86, Glu83, Pro87, Met82, Leu84, Ile15, Ile148, Ile23, and Leu135, all displaying contact persistence values exceeding 80% (Figure 6B). This high conservation of contacts across all replicas confirms that the core of the molecule maintains a well-defined and stable binding mode within the kinase active site. In contrast, residues located at the solvent-exposed boundary of the binding site displayed substantially lower and more variable contact persistence values. Asp91 maintained contact in only 69.9% of the frames, while Ser88 showed the lowest persistence of the entire contact profile, at 46.5% (Figure 6B, highlighted in red). Notably, both residues also exhibited the highest inter-replica variability, with standard deviations of 4.88% and 5.63%, respectively, indicating that the interactions between the solvent-exposed substituent and these residues are not only transient but also poorly reproducible across independent MD trajectories.

Taken together, these results indicate that, despite the stabilization of the benzimidazole core in the hinge region, the piperidin-4-ylmethanamine moiety fails to establish a persistent and reproducible interaction network at the solvent-exposed edge of the binding site. The high conformational mobility and inter-replica variability of this portion of the molecule provide a structural rationale for the absence of a significant improvement in inhibitory potency relative to the best compounds of the previously reported series **(I**–**V**), confirming that substituents targeting highly solvated and flexible regions of kinase binding sites are unlikely to translate into measurable affinity gains.

In parallel, to provide a comparative reference frame, the same simulation and analysis protocol was applied to a reference inhibitor, the structurally characterized reference CK1δ inhibitor **Bischof-5** (Figure 1), co-crystallized with the kinase in the structure 4TWC, which shares the benzimidazole hinge-binding motif with the present series but bears a more extended solvent-exposed substituent and is appreciably more potent (IC_50_ = 29 nM, against 0.172 μM for compound **20**). The corresponding analyses are reported in Figure S1 (Supplementary Materials). The reference compound reproduces the hydrophobic core described above for compound **20**, but differs at the solvent-exposed boundary of the site: the contact with Asp91, retained in only 69.9% of the frames for compound **20**, exceeds 95% persistence for **Bischof-5**, whose trifluoromethoxyphenyl portion is held at the outer rim by additional hydrophobic interactions involving Leu92, Phe95, Leu293 and Phe295, whereas the piperidin-4-ylmethanamine substituent of compound **20** engages the same region only through a single polar contact. Ser88 is the least persistent contact in both profiles (about 50% for **Bischof-5**), indicating that this residue is intrinsically poorly engaged in this region of the site rather than specifically neglected by the new derivatives. The descriptors extracted from the trajectories are collected in Table 2: the solvent-exposed region of the reference compound appears to fluctuate slightly less as compared to compound **20**, as exemplified by the lower average RMSD and RMSF values of the substituent atoms (RMSD 1.54 ± 0.28 Å and 2.14 ± 0.69 Å; RMSF 1.21 ± 0.13 Å and 1.67 ± 0.20 Å, for **Bischof-5** and compound **20**, respectively), while the average number of hydrogen bonds occurring every frame between the substituent and the protein is almost the same (0.46 ± 0.09 and 0.38 ± 0.02, for **Bischof-5** and compound **20**, respectively). Moreover, the inter-replica standard deviation is more than twice as large in the case of compound **20**, whose solvent-exposed substituent therefore explores a wider conformational space and does so differently in each replica. The RMSD time profiles of the solvent-exposed moiety of the two compounds, collected in Figures S2 and S3 (Supplementary Materials), illustrate this difference: the RMSD profile of **Bischof-5** substituent oscillates within a narrow band around its mean for the whole trajectory, whereas that of compound **20** broadens progressively and, over the last 20 ns, exhibits sample values between 3 and 5 Å with an inter-replica span extending beyond 8 Å. This dynamic behaviour may contribute, among other factors, to the potency difference between the two compounds (IC_50_ = 0.172 μM and 29 nM, respectively).

### 2.5. Cell Viability Assay

**Figure 7:**
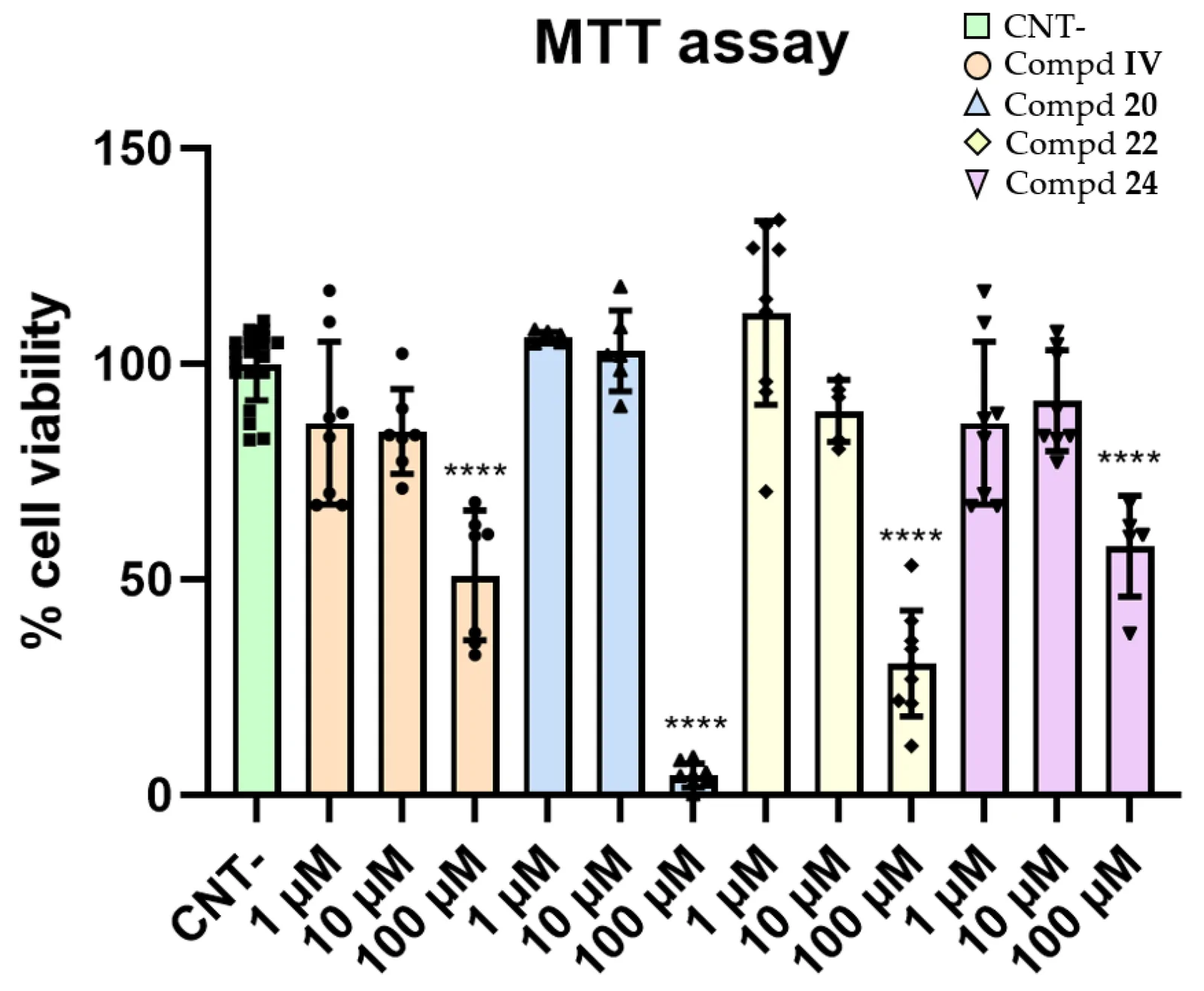
Cell viability (%) of treated cells relative to untreated control (CNT-), as determined by the MTT assay. Each column represents the mean ± SD. Statistical analysis was performed using one-way ANOVA: **** *p* ≤ 0.0001.

To complement the enzyme inhibition data with a preliminary cellular evaluation, compound **IV**, the most potent CK1δ inhibitor of the lead series [36], and derivatives **20**, **22**, and **24**, the most active compounds of the new series, were evaluated in human SH-SY5Y neuroblastoma cells. Although derived from a neuroblastoma, SH-SY5Y cells were employed in this study as a neuronal model. Indeed, these cells are widely used as experimental models of neurological disorders, including AD, PD, and ALS [43,44,45,46,47], in the pathogenesis of which CK1δ dysregulation may play a role [15,19,21,23,24]. Moreover, *CSNK1D*, the gene encoding CK1δ, is expressed at moderate-to-high levels in a wide range of human tissues and cell lines, including SH-SY5Y cells [22,25], further supporting the relevance of this cellular model. Accordingly, the experiments were designed to determine the effects of the compounds on cell viability and provide a preliminary assessment of their cellular tolerability [43]. Cell viability was measured using the MTT (3-(4,5-dimethylthiazol-2-yl)-2,5-diphenyltetrazolium bromide) assay, and the results are reported in Figure 7.

Derivatives **IV**, **20**, **22** and **24** were well tolerated at concentrations up to 10 μM, with no significant effects on cell viability. A marked reduction in viability was observed only at the highest tested concentration (100 μM) for all compounds. At this concentration, compound **IV** reduced cell viability to approximately 50%, whereas compounds **22** and **24** retained approximately 30% and 60% cell viability, respectively. Derivative **20** markedly reduced cell viability at 100 μM, with viability approaching 0%.

## 3. Materials and Methods

### 3.1. Chemistry

All the commercially available reagents and solvents were used as purchased from Merck without further purification. Analytical silica gel plates (Merck F254) and silica gel 60 (Merck Life Science S.r.l., Milan, Italy, 70–230 mesh) were used for analytical TLC and column chromatography, respectively. All melting points were determined on a Gallenkamp melting point apparatus (Gallenkamp, Loughborough, UK) and were uncorrected. Compounds were named following IUPAC rules as applied by ChemDrawUtra 9.0. Elemental analyses were performed with a Flash E1112 Thermofinnigan elemental analyser for C, H, and N (Termofisher, Milan, Italy), and the results were within ±0.4% of the theoretical values (Table S1, Supplementary Materials). All final compounds revealed a purity of no less than 95%. NMR spectra were recorded on a Bruker Avance 400 spectrometer (400 MHz for ^1^H NMR and 100 MHz for ^13^C NMR, Bruker Italia, S.r.l., Milan, Italy). The chemical shifts are reported in δ (ppm) and are relative to the central peak of the solvent, which was CDCl_3_ or DMSO-*d*_6_. The following abbreviations are used: s = singlet, d = doublet, t = triplet, q = quartet, m = multiplet, br = broad, and ar = aromatic protons. Scanned ^1^H NMR and ^13^C NMR spectra of derivatives **1**–**32** are reported in the Supplementary Materials. The exchangeable NH signals of some derivatives (such as compounds **1**, **3**, and **4**) were not clearly detected in the ^1^H NMR spectra recorded in DMSO-*d*_6_, most likely because of substantial line broadening resulting from rapid proton exchange with residual water and benzimidazole prototropic tautomerism, possibly facilitated by the basic piperazine moiety.

#### 3.1.1. General Procedure for the Synthesis of N-(Benzimidazol-2-yl)-2-(piperazin-1-yl)acetamide Derivatives **1**–**32**

The suitable amine (piperazine, piperidine, piperidin-4-ol, piperidine-4-carboxamide, piperidin-4-ylmethanamine, morpholine, 2-morpholinoethan-1-amine and pyrrolidine (2 mmol) and K_2_CO_3_ (1.5 mmol) were added to a solution of the benzimidazol-2-chloroacetamide derivatives **38**–**42** (1 mmol) in dry CH_3_CN (10 mL). The mixture was heated at reflux for 4 h under nitrogen atmosphere; then, the solvent was evaporated under reduced pressure. The residue was treated with H_2_O (15 m), and the mixture was acidified with 1M HCl solution until pH = 8. The obtained solid was collected by filtration and purified by recrystallization or silica gel column chromatography.

N-(Benzimidazol-2-yl)-2-(piperazin-1-yl)acetamide **1**. Yield 56%; m.p. = 233–235 °C. Purified via silica gel column chromatography, eluent DCM 8/MeOH 2. ^1^H NMR (DMSO-*d*_6_) 11.61 (br s, 1H, NH), 7.45–7.44 (m, 2H, ar), 7.09–7.08 (m, 2H, ar), 3.22 (s, 2H, CH_2_), 2.73–2.71 (m, 4H, 2CH_2_), 2.47–2.44 (m, 4H, 2CH_2_). ^13^C-NMR (DMSO-*d*_6_) 170.06, 146.47, 121.50, 61.90, 54.53, 46.11.

N-(5-Chlorobenzimidazol-2-yl)-2-(piperazin-1-yl)acetamide **2**. Yield 53%; m.p. = 290–292 °C. Purified via silica gel column chromatography, eluent DCM 8/MeOH 2. ^1^H NMR (DMSO-*d*_6_) 12.25 (br s, 1H, NH), 11.40 (br s, 1H, NH), 7.48–7.44 (m, 1H, ar), 7.12 (d, 1H, ar, J = 8.4 Hz), 3.40 (s, 2H, CH_2_), 3.11–3.08 (m, 4H, 2CH_2_), 2.78–2.77 (m, 4H, 2CH_2_).

N-(5,6-Dichlorobenzimidazol-2-yl)-2-(piperazin-1-yl)acetamide **3**. Yield 50%; mp > 300 °C. Purified via silica gel column chromatography, eluent DCM 8/MeOH 2. ^1^H NMR (DMSO-*d*_6_) 12.28 (br s, 1 H, NH), 11.51 (br s, 1H, NH), 7.67 (s, 2H, ar), 3.41 (s, 2H, CH_2_), 3.12–3.09 (m, 4H, 2CH_2_), 2.79–2.77 (m, 4H, 2CH_2_). ^13^C NMR (DMSO-*d*_6_) 170.09, 148.56, 60.33, 49.67, 43.38.

N-(5-Nitro-1-benzimidazol-2-yl)-2-(piperazin-1-yl)acetamide **4**. Yield 22%; mp > 300 °C. Purified via silica gel column chromatography, eluent DCM 8/MeOH 2. ^1^H NMR (DMSO-*d*_6_) 10.43 (br s, 2H, 2NH), 8.34 (s, 1H, ar), 8.06 (d, 1H, ar, J = 8.6 Hz), 7.60 (d, 1H, ar, J = 8.6 Hz), 3. 30 (br s, 2H, CH_2_), 2.80–2.76 (m, 4H, 2CH_2_), 2.68–2.65 (m, 4H, 2CH_2_).

N-(5-Cyano-1H-benzimidazol-2-yl)-2-(piperazin-1-yl)acetamide **5**. Yield 44%; m.p. = 205–207 °C. Purified via silica gel column chromatography, eluent DCM 8.2/MeOH 1.8/NH_4_OH 0.18. ^1^H NMR (DMSO-*d*_6_) 7.89 (s, 1H, ar), 7.60 (d, 1H, ar, J = 8.3 Hz), 7.50 (d, 1H, ar, J = 8.3 Hz), 3.27 (br s, 2H, CH_2_), 2.75–2.73 (m, 4H, 2CH_2_), 2.52–2.48 (m, 4H, 2CH_2_). ^13^C NMR (DMSO-*d*_6_) 170.47, 149.18, 125.42, 120.69, 103.25, 61.79, 54.34, 46.01.

N-(5-Chloro-1-benzimidazol-2-yl)-2-(piperidin-1-yl)acetamide **6**. Yield 50%; m.p. = 207–209 °C. Purified by recrystallization from EtOH. ^1^H NMR (DMSO-*d*_6_) 11.74 (br s, 1H, NH), 7.48 (s, 1H, ar), 7.47 (d, 1H, ar, J = 2.2 Hz); 7.44 (d, 1 H, ar, J = 8.3 Hz), 7.11 (dd, 1H, ar, J = 8.3 Hz, J = 2.2 Hz), 3.24 (s, 2H, CH_2_), 2.53–2.50 (m, 4H, 2CH_2_), 1.58–1.52 (m, 4H, 2CH_2_), 1.43–1.41 (m, 2H, CH_2_). ^13^C NMR (DMSO-*d*_6_) 170.41, 147.63, 125.74, 121.56, 61.85, 54.28, 26.05, 24.00.

N-(5,6-Dichloro-1-benzimidazol-2-yl)-2-(piperidin-1-yl)acetamide **7**. Yield 52%; m.p. = 247–249 °C. Purified by recrystallization from EtOH. ^1^H NMR (DMSO-*d*_6_) 12.36 (s, 1H, NH), 12.28 (br s, 1H, NH), 7.66 (s, 2H, ar), 3.25 (s, 2H, CH_2_), 2.52–2.49 (m, 4H, 2CH_2_), 1.56–1.50 (m, 4H, 2CH_2_), 1.41–1.37 (m, 2H, CH_2_). ^13^C NMR (DMSO-*d*_6_) 170.62, 148.53, 61.80, 54.25, 26.01, 23.97.

N-(5-Nitro-1H-benzimidazol-2-yl)-2-(piperidin-1-yl)acetamide **8**. Yield 58%; m.p. = 239–241 °C. Purified via silica gel column chromatography, eluent DCM 9.6/MeOH 0.4. ^1^H NMR (DMSO-*d*_6_) 12.69 (s, 1H, NH), 11.46 (br s, 1H, NH), 8.33 (s, 1H, ar), 8.06 (d, 1H, ar, J = 8.8 Hz), 7.60 (d, 1H, ar, J = 8.8 Hz), 3.28 (s, 2H, CH_2_), 2.54–2.50 (m, 4H, 2CH_2_), 1.58–1.52 (m, 4H, 2CH_2_), 1.45–1.43 (m, 2H, CH_2_).

N-(5-Cyano-1H-benzimidazol-2-yl)-2-(piperidin-1-yl)acetamide **9**. Yield 68%; m.p. = 210–212 °C. Purified via silica gel column chromatography, eluent DCM 9.5/MeOH 0.5. ^1^H NMR (DMSO-*d*_6_) 12.54 (s, 1H, NH), 11.37 (br s, 1H, NH), 7.88 (s, 1H, ar), 7.60 (d, 1H, ar, J = 8.2 Hz), 7.50 (d, 1H, ar, J = 8.2 Hz), 3.27 (s, 2H, CH_2_), 2.53–2.51 (m, 4H, 2CH_2_), 1.57–1.52 (m, 4H, 2CH_2_), 1.45–1.42 (m, 2H, CH_2_). ^13^C NMR (DMSO-*d*_6_) 170.69, 120.66, 61.81, 54.24, 26.03, 23.99.

N-(Benzimidazol-2-yl)-2-(4-hydroxypiperidin-1-yl)acetamide **10**. Yield 53%; m.p. = 216–217 °C. Purified via silica gel column chromatography, eluent DCM 8/MeOH 2. ^1^H NMR (DMSO-*d*_6_) 12.06 (br s, 1H, NH), 11.14 (br s, 1H, NH), 7.44–7.43 (m, 2H, ar), 7.10–7.08 (m, 2H, ar), 4.61 (br s, 1H, OH), 3.50–3.45 (m, 1H, CH), 3.25 (s, 2H, CH_2_), 3.80–3.77 (m, 2H, CH_2_), 2.32–2.28 (m, 2H, CH_2_), 1.75–1.73 (m, 2H, CH_2_), 1.50–1.44 (m, 2H, CH_2_). ^13^C NMR (DMSO-*d*_6_) 170.25, 146.48, 121.50, 66.01, 61.19, 51.30, 34.84.

N-(5-Chlorobenzimidazol-2-yl)-2-(4-hydroxypiperidin-1-yl)acetamide **11**. Yield 35%; m.p. = 220–221 °C. Purified via silica gel column chromatography, eluent DCM 8/MeOH 2. ^1^H NMR (DMSO-*d*_6_) 12.26 (br s, 1H, NH), 11.20 (br s, 1H, NH), 7.47–7.45 (m, 2H, ar), 7.11 (dd, 1H, ar, J = 8.3 Hz), 4.60 (br s, 1H, OH), 3.49–3.46 (m, 1H, CH), 3.27 (s, 2H, CH_2_), 2.80–2.78 (m, 2H CH_2_), 2.33–2.30 (m, 2H, CH_2_), 1.75–1.72 (m, 2H, CH_2_), 1.46–1.44 (m, 2H, CH_2_).

N-(5,6-Dichlorobenzimidazol-2-yl)-2-(4-hydroxypiperidin-1-yl)acetamide **12**. Yield 46%; m.p. = 260–261 °C. Purified via silica gel column chromatography, eluent DCM 8/MeOH 2. ^1^H NMR (DMSO-*d*_6_) 11.74 (br s, 1H, NH), 7.65 (s, 2H, ar), 4.59 (br s, 1H, OH), 3.49–3.46 (m, 1H, CH), 3.26 (s, 2H, CH_2_), 2.79–2.76 (m, 2H, CH_2_), 2.30–2.27 (m, 2H, CH_2_), 1.74–1.71 (m, 2H, CH_2_), 1.46–1.42 (m, 2H, CH_2_). ^13^C NMR (DMSO-*d*_6_) 170.58, 148.69, 123.64, 66.18, 61.13, 51.24, 34.81.

2-(4-Hydroxypiperidin-1-yl)-N-(5-nitro-1H-benzimidazol-2-yl)acetamide **13**. Yield 55%; m.p. = 248–250 °C. Purified via silica gel column chromatography, eluent DCM 9.5/MeOH 0.5. ^1^H NMR (DMSO-*d*_6_) 12.70 (br s, 1H, NH), 8.34 (s, 1H, ar), 8.06 (d, 1H, ar, J = 8.8 Hz), 7.66–7.60 (m, 1H, ar), 4.57 (br s, 1H, OH), 3.51–3.45 (m, 1H, CH), 3.30 (s, 2H, CH_2_), 2.83–2.78 (m, 2H, CH_2_), 2.36–2.30 (m, 2H, CH_2_), 1.77–1.74 (m, 2H CH_2_),1.49–1.42 (m, 2H CH_2_).

N-(5-Cyano-1H-benzimidazol-2-yl)-2-(4-hydroxypiperidin-1-yl)acetamide **14**. Yield 57%; m.p. = 256–258 °C. Purified via silica gel column chromatography, eluent DCM 8.8/MeOH 1.2/NH_4_OH 0.12. ^1^H NMR (DMSO-*d*_6_) 12.53 (br s, 1H, NH), 11.37 (br s, 1H, NH), 7.88 (s, 1H, ar), 7.60 (d, 1H, ar, J = 8.2 Hz), 7.50 (d, 1H, ar, J = 8.2 Hz), 4.56 (br s, 1H, OH), 3.50–3.45 (m, 1H, CH), 3.28 (s, 2H, CH_2_), 2.81–2.77 (m, 2H, CH_2_), 2.34–2.28 (m, 2H, CH_2_), 1.76–1.72 (m, 2H CH_2_), 1.49–1.41 (m, 2H CH_2_). ^13^C NMR (DMSO-*d*_6_) 170.69, 120.67, 66.21, 61.10, 51.23, 34.81.

1-(2-((5-Chloro-1H-benzimidazol-2-yl)amino)-2-oxoethyl)piperidine-4-carboxamide **15**. Yield 10%; m.p. = 278–280 °C. Purified via silica gel column chromatography, eluent DCM 9/MeOH 1/NH_4_OH 0.1. ^1^H NMR (DMSO-*d*_6_) 12.24 (br s, 1H, NH), 11.18 (br s, 1H, NH), 7.47–7.44 (m, 2H, ar), 7.22 (br s, 1H, NH_2_), 7.11 (d, 1H, ar, J = 8.5 Hz), 6.73 (br s, 1H, NH_2_), 3.27 (s, 2H, CH_2_), 2.93–2.90 (m, 2H, CH_2_), 2.25–2.18 (td, 2H, CH_2_, J = 11.4 Hz, J = 2.8 Hz), 2.10–2.03 (m, 1H, CH), 1.72–1.58 (m, 4H, 2CH_2_).

1-(2-((5,6-Dichloro-1H-benzimidazol-2-yl)amino)-2-oxoethyl)piperidine-4-carboxamide **16**. Yield 21%; m.p. = 278–280 °C. Purified via silica gel column chromatography, eluent DCM 9.2/MeOH 0.8/NH_4_OH 0.08. ^1^H NMR (DMSO-*d*_6_) 12.35 (br s, 1H, NH), 11.32 (br s, 1H, NH), 7.66 (s, 2H, ar), 7.22 (br s, 1H, NH_2_), 6.72 (br s, 1H, NH_2_), 3.28 (s, 2H, CH_2_), 2.92–2.90 (m, 2H, CH_2_), 2.25–2.18 (td, 2H, CH_2_, J = 11.4 Hz, J = 2.6 Hz), 2.09–2.04 (m, 1H, CH), 1.70–1.59 (m, 4H, 2CH_2_). ^13^C NMR (DMSO-*d*_6_): 176.93, 170.54, 148.57, 118.56, 113.23, 61.30, 53.06, 41.66, 28.99.

1-(2-((5-Nitro-1H-benzimidazol-2-yl)amino)-2-oxoethyl)piperidine-4-carboxamide **17**. Yield 15%; m.p. = 298–300 °C. Purified via silica gel column chromatography, eluent DCM 9/MeOH 1/NH_4_OH 0.1. ^1^H NMR (DMSO-*d*_6_) 12.71 (br s, 1H, NH), 11.41 (br s, 1H, NH), 8.36 (s, 2H, ar), 8.60 (d, 2H, ar, J = 8.8 Hz), 7.61–7.60 (m, 2H, ar), 7.22 (br s, 1H, NH_2_), 6.73 (br s, 1H, NH_2_), 3.38 (s, 2H, CH_2_), 2.94–2.91 (m, 2H, CH_2_), 2.26–2.20 (td, 2H, CH_2_, J = 11.2 Hz, J = 2.8 Hz), 2.10–2.06 (m, 1H, CH), 1.71–1.62 (m, 4H, 2CH_2_).

1-(2-((5-Cyano-1H-benzimidazol-2-yl)amino)-2-oxoethyl)piperidine-4-carboxamide **18**. Yield 14%; m.p. = 292–294 °C. Purified via silica gel column chromatography, eluent DCM 9.2/MeOH 0.8/NH_4_OH 0.08. ^1^H NMR (DMSO-*d*_6_) 12.56 (br s, 1H, NH), 11.38 (br s, 1H, NH), 7.88 (s, 2H, ar), 8.60 (d, 2H, ar, J = 8.2 Hz), 7.51 (d, 2H, ar, J = 8.2 Hz), 7.22 (br s, 1H, NH_2_), 6.73 (br s, 1H, NH_2_), 3.30 (s, 2H, CH_2_), 2.93–2.90 (m, 2H, CH_2_), 2.25–2.19 (td, 2H, CH_2_, J = 11.3 Hz, J = 2.8 Hz), 2.11–2.03 (m, 1H, CH), 1.71–1.59 (m, 4H, 2CH_2_). ^13^C NMR (DMSO-*d*_6_) 176.93, 170.66, 120.67, 61.32, 53.07, 41.67, 28.99.

2-(4-(Aminomethyl)piperidin-1-yl)-N-(5-chloro-1H-benzimidazol-2-yl)acetamide **19**. Yield 21%; m.p. = 195–197 °C. Purified via silica gel column chromatography, eluent DCM 8.8/MeOH 1.2/NH_4_OH 0.12. ^1^H NMR (DMSO-*d*_6_) 7.47–7.43 (m, 2H, ar), 7.11 (d, 2H, ar, J = 8.4 Hz), 3.26 (s, 2H, CH_2_), 2.91–2.88 (m, 2H, CH_2_), 2.43–2.42 (m, 2H, CH_2_), 2.20–2.15 (m, 2H, CH_2_), 1.69–1.67 (m, 2H, CH_2_), 1.18–1.15 (m, 3H, CH and CH_2_). ^13^C NMR (DMSO-*d*_6_) 170.54, 147.67, 125.73, 121.54, 115.66, 114.47, 61.52, 53.65, 48.14, 38.93, 30.23.

2-(4-(Aminomethyl)piperidin-1-yl)-N-(5,6-dichloro-1H-benzimidazol-2-yl)acetamide **20**. Yield 16%; m.p. = 198–200 °C. Purified via silica gel column chromatography, eluent DCM 9.2/MeOH 0.8/NH_4_OH 0.08. ^1^H NMR (DMSO-*d*_6_) 7.65 (s, 2H, ar), 3.26 (s, 2H, CH_2_), 2.91–2.88 (m, 2H, CH_2_), 2.45–2.44 (m, 2H, CH_2_), 2.20–2.15 (m, 2H, CH_2_), 1.69–1.67 (m, 2H, CH_2_), 1.20–1.16 (m, 3H, CH and CH_2_). ^13^C NMR (DMSO-*d*_6_) 170.57, 148.88, 137.21, 123.52, 115.84, 61.50, 53.58, 47.88, 38.60, 30.18.

2-(4-(Aminomethyl)piperidin-1-yl)-N-(5-nitro-1H-benzimidazol-2-yl)acetamide **21**. Yield 10%; m.p. = 204–206 °C. Purified via silica gel column chromatography, eluent DCM 8.4/MeOH 1.6/NH_4_OH 0.16. ^1^H NMR (DMSO-*d*_6_) 8.01 (s, 2H, ar), 8.01 (d, 2H, ar, J = 8.8 Hz), 7.55 (d, 2H, ar, J = 8.8 Hz), 3.28 (s, 2H, CH_2_), 2.93–2.90 (m, 2H, CH_2_), 2.21–2.16 (m, 2H, CH_2_), 1.70–1.68 (m, 2H, CH_2_), 1.24- 1.21 (m, 3H, CH and CH_2_). ^13^C NMR (DMSO-*d*_6_) 170.50, 141.78, 117.35, 114.74, 110.49, 61.62, 53.53, 47.50, 38.10, 30.13.

2-(4-(Aminomethyl)piperidin-1-yl)-N-(5-cyano-1H-benzimidazol-2-yl)acetamide **22**. Yield 10%; m.p. = 189–191 °C. Purified via silica gel column chromatography, eluent DCM 9.4/MeOH 0.6/NH_4_OH 0.06. Purified on silica gel column chromatography, eluent DCM 8.6/MeOH 1.4/NH_4_OH 0.14. ^1^H NMR (DMSO-*d*_6_) 7.87 (s, 2H, ar), 7.58 (d, 2H, ar, J = 8.2 Hz), 7.48 (d, 2H, ar, J = 8.2 Hz), 3.28 (s, 2H, CH_2_), 2.91–2.89 (m, 2H, CH_2_), 2.20–2.15 (m, 2H, CH_2_), 1.69–1.67 (m, 2H, CH_2_), 1.22–1.19 (m, 3H, CH and CH_2_). ^13^C NMR (DMSO-*d_6_*) 170.61, 149.69, 125.20, 120.81, 119.02, 115.65, 102.95, 61.54, 53.53, 47.61, 38.24, 30.13.

N-(5-Chloro-1-benzimidazol-2-yl)-2-morfolinoacetamide **23**. Yield 22%; m.p. = 194–196 °C. Purified via silica gel column chromatography, eluent DCM 9.5/MeOH 0.5. ^1^H NMR (DMSO-*d*_6_) 12.20 (br s, 1H, NH), 11.30 (br s, 1H, NH), 7.47–7.45 (m, 2H, ar), 7.11 (d, 1H, ar, J = 8.5 Hz), 3.63 (t, 2H, CH_2_, J = 4.7 Hz), 3.30 (s, 2H, CH_2_), 2.57 (t, 2H, CH_2_, J = 4.7 Hz).

N-(5,6-Dichloro-1-benzimidazol-2-yl)-2-morfolinoacetamide **24**. Yield 15%; m.p. = 227–229 °C. Purified via silica gel column chromatography, eluent DCM 9.5/MeOH 0.5. ^1^H NMR (DMSO-*d_6_*) 12.33 (br s, 1H, NH), 11.41 (br s, 1H, NH), 7.66 (s, 2H, ar), 3.63 (t, 2H, CH_2_, J = 4.5 Hz), 3.33 (s, 2H, CH_2_), 2.57 (t, 2H, CH_2_, J = 4.5 Hz). ^13^C NMR (DMSO-*d_6_*) 170.18, 148.58, 66.63, 61.16, 53.36.

2-Morpholino-N-(5-nitro-1H-benzimidazol-2-yl)acetamide **25**. Yield 54%; m.p. = 250–252 °C. Purified via silica gel column chromatography, eluent DCM 9.6/MeOH 0.4. ^1^H NMR (DMSO-*d_6_*) 12.70 (br s, 1H, NH), 11.62 (br s, 1H, NH), 8.35 (s, 1H, ar), 8.06 (d, 1H, ar, J = 8.8 Hz), 8.35 (s, 1H, ar), 7.62–7.60 (m, 1H, ar), 3.63 (t, 2H, CH_2_, J = 4.6 Hz), 3.35 (s, 2H, CH_2_), 2.58 (t, 2H, CH_2_, J = 4.6 Hz).

N-(5-Cyano-1H-benzimidazol-2-yl)-2-morpholinoacetamide **26**. Yield 66%; m.p. = 247–249 °C. Purified via silica gel column chromatography, eluent DCM 9.6/MeOH 0.4/NH_4_OH 0.1. ^1^H NMR (DMSO-*d_6_*) 12.54 (br s, 1H, NH), 11.49 (br s, 1H, NH), 7.88 (s, 1H, ar), 7.61–7.59 (m, 1H, ar), 7.50 (d, 1H, ar, J = 8.2 Hz), 3.63 (t, 2H, CH_2_, J = 4.5 Hz), 3.33 (s, 2H, CH_2_), 2.57 (t, 2H, CH_2_, J = 4.5 Hz).

N-(5-Chloro-1H-benzo[d]imidazol-2-yl)-2-((2-morpholinoethyl)amino)acetamide **27**. Yield 32%; m.p. = 172–174 °C. Purified via silica gel column chromatography, eluent DCM 9/MeOH 1/NH4OH 0.1. ^1^H NMR (DMSO-*d_6_*) 12.23 (br s, 1H, NH), 7.47–7.43 (m, 2H, ar), 7.10 (dd, ar, J = 8.5, J = 1.8 Hz), 6.94 (br s, 1H, NH), 3.61–3.59 (m, 4H, 2CH_2_), 3.45 (s, 2H, CH_2_), 2.70–2.67 (m, 2H, CH_2_), 2.41–2.38 (m, 4H, 2 CH_2_).

N-(6-Cyano-3H-indol-2-yl)-2-((2-morpholinoethyl)amino)acetamide **28**. Yield 21%; m.p. = 175–177 °C. Purified via silica gel column chromatography, eluent DCM 9/MeOH 1/NH4OH 0.1. ^1^H NMR (DMSO-*d_6_*) 7.88 (s, 1H, ar), 7.60 (m, 1H, ar), 7.49 (d, 1H, ar, J = 8.2 Hz), 6.94 (br s, 1H, NH), 3.60–3.59 (m, 4H, 2CH_2_), 3.49 (s, 2H, CH_2_), 2.71–2.69 (m, 2H, CH_2_), 2.42–2.38 (m, 4H, 2 CH_2_).

N-(5-Chloro-1H-benzimidazol-2-yl)-2-(pyrrolidin-1-yl)acetamide **29**. Yield 24%; m.p. = 242–244 °C. ^1^H NMR (DMSO-*d_6_*) 12.20 (br s, 1H, NH), 11.28 (br s, 1H, NH), 7.47–7.43 (m, 2H, ar), 7.11 (d, 2H, ar, J = 8.5 Hz), 3.41 (s, 2H, CH_2_), 2.65–2.62 (m, 4H, 2 CH_2_), 1.76–1.73 (m, 4H, 2 CH_2_).

N-(5,6-Dichloro-1H-benzimidazol-2-yl)-2-(pyrrolidin-1-yl)acetamide **30**. Yield 44%; m.p. = 249–251 °C. Purified via silica gel column chromatography, eluent DCM 9.5/MeOH 0.5/NH_4_OH 0.05 ^1^H NMR (DMSO-*d_6_*) 12.30 (br s, 1H, NH), 11.41 (br s, 1H, NH), 7.66 (s, 2H, ar), 3.42 (s, 2H, CH_2_), 2.68–2.62 (m, 4H, 2 CH_2_), 1.79–1.76 (m, 4H, 2 CH_2_). ^13^C NMR (DMSO-*d*_6_): 170.76, 148.68, 58.41, 53.87, 23.97.

N-(5-Nitro-1H-benzimidazol-2-yl)-2-(pyrrolidin-1-yl)acetamide **31**. Yield 17%; m.p. = 247–249 °C. Purified via silica gel column chromatography, eluent DCM 9.4/MeOH 0.6/NH_4_OH 0.06. ^1^H NMR (DMSO-*d_6_*) 12.46 (br s, 1H, NH), 11.80 (br s, 1H, NH), 8.33 (s, 2H, ar), 8.06 (d, 2H, ar, J = 8.8 Hz), 7.60 (d, 2H, ar, J = 8.8 Hz), 3.45 (s, 2H, CH_2_), 2.66–2.63 (m, 4H, 2 CH_2_), 1.76–1.73 (m, 4H, 2 CH_2_).

N-(5-Cyano-1H-benzimidazol-2-yl)-2-(pyrrolidin-1-yl)acetamide **32**. Yield 41%; m.p. = 200–202 °C. Purified via silica gel column chromatography, eluent DCM 9.4/MeOH 0.6/NH_4_OH 0.06. ^1^H NMR (DMSO-*d_6_*) 12.44 (br s, 1H, NH), 11.54 (br s, 1H, NH), 7.88 (s, 2H, ar), 7.60 (d, 2H, ar, J = 8.2 Hz), 7.50 (d, 2H, ar, J = 8.2 Hz), 3.44 (s, 2H, CH_2_), 2.66–2.63 (m, 4H, 2 CH_2_), 1.76–1.73 (m, 4H, 2 CH_2_). ^13^C NMR (DMSO-*d*_6_) 170.86, 149.17, 125.44, 120.68, 58.44, 53.87, 23.98.

#### 3.1.2. General Procedure for the Synthesis of 2-Chloro-N-(5-Chloro-1H-benzimidazol-2-yl)acetamide Derivatives **38**–**42**

The appropriate 2-aminobenzimidazole **33**–**37** [37] (1 mmol) and triethylamine (1 mmol) were dissolved in anhydrous THF and stirred at room temperature under a nitrogen atmosphere for 45 min. The mixture was subsequently cooled to 0 °C, and a solution of chloroacetyl chloride (2 mmol) in anhydrous THF (1–2 mL) was slowly added dropwise. The reaction mixture was stirred at 0 °C for 2 h and then allowed to warm to room temperature and stirred overnight. After filtration to remove the precipitated triethylamine hydrochloride, the solvent was removed under reduced pressure. The resulting residue was washed with H_2_O (15 mL) to eliminate any unreacted chloroacetyl chloride, then was collected by filtration and recrystallized from EtOH.

2-Chloro-N-(5-chloro-1H-benzimidazol-2-yl)acetamide **38**. Yield 91%; m.p. = 170–172 °C. ^1^H NMR (DMSO-*d_6_*) 12.07 (br s, 1H, NH), 7.45 (dd, 2 H, ar, J = 3.2 Hz, J = 5.8 Hz), 7.12 (dd, 2 H, ar, J = 3.2 Hz, J = 5.8 Hz), 4.38 (s, 2 H, CH_2_).

2-Chloro-N-(5-chloro-1H-benzimidazol-2-yl)acetamide **39**. Yield 80%; m.p. = 188–189 °C. ^1^H NMR (DMSO-*d_6_*) 12.02 (br s, 1H, NH), 7.49 (d, 1H, ar, J = 2.1 Hz), 7.46 (d, 1H, ar, J = 8.51 Hz), 7.14 (dd, 1H, ar, J = 2.1 Hz, J = 8.51 Hz), 4.40 (s, 2 H, CH_2_).

2-Chloro-N-(5,6-dichloro-1H-benzimidazol-2-yl)acetamide **40**. Yield 55%; m.p. = 252–253 °C. ^1^H NMR (DMSO-*d_6_*) 12.60 (br s, 1H, NH), 12.07 (br s, 1 H, NH), 7.68 (s, 2 H, ar), 4.42 (s, 2 H, CH_2_).

2-Chloro-N-(5-nitro-1H-benzimidazol-2-yl)acetamide **41**. Yield 50%; m.p. = 216–218 °C. Purified by recrystallization from EtOH. TLC eluent DCM 8/MeOH 2. ^1^H NMR (DMSO-*d_6_*) 12.80 (br s, 1H, NH), 12.29 (br s, 1H, NH), 8.37 (s, 1H, ar), 8.11 (dd, 1H, ar, J = 2.1 Hz, J = 8.8 Hz), 7.63 (d, 1H, ar, J = 8.8 Hz); 4.49 (s, 2 H, CH_2_).

2-Chloro-N-(5-cyano-1H-benzimidazol-2-yl)acetamide **42**. Yield 65%; m.p. = 207–209 °C. Purified by recrystallization from EtOH. ^1^H NMR (DMSO-*d_6_*) 12.52 (br s, 1H, NH), 12.19 (br s, 1H, NH), 7.90 (s, 1H, ar); 7.62 (d, 1H, ar, 8.2 Hz); 7.52 (d, 1H, ar, J = 8.2 Hz), 4.44 (s, 2 H, CH_2_).

### 3.2. CK1δ Activity Assays

The inhibitory effects of the synthesized molecules on CK1δ were evaluated using a luminescence-based kinase assay (KinaseGlo^®^ kit, Promega Italy Srl, Milan, Italy), following previously described general procedures [37,48]. The experiments were carried out in white 96-well plates, with each reaction brought to a final volume of 40 μL. The assay buffer consisted of Milli-Q water supplemented with 50 mM HEPES (pH 7.5), 1 mM EDTA, 1 mM EGTA, and 15 mM MgCl_2_. For each measurement, 10 μL of the test compound (prepared by diluting a 10 mM stock in DMSO to the desired concentration using assay buffer) was added to the wells, followed by 10 μL of CK1δ enzyme solution (recombinant human CK1δ, truncated form, GST-tagged, aa 1-294; Merck Millipore, Milan, Italy). The reaction was initiated by adding 20 μL of assay buffer containing casein as the substrate (5% aqueous solution from bovine milk, Sigma-Aldrich, Milan, Italy) and ATP, resulting in final concentrations of 0.1% casein and 2 μM ATP. The enzyme concentration in the final mixture was 6.5 nM. As reference controls, the CK1δ inhibitor **PF-670462** (IC_50_ = 14 nM) [49] was included at 40 μM as the positive control, while a buffer/DMSO mixture served as the negative control. The total DMSO percentage never exceeded 1%.

After assembling the reaction mixtures, plates were incubated for 60 min at 30 °C. The reactions were then quenched by adding 40 μL of KinaseGlo reagent. After a 10 min equilibration at 25 °C, luminescence (expressed as relative light units, RLU) was recorded using a Tecan Infinite M1000 reader (Tecan Group Ltd, Männerdorf, Switzerland). For each compound, the residual kinase activity at 40 μM was first assessed in duplicate relative to the DMSO control. Only compounds reducing CK1δ activity to below 50% at this screening concentration were selected for full dose–response analysis. IC_50_ determination was carried out using a ten-point dilution series ranging from 100 μM to 0.010 μM. Data processing and curve fitting were performed using Excel and GraphPad Prism (v. 8.0.2).

#### ATP-Competition Assay

ATP-competition experiments were performed using the ADP-Glo™ Kinase Assay (Promega), by varying the ATP concentration in the presence of two concentrations of the test compound, corresponding to its IC_50_ and 2× IC_50_ values. The experiments were carried out in white 96-well plates, and all assay components were diluted using the same buffer described in Section 3.2. For each reaction, 6.25 μL of the test compound, at either 1× IC_50_ or 2× IC_50_, was added to the wells, followed by 6.25 μL of ATP, tested at six different concentrations ranging from 1 to 50 μM and prepared from an 8 mM stock solution. Subsequently, 6.25 μL of casein solution and 6.25 μL of CK1δ enzyme solution were added, resulting in a final casein concentration of 0.1% and an enzyme concentration of 6.5 nM, respectively. The plates were incubated for 60 min at 30 °C. Subsequently, 25 μL of ADP-Glo™ reagent was added to each well, and the plates were incubated for 40 min at 25 °C. Then, 50 μL of ADP-Glo™ detection reagent was added to each well, followed by a further 30 min incubation at 25 °C. Luminescence was subsequently measured using a Tecan Infinite M1000 plate reader (Tecan Group Ltd, Männerdorf, Switzerland). An ADP calibration curve was generated on the same plate using six ADP concentrations ranging from 1 to 50 μM. The amount of ADP produced during the kinase reaction was determined by interpolating the luminescence signal, expressed as relative luminescence units (RLU), against the ADP standard calibration curve. In parallel, an experiment was performed at the different ATP concentrations described above, in the presence of casein and CK1δ, without inhibitors, using the same experimental procedure, to assess the ATP-competitive behaviour of the compounds. All experiments were performed in duplicate. Reaction velocities were calculated from the amount of ADP produced, and the reciprocal values of reaction velocity (1/V) were plotted against the reciprocal ATP concentrations (1/[ATP]) using GraphPad Prism 8.0 to generate Lineweaver–Burk plots.

### 3.3. Molecular Modelling Studies

#### 3.3.1. Hardware and Software Overview

All molecular modelling operations were conducted using the MOE software suite [50]. For protein–ligand docking, the PLANTS (Protein–Ligand ANT System) algorithm [51] was employed; the resulting poses were evaluated through interaction energy calculations, pharmacophoric filtering, and visual inspection, all performed within the integrated MOE environment.

Molecular dynamics (MD) systems were built using AmberTools22 [52], MD simulations were run using ACEMD engine [53], and the collected simulations were analysed with VMD-1.9.3 [54].

Structure preparation, simulation setup, and trajectory processing were carried out on a Linux workstation equipped with a 32-core Intel Core i9-14900K processor (6.0 GHz). MD simulations were executed on a GPU cluster consisting of 30 NVIDIA graphics processing units, spanning from GTX 980 to RTX 5090 models.

#### 3.3.2. Protein Structure Preparation

The three-dimensional structure of the selected complex for analysis was retrieved from the Protein Data Bank [55] (PDB ID: 5OKT) and subsequently prepared within the MOE 2024.06 suite [50]. Structure preparation was carried out using the “Structure Preparation” tool provided within the MOE environment (capping the N- and C-termini, assigning tautomeric forms, and side-chain conformations for ambiguous residues). Partial charges were assigned using the AMBER14EHT force field, which was also used to minimize the energy of hydrogen atoms added during the preparation workflow. Protonation states were identified at pH 7.4 using the Protonate3D tool, which identifies the most probable ionization state for each residue by assessing its hydrogen bonding contribution to overall structural stability. In conditions where numerous side-chain conformations existed, the rotamer with the highest occupancy was chosen. The crystallographic complex used as a reference term for comparison in the molecular dynamics study (PDB ID: 4TWC [35]) was retrieved and prepared following the same procedure.

#### 3.3.3. Molecular Docking Simulation

Ligand preparation was performed through a pipeline of tools from the OpenEye (QUACPAC 2.3.0.0) suite [56], three-dimensional coordinates were generated with Omega, the most probable tautomeric states were predicted with tautomers, protonation states were assigned using Fixpka, and partial charges were assigned through Molcharge.

Protein–ligand docking was carried out using the PLANTS (Protein–Ligand ANT System) algorithm [51], using the CHEM-PLP scoring function. A sphere of radius 10 Å centred on the centre of mass of the crystallographic ligand was used for the docking search. Twenty poses were generated for each ligand.

#### 3.3.4. Pharmacophoric Filter

The MOE suite was used to generate a structure-based pharmacophore model for the crystallographic complex 5OKT. This resulted in properties identifying the ligand’s aromatic scaffold and a bidentate hydrogen bond with Leu85. Donor and acceptor features were positioned on the ligand’s 2-aminobenzothiazole NH and N, whereas acceptor and donor features were positioned on the Leu85 carbonyl and amino group. The feature sphere’s dimension was always 2 Å.

#### 3.3.5. Docking Video Maker

MMsDocking Video Maker, an in-house tool, was used to automatically generate video representations of docking results, integrating docking poses, per-residue hydrophobic IEhyd and electrostatic IEele interaction energy data, and experimental binding data into a unified visualization to facilitate comparative analysis of docking outcomes. Individual frames were assembled into video format using MEncoder 1.4 [57]. Background heatmaps of per-residue IEhyd and IEele values, computed within MOE using Amber14EHT and MMFF94 partial charges for protein and ligands, respectively, were rendered using GNUPLOT 5.2.8 [58]. Two-dimensional ligand representations were generated using the RDKit cheminformatics toolkit 2025.3.4-1.0.0 [59]. Docking pose visualization within the binding site was performed using UCSF Chimera 1.16 [60].

#### 3.3.6. System Setup for MD Simulations and Equilibration Protocol

Ligand–protein complexes for molecular dynamics (MD) simulations were prepared using Visual Molecular Dynamics (VMD) 1.9.3 [54] and AmberTools22 [61]. The protein was parameterized with the ff14SB force field [62], while ligands were described using the General Amber Force Field (GAFF) [63]; partial charges were assigned using the AM1-BCC method. The TIP3P water model was used to solve systems in an orthorhombic box with a 20 Å buffer between complex and box boundaries. The system was neutralized by adding sodium (Na^+^) and chloride (Cl^−^) ions to achieve a physiological ionic strength of 0.154 M. To minimize steric clashes and unfavourable interactions, a 500-step energy minimization was carried out using the conjugate gradient approach.

Prior to the MD production phase, a two-step equilibration approach was performed. During the initial equilibration, all protein and ligand atoms were constrained with a harmonic positional constraint of 5 kcal mol^−1^ Å^−2^ over a 1 ns simulation performed in the canonical (NVT) ensemble. In the second equilibration step, the same restraint was exclusively applied to ligand and protein backbones during an equal-length simulation in the isothermal–isobaric ensemble (NPT). Pressure was maintained at 1 atm using a Monte Carlo barostat [64], while temperature was maintained at 310 K throughout all equilibration phases via a Langevin thermostat [65].

The ACEMD 3.7.6 [53] engine, which is based on the open-source Python package OpenMM 8.2.0 [66], was used for all MD simulations. An integration time step of 2 fs was adopted, and bonds involving hydrogen atoms were constrained using the M-SHAKE method [67]. Long-range electrostatic interactions were computed using the particle-mesh Ewald approach [68] with a switching distance of 7.5 Å for Lennard-Jones interactions and a cutoff of 9.0 Å for Lennard-Jones and real-space electrostatic interactions. Production runs of 100 ns were carried out in triplicate at 310 K for both the compound 20/CK1δ complex and the reference **Bischof-5**/CK1δ complex, saving one frame every 20 ps, for a total of 5000 frames per replica.

#### 3.3.7. MD Trajectory Analysis

Ligand RMSD was computed for the solvent-exposed atoms of compound **20**, corresponding to the piperidin-4-ylmethanamine substituent (atoms C8, C9, C10, C11, C12, C13, C14, N2, and N3). For each trajectory frame, the system was first superimposed onto the initial frame via roto-translational alignment of the protein backbone atoms, and the RMSD of the selected ligand atoms was subsequently calculated without further fitting.

The trajectory-averaged RMSD of each replica was then computed. Mean RMSD profiles and the corresponding inter-replica standard deviations were also computed as a function of simulation time and are reported, for both complexes, in Figure S2 (Supplementary Materials). The same procedure was applied to the reference compound Bischof-5, whose solvent-exposed selection comprised atoms C1, C2, C3, C5, C6, C7, C8, C9, C10, C11, C12, S, N1, N2, O2, O3, F1, F2 and F3. RMSD values were computed with MDAnalysis 2.5.0 [69] and reported as mean ± SD over the three replicas in Table 2.

Ligand–protein contact persistence was calculated over the 100 ns trajectory for each replica using a distance cutoff of 3.5 Å. A contact between the ligand and a given protein residue was considered established when the minimum distance between any heavy atom of the ligand and any heavy atom of the residue fell within the defined threshold. For each residue, the contact persistence was expressed as the percentage of simulation frames in which the contact was detected within each individual replica, and the mean value and inter-replica standard deviation were computed across the three independent simulations. Only residues displaying a mean contact persistence ≥ 20% across the replicas were retained for analysis and visualization.

Root-mean-square fluctuations (RMSF) of the ligand atoms were computed with VMD 1.9.3 [54]. Each frame was superimposed on the first frame of the corresponding replica using the same protein backbone selection and the same roto-translational fit adopted for the RMSD calculation, and the resulting transformation was applied to all the atoms of the frame. The RMSF of each ligand atom was then obtained as the root-mean-square deviation of its position from the average position of the same atom over the whole set of superimposed frames, the average structure being derived directly from the trajectory. Per-atom values were averaged over the three replicas and are reported, atom by atom, in Figure 6D, for compound **20** and in Figure S1D, for **Bischof-5**, with error bars corresponding to the inter-replica SD. In addition, per-atom values were averaged over the solvent-exposed selection defined above to give a single value per replica, reported as mean ± SD over the three replicas in Table 2.

Hydrogen bonds between the ligand and the protein were identified with the HydrogenBondAnalysis module of MDAnalysis 2.5.0 [69], restricting the search to donor–acceptor pairs in which one partner belongs to the solvent-exposed atom selection of the ligand and the other to the protein, in either direction. Donor, hydrogen and acceptor atoms were assigned automatically on the basis of element type and connectivity. A hydrogen bond was counted when the donor–acceptor distance was ≤3.5 Å and the donor–H···acceptor angle ≥ 150°. Since the analysis was performed on the water- and ion-stripped trajectories, the resulting counts refer exclusively to direct ligand–protein hydrogen bonds and do not include water-mediated interactions. For each replica, the number of hydrogen bonds detected in each frame was averaged over the trajectory, and the mean ± SD over the three replicas is reported in Table 2.

Two-dimensional ligand interaction diagrams (Figure 6C and Figure S1C) were generated with the Ligand Interactions tool of MOE [50] from the representative pose extracted from the MD trajectories, and for each complex, the hydrogen bonds and the hydrophobic contacts established with the residues lining the binding site have been summarized.

### 3.4. Cell Viability Assay

Cell viability was determined using the MTT colorimetric assay according to the Biotium protocol, with minor modifications. SH-SY5Y cells (purchased from Merck, 1VL 94030304) were cultured at 37 °C under an atmosphere containing 5% CO_2_. For the assay, cells were seeded into 96-well tissue culture plates at a concentration of 1.0 × 10^5^ cells per well in 100 µL of complete culture medium containing up to 10% foetal bovine serum (FBS). Under these conditions, approximately 6.0 × 10^4^ cells were sufficient to reach confluence after about 48 h.

After 24 h, to allow cell attachment, cells were treated with compounds **IV**, **20**, **22** and **24** at the indicated concentrations and incubated for an additional 24 h. Untreated cells cultured in complete medium were used as the positive control and defined as 100% viability (**CNT−**), whereas cells treated with 2% Triton X-100 were used as the negative control and defined as 0% viability (**CNT+**).

At the end of the treatment period, 10 µL of MTT solution was added to each well, and the plates were incubated at 37 °C for 4 h; for high cell densities (>1.0 × 10^5^ cells per well), the incubation time was reduced to 2 h. Formazan crystals were then solubilized by the addition of 200 µL of DMSO, followed by thorough mixing.

Absorbance was recorded at 570 nm using a microplate reader, with background absorbance measured at 630 nm. Normalized absorbance values were calculated by subtracting the background signal, and cell viability was expressed as a percentage relative to untreated control cells.

## 4. Conclusions

In conclusion, this study identified a novel series of benzimidazol-2-acetamide derivatives (**1**–**32**) as ATP-competitive CK1δ inhibitors. These derivatives were designed by replacing the pyrazol-3-yl moiety in the 2-acetamido side chain of the lead compounds with basic saturated N-heterocycles to improve their physicochemical profile. Importantly, although the novel compounds were in general slightly less potent than the leads, they retained inhibitory activity in the micromolar range while displaying improved predicted drug-like properties.

Among the substitution patterns on the fused benzene ring, the 5,6-dichloro substitution proved to be the most advantageous, followed by the 5-cyano group. In particular, the most active compounds in the series were the 5,6-dichloro derivatives **20** and **24**, bearing piperidin-4-ylmethanamine and morpholine moieties at the R_1_ position, respectively (IC_50_ = 0.172 and 0.223 μM, respectively), together with the 5-cyano derivative **22** (IC_50_ = 0.224 μM). Compounds **20**, **22**, and **24** showed good tolerability in SH-SY5Y neuroblastoma cells, with no significant cytotoxicity at concentrations up to 10 μM. In silico ADME predictions indicated favourable oral absorption for most derivatives and suggested potential blood–brain barrier penetration for compounds **6**, **7**, **24**, **29**, and **30**, supporting the suitability of this scaffold for the development of CNS-directed CK1δ inhibitors.

Computational studies supported the experimental findings by confirming that the new derivatives preserve the canonical CK1δ binding mode through stable interactions of the benzimidazole core with the hinge region. In contrast, molecular dynamics simulations indicated that the newly introduced solvent-exposed substituents remain conformationally flexible and establish only transient interactions with residues at the outer edge of the binding site. This dynamic behaviour provides a plausible explanation for why derivatives of the new series generally did not surpass the potency of the best compounds previously reported. These insights offer valuable guidance for the future structure-based optimization of CK1δ inhibitors, indicating that further improvements may require the identification of alternative binding vectors capable of engaging more stable interaction networks beyond the conserved hinge region.

## Figures and Tables

**Table 1:** Inhibitory activity of the novel benzimidazole derivatives **1**–**32** and lead compounds **I**–**IV** against CK1δ ^a^.

	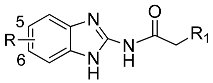									
**R_1_**	**R**									
	H		5-Cl		5,6-diCl		5-NO_2_		5-CN	
		IC_50_ (μM)		IC_50_ (μM)		IC_50_ (μM)		IC_50_ (μM)		IC_50_ (μM)
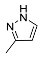	-		**I**	0.485 ± 0.310	**II**	0.98 ± 0.35	**III**	0.12 ± 0.083	**IV**	0.0986 ± 0.0394
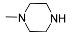	**1**	>40	**2**	1.11 ± 0.41	**3**	>40	**4**	0.647 ± 0.236	**5**	>40
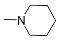	-		**6**	1.54 ± 0.34	**7**	1.66 ± 0.45	**8**	1.02 ± 0.197	**9**	0.632 ± 0.263
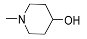	**10**	>40	**11**	1.92 ± 0.54	**12**	0.616 ± 0.177	**13**	0.886 ± 0.262	**14**	1.19 ± 0.37
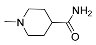	-		**15**	2.7 ± 0.41	**16**	0.356 ± 0.053	**17**	1.27 ± 0.31	**18**	0.343 ± 0.060
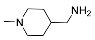	-		**19**	1.61 ± 0.28	**20**	0.172 ± 0.026	**21**	>40	**22**	0.224 ± 0.069
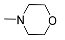	-		**23**	1.48 ± 0.21	**24**	0.223 ± 0.075	**25**	4.37 ± 1.45	**26**	1.54 ± 0.55
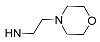	-		**27**	5.44 ± 1.00	-		-		**28**	2.14 ± 0.44
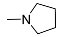	-		**29**	7.96 ± 2.15	**30**	0.951 ± 0.138	**31**	3.31 ± 0.61	**32**	1.80 ± 0.52

^a^ IC_50_ values represent the mean ± SE of three independent experiments performed in duplicate.

**Table 2:** Stability descriptors of the solvent-exposed region of compound **20** and of the reference inhibitor **Bischof-5**, computed over three independent 100 ns MD replicas of the corresponding CK1δ complexes. Values are reported as mean ± SD across replicas.

Complex	RMSD (Å) ^a^	RMSF (Å) ^b^	H-Bonds per Frame ^c^
Bischof-5/CK1δ (4TWC)	1.54 ± 0.28	1.21 ± 0.13	0.46 ± 0.09
Compound **20**/CK1δ (5OKT)	2.14 ± 0.69	1.67 ± 0.20	0.38 ± 0.02

^a^ Mean RMSD of the solvent-exposed ligand atoms, averaged over the trajectory of each replica after roto-translational superposition of the protein backbone. ^b^ Mean RMSF of the same atom selection, computed with respect to the average structure of each replica. ^c^ Average number of direct hydrogen bonds per frame between the solvent-exposed atom selection and the protein.

## Data Availability

The original contributions presented in this study are included in the article/Supplementary Material. Further inquiries can be directed to the corresponding author.

## Supplementary Materials

The following supporting information can be downloaded at: https://www.mdpi.com/article/10.3390/ph19101666/s1. Video_S1: Selected docking poses and relative colour-coded per-residue interaction energy fingerprint (electrostatic and hydrophobic) for all the compounds. Figure S1: Molecular dynamics analysis of the reference inhibitor **Bischof-5** in complex with CK1δ (PDB: 4TWC), including binding pose, ligand–protein contact persistence, two-dimensional interaction diagram and per-atom RMSF. Figure S2: Time profiles of the ligand RMSD computed for the solvent-exposed atom selection of compound **20** over three independent 100 ns MD replicas. Figure S3: Time profiles of the ligand RMSD computed for the solvent-exposed atom selection of the reference inhibitor Bischof-5 over three independent 100 ns MD replicas; predicted ADME properties using SwissADME; ^1^H-NMR and ^13^C-APT NMR scanned spectra of compounds **1**–**32**. Table S1: Combustion analysis data of the newly synthesized compounds.

## References

[1] Landrock M.F., Krutyhołowa R., Böhnert P., Mazur J., Honc M., Hammermeister A., Bessler L., Scherf D., Elms A., Radczuk N. (2025). Autophosphorylation of conserved yeast and human casein kinase 1 isozymes regulates Elongator-dependent tRNA modifications. Nucleic Acids Res..

[2] Francisco J.C., Virshup D.M. (2022). Casein Kinase 1 and Human Disease: Insights from the Circadian Phosphoswitch. Front. Mol. Biosci..

[3] Knippschild U., Marc Krüger M., Richter J., Xu P., García-Reyes B., Peifer C., Halekotte J., Bakulev V., Bischof J. (2014). The CK1 Family: Contribution to Cellular Stress Response and Its Role in Carcinogenesis. Front. Oncol..

[4] Xu P., Fischer-Posovszky P., Bischof J., Radermacher P., Wabitsch M., Henne-Bruns D., Anna- Wolf M., Hillenbrand A., Knippschild U. (2015). Gene expression levels of Casein kinase 1 (CK1) isoforms are correlated to adiponectin levels in adipose tissue of morbid obese patients and site-specific phosphorylation mediated by CK1 influences multimerization of adiponectin. Mol. Cell. Endocrinol..

[5] Narasimamurthy R., Virshup D.M. (2021). The phosphorylation switch that regulates ticking of the circadian clock. Mol. Cell.

[6] Cruciat C.M. (2014). Casein kinase 1 and Wnt/β-catenin signalling. Curr. Opin. Cell Biol..

[7] Long N.H., Lee S.-J. (2023). Targeting casein kinase 1 for cancer therapy: Current strategies and future perspectives. Front. Oncol..

[8] Zhu M., Zhang J., Bian S., Zhang X., Shen Y., Ni Z., Xu S., Cheng C., Zheng W. (2022). Circadian gene CSNK1D promoted the progression of hepatocellular carcinoma by activating Wnt/β-catenin pathway via stabilizing Dishevelled Segment Polarity Protein 3. Biol. Proced. Online.

[9] Ning J., Sun Q., Su Z., Tan L., Tang Y., Sayed S., Li H., Xue V.W., Liu S., Chen X. (2022). The CK1δ/ϵ-Tip60 Axis Enhances Wnt/β-Catenin Signaling via Regulating β-Catenin Acetylation in Colon Cancer. Front. Oncol..

[10] Wen Y., Wang H., Yang X., Zhu Y., Li M., Ma X., Huang L., Wan R., Zhang C., Li S. (2024). Pharmacological targeting of casein kinase 1δ suppresses oncogenic NRAS-driven melanoma. Nat. Commun..

[11] Wang Z., Zhou L., Wang Y., Peng Q., Li H., Zhang X., Su Z., Song J., Sun Q., Sayed S. (2021). The CK1δ/ε-AES axis regulates tumorigenesis and metastasis in colorectal cancer. Theranostics.

[12] Burger K.L., Fernandez M.R., Meads M.B., Sudalagunta P., Oliveira P.S., Canevarolo R.R., Alugubelli R.R., Tungsevik A., De Avila G., Silva M. (2023). CK1δ and CK1ε Signaling Sustains Mitochondrial Metabolism and Cell Survival in Multiple Myeloma. Cancer. Res..

[13] Rosenberg L.H., Cleveland J.L., Roush W.R., Duckett D.R. (2016). CK1δ: An exploitable vulnerability in breast cancer. Ann. Transl. Med..

[14] Wu X., Yang Z., Zou J., Gao H., Shao Z., Li C., Lei P. (2025). Protein kinases in neurodegenerative diseases: Current understandings and implications for drug discovery. Signal Transduct. Target Ther..

[15] Catarzi D., Varano F., Vigiani E., Lambertucci C., Spinaci A., Volpini R., Colotta V. (2022). Casein Kinase 1δ Inhibitors as Promising Therapeutic Agents for Neurodegenerative Disorders. Curr. Med. Chem..

[16] Brennan K.C., Bates E.A., Shapiro R.E., Zyuzin J., Hallows W.C., Huang Y., Lee H.Y., Jones C.R., Fu Y.H., Charles A.C. (2013). Casein kinase δ mutations in familial migraine and advanced sleep phase. Sci. Transl. Med..

[17] Mainieri G., Montini A., Nicotera A., Di Rosa G., Provini F., Loddo G. (2021). The Genetics of Sleep Disorders in Children: A Narrative Review. Brain Sci..

[18] Philpott J.M., Freeberg A.M., Park J., Lee K., Ricci C.G., Hunt S.R., Narasimamurthy R., Segal D.H., Robles R., Cai Y. (2023). PERIOD phosphorylation leads to feedback inhibition of CK1 activity to control circadian period. Mol. Cell.

[19] Adler P., Mayne J., Walker K., Ning Z., Figeys D. (2019). Therapeutic Targeting of Casein Kinase 1δ/ε in an Alzheimer’s Disease Mouse Model. J. Proteome Res..

[20] Hondius D.C., Koopmans F., Leistner C., Pita-Illobre D., Peferoen-Baert R.M., Marbus F., Paliukhovich I., Li K.W., Rozemuller A.J.M., Hoozemans J.J.M. (2021). The proteome of granulovacuolar degeneration and neurofibrillary tangles in Alzheimer’s disease. Acta Neuropathol..

[21] Roth A., Sander A., Oswald M.S., Gärtner F., Knippschild U., Bischof J. (2022). Comprehensive Characterization of CK1δ-Mediated Tau Phosphorylation in Alzheimer’s Disease. Front. Mol. Biosci..

[22] Ko V.I., Ong K., Cleveland D.W., Yu H., Ravits J.M. (2024). CK1δ/ε kinases regulate TDP-43 phosphorylation and are therapeutic targets for ALS-related TDP-43 hyperphosphorylation. Neurobiol. Dis..

[23] Ko V.I., Ong K., Kwon D.Y., Li X., Pietrasiewicz A., Harvey J.S., Lulla M., Bhat G., Cleveland D.W., Ravits J.M. (2024). CK1δ/ε-mediated TDP-43 phosphorylation contributes to early motor neuron disease toxicity in amyotrophic lateral sclerosis. Acta Neuropathol. Commun..

[24] Bissaro M., Federico S., Salmaso V., Sturlese M., Spalluto G., Moro S. (2018). Targeting Protein Kinase CK1δ with Riluzole: Could It Be One of the Possible Missing Bricks to Interpret Its Effect in the Treatment of ALS from a Molecular Point of View?. ChemMedChem.

[25] Alquezar C., Salado I.G., de la Encarnación A., Pérez D.I., Moreno F., Gil C., López de Munain A., Martínez A., Martín-Requero A. (2016). Targeting TDP-43 phosphorylation by Casein Kinase-1δ inhibitors: A novel strategy for the treatment of frontotemporal dementia. Mol. Neurodegener..

[26] Nonaka T., Suzuki G., Tanaka Y., Kametani F., Hirai S., Okado H., Miyashita T., Saitoe M., Akiyama H., Masai H. (2016). Phosphorylation of TAR DNA-binding Protein of 43 kDa (TDP-43) by Truncated Casein Kinase 1δ Triggers Mislocalization and Accumulation of TDP-43. J. Biol. Chem..

[27] Waxman E.A., Giasson B.I. (2008). Specificity and Regulation of Casein Kinase/Mediated Phosphorylation of α-Synuclein. J. Neuropathol. Exp. Neurol..

[28] Torii S., Arakawa S., Sato S., Ishikawa K.I., Taniguchi D., Sakurai H.T., Honda S., Hiraoka Y., Ono M., Akamatsu W. (2023). Involvement of casein kinase 1 epsilon/delta (Csnk1e/d) in the pathogenesis of familial Parkinson’s disease caused by CHCHD2. EMBO Mol. Med..

[29] Wager T.T., Chandrasekaran R.Y., Bradley J., Rubitski D., Berke H., Mente S., Butler T., Doran A., Cheng C., Fisher K. (2014). Casein Kinase 1δ/ε Inhibitor PF-5006739 Attenuates Opioid Drug-Seeking Behavior. ACS Chem. Neurosci..

[30] Cunningham P.S., Ahern S.A., Smith L.C., da Silva Santos C.S., Wager T.T., David A., Bechtold D.A. (2016). Targeting of the circadian clock via CK1δ/ε to improve glucose homeostasis in obesity. Sci. Rep..

[31] Němec V., Khirsariya P., Janovská P., Moyano P.M., Maier L., Procházková P., Kebková P., Gybel’ T., Berger B.T., Chaikuad A. (2023). Discovery of Potent and Exquisitely Selective Inhibitors of Kinase CK1 with Tunable Isoform Selectivity. Angew. Chem. Int. Ed. Engl..

[32] McCarver S., Hanna L., Samant A., Thompson A.A., Seierstad M., Saha A., Wu D., Lord B., Sutton S.W., Shah V. (2024). Structure-Based Optimization of Selective and Brain Penetrant CK1***δ*** Inhibitors for the Treatment of Circadian Disruptions. ACS Med. Chem. Lett..

[33] Lin J., Gaudreault F., Johnson N., Lin Z., Nouri P., Goosen T.C., Sawant-Basak A. (2022). Investigation of CYP3A induction by PF-05251749 in early clinical development: Comparison of linear slope physiologically based pharmacokinetic prediction and biomarker response. Clin. Transl. Sci..

[34] De Maeyer J., Geese M., Monecke S., Hirsch R., Loke P.L. (2020). Casein Kinase 1 Inhibitors for Use in the Treatment of Diseases Related to DUX4 Expression Such as Muscular Dystrophy and Cancer. International Patent.

[35] Bischof J., Leban J., Zaja M., Grothey A., Radunsky B., Othersen O., Strobl S., Vitt D., Knippschild U. (2012). 2-Benzamido-N-(1H-benzo[d]imidazol-2-yl)thiazole-4-carboxamide derivatives as potent inhibitors of CK1delta/epsilon. Amino Acids.

[36] Richter J., Bischof J., Zaja M., Kohlhof H., Othersen O., Vitt D., Alscher V., Pospiech I., García-Reyes B., Berg S. (2014). Difluoro-dioxolo-benzoimidazol-benzamides as potent inhibitors of CK1δ and ε with nanomolar inhibitory activity on cancer cell proliferation. J. Med. Chem..

[37] Calenda S., Catarzi D., Varano F., Vigiani E., Volpini R., Lambertucci C., Spinaci A., Trevisan L., Grieco I., Federico S. (2024). Structural Investigations on 2-Amidobenzimidazole Derivatives as New Inhibitors of Protein Kinase CK1 Delta. Pharmaceuticals.

[38] Morgenthaler M., Schweizer E., Hoffmann-Röder A., Benini F., Martin R.E., Jaeschke G., Wagner B., Fischer H., Bendels S., Zimmerli D. (2007). Predicting and Tuning Physicochemical Properties in Lead Optimization: Amine Basicities. ChemMedChem.

[39] Charifson P.S., Walters W.P. (2014). Acidic and Basic Drugs in Medicinal Chemistry: A Perspective. J. Med. Chem..

[40] Romanelli M.N., Manetti D., Braconi L., Dei S., Gabellini A., Teodori E. (2022). The piperazine scaffold for novel drug discovery efforts: The evidence to date. Expert Opin. Drug Discov..

[41] Kumari A., Singh R.K. (2020). Morpholine as ubiquitous pharmacophore in medicinal chemistry: Deep insight into the structure–activity relationship (SAR). Bioorg. Chem..

[42] Daina A., Michielin O., Zoete V. (2017). SwissADME: A free web tool to evaluate pharmacokinetics, drug-likeness and medicinal chemistry friendliness of small molecules. Sci. Rep..

[43] Tayeb B.A., Njangiru I.K., Minorics R. (2024). Beyond boundaries: Neuroprotective effects of steroids and ecdysteroids in SH-SY5Y cells—A systematic review. Neuroprotection.

[44] Artimagnella O., Floramo A., Cipriano G.L., Argento V., Lui M. (2026). Dopaminergic Identity of SH-SY5Y Cells Across Differentiation Protocols in Parkinson’s Disease Research: A Systematic Review. Int. J. Mol. Sci..

[45] Voogd E.J.H.F., Frega M., Hofmeijer J. (2023). Neuronal Responses to Ischemia: Scoping Review of Insights from Human-Derived In Vitro Models. Cell. Mol. Neurobiol..

[46] Wang X., Zhang M., Liu H. (2019). LncRNA17A regulates autophagy and apoptosis of SH-SY5Y cell line as an in vitro model for Alzheimer’s disease. Biosci. Biotechnol. Biochem..

[47] Lopez-Suarez L., Awabdh S.A., Coumoul X., Chauvet C. (2022). The SH-SY5Y human neuroblastoma cell line, a relevant in vitro cell model for investigating neurotoxicology in human: Focus on organic pollutants. Neurotoxicology.

[48] Grieco I., Bissaro M., Tiz D.B., Perez D.I., Perez C., Martinez A., Redenti S., Mariotto E., Bortolozzi R., Viola G. (2021). Developing novel classes of protein kinase CK1δ inhibitors by fusing [1,2,4]triazole with different bicyclic heteroaromatic systems. Eur. J. Med. Chem..

[49] Badura L., Swanson T., Adamowicz W., Adams J., Cianfrogna J., Fisher K., Holland J., Kleiman R., Nelson F., Reynolds L. (2007). An inhibitor of casein kinase I epsilon induces phase delays in circadian rhythms under free-running and entrained conditions. J. Pharmacol. Exp. Ther..

[50] MOE 2024.06 Release Notes. https://www.chemcomp.com/release_notes/moe202406/rnotes.htm.

[51] Korb O., Stützle T., Exner T.E. (2006). PLANTS: Application of Ant Colony Optimization to Structure-Based Drug Design. International Workshop on Ant Colony Optimization and Swarm Intelligence.

[52] Case D.A. (2022). AmberTools22. http://ambermd.org/doc12/Amber22.pdf.

[53] Harvey M.J., Giupponi G., Fabritiis G.D. (2009). ACEMD: Accelerating Biomolecular Dynamics in the Microsecond Time Scale. J. Chem. Theory Comput..

[54] Humphrey W., Dalke A., Schulten K. (1996). VMD: Visual Molecular Dynamics. J. Mol. Graph..

[55] Berman H.M., Westbrook J., Feng Z., Gilliland G., Bhat T.N., Weissig H., Shindyalov I.N., Bourne P.E. (2000). The Protein Data Bank. Nucleic Acids Res..

[56] (2023). Cadence Molecular Sciences|OpenEye. http://www.eyesopen.com.

[57] MPlayer Team MPlayer—The Movie Player. https://www.mplayerhq.hu/design7/dload.html.

[58] Gnuplot Development Team Gnuplot Documentation. https://gnuplot.sourceforge.net/docs/.

[59] The RDKit Documentation—The RDKit 2025.09.6 Documentation. https://www.rdkit.org/docs/index.html.

[60] Pettersen E.F., Goddard T.D., Huang C.C., Couch G.S., Greenblatt D.M., Meng E.C., Ferrin T.E. (2004). UCSF Chimera—A Visualization System for Exploratory Research and Analysis. J. Comput. Chem..

[61] Case D.A., Cheatham T.E., Darden T., Gohlke H., Luo R., Merz K.M., Onufriev A., Simmerling C., Wang B., Woods R.J. (2005). The Amber Biomolecular Simulation Programs. J. Comput. Chem..

[62] Maier J.A., Martinez C., Kasavajhala K., Wickstrom L., Hauser K.E., Simmerling C. (2015). ff14SB: Improving the Accuracy of Protein Side Chain and Backbone Parameters from ff99SB. J. Chem. Theory Comput..

[63] Wang J., Wolf R.M., Caldwell J.W., Kollman P.A., Case D.A. (2004). Development and Testing of a General Amber Force Field. J. Comput. Chem..

[64] Faller R., de Pablo J.J. (2002). Constant Pressure Hybrid Molecular Dynamics–Monte Carlo Simulations. J. Chem. Phys..

[65] Davidchack R.L., Handel R., Tretyakov M.V. (2009). Langevin Thermostat for Rigid Body Dynamics. J. Chem. Phys..

[66] Eastman P., Swails J., Chodera J.D., McGibbon R.T., Zhao Y., Beauchamp K.A., Wang L.P., Simmonett A.C., Harrigan M.P., Stern C.D. (2017). OpenMM 7: Rapid Development of High Performance Algorithms for Molecular Dynamics. PLoS Comput. Biol..

[67] Kräutler V., van Gunsteren W.F., Hünenberger P.H. (2001). A Fast SHAKE Algorithm to Solve Distance Constraint Equations for Small Molecules in Molecular Dynamics Simulations. J. Comput. Chem..

[68] Essmann U., Perera L., Berkowitz M.L., Darden T., Lee H., Pedersen L.G. (1995). A Smooth Particle Mesh Ewald Method. J. Chem. Phys..

[69] Michaud-Agrawal N., Denning E.J., Woolf T.B., Beckstein O. (2011). MDAnalysis: A Toolkit for the Analysis of Molecular Dynamics Simulations. J. Comput. Chem..

