# Supplementary material for: Novel Benzimidazol-2-Acetamide Derivatives as Submicromolar Casein Kinase 1δ Inhibitors with Favourable Predicted Drug-like Properties
Source: Pharmaceuticals (Basel). 2026 Oct 21;19(10):1666. doi: 10.3390/ph19101666 (PMC13708543; doi:10.3390/ph19101666)
Supplement: Supplementary file 1 [file pharmaceuticals-19-01666-s001.zip › Supplementary Materials.pdf]

Supplementary Materials

Novel Benzimidazol-2-acetamide Derivatives as Submicromolar Casein Kinase 1δ Inhibitors with Favorable Predicted Drug-Like Properties

Costanza Ceni, Sara Calenda, Giulia Vagnoni, Elisa Marinari, Daniela Catarzi, Flavia Varano, Letizia Trevisan, Michelle Fornasier, Stephanie Federico, Giampiero Spalluto, Gianluca Novello, Veronica Salmaso, Stefano Moro, Martino Calamai, Vittoria Colotta

Table of contents

-Figure S1: Molecular dynamics analysis of the reference inhibitor **Bischof-5** in complex with CK1δ (PDB: 4TWC), including binding pose, ligand–protein contact persistence, two-dimensional interaction diagram and per-atom RMSF).	Page S2
-Figure S2: Time profiles of the ligand RMSD computed for the solvent-exposed atom selection of compound **20** over three independent 100 ns MD replicas.	Page S2
- Figure S3: Time profiles of the ligand RMSD computed for the solvent-exposed atom selection of the reference inhibitor **Bischof-5** over three independent 100 ns MD replicas.	Page S3
-Predicted ADME properties using SwissADME.	Page S4-S36
-^{1}H-NMR and ^{13}C-APT NMR scanned spectra of compounds **1-32.**	Page S37-S100
-Table S1. Combustion Analysis data of the newly synthesized compounds.	Page. S101-S102

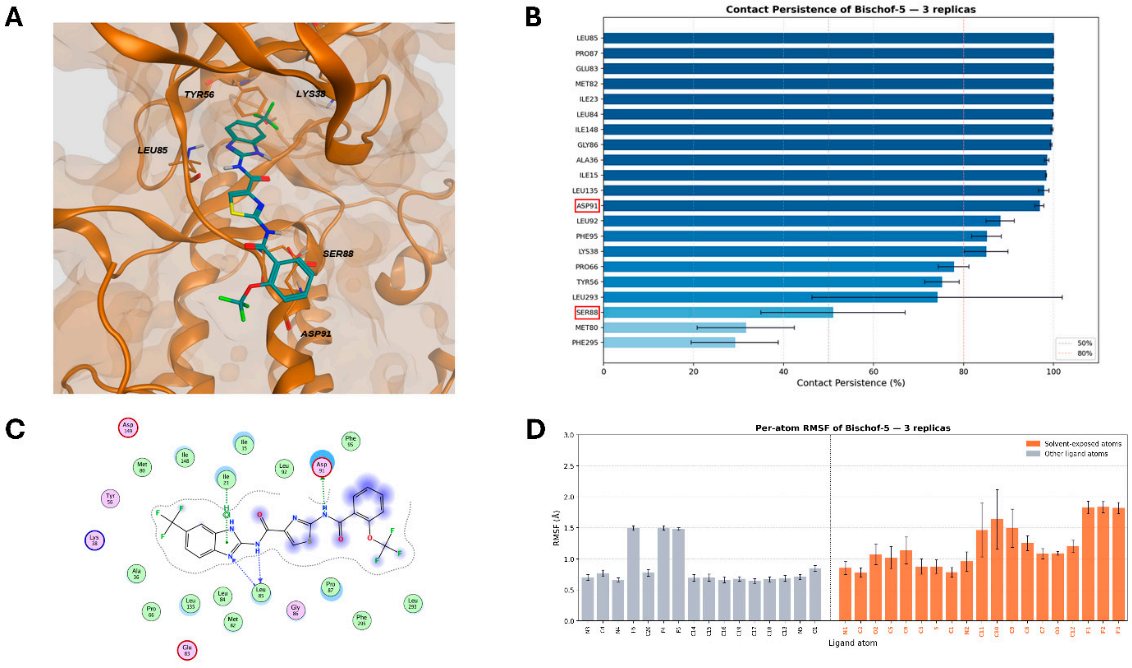

Figure S1. (A) Representative docking pose of compound **Bischof-5** in the ATP-binding site, showing the conserved hinge interactions and the orientation of the solvent-exposed substituent. (B) Ligand-protein contact persistence calculated over three independent 100 ns MD replicas. Error bars represent the standard deviation across replicas. (C) Two-dimensional ligand interaction diagram of compound **Bischof-5** in the CK1δ binding site: hydrogen bonds are drawn as dashed arrows, the residues lining the pocket are colored according to their hydrophobic (green) or polar (violet) character, and the dotted contour delimits the sol-vent-accessible surface of the ligand. (D) Per-atom RMSF of compound **Bischof-5**, computed for each of the three 100 ns MD replicas and reported as the mean over the replicas; error bars represent ±SD. Atoms are ordered from the core to the solvent-exposed substituent.

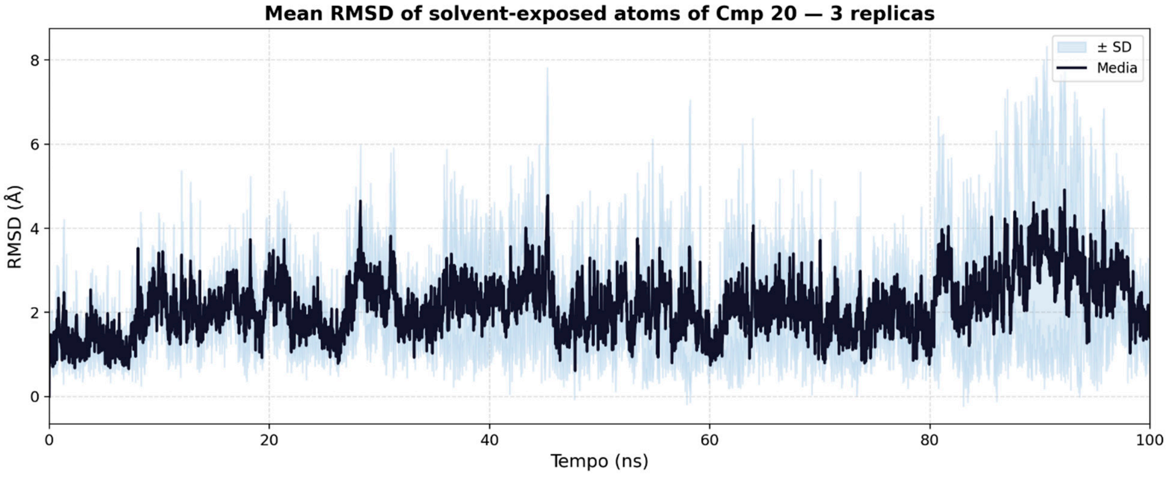

Figure S2. Mean RMSD of the solvent-exposed atoms of compound **20**, computed over three independent 100 ns MD replicas. The solid line represents the mean over the replicas and the shaded area the ±SD.

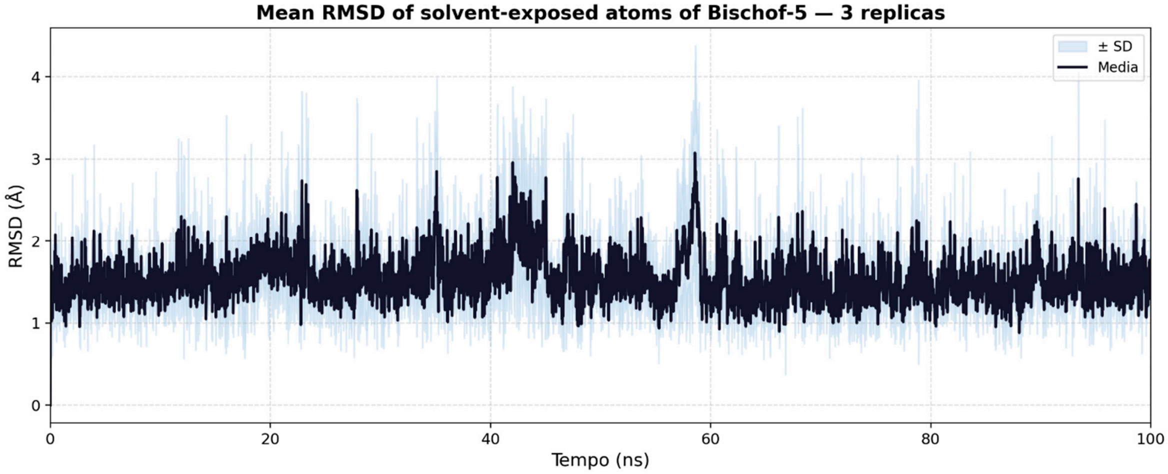

Figure S3. Mean RMSD of the solvent-exposed atoms of compound **Bischof-5**, computed over three independent 100 ns MD replicas. The solid line represents the mean over the replicas and the shaded area the ±SD.

ADME properties predicted using the web program SwissADME (http://www.swissadme.ch)

iLOGP: in-house physics-based method

XLOGP3: Atomistic andknowledge-based method calculated by XLOGP program

WLOGP: Atomistic method implemented from Wildman SA and CrippenGM. 1999 J. Chem. Inf.Model.

MLOGP: Topological method implemented

SILICOS-IT: Hybrid fragmental/topological method

Consensus LogP $_{o/w}$: Average of all five predictions

ESOL: Topological method

Solubility class: Log S scale Insoluble < -10 < Poorly < -6< Moderately < -4 < Soluble <-2 Very < 0 < Highly

Ali: Topological method

SILICOS-IT: Fragmental method calculated by FILTER-IT program

Gatrointestinal absorption: according to the white of theBOILED-Egg

BBB permeation: according to the yolk of the BOILED-Egg

P-glycoprotein substrate: SVM model built on 1033 molecules (training set) and tested on 415 molecules (test set) 10-fold CV: ACC=0.72 /AUC=0.77. External: ACC=0.88 /AUC=0.94

CYP1A2 (Cytochrome P450 1A2) inhibitor: SVM model built on 9145 molecules (training set) and tested on 3000 molecules (test set) 10-fold CV: ACC=0.83 /AUC=0.90. External: ACC=0.84 /AUC=0.91

CYP2C19 (Cytochrome P450 2C19) inhibitor: SVM model built on 9272 molecules (training set) and tested on 3000 molecules (test set) 10-fold CV: ACC=0.80 /AUC=0.86. External: ACC=0.80 /AUC=0.87

CYP2C9 (Cytochrome P450 2C9) inhibitor: SVM model built on 5940 molecules (training set) and tested on 2075 molecules (test set) 10-fold CV: ACC=0.78 /AUC=0.85. External: ACC=0.71 /AUC=0.81

CYP2D6 (Cytochrome P450 2D6) inhibitor: SVM model built on 3664 molecules (training set) and tested on 1068 molecules (test set) 10-fold CV: ACC=0.79 /AUC=0.85. External: ACC=0.81 /AUC=0.87

CYP3A4 (Cytochrome P450 3A4) inhibitor: SVM model built on 7518 molecules (training set) and tested on 2579 molecules (test set) 10-fold CV: ACC=0.77 /AUC=0.85. External: ACC=0.78 /AUC=0.86

Skin permeation: QSPR model

Lipinski (Pfizer) filter: MW ≤ 500 MLOGP ≤ 4.15 N or O ≤ 10 NH or OH ≤ 5

Ghose filter: 160 ≤ MW ≤ 480 -0.4 ≤ WLOGP ≤ 5.6, 40 ≤ MR ≤ 130, 20 ≤ atoms ≤ 70

Veber (GSK) filter: Rotatable bonds ≤ 10. TPSA ≤ 140

Egan (Pharmacia) filter: WLOGP ≤ 5.88. TPSA ≤ 131.6

Muegge (Bayer) filter: 200 ≤ MW ≤ 600, -2 ≤ XLOGP ≤ 5, TPSA ≤ 150, Num. rings ≤ 7, Num. carbon > 4, Num. heteroatoms > 1, Num. rotatable bonds ≤ 15, H-bond acc. ≤ 10, H-bond don. ≤ 5

Abbott Bioavailability Score: Probability of F > 10% in rat implemented from Martin YC. 2005 J. Med. Chem.

PAINS: Pan Assay Interference Structures

Brenk: Structural Alert

Leadlikeness: 250 ≤ MW ≤ 350, XLOGP ≤ 3.5, Num. rotatable bonds ≤ 7

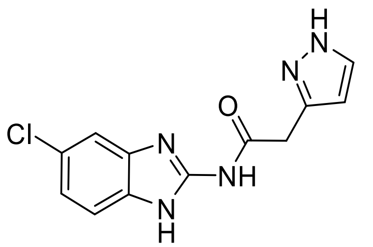

I

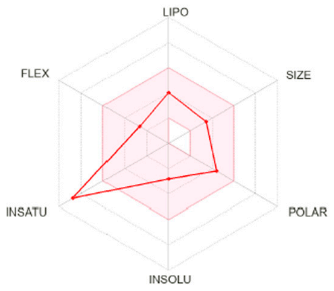

Physicochemical Properties
Formula: $C_{12}H_{10}ClN_5O$
Molecular weight: 275.69 g/mol
Num. heavy atoms: 19
Num. arom. heavy atoms: 14
Fraction Csp3: 0.08
Num. rotatable bonds: 4
Num. H-bond acceptors: 3
Num. H-bond donors: 3
Molar Refractivity: 72.05
TPSA (Topological Polar SurfaceArea): 86.46 Å²

Lipophilicity
LogP $_{o/w}$ (iLOGP): 0.07
LogP $_{o/w}$ (XLOGP3): 1.55
LogP $_{o/w}$ (WLOGP): 1.93
LogP $_{o/w}$ (MLOGP): 1.29
LogP $_{o/w}$ (SILICOS-IT): 2.61
Consensus LogP $_{o/w}$: 1.49

Water Solubility
Log S (ESOL): -2.81
Solubility: 4.30e^{-01} mg/ml; 1.56e^{-03} mol/l
Class: Soluble
Log S (Ali): -2.98
Solubility Class: 2.92e^{-01} mg/ml; 1.06e^{-03} mol/l
Class: Soluble
Log S (SILICOS-IT): -5.29
Solubility: 1.43e^{-03} mg/ml; 5.18e^{-06} mol/l
Class: Moderately soluble

Pharmacokinetics
GI absorption: High
BBB permeant: No
P-gp substrate: Yes
CYP1A2 inhibitor: Yes
CYP2C19 inhibitor: No
CYP2C9 inhibitor: No
CYP2D6 inhibitor: No
CYP3A4 inhibitor: No
Log K_p (skin permeation): -6.88 cm/s

Druglikeness
Lipinski: Yes; 0 violation
Ghose: Yes
Veber: Yes
Egan: Yes
Muegge: Yes
Bioavailability Score: 0.55

Medicinal Chemistry
PAINS: 0 alert
Brenk: 0 alert
Leadlikeness: Yes

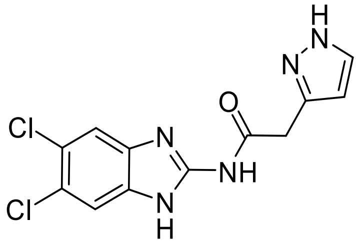

II

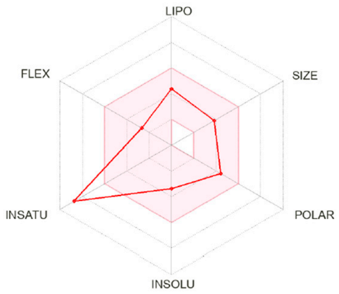

Physicochemical Properties
Formula: $C_{12}H_9Cl_2N_5O$
Molecular weight: 310.14 g/mol
Num. heavy atoms: 20
Num. arom. heavy atoms: 14
Fraction Csp3: 0.08
Num. rotatable bonds: 4
Num. H-bond acceptors: 3
Num. H-bond donors: 3
Molar Refractivity: 77.06
TPSA (Topological Polar SurfaceArea): 86.46 Å²

Lipophilicity
LogP $_{o/w}$ (iLOGP): 0.13
LogP $_{o/w}$ (XLOGP3): 2.18
LogP $_{o/w}$ (WLOGP): 2.58
LogP $_{o/w}$ (MLOGP): 1.82
LogP $_{o/w}$ (SILICOS-IT): 3.25
Consensus LogP $_{o/w}$: 1.99

Water Solubility
Log S (ESOL): -3.39
Solubility: 1.26e^{-01} mg/ml; 4.07e^{-04} mol/l
Class: Soluble
Log S (Ali): -3.63
Solubility: 7.29e^{-02} mg/ml; 2.35e^{-04} mol/l
Class: Soluble
Log S (SILICOS-IT): -5.89
Solubility: 4.01e^{-04} mg/ml; 1.29e^{-06} mol/l
Class: Moderately soluble

Pharmacokinetics
GI absorption: High
BBB permeant: No
P-gp substrate: Yes
CYP1A2 inhibitor: Yes
CYP2C19 inhibitor: No
CYP2C9 inhibitor: No
CYP2D6 inhibitor: No
CYP3A4 inhibitor: No
Log K_p (skin permeation): -6.64 cm/s

Druglikeness
Lipinski: Yes; 0 violation
Ghose: Yes
Veber: Yes
Egan: Yes
Muegge: Yes
Bioavailability Score: 0.55

Medicinal Chemistry
PAINS: 0 alert
Brenk: 0 alert
Leadlikeness: Yes

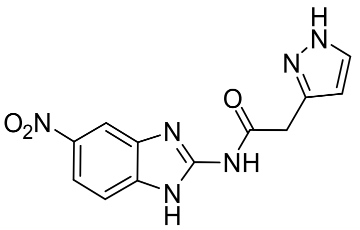

III

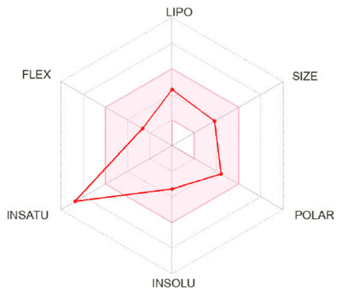

Physicochemical Properties
Formula: $C_{12}H_9Cl_2N_5O$
Molecular weight: 310.14 g/mol
Num. heavy atoms: 20
Num. arom. heavy atoms: 14
Fraction Csp3: 0.08
Num. rotatable bonds: 4
Num. H-bond acceptors: 3
Num. H-bond donors: 3
Molar Refractivity: 77.06
TPSA (Topological Polar SurfaceArea): 86.46 Å^2

Lipophilicity
LogP $_{o/w}$ (iLOGP): 0.13
LogP $_{o/w}$ (XLOGP3): 2.18
LogP $_{o/w}$ (WLOGP): 2.58
LogP $_{o/w}$ (MLOGP): 1.82
LogP $_{o/w}$ (SILICOS-IT): 3.25
Consensus LogP $_{o/w}$: 1.99

Water Solubility
Log S (ESOL): -3.39
Solubility: 1.26e^{-01} mg/ml; 4.07e^{-04} mol/l
Class: Soluble
Log S (Ali): -3.63
Solubility: 7.29e^{-02} mg/ml; 2.35e^{-04} mol/l
Class: Soluble
Log S (SILICOS-IT): -5.89
Solubility: 4.01e^{-04} mg/ml; 1.29e^{-06} mol/l
Class: Moderately soluble

Pharmacokinetics
GI absorption: High
BBB permeant: No
P-gp substrate: Yes
CYP1A2 inhibitor: Yes
CYP2C19 inhibitor: No
CYP2C9 inhibitor: No
CYP2D6 inhibitor: No
CYP3A4 inhibitor: No
Log K_p (skin permeation): -6.64 cm/s

Druglikeness
Lipinski: Yes; 0 violation
Ghose: Yes
Veber: Yes
Egan: Yes
Muegge: Yes
Bioavailability Score: 0.55

Medicinal Chemistry
PAINS: 0 alert
Brenk: 0 alert
Leadlikeness: Yes

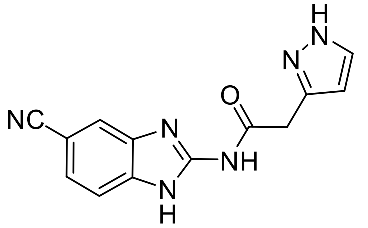

IV

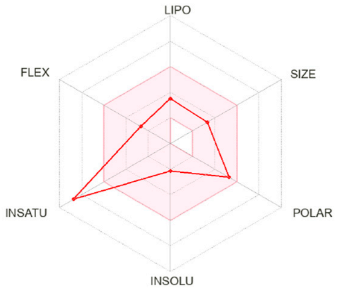

Physicochemical Properties
Formula: $C_{13}H_{10}N_6O$
Molecular weight: 266.26 g/mol
Num. heavy atoms: 20
Num. arom. heavy atoms: 14
Fraction Csp3: 0.08
Num. rotatable bonds: 4
Num. H-bond acceptors: 4
Num. H-bond donors: 3
Molar Refractivity: 71.75
TPSA (Topological Polar SurfaceArea): 110.25 Å²

Lipophilicity
LogP $_{o/w}$ (iLOGP): -0.32
LogP $_{o/w}$ (XLOGP3): 0.64
LogP $_{o/w}$ (WLOGP): 1.15
LogP $_{o/w}$ (MLOGP): 0.15
LogP $_{o/w}$ (SILICOS-IT): 1.97
Consensus LogP $_{o/w}$: 0.72

Water Solubility
Log S (ESOL): -2.15
Solubility: 1.89e^{+00} mg/ml; 7.11e^{-03} mol/l
Class: Soluble
Log S (Ali): -2.53
Solubility: 7.85e^{-01} mg/ml; 2.95e^{-03} mol/l
Class: Soluble
Log S (SILICOS-IT): -4.76
Solubility: 4.64e^{-03} mg/ml; 1.74e^{-05} mol/l
Class: Moderately soluble

Pharmacokinetics
GI absorption: High
BBB permeant: No
P-gp substrate: No
CYP1A2 inhibitor: Yes
CYP2C19 inhibitor: No
CYP2C9 inhibitor: No
CYP2D6 inhibitor: No
CYP3A4 inhibitor: No
Log K_p (skin permeation): -7.47 cm/s

Druglikeness
Lipinski: Yes; 0 violation
Ghose: Yes
Veber: Yes
Egan: Yes
Muegge: Yes
Bioavailability Score: 0.55

Medicinal Chemistry
PAINS: 0 alert
Brenk: 0 alert
Leadlikeness: Yes

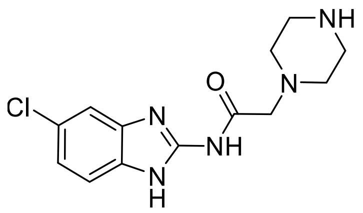

2

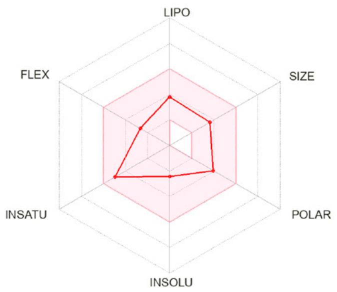

Physicochemical Properties
Formula: $C_{13}H_{16}ClN_5O$
Molecular weight: 293.75 g/mol
Num. heavy atoms: 20
Num. arom. heavy atoms: 9
Fraction Csp3: 0.38
Num. rotatable bonds: 4
Num. H-bond acceptors: 4
Num. H-bond donors: 3
Molar Refractivity: 86.06
TPSA (Topological Polar SurfaceArea): 73.05 Å^2

Lipophilicity
LogP $_{o/w}$ (iLOGP): 1.60
LogP $_{o/w}$ (XLOGP3): 1.15
LogP $_{o/w}$ (WLOGP): 0.11
LogP $_{o/w}$ (MLOGP): 0.94
LogP $_{o/w}$ (SILICOS-IT): 1.68
Consensus LogP $_{o/w}$: 1.10

Water Solubility
Log S (ESOL): -2.45
Solubility: 1.03e^{+00} mg/ml; 3.51e^{-03} mol/l
Class: Soluble
Log S (Ali): -2.28
Solubility: 1.55e^{+00} mg/ml; 5.26e^{-03} mol/l
Class: Soluble
Log S (SILICOS-IT): -4.42
Solubility: 1.11e^{-02} mg/ml; 3.79e^{-05} mol/l
Class: Moderately soluble

Pharmacokinetics
GI absorption: High
BBB permeant: No
P-gp substrate: Yes
CYP1A2 inhibitor: Yes
CYP2C19 inhibitor: No
CYP2C9 inhibitor: No
CYP2D6 inhibitor: No
CYP3A4 inhibitor: No
Log K_p (skin permeation): -7.28 cm/s

Druglikeness
Lipinski: Yes; 0 violation
Ghose: Yes
Veber: Yes
Egan: Yes
Muegge: Yes
Bioavailability Score: 0.55

Medicinal Chemistry
PAINS: 0 alert
Brenk: 0 alert
Leadlikeness: Yes

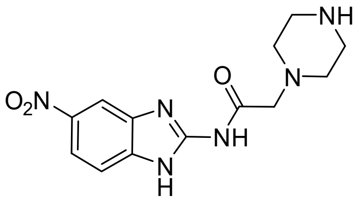

4

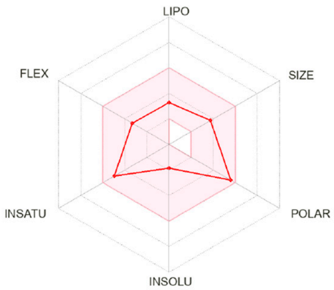

Physicochemical Properties
Formula: $C_{13}H_{16}N_6O_3$
Molecular weight: 304.30 g/mol
Num. heavy atoms: 22
Num. arom. heavy atoms: 9
Fraction Csp3: 0.38
Num. rotatable bonds: 5
Num. H-bond acceptors: 6
Num. H-bond donors: 3
Molar Refractivity: 89.87
TPSA (Topological Polar SurfaceArea): 118.87 Å²

Lipophilicity
LogP $_{o/w}$ (iLOGP): 1.20
LogP $_{o/w}$ (XLOGP3): 0.24
LogP $_{o/w}$ (WLOGP): -0.64
LogP $_{o/w}$ (MLOGP): -0.49
LogP $_{o/w}$ (SILICOS-IT): -1.12
Consensus LogP $_{o/w}$: -0.16

Water Solubility
Log S (ESOL): -1.85
Solubility: $4.29e^{+00}$ mg/ml; $1.41e^{-02}$ mol/l
Class: Very soluble
Log S (Ali): -2.30
Solubility: $1.54e^{+00}$ mg/ml; $5.05e^{-03}$ mol/l
Class: Soluble
Log S (SILICOS-IT): -3.18
Solubility: $2.01e^{-01}$ mg/ml ; $6.59e^{-04}$ mol/l
Class: Soluble

Pharmacokinetics
GI absorption: High
BBB permeant: No
P-gp substrate: Yes
CYP1A2 inhibitor: Yes
CYP2C19 inhibitor: No
CYP2C9 inhibitor: No
CYP2D6 inhibitor: No
CYP3A4 inhibitor: No
Log K_p (skin permeation): -7.99 cm/s

Druglikeness
Lipinski: Yes; 0 violation
Ghose: 1 violation: WLOGP<-0.4
Veber: Yes
Egan: Yes
Muegge: Yes
Bioavailability Score: 0.55

Medicinal Chemistry
PAINS: 0 alert
Brenk: 2 alerts: nitro group, oxygen-nitrogen single bond
Leadlikeness: Yes

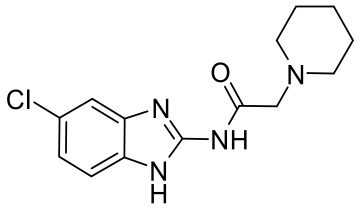

6

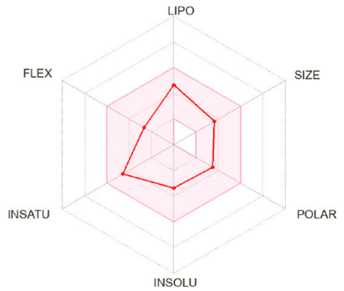

Physicochemical Properties
Formula: $C_{14}H_{17}ClN_4O$
Molecular weight: 292.76 g/mol
Num. heavy atoms: 20
Num. arom. heavy atoms: 9
Fraction Csp3: 0.43
Num. rotatable bonds: 4
Num. H-bond acceptors: 3
Num. H-bond donors: 2
Molar Refractivity: 84.15
TPSA (Topological Polar SurfaceArea): 61.02 Å²

Lipophilicity
LogP $_{o/w}$ (iLOGP): 2.14
LogP $_{o/w}$ (XLOGP3): 2.65
LogP $_{o/w}$ (WLOGP): 2.07
LogP $_{o/w}$ (MLOGP): 2.01
LogP $_{o/w}$ (SILICOS-IT): 2.61
Consensus LogP $_{o/w}$: 2.30

Water Solubility
Log S (ESOL): -3.39
Solubility: 1.18e^{-01} mg/ml; 4.04e^{-04} mol/l
Class: Soluble
Log S (Ali): -3.58
Solubility: 7.66e^{-02} mg/ml; 2.62e^{-04} mol/l
Class: Soluble
Log S (SILICOS-IT): -4.65
Solubility: 6.57e^{-03} mg/ml; 2.24e^{-05} mol/l
Class: Moderately soluble

Pharmacokinetics
GI absorption: High
BBB permeant: Yes
P-gp substrate: Yes
CYP1A2 inhibitor: Yes
CYP2C19 inhibitor: No
CYP2C9 inhibitor: No
CYP2D6 inhibitor: Yes
CYP3A4 inhibitor: No
Log K_p (skin permeation): -6.20 cm/s

Druglikeness
Lipinski: Yes; 0 violation
Ghose: Yes
Veber: Yes
Egan: Yes
Muegge: Yes
Bioavailability Score: 0.55

Medicinal Chemistry
PAINS: 0 alert
Brenk: 0 alert
Leadlikeness: Yes

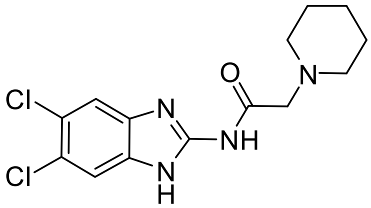

7

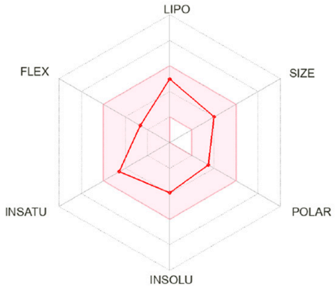

Physicochemical Properties
Formula: $C_{14}H_{16}Cl_2N_4O$
Molecular weight: 327.21 g/mol
Num. heavy atoms: 21
Num. arom. heavy atoms: 9
Fraction Csp3: 0.43
Num. rotatable bonds: 4
Num. H-bond acceptors: 3
Num. H-bond donors: 2
Molar Refractivity: 89.16
TPSA (Topological Polar SurfaceArea): 61.02 Å²

Lipophilicity
LogP $_{o/w}$ (iLOGP): 2.30
LogP $_{o/w}$ (XLOGP3): 3.17
LogP $_{o/w}$ (WLOGP): 2.72
LogP $_{o/w}$ (MLOGP): 2.52
LogP $_{o/w}$ (SILICOS-IT): 3.25
Consensus LogP $_{o/w}$: 2.79

Water Solubility
Log S (ESOL): -3.92
Solubility: 3.94e^{-02} mg/ml; 1.21e^{-04} mol/l
Class: Soluble
Log S (Ali): -4.12
Solubility: 2.47e^{-02} mg/ml; 7.55e^{-05} mol/l
Class: Moderately soluble
Log S (SILICOS-IT): -5.25
Solubility: 1.85e^{-03} mg/ml; 5.65e^{-06} mol/l
Class: Moderately soluble

Pharmacokinetics
GI absorption: High
BBB permeant: Yes
P-gp substrate: Yes
CYP1A2 inhibitor: Yes
CYP2C19 inhibitor: No
CYP2C9 inhibitor: No
CYP2D6 inhibitor: Yes
CYP3A4 inhibitor: No
Log K_p (skin permeation): -6.05 cm/s

Druglikeness
Lipinski: Yes; 0 violation
Ghose: Yes
Veber: Yes
Egan: Yes
Muegge: Yes
Bioavailability Score: 0.55

Medicinal Chemistry
PAINS: 0 alert
Brenk: 0 alert
Leadlikeness: Yes

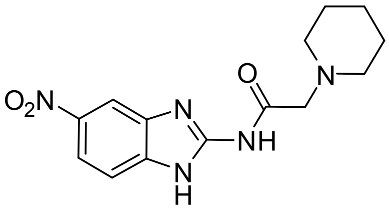

8

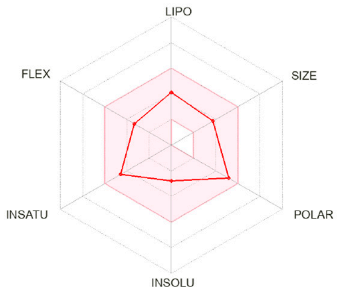

Physicochemical Properties
Formula: $C_{14}H_{17}N_5O_3$
Molecular weight: 303.32 g/mol
Num. heavy atoms: 21
Num. arom. heavy atoms: 9
Fraction Csp3: 0.43
Num. rotatable bonds: 5
Num. H-bond acceptors: 5
Num. H-bond donors: 2
Molar Refractivity: 87.96
TPSA (Topological Polar SurfaceArea): 106.84 Å²

Lipophilicity
LogP $_{o/w}$ (iLOGP): 1.48
LogP $_{o/w}$ (XLOGP3): 1.74
LogP $_{o/w}$ (WLOGP): 1.32
LogP $_{o/w}$ (MLOGP): 0.56
LogP $_{o/w}$ (SILICOS-IT): -0.20
Consensus LogP $_{o/w}$: 0.98

Water Solubility
Log S (ESOL): -2.79
Solubility: $4.93e^{-01}$ mg/ml; $1.62e^{-03}$ mol/l
Class: Soluble
Log S (Ali): -3.60
Solubility: $7.61e^{-02}$ mg/ml; $2.51e^{-04}$ mol/l
Class: Soluble
Log S (SILICOS-IT): -3.41
Solubility: $1.18e^{-01}$ mg/ml; $3.90e^{-04}$ mol/l
Class: Soluble

Pharmacokinetics
GI absorption: High
BBB permeant: No
P-gp substrate: Yes
CYP1A2 inhibitor: Yes
CYP2C19 inhibitor: No
CYP2C9 inhibitor: No
CYP2D6 inhibitor: No
CYP3A4 inhibitor: No
Log K_p (skin permeation): -6.91 cm/s

Druglikeness
Lipinski: Yes; 0 violation
Ghose: Yes
Veber: Yes
Egan: Yes
Muegge: Yes
Bioavailability Score: 0.55

Medicinal Chemistry
PAINS: 0 alert
Brenk: nitro group, oxygen-nitrogen single bond
Leadlikeness: Yes

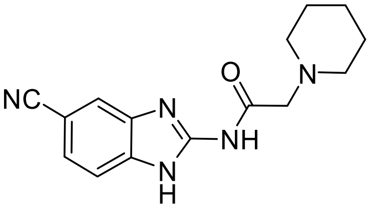

9

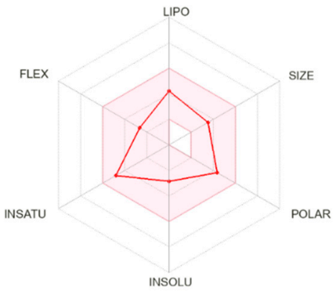

Physicochemical Properties
Formula: $C_{15}H_{17}N_5O$
Molecular weight: 283.33 g/mol
Num. heavy atoms: 21
Num. arom. heavy atoms: 9
Fraction Csp3: 0.40
Num. rotatable bonds: 4
Num. H-bond acceptors: 4
Num. H-bond donors: 2
Molar Refractivity: 83.85
TPSA (Topological Polar SurfaceArea): 84.81 Å²

Lipophilicity
LogP $_{o/w}$ (iLOGP): 1.64
LogP $_{o/w}$ (XLOGP3): 1.90
LogP $_{o/w}$ (WLOGP): 1.29
LogP $_{o/w}$ (MLOGP): 0.85
LogP $_{o/w}$ (SILICOS-IT): 1.99
Consensus LogP $_{o/w}$: 1.53

Water Solubility
Log S (ESOL): -2.85
Solubility: 4.03e^{-01} mg/ml; 1.42e^{-03} mol/l
Class: Soluble
Log S (Ali): -3.30
Solubility: 1.41e^{-01} mg/ml; 4.97e^{-04} mol/l
Class: Soluble
Log S (SILICOS-IT): -4.12
Solubility: 2.13e^{-02} mg/ml; 7.53e^{-05} mol/l
Class: Moderately soluble

Pharmacokinetics
GI absorption: High
BBB permeant: No
P-gp substrate: Yes
CYP1A2 inhibitor: Yes
CYP2C19 inhibitor: No
CYP2C9 inhibitor: No
CYP2D6 inhibitor: No
CYP3A4 inhibitor: No
Log K_p (skin permeation): -6.68 cm/s

Druglikeness
Lipinski: Yes; 0 violation
Ghose: Yes
Veber: Yes
Egan: Yes
Muegge: Yes
Bioavailability Score: 0.55

Medicinal Chemistry
PAINS: 0 alert
Brenk: nitro group, oxygen-nitrogen single bond
Leadlikeness: Yes

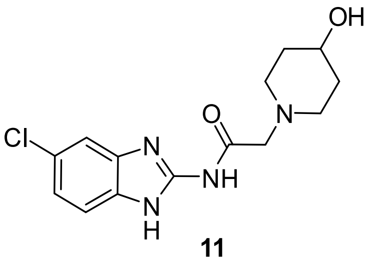

11

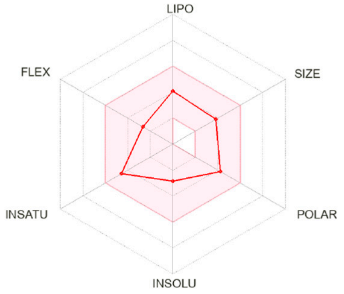

Physicochemical Properties
Formula: $C_{14}H_{17}ClN_4O_2$
Molecular weight: 308.76 g/mol
Num. heavy atoms: 21
Num. arom. heavy atoms: 9
Fraction Csp3: 0.43
Num. rotatable bonds: 4
Num. H-bond acceptors: 4
Num. H-bond donors: 3
Molar Refractivity: 85.21
TPSA (Topological Polar SurfaceArea): 81.25 Å²

Lipophilicity
LogP $_{o/w}$ (iLOGP): 1.53
LogP $_{o/w}$ (XLOGP3): 1.68
LogP $_{o/w}$ (WLOGP): 1.04
LogP $_{o/w}$ (MLOGP): 1.19
LogP $_{o/w}$ (SILICOS-IT): 1.71
Consensus LogP $_{o/w}$: 1.43

Water Solubility
Log S (ESOL): -2.87
Solubility: 4.20e^{-01} mg/ml; 1.36e^{-03} mol/l
Class: Soluble
Log S (Ali): -3.00
Solubility: 3.08e^{-01} mg/ml; 9.98e^{-04} mol/l
Class: Soluble
Log S (SILICOS-IT): -3.84
Solubility: 4.50e^{-02} mg/ml; 1.46e^{-04} mol/l
Class: Soluble

Pharmacokinetics
GI absorption: High
BBB permeant: No
P-gp substrate: Yes
CYP1A2 inhibitor: Yes
CYP2C19 inhibitor: No
CYP2C9 inhibitor: No
CYP2D6 inhibitor: No
CYP3A4 inhibitor: No
Log K_p (skin permeation): -6.9cm/s

Druglikeness
Lipinski: Yes; 0 violation
Ghose: Yes
Veber: Yes
Egan: Yes
Muegge: Yes
Bioavailability Score: 0.55

Medicinal Chemistry
PAINS: 0 alert
Brenk: Yes
Leadlikeness: Yes

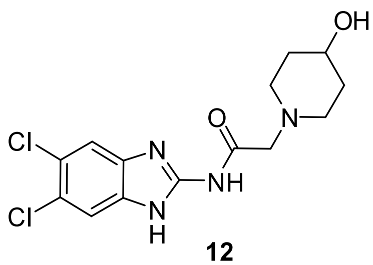

12

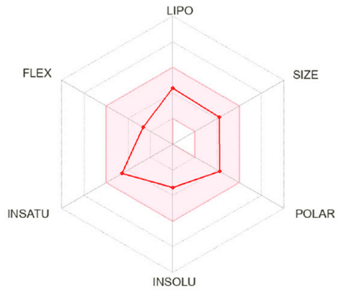

Physicochemical Properties
Formula: $C_{14}H_{17}Cl_2N_4O_2$
Molecular weight: 343.21 g/mol
Num. heavy atoms: 22
Num. arom. heavy atoms: 9
Fraction Csp3: 0.43
Num. rotatable bonds: 4
Num. H-bond acceptors: 4
Num. H-bond donors: 3
Molar Refractivity: 90.32
TPSA (Topological Polar SurfaceArea): 81.25 Å²

Lipophilicity
LogP $_{o/w}$ (iLOGP): 1.82
LogP $_{o/w}$ (XLOGP3): 2.19
LogP $_{o/w}$ (WLOGP): 1.69
LogP $_{o/w}$ (MLOGP): 1.71
LogP $_{o/w}$ (SILICOS-IT): 2.35
Consensus LogP $_{o/w}$: 1.95

Water Solubility
Log S (ESOL): -3.39
Solubility: 1.41e^{-01} mg/ml; 4.11e^{-04} mol/l
Class: Soluble
Log S (Ali): -3.53
Solubility: 1.01e^{-01} mg/ml; 2.95e^{-04} mol/l
Class: Soluble
Log S (SILICOS-IT): -4.43
Solubility: 1.27e^{-02} mg/ml; 3.70e^{-05} mol/l
Class: Moderately soluble

Pharmacokinetics
GI absorption: High
BBB permeant: No
P-gp substrate: Yes
CYP1A2 inhibitor: Yes
CYP2C19 inhibitor: No
CYP2C9 inhibitor: No
CYP2D6 inhibitor: Yes
CYP3A4 inhibitor: No
Log K_p (skin permeation): -6.84 cm/s

Druglikeness
Lipinski: Yes; 0 violation
Ghose: Yes
Veber: Yes
Egan: Yes
Muegge: Yes
Bioavailability Score: 0.55

Medicinal Chemistry
PAINS: 0 alert
Brenk: 0 alert
Leadlikeness: Yes

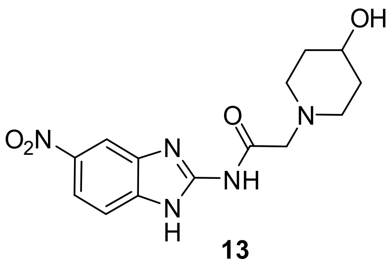

13

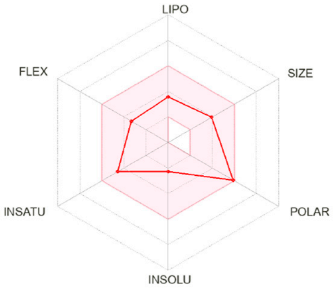

Physicochemical Properties
Formula: $C_{14}H_{17}N_5O_4$
Molecular weight: 319.32 g/mol
Num. heavy atoms: 23
Num. arom. heavy atoms: 9
Fraction Csp3: 0.43
Num. rotatable bonds: 5
Num. H-bond acceptors: 6
Num. H-bond donors: 3
Molar Refractivity: 89.12
TPSA (Topological Polar SurfaceArea): 127.07 $Å^2$

Lipophilicity
LogP $_{o/w}$ (iLOGP): 0.96
LogP $_{o/w}$ (XLOGP3): 0.76
LogP $_{o/w}$ (WLOGP): 0.29
LogP $_{o/w}$ (MLOGP): -0.22
LogP $_{o/w}$ (SILICOS-IT): -1.08
Consensus LogP $_{o/w}$: 0.14

Water Solubility
Log S (ESOL): -2.26
Solubility: $1.76e^{+00}$ mg/ml; $5.52e^{-03}$ mol/l
Class: Soluble
Log S (Ali): -3.01
Solubility: $3.13e^{-01}$ mg/ml; $9.81e^{-04}$ mol/l
Class: Soluble
Log S (SILICOS-IT): -2.60
Solubility: $8.11e^{-01}$ mg/ml; $2.54e^{-03}$ mol/l
Class: Soluble

Pharmacokinetics
GI absorption: High
BBB permeant: No
P-gp substrate: Yes
CYP1A2 inhibitor: No
CYP2C19 inhibitor: No
CYP2C9 inhibitor: No
CYP2D6 inhibitor: No
CYP3A4 inhibitor: No
Log K_p (skin permeation): -7.71 cm/s

Druglikeness
Lipinski: Yes; 0 violation
Ghose: Yes
Veber: Yes
Egan: Yes
Muegge: Yes
Bioavailability Score: 0.55

Medicinal Chemistry
PAINS: 0 alert
Brenk: nitro group, oxygen-nitrogen single bond
Leadlikeness: Yes

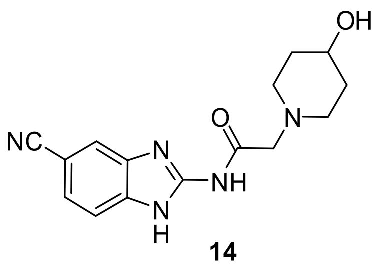
14

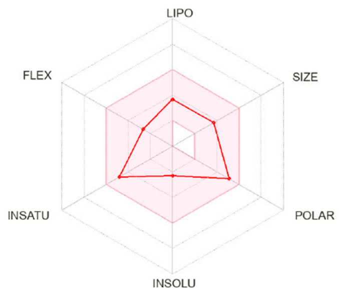

Physicochemical Properties
Formula: $C_{15}H_{17}N_5O_2$
Molecular weight: 299.33 g/mol
Num. heavy atoms: 22
Num. arom. heavy atoms: 9
Fraction Csp3: 0.40
Num. rotatable bonds: 4
Num. H-bond acceptors: 5
Num. H-bond donors: 3
Molar Refractivity: 85.02
TPSA (Topological Polar SurfaceArea): 105.04 Å²

Lipophilicity
LogP $_{o/w}$ (iLOGP): 1.35
LogP $_{o/w}$ (XLOGP3): 0.93
LogP $_{o/w}$ (WLOGP): 0.26
LogP $_{o/w}$ (MLOGP): 0.05
LogP $_{o/w}$ (SILICOS-IT): 1.09
Consensus LogP $_{o/w}$: 0.73

Water Solubility
Log S (ESOL): -2.32
Solubility: 1.43e^{+00} mg/ml; 4.78e^{-03} mol/l
Class: Soluble
Log S (Ali): -2.72
Solubility: 5.68e^{-01} mg/ml; 1.90e^{-03} mol/l
Class: Soluble
Log S (SILICOS-IT): -3.31
Solubility: 1.46e^{-01} mg/ml; 4.89e^{-04} mol/l
Class: Soluble

Pharmacokinetics
GI absorption: High
BBB permeant: No
P-gp substrate: Yes
CYP1A2 inhibitor: No
CYP2C19 inhibitor: No
CYP2C9 inhibitor: No
CYP2D6 inhibitor: No
CYP3A4 inhibitor: No
Log K_p (skin permeation): -7.47 cm/s

Druglikeness
Lipinski: Yes; 0 violation
Ghose: Yes
Veber: Yes
Egan: Yes
Muegge: Yes
Bioavailability Score: 0.55

Medicinal Chemistry
PAINS: 0 alert
Brenk: 0 alert
Leadlikeness: Yes

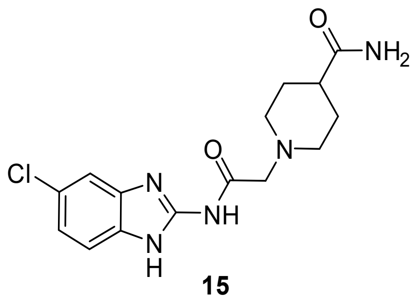

15

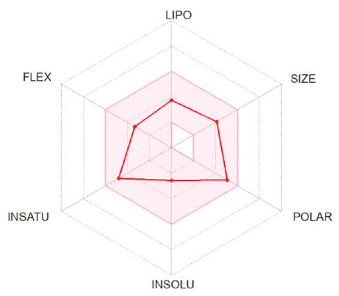

Physicochemical Properties
Formula: $C_{15}H_{18}ClN_5O_2$
Molecular weight: 335.79 g/mol
Num. heavy atoms: 23
Num. arom. heavy atoms: 9
Fraction Csp3: 0.40
Num. rotatable bonds: 5
Num. H-bond acceptors: 4
Num. H-bond donors: 3
Molar Refractivity: 91.86
TPSA (Topological Polar SurfaceArea): 104.11 Å^2

Lipophilicity
LogP $_{o/w}$ (iLOGP): 1.45
LogP $_{o/w}$ (XLOGP3): 1.06
LogP $_{o/w}$ (WLOGP): 0.78
LogP $_{o/w}$ (MLOGP): 0.97
LogP $_{o/w}$ (SILICOS-IT): 1.39
Consensus LogP $_{o/w}$: 1.13

Water Solubility
Log S (ESOL): -2.55
Solubility: 9.48e^{-01} mg/ml; 2.82e^{-03} mol/l
Class: Soluble
Log S (Ali): -2.84
Solubility: 4.88e^{-01} mg/ml; 1.45e^{-03} mol/l
Class: Soluble
Log S (SILICOS-IT): -3.99
Solubility: 3.47e^{-02} mg/ml; 1.03e^{-04} mol/l
Class: Soluble

Pharmacokinetics
GI absorption: High
BBB permeant: No
P-gp substrate: Yes
CYP1A2 inhibitor: No
CYP2C19 inhibitor: No
CYP2C9 inhibitor: No
CYP2D6 inhibitor: No
CYP3A4 inhibitor: No
Log K_p (skin permeation): -7.60 cm/s

Druglikeness
Lipinski: Yes; 0 violation
Ghose: Yes
Veber: Yes
Egan: Yes
Muegge: Yes
Bioavailability Score: 0.55

Medicinal Chemistry
PAINS: 0 alert
Brenk: 0 alert
Leadlikeness: Yes

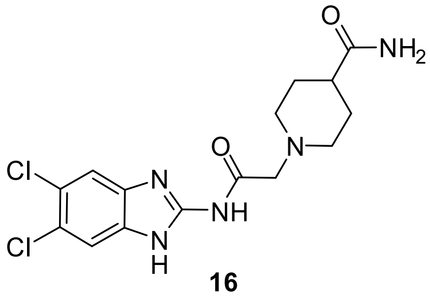

16

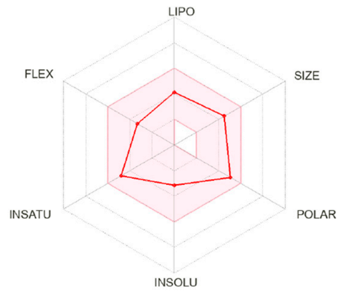

Physicochemical Properties
Formula: $C_{15}H_{17}Cl_2N_5O_2$
Molecular weight: 370.23 g/mol
Num. heavy atoms: 24
Num. arom. heavy atoms: 9
Fraction Csp3: 0.40
Num. rotatable bonds: 5
Num. H-bond acceptors: 4
Num. H-bond donors: 3
Molar Refractivity: 96.87
TPSA (Topological Polar SurfaceArea): 104.11 Å^2

Lipophilicity
LogP $_{o/w}$ (iLOGP): 1.57
LogP $_{o/w}$ (XLOGP3): 1.69
LogP $_{o/w}$ (WLOGP): 1.43
LogP $_{o/w}$ (MLOGP): 1.48
LogP $_{o/w}$ (SILICOS-IT): 2.04
Consensus LogP $_{o/w}$: 1.64

Water Solubility
Log S (ESOL): -3.15
Solubility: 2.64e^{-01} mg/ml; 7.12e^{-04} mol/l
Class: Soluble
Log S (Ali): -3.49
Solubility: 1.19e^{-01} mg/ml; 3.23e^{-04} mol/l
Class: Soluble
Log S (SILICOS-IT): -4.58
Solubility: 9.78e^{-03} mg/ml; 2.64e^{-05} mol/l
Class: Moderately soluble

Pharmacokinetics
GI absorption: High
BBB permeant: No
P-gp substrate: Yes
CYP1A2 inhibitor: Yes
CYP2C19 inhibitor: No
CYP2C9 inhibitor: No
CYP2D6 inhibitor: No
CYP3A4 inhibitor: No
Log K_p (skin permeation): -7.36 cm/s

Druglikeness
Lipinski: Yes; 0 violation
Ghose: Yes
Veber: Yes
Egan: Yes
Muegge: Yes
Bioavailability Score: 0.55

Medicinal Chemistry
PAINS: 0 alert
Brenk: 0 alert
Leadlikeness: No, 1 violation: MW>350

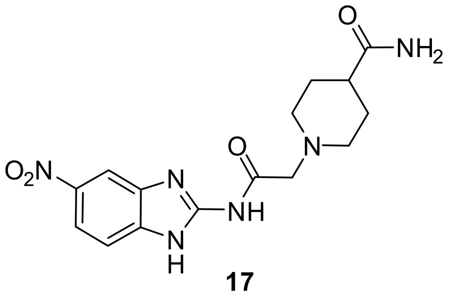

17

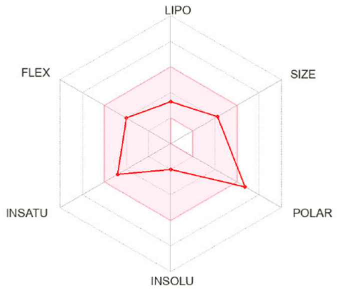

Physicochemical Properties
Formula: $C_{15}H_{18}N_6O_4$
Molecular weight: 346.34 g/mol
Num. heavy atoms: 25
Num. arom. heavy atoms: 9
Fraction Csp3: 0.40
Num. rotatable bonds: 6
Num. H-bond acceptors: 6
Num. H-bond donors: 3
Molar Refractivity: 95.67
TPSA (Topological Polar SurfaceArea): 149.93 Å^2

Lipophilicity
LogP $_{o/w}$ (iLOGP): 0.90
LogP $_{o/w}$ (XLOGP3): 0.26
LogP $_{o/w}$ (WLOGP): 0.04
LogP $_{o/w}$ (MLOGP): -0.41
LogP $_{o/w}$ (SILICOS-IT): -1.40
Consensus LogP $_{o/w}$: -0.12

Water Solubility
Log S (ESOL): -2.02
Solubility: 3.30e^{+00} mg/ml; 9.52e^{-03} mol/l
Class: Soluble
Log S (Ali): -2.97
Solubility: 3.72e^{-01} mg/ml; 1.07e^{-03} mol/l
Class: Soluble
Log S (SILICOS-IT): -2.74
Solubility: 6.26e^{-01} mg/ml; 1.81e^{-03} mol/l
Class: Soluble

Pharmacokinetics
GI absorption: Low
BBB permeant: No
P-gp substrate: Yes
CYP1A2 inhibitor: No
CYP2C19 inhibitor: No
CYP2C9 inhibitor: No
CYP2D6 inhibitor: No
CYP3A4 inhibitor: No
Log K_p (skin permeation): -8.23 cm/s

Druglikeness
Lipinski: Yes; 0 violation
Ghose: Yes
Veber: No; 1 violation: TPSA>140
Egan: No; 1 violation: TPSA>131.6
Muegge: Yes
Bioavailability Score: 0.55

Medicinal Chemistry
PAINS: 0 alert
Brenk: nitro group, oxygen-nitrogen single bond
Leadlikeness: Yes

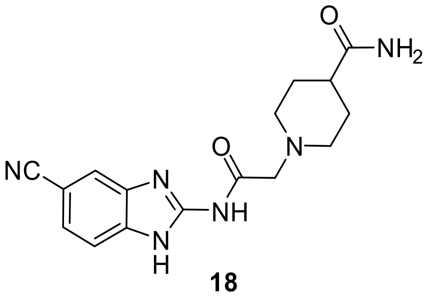

18

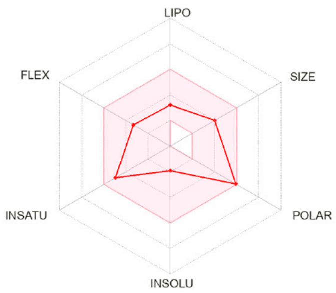

Physicochemical Properties
Formula: $C_{16}H_{18}N_6O_2$
Molecular weight: 326.35 g/mol
Num. heavy atoms: 24
Num. arom. heavy atoms: 9
Fraction Csp3: 0.38
Num. rotatable bonds: 5
Num. H-bond acceptors: 5
Num. H-bond donors: 3
Molar Refractivity: 91.57
TPSA (Topological Polar SurfaceArea): 127.90 Å²

Lipophilicity
LogP $_{o/w}$ (iLOGP): 1.12
LogP $_{o/w}$ (XLOGP3): 0.15
LogP $_{o/w}$ (WLOGP): -0.00
LogP $_{o/w}$ (MLOGP): -0.16
LogP $_{o/w}$ (SILICOS-IT): 0.78
Consensus LogP $_{o/w}$: 0.38

Water Solubility
Log S (ESOL): -1.91
Solubility: 4.06e^{+00} mg/ml; 1.24e^{-02} mol/l
Class: Very soluble
Log S (Ali): -2.39
Solubility: 1.32e^{+00} mg/ml; 4.05e^{-03} mol/l
Class: Soluble
Log S (SILICOS-IT): -3.46
Solubility: 1.13e^{-01} mg/ml; 3.45e^{-04} mol/l
Class: Soluble

Pharmacokinetics
GI absorption: High
BBB permeant: No
P-gp substrate: Yes
CYP1A2 inhibitor: No
CYP2C19 inhibitor: No
CYP2C9 inhibitor: No
CYP2D6 inhibitor: No
CYP3A4 inhibitor: No
Log K_p (skin permeation): -8.18 cm/s

Druglikeness
Lipinski: Yes; 0 violation
Ghose: Yes
Veber: Yes
Egan: Yes
Muegge: Yes
Bioavailability Score: 0.55

Medicinal Chemistry
PAINS: 0 alert
Brenk: 0 alert
Leadlikeness: Yes

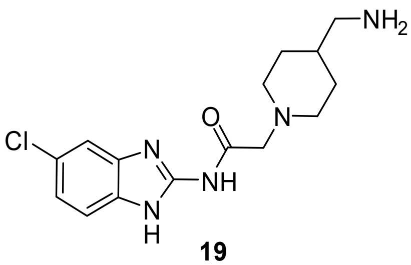

19

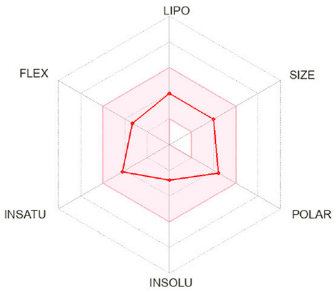

Physicochemical Properties
Formula: $C_{15}H_{20}ClN_5O$
Molecular weight: 321.81 g/mol
Num. heavy atoms: 22
Num. arom. heavy atoms: 9
Fraction Csp3: 0.47
Num. rotatable bonds: 5
Num. H-bond acceptors: 4
Num. H-bond donors: 3
Molar Refractivity: 91.66
TPSA (Topological Polar SurfaceArea): 87.04 Å^2

Lipophilicity
LogP $_{o/w}$ (iLOGP): 1.69
LogP $_{o/w}$ (XLOGP3): 0.15
LogP $_{o/w}$ (WLOGP): -0.00
LogP $_{o/w}$ (MLOGP): -0.16
LogP $_{o/w}$ (SILICOS-IT): 0.78
Consensus LogP $_{o/w}$: 0.38

Water Solubility
Log S (ESOL): -1.91
Solubility: 4.06e^{+00} mg/ml; 1.24e^{-02} mol/l
Class: Very soluble
Log S (Ali): -2.39
Solubility: 1.32e^{+00} mg/ml; 4.05e^{-03} mol/l
Class: Soluble
Log S (SILICOS-IT): -3.46
Solubility: 1.13e^{-01} mg/ml; 3.45e^{-04} mol/l
Class: Soluble

Pharmacokinetics
GI absorption: High
BBB permeant: No
P-gp substrate: Yes
CYP1A2 inhibitor: No
CYP2C19 inhibitor: No
CYP2C9 inhibitor: No
CYP2D6 inhibitor: Yes
CYP3A4 inhibitor: No
Log K_p (skin permeation): -7.20 cm/s

Druglikeness
Lipinski: Yes; 0 violation
Ghose: Yes
Veber: Yes
Egan: Yes
Muegge: Yes
Bioavailability Score: 0.55

Medicinal Chemistry
PAINS: 0 alert
Brenk: 0 alert
Leadlikeness: Yes

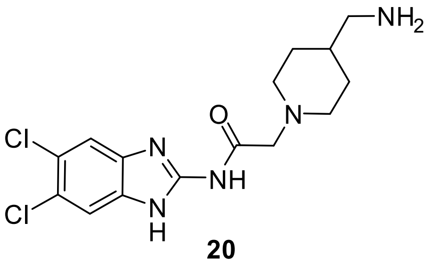

20

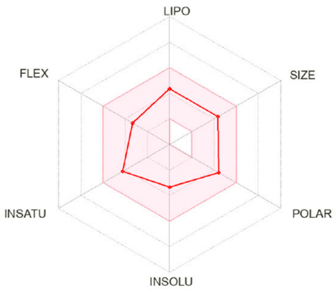

Physicochemical Properties
Formula: $C_{15}H_{19}Cl_2N_5O$
Molecular weight: 356.25 g/mol
Num. heavy atoms: 23
Num. arom. heavy atoms: 9
Fraction Csp3: 0.47
Num. rotatable bonds: 5
Num. H-bond acceptors: 4
Num. H-bond donors: 3
Molar Refractivity: 96.67
TPSA (Topological Polar SurfaceArea): 87.04 Å^2

Lipophilicity
LogP $_{o/w}$ (iLOGP): 2.00
LogP $_{o/w}$ (XLOGP3): 2.13
LogP $_{o/w}$ (WLOGP): 1.91
LogP $_{o/w}$ (MLOGP): 1.95
LogP $_{o/w}$ (SILICOS-IT): 2.49
Consensus LogP $_{o/w}$: 2.10

Water Solubility
Log S (ESOL): -3.35
Solubility: 1.59e^{-01} mg/ml; 4.46e^{-04} mol/l
Class: Soluble
Log S (Ali): -3.59
Solubility: 9.17e^{-02} mg/ml; 2.57e^{-04} mol/l
Class: Soluble
Log S (SILICOS-IT): -5.04
Solubility: 3.22e^{-03} mg/ml; 9.04e^{-06} mol/l
Class: Moderately soluble

Pharmacokinetics
GI absorption: High
BBB permeant: No
P-gp substrate: Yes
CYP1A2 inhibitor: Yes
CYP2C19 inhibitor: No
CYP2C9 inhibitor: No
CYP2D6 inhibitor: Yes
CYP3A4 inhibitor: No
Log K_p (skin permeation): -6.96 cm/s

Druglikeness
Lipinski: Yes; 0 violation
Ghose: Yes
Veber: Yes
Egan: Yes
Muegge: Yes
Bioavailability Score: 0.55

Medicinal Chemistry
PAINS: 0 alert
Brenk: 0 alert
Leadlikeness: No; 1 violation: MW>350

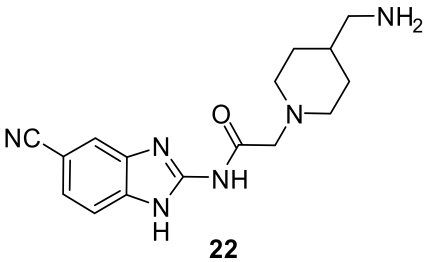

22

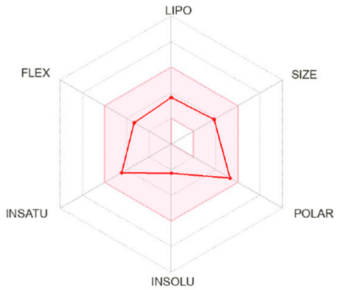

Physicochemical Properties
Formula: $C_{16}H_{20}N_6O$
Molecular weight: 312.37 g/mol
Num. heavy atoms: 23
Num. arom. heavy atoms: 9
Fraction Csp3: 0.44
Num. rotatable bonds: 5
Num. H-bond acceptors: 5
Num. H-bond donors: 3
Molar Refractivity: 91.37
TPSA (Topological Polar SurfaceArea): 110.83 Å^2

Lipophilicity
Log$P_{o/w}$ *(iLOGP)*: 1.34
Log$P_{o/w}$ *(XLOGP3)*: 0.86
Log$P_{o/w}$ *(WLOGP)*: 0.47
Log$P_{o/w}$ *(MLOGP)*: 0.30
Log$P_{o/w}$ *(SILICOS-IT)*: 1.23
Consensus Log$P_{o/w}$: 0.84

Water Solubility
Log S (ESOL): -2.28
Solubility: 1.65e^{+00} mg/ml; 5.27e^{-03} mol/l
Class: Soluble
Log S (Ali): -2.77
Solubility: 5.29e^{-01} mg/ml; 1.69e^{-03} mol/l
Class: Soluble
Log S (SILICOS-IT): -3.92
Solubility: 3.71e^{-02} mg/ml; 1.19e^{-04} mol/l
Class: Soluble

Pharmacokinetics
GI absorption: High
BBB permeant: No
P-gp substrate: Yes
CYP1A2 inhibitor: No
CYP2C19 inhibitor: No
CYP2C9 inhibitor: No
CYP2D6 inhibitor: No
CYP3A4 inhibitor: No
Log K_p *(skin permeation):* -7.59 cm/s

Druglikeness
Lipinski: Yes; 0 violation
Ghose: Yes
Veber: Yes
Egan: Yes
Muegge: Yes
Bioavailability Score: 0.55

Medicinal Chemistry
PAINS: 0 alert
Brenk: 0 alert
Leadlikeness: Yes

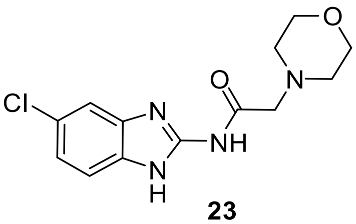

23

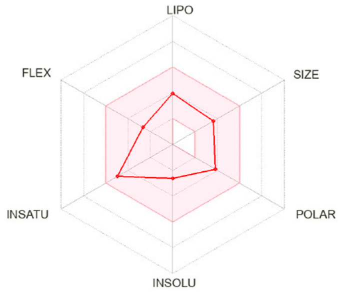

Physicochemical Properties
Formula: $C_{13}H_{15}ClN_4O_2$
Molecular weight: 294.74 g/mol
Num. heavy atoms: 20
Num. arom. heavy atoms: 9
Fraction Csp3: 0.38
Num. rotatable bonds: 4
Num. H-bond acceptors: 4
Num. H-bond donors: 2
Molar Refractivity: 80.43
TPSA (Topological Polar SurfaceArea): 70.25 Å^2

Lipophilicity
LogP $_{o/w}$ (iLOGP): 1.99
LogP $_{o/w}$ (XLOGP3): 1.43
LogP $_{o/w}$ (WLOGP): 0.92
LogP $_{o/w}$ (MLOGP): 0.94
LogP $_{o/w}$ (SILICOS-IT): 2.00
Consensus LogP $_{o/w}$: 1.45

Water Solubility
Log S (ESOL): -2.64
Solubility: 6.79e^{-01} mg/ml; 2.31e^{-03} mol/l
Class: Soluble
Log S (Ali): -2.51
Solubility: 9.10e^{-01} mg/ml; 3.09e^{-03} mol/l
Class: Soluble
Log S (SILICOS-IT): -4.11
Solubility: 2.29e^{-02} mg/ml; 7.76e^{-05} mol/l
Class: Moderately soluble

Pharmacokinetics
GI absorption: High
BBB permeant: No
P-gp substrate: Yes
CYP1A2 inhibitor: Yes
CYP2C19 inhibitor: No
CYP2C9 inhibitor: No
CYP2D6 inhibitor: No
CYP3A4 inhibitor: No
Log K_p (skin permeation): -7.08 cm/s

Druglikeness
Lipinski: Yes; 0 violation
Ghose: Yes
Veber: Yes
Egan: Yes
Muegge: Yes
Bioavailability Score: 0.55

Medicinal Chemistry
PAINS: 0 alert
Brenk: 0 alert
Leadlikeness: Yes

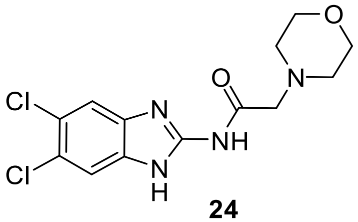

24

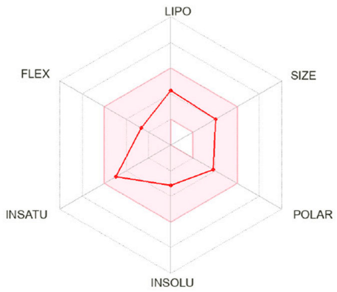

Physicochemical Properties
Formula: $C_{13}H_{14}Cl_2N_4O_2$
Molecular weight: 329.18 g/mol
Num. heavy atoms: 21
Num. arom. heavy atoms: 9
Fraction Csp3: 0.38
Num. rotatable bonds: 4
Num. H-bond acceptors: 4
Num. H-bond donors: 2
Molar Refractivity: 85.44
TPSA (Topological Polar SurfaceArea): 70.25 Å^2

Lipophilicity
LogP $_{o/w}$ (iLOGP): 2.03
LogP $_{o/w}$ (XLOGP3): 1.95
LogP $_{o/w}$ (WLOGP): 1.57
LogP $_{o/w}$ (MLOGP): 1.46
LogP $_{o/w}$ (SILICOS-IT): 2.64
Consensus LogP $_{o/w}$: 1.93

Water Solubility
Log S (ESOL): -3.16
Solubility: 2.26e^{-01} mg/ml; 6.88e^{-04} mol/l
Class: Soluble
Log S (Ali): -3.05
Solubility: 2.93e^{-01} mg/ml; 8.91e^{-04} mol/l
Class: Soluble
Log S (SILICOS-IT): -4.71
Solubility: 6.45e^{-03} mg/ml; 1.96e^{-05} mol/l
Class: Moderately soluble

Pharmacokinetics
GI absorption: High
BBB permeant: Yes
P-gp substrate: Yes
CYP1A2 inhibitor: Yes
CYP2C19 inhibitor: No
CYP2C9 inhibitor: No
CYP2D6 inhibitor: Yes
CYP3A4 inhibitor: No
Log K_p (skin permeation): -6.92 cm/s

Druglikeness
Lipinski: Yes; 0 violation
Ghose: Yes
Veber: Yes
Egan: Yes
Muegge: Yes
Bioavailability Score: 0.55

Medicinal Chemistry
PAINS: 0 alert
Brenk: 0 alert
Leadlikeness: Yes

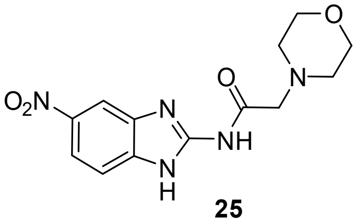

25

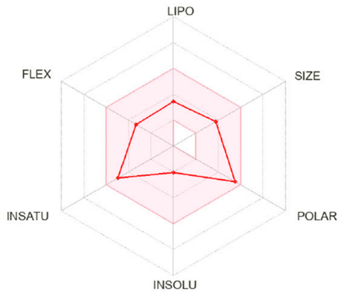

Physicochemical Properties
Formula: $C_{13}H_{15}N_5O_4$
Molecular weight: 305.29 g/mol
Num. heavy atoms: 22
Num. arom. heavy atoms: 9
Fraction Csp3: 0.38
Num. rotatable bonds: 5
Num. H-bond acceptors: 6
Num. H-bond donors: 2
Molar Refractivity: 84.24
TPSA (Topological Polar SurfaceArea): 116.07 Å^2

Lipophilicity
Log$P_{o/w}$ *(iLOGP)*: 0.35
Log$P_{o/w}$ *(XLOGP3)*: 0.52
Log$P_{o/w}$ *(WLOGP)*: 0.17
Log$P_{o/w}$ *(MLOGP)*: -0.49
Log$P_{o/w}$ *(SILICOS-IT)*: -0.80
Consensus Log$P_{o/w}$: -0.05

Water Solubility
Log S (ESOL): -2.03
Solubility: 2.83e^{+00} mg/ml; 9.27e^{-03} mol/l
Class: Soluble
Log S (Ali): -2.53
Solubility: 9.05e^{-01} mg/ml; 2.96e^{-03} mol/l
Class: Soluble
Log S (SILICOS-IT): -2.87
Solubility: 4.13e^{-01} mg/ml; 1.35e^{-03} mol/l
Class: Soluble

Pharmacokinetics
GI absorption: High
BBB permeant: No
P-gp substrate: Yes
CYP1A2 inhibitor: No
CYP2C19 inhibitor: No
CYP2C9 inhibitor: No
CYP2D6 inhibitor: No
CYP3A4 inhibitor: No
Log K_p *(skin permeation):* -7.79 cm/s

Druglikeness
Lipinski: Yes; 0 violation
Ghose: Yes
Veber: Yes
Egan: Yes
Muegge: Yes
Bioavailability Score: 0.55

Medicinal Chemistry
PAINS: 0 alert
Brenk: nitro group, oxygen-nitrogen single bond
Leadlikeness: Yes

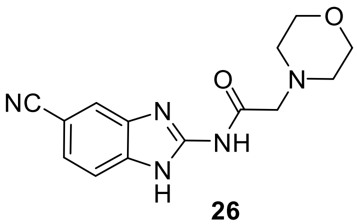

26

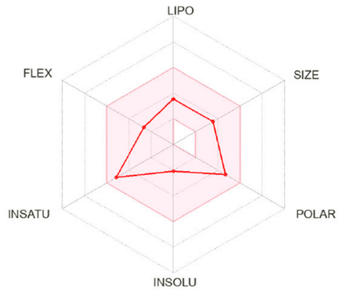

Physicochemical Properties
Formula: $C_{14}H_{15}N_5O_2$
Molecular weight: 285.30 g/mol
Num. heavy atoms: 21
Num. arom. heavy atoms: 9
Fraction Csp3: 0.36
Num. rotatable bonds: 4
Num. H-bond acceptors: 5
Num. H-bond donors: 2
Molar Refractivity: 80.13
TPSA (Topological Polar SurfaceArea): 94.04 Å^2

Lipophilicity
LogP $_{o/w}$ (iLOGP): 1.43
LogP $_{o/w}$ (XLOGP3): 0.68
LogP $_{o/w}$ (WLOGP): 0.13
LogP $_{o/w}$ (MLOGP): -0.21
LogP $_{o/w}$ (SILICOS-IT): 1.37
Consensus LogP $_{o/w}$: 0.68

Water Solubility
Log S (ESOL): -2.09
Solubility: 2.32e^{+00} mg/ml; 8.12e^{-03} mol/l
Class: Soluble
Log S (Ali): -2.23
Solubility: 1.67e^{+00} mg/ml; 5.87e^{-03} mol/l
Class: Soluble
Log S (SILICOS-IT): -3.58
Solubility: 7.44e^{-02} mg/ml; 2.61e^{-04} mol/l
Class: Soluble

Pharmacokinetics
GI absorption: High
BBB permeant: No
P-gp substrate: Yes
CYP1A2 inhibitor: Yes
CYP2C19 inhibitor: No
CYP2C9 inhibitor: No
CYP2D6 inhibitor: No
CYP3A4 inhibitor: No
Log K_p (skin permeation): -7.56 cm/s

Druglikeness
Lipinski: Yes; 0 violation
Ghose: Yes
Veber: Yes
Egan: Yes
Muegge: Yes
Bioavailability Score: 0.55

Medicinal Chemistry
PAINS: 0 alert
Brenk: 0 alert
Leadlikeness: Yes

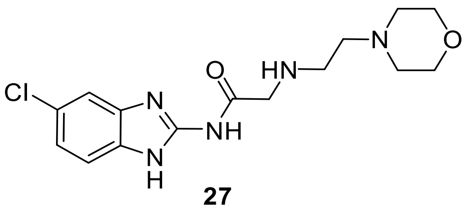

27

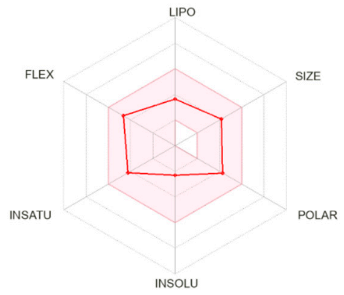

Physicochemical Properties
Formula: $C_{15}H_{20}ClN_5O_2$
Molecular weight: 237.80 g/mol
Num. heavy atoms: 23
Num. arom. heavy atoms: 9
Fraction Csp3: 0.47
Num. rotatable bonds: 7
Num. H-bond acceptors: 5
Num. H-bond donors: 3
Molar Refractivity: 92.84
TPSA (Topological Polar SurfaceArea): 82.28 Å^2

Lipophilicity
LogP $_{o/w}$ (iLOGP): 1.60
LogP $_{o/w}$ (XLOGP3): 0.89
LogP $_{o/w}$ (WLOGP): 0.51
LogP $_{o/w}$ (MLOGP): 0.64
LogP $_{o/w}$ (SILICOS-IT): 1.95
Consensus LogP $_{o/w}$: 1.12

Water Solubility
Log S (ESOL): -2.32
Solubility: 1.61e^{+00} mg/ml; 4.76e^{-03} mol/l
Class: Soluble
Log S (Ali): -2.20
Solubility: 2.12e^{+00} mg/ml; 6.27e^{-03} mol/l
Class: Soluble
Log S (SILICOS-IT): -4.95
Solubility: 3.81e^{-03} mg/ml; 1.13e^{-05} mol/l
Class: Moderately soluble

Pharmacokinetics
GI absorption: High
BBB permeant: No
P-gp substrate: Yes
CYP1A2 inhibitor: No
CYP2C19 inhibitor: No
CYP2C9 inhibitor: No
CYP2D6 inhibitor: No
CYP3A4 inhibitor: No
Log K_p (skin permeation): -7.73 cm/s

Druglikeness
Lipinski: Yes; 0 violation
Ghose: Yes
Veber: Yes
Egan: Yes
Muegge: Yes
Bioavailability Score: 0.55

Medicinal Chemistry
PAINS: 0 alert
Brenk: 0 alert
Leadlikeness: Yes

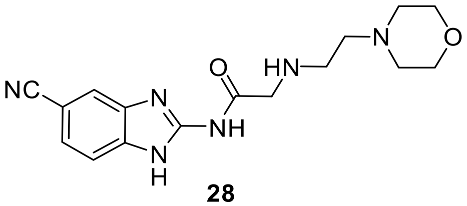

28

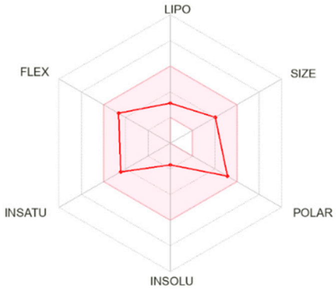

Physicochemical Properties
Formula: $C_{16}H_{20}N_6O_2$
Molecular weight: 328.37 g/mol
Num. heavy atoms: 24
Num. arom. heavy atoms: 9
Fraction Csp3: 0.44
Num. rotatable bonds: 7
Num. H-bond acceptors: 6
Num. H-bond donors: 3
Molar Refractivity: 92.55
TPSA (Topological Polar SurfaceArea): 106.07 Å^2

Lipophilicity
LogP $_{o/w}$ (iLOGP): 1.22
LogP $_{o/w}$ (XLOGP3): -0.02
LogP $_{o/w}$ (WLOGP): -0.28
LogP $_{o/w}$ (MLOGP): -0.49
LogP $_{o/w}$ (SILICOS-IT): 1.34
Consensus LogP $_{o/w}$: 0.35

Water Solubility
Log S (ESOL): -1.68
Solubility: 6.88e^{+00} mg/ml; 2.10e^{-02} mol/l
Class: Very soluble
Log S (Ali): -1.76
Solubility: 5.73e^{+00} mg/ml; 1.75e^{-02} mol/l
Class: Very soluble
Log S (SILICOS-IT): -4.42
Solubility: 1.24e^{-02} mg/ml; 3.77e^{-05} mol/l
Class: Moderately soluble

Pharmacokinetics
GI absorption: High
BBB permeant: No
P-gp substrate: Yes
CYP1A2 inhibitor: No
CYP2C19 inhibitor: No
CYP2C9 inhibitor: No
CYP2D6 inhibitor: No
CYP3A4 inhibitor: No
Log K_p (skin permeation): -8.32 cm/s

Druglikeness
Lipinski: Yes; 0 violation
Ghose: Yes
Veber: Yes
Egan: Yes
Muegge: Yes
Bioavailability Score: 0.55

Medicinal Chemistry
PAINS: 0 alert
Brenk: 0 alert
Leadlikeness: Yes

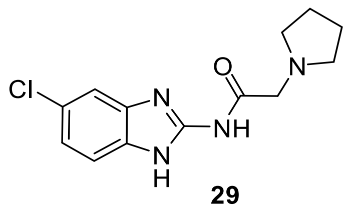

29

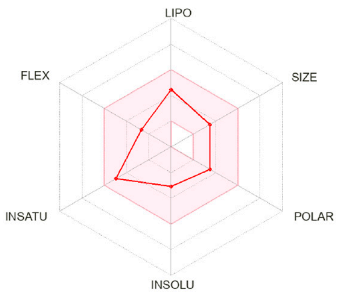

Physicochemical Properties
Formula: $C_{13}H_{15}ClN_4O$
Molecular weight: 278.74 g/mol
Num. heavy atoms: 19
Num. arom. heavy atoms: 9
Fraction Csp3: 0.38
Num. rotatable bonds: 4
Num. H-bond acceptors: 3
Num. H-bond donors: 2
Molar Refractivity: 79.34
TPSA (Topological Polar SurfaceArea): 61.02 Å^2

Lipophilicity
LogP $_{o/w}$ (iLOGP): 1.67
LogP $_{o/w}$ (XLOGP3): 2.29
LogP $_{o/w}$ (WLOGP): 1.68
LogP $_{o/w}$ (MLOGP): 1.75
LogP $_{o/w}$ (SILICOS-IT): 2.38
Consensus LogP $_{o/w}$: 1.95

Water Solubility
Log S (ESOL): -3.10
Solubility: 2.23e^{-01} mg/ml; 7.99e^{-04} mol/l
Class: Soluble
Log S (Ali): -3.21
Solubility: 1.72e^{-01} mg/ml ; 6.18e^{-04} mol/l
Class: Soluble
Log S (SILICOS-IT): -4.38
Solubility: 1.18e^{-02} mg/ml ; 4.22e^{-05} mol/l
Class: Moderately soluble

Pharmacokinetics
GI absorption: High
BBB permeant: Yes
P-gp substrate: Yes
CYP1A2 inhibitor: Yes
CYP2C19 inhibitor: No
CYP2C9 inhibitor: No
CYP2D6 inhibitor: Yes
CYP3A4 inhibitor: No
Log K_p (skin permeation): -6.22 cm/s

Druglikeness
Lipinski: Yes; 0 violation
Ghose: Yes
Veber: Yes
Egan: Yes
Muegge: Yes
Bioavailability Score: 0.55

Medicinal Chemistry
PAINS: 0 alert
Brenk: 0 alert
Leadlikeness: Yes

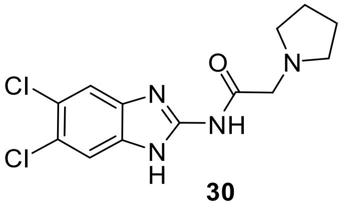

30

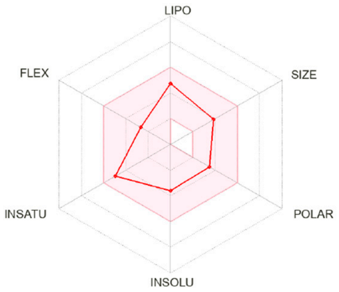

Physicochemical Properties
Formula: $C_{13}H_{14}Cl_2N_4O$
Molecular weight: 313.18 g/mol
Num. heavy atoms: 20
Num. arom. heavy atoms: 9
Fraction Csp3: 0.38
Num. rotatable bonds: 4
Num. H-bond acceptors: 3
Num. H-bond donors: 2
Molar Refractivity: 84.35
TPSA (Topological Polar SurfaceArea): 61.02 Å²

Lipophilicity
LogP $_{o/w}$ (iLOGP): 2.10
LogP $_{o/w}$ (XLOGP3): 2.81
LogP $_{o/w}$ (WLOGP): 2.33
LogP $_{o/w}$ (MLOGP): 2.27
LogP $_{o/w}$ (SILICOS-IT): 3.02
Consensus LogP $_{o/w}$: 2.51

Water Solubility
Log S (ESOL): -3.62
Solubility: 7.49e^{-02} mg/ml; 2.39e^{-04} mol/l
Class: Soluble
Log S (Ali): -3.75
Solubility: 5.59e^{-02} mg/ml; 1.78e^{-04} mol/l
Class: Soluble
Log S (SILICOS-IT): -4.98
Solubility: 3.31e^{-03} mg/ml; 1.06e^{-05} mol/l
Class: Moderately soluble

Pharmacokinetics
GI absorption: High
BBB permeant: Yes
P-gp substrate: Yes
CYP1A2 inhibitor: Yes
CYP2C19 inhibitor: No
CYP2C9 inhibitor: No
CYP2D6 inhibitor: Yes
CYP3A4 inhibitor: No
Log K_p (skin permeation): -6.37 cm/s

Druglikeness
Lipinski: Yes; 0 violation
Ghose: Yes
Veber: Yes
Egan: Yes
Muegge: Yes
Bioavailability Score: 0.55

Medicinal Chemistry
PAINS: 0 alert
Brenk: 0 alert
Leadlikeness: Yes

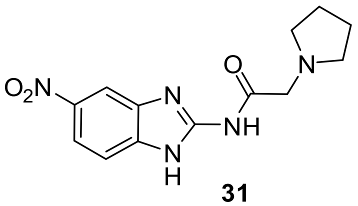

31

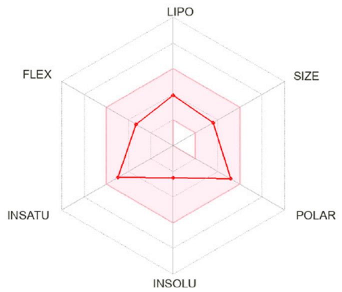

Physicochemical Properties
Formula: $C_{13}H_{15}N_5O_3$
Molecular weight: 289.29 g/mol
Num. heavy atoms: 21
Num. arom. heavy atoms: 9
Fraction Csp3: 0.38
Num. rotatable bonds: 5
Num. H-bond acceptors: 5
Num. H-bond donors: 2
Molar Refractivity: 83.15
TPSA (Topological Polar SurfaceArea): 106.84 Å^2

Lipophilicity
LogP $_{o/w}$ (iLOGP): 0.34
LogP $_{o/w}$ (XLOGP3): 1.38
LogP $_{o/w}$ (WLOGP): 0.93
LogP $_{o/w}$ (MLOGP): 0.29
LogP $_{o/w}$ (SILICOS-IT): -0.43
Consensus LogP $_{o/w}$: 0.50

Water Solubility
Log S (ESOL): -2.49
Solubility: 9.36e^{-01} mg/ml; 3.23e^{-03} mol/l
Class: Soluble
Log S (Ali): -3.23
Solubility: 1.72e^{-01} mg/ml; 5.93e^{-04} mol/l
Class: Soluble
Log S (SILICOS-IT): -3.14
Solubility: 2.12e^{-01} mg/ml; 7.31e^{-04} mol/l
Class: Soluble

Pharmacokinetics
GI absorption: High
BBB permeant: No
P-gp substrate: Yes
CYP1A2 inhibitor: No
CYP2C19 inhibitor: No
CYP2C9 inhibitor: No
CYP2D6 inhibitor: Yes
CYP3A4 inhibitor: No
Log K_p (skin permeation): -7.08 cm/s

Druglikeness
Lipinski: Yes; 0 violation
Ghose: Yes
Veber: Yes
Egan: Yes
Muegge: Yes
Bioavailability Score: 0.55

Medicinal Chemistry
PAINS: 0 alert
Brenk: nitro group, oxygen-nitrogen single bond
Leadlikeness: Yes

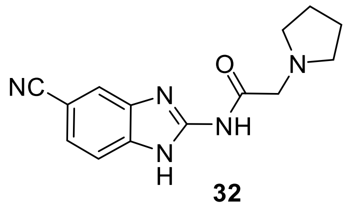

32

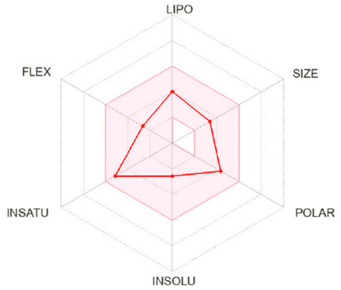

Physicochemical Properties
Formula: $C_{14}H_{15}N_5O$
Molecular weight: 269.30 g/mol
Num. heavy atoms: 20
Num. arom. heavy atoms: 9
Fraction Csp3: 0.36
Num. rotatable bonds: 4
Num. H-bond acceptors: 4
Num. H-bond donors: 2
Molar Refractivity: 79.05
TPSA (Topological Polar SurfaceArea): 84.81 Å^2

Lipophilicity
LogP $_{o/w}$ (iLOGP): 0.59
LogP $_{o/w}$ (XLOGP3): 1.54
LogP $_{o/w}$ (WLOGP): 0.90
LogP $_{o/w}$ (MLOGP): 0.59
LogP $_{o/w}$ (SILICOS-IT): 1.75
Consensus LogP $_{o/w}$: 1.07

Water Solubility
Log S (ESOL): -2.55
Solubility: 7.61e^{-01} mg/ml; 2.83e^{-03} mol/l
Class: Soluble
Log S (Ali): -2.93
Solubility: 3.16e^{-01} mg/ml; 1.17e^{-03} mol/l
Class: Soluble
Log S (SILICOS-IT): -3.85
Solubility: 3.81e^{-02} mg/ml; 1.42e^{-04} mol/l
Class: Soluble

Pharmacokinetics
GI absorption: High
BBB permeant: No
P-gp substrate: Yes
CYP1A2 inhibitor: Yes
CYP2C19 inhibitor: No
CYP2C9 inhibitor: No
CYP2D6 inhibitor: Yes
CYP3A4 inhibitor: No
Log K_p (skin permeation): -6.85 cm/s

Druglikeness
Lipinski: Yes; 0 violation
Ghose: Yes
Veber: Yes
Egan: Yes
Muegge: Yes
Bioavailability Score: 0.55

Medicinal Chemistry
PAINS: 0 alert
Brenk: 0 alert
Leadlikeness: Yes

^{1}H-NMR and ^{13}C-APT NMR scanned spectra of compounds **1-32**, all recorded in DMSO-d$_6$.

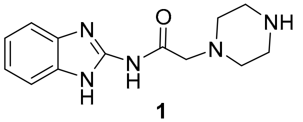

1

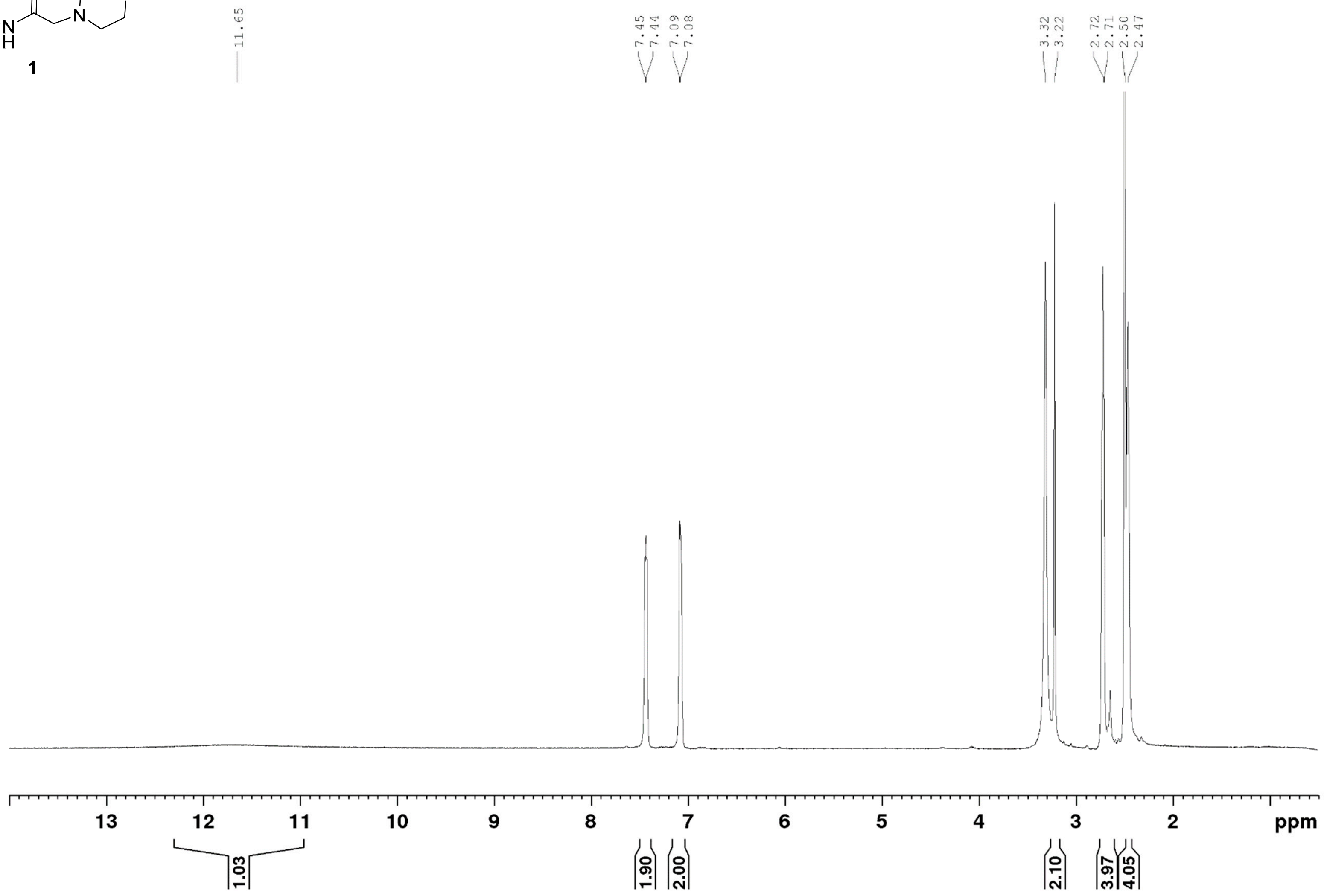

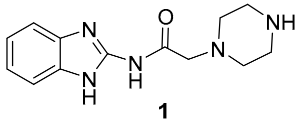
N
NH
O
N
H
N
H

1

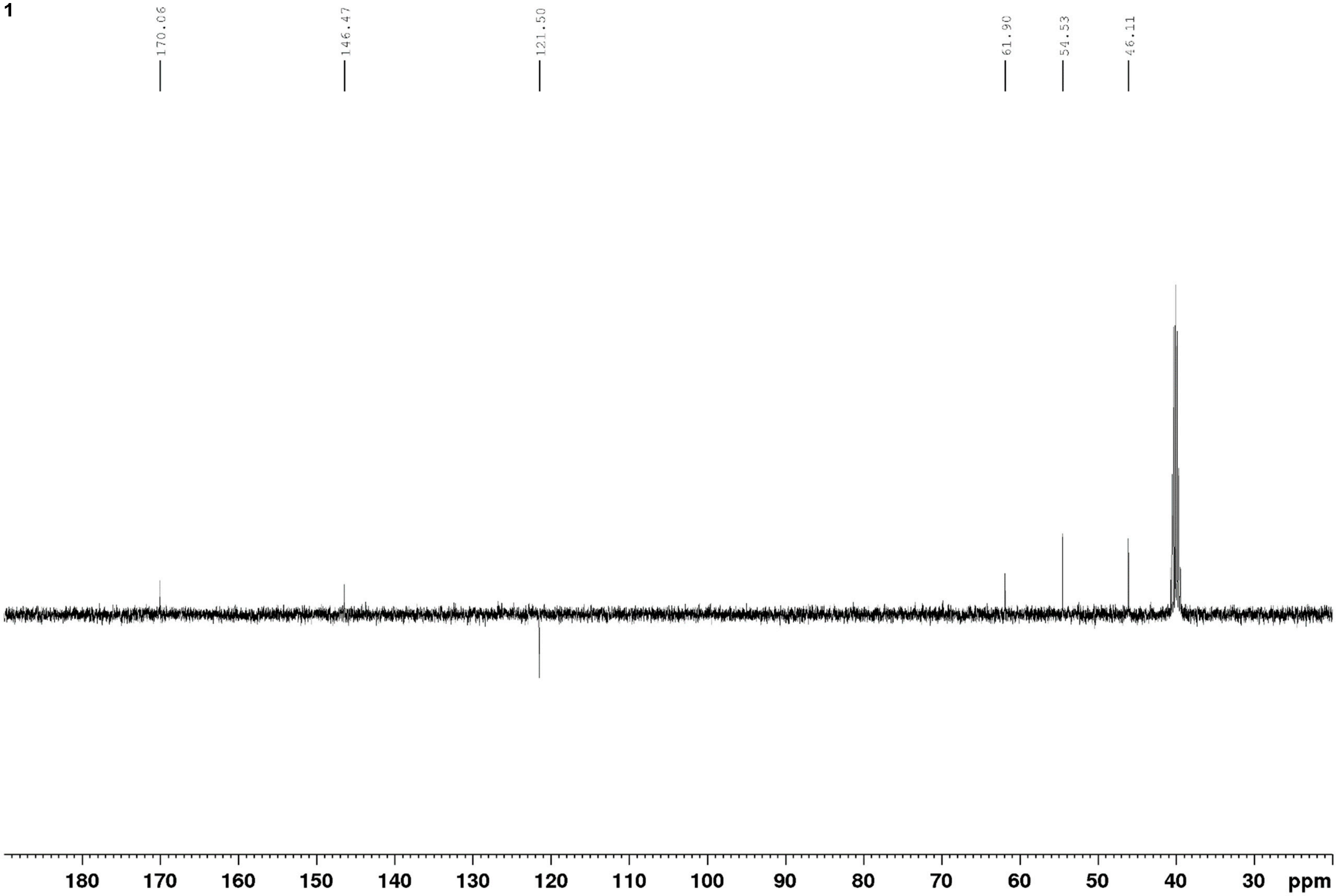
170.06
146.47
121.50
61.90
54.53
46.11
180
170
160
150
140
130
120
110
100
90
80
70
60
50
40
30
ppm

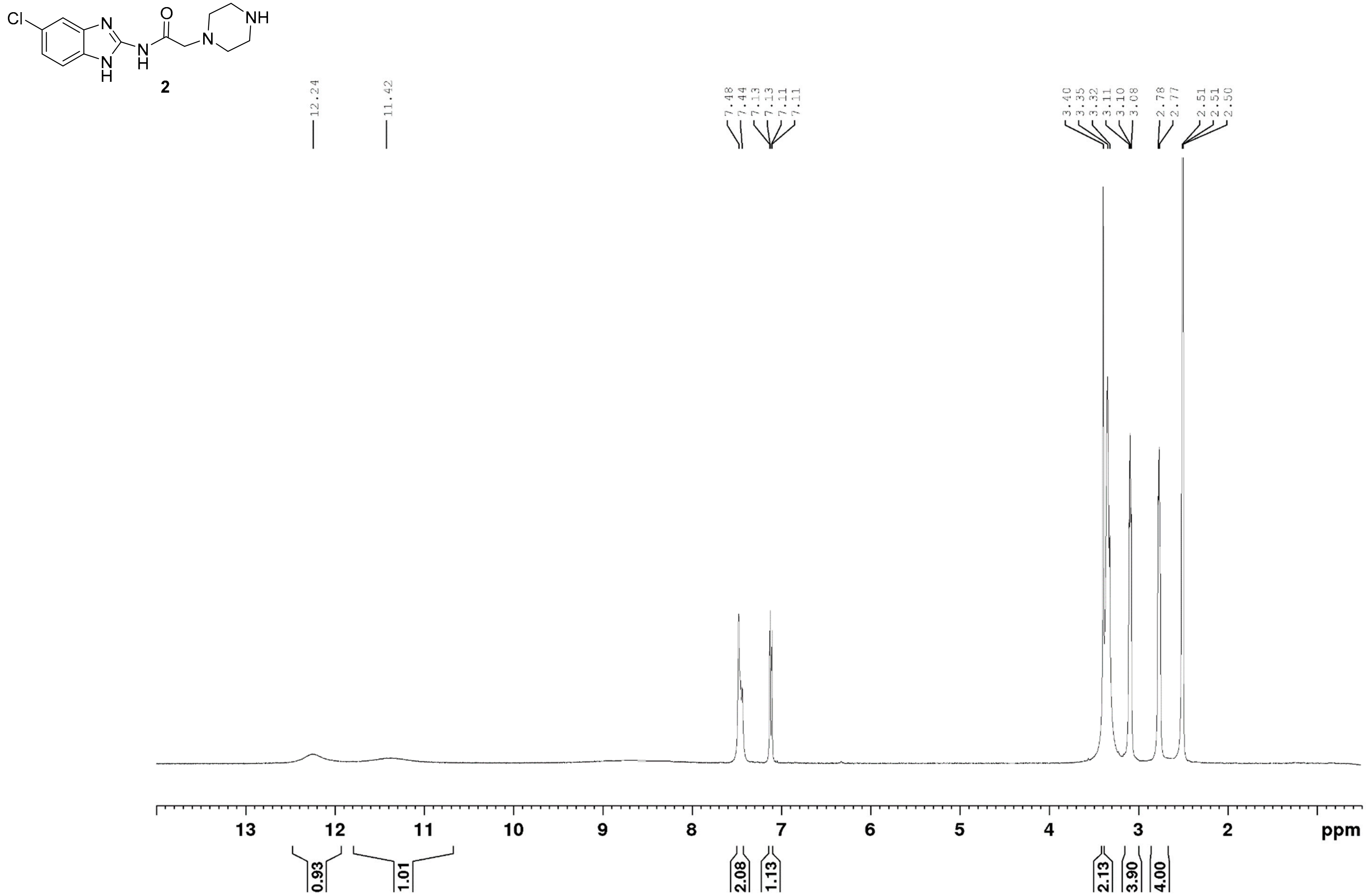
Cl
N
N
H
N
H
O
N
NH
2
12.24
11.42
7.48
7.44
7.13
7.13
7.11
7.11
3.40
3.35
3.32
3.11
3.10
3.08
2.78
2.77
2.51
2.51
2.50
13
12
11
10
9
8
7
6
5
4
3
2
ppm
0.93
1.01
2.08
1.13
2.13
3.90
4.00

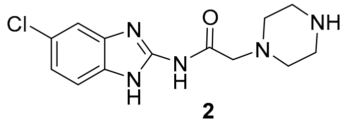

2

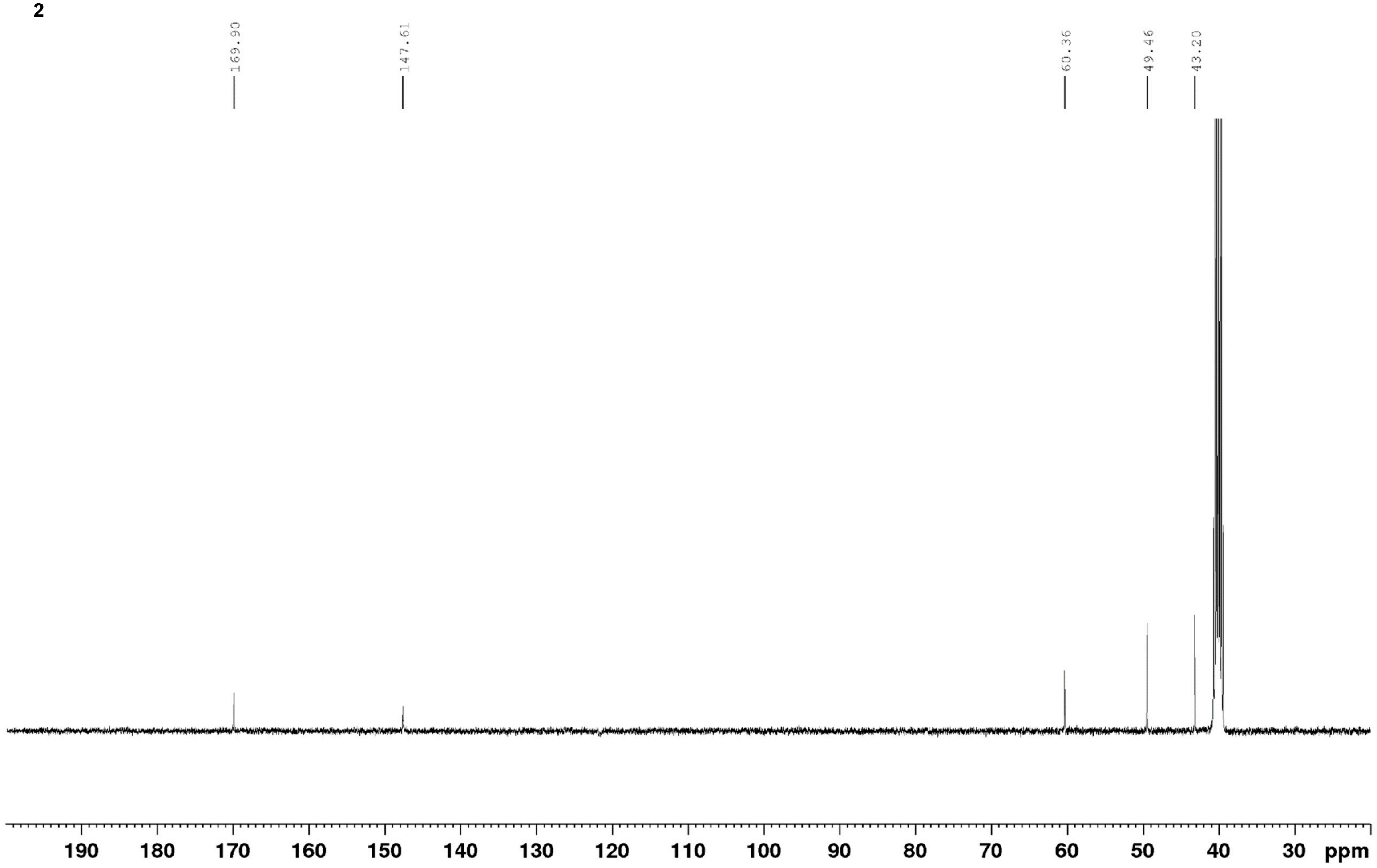

169.90
147.61
60.36
49.46
43.20
190
180
170
160
150
140
130
120
110
100
90
80
70
60
50
40
30
ppm

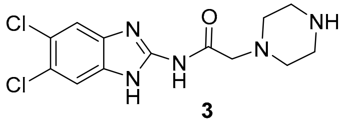

3

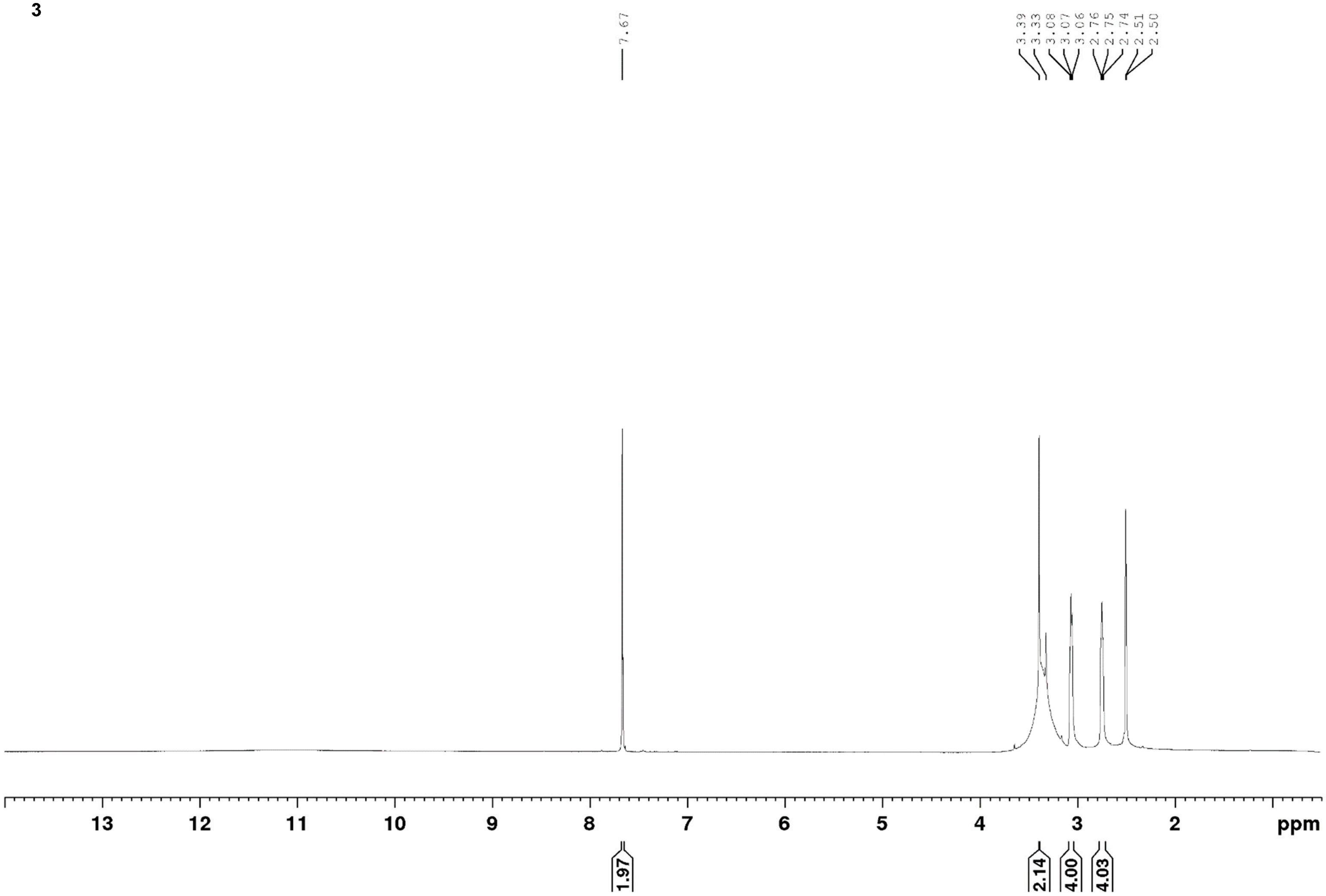
7.67
3.39
3.33
3.08
3.07
3.06
2.76
2.75
2.74
2.51
2.50
13
12
11
10
9
8
7
6
5
4
3
2
ppm
1.97
2.14
4.00
4.03

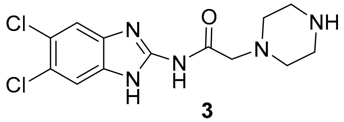

3

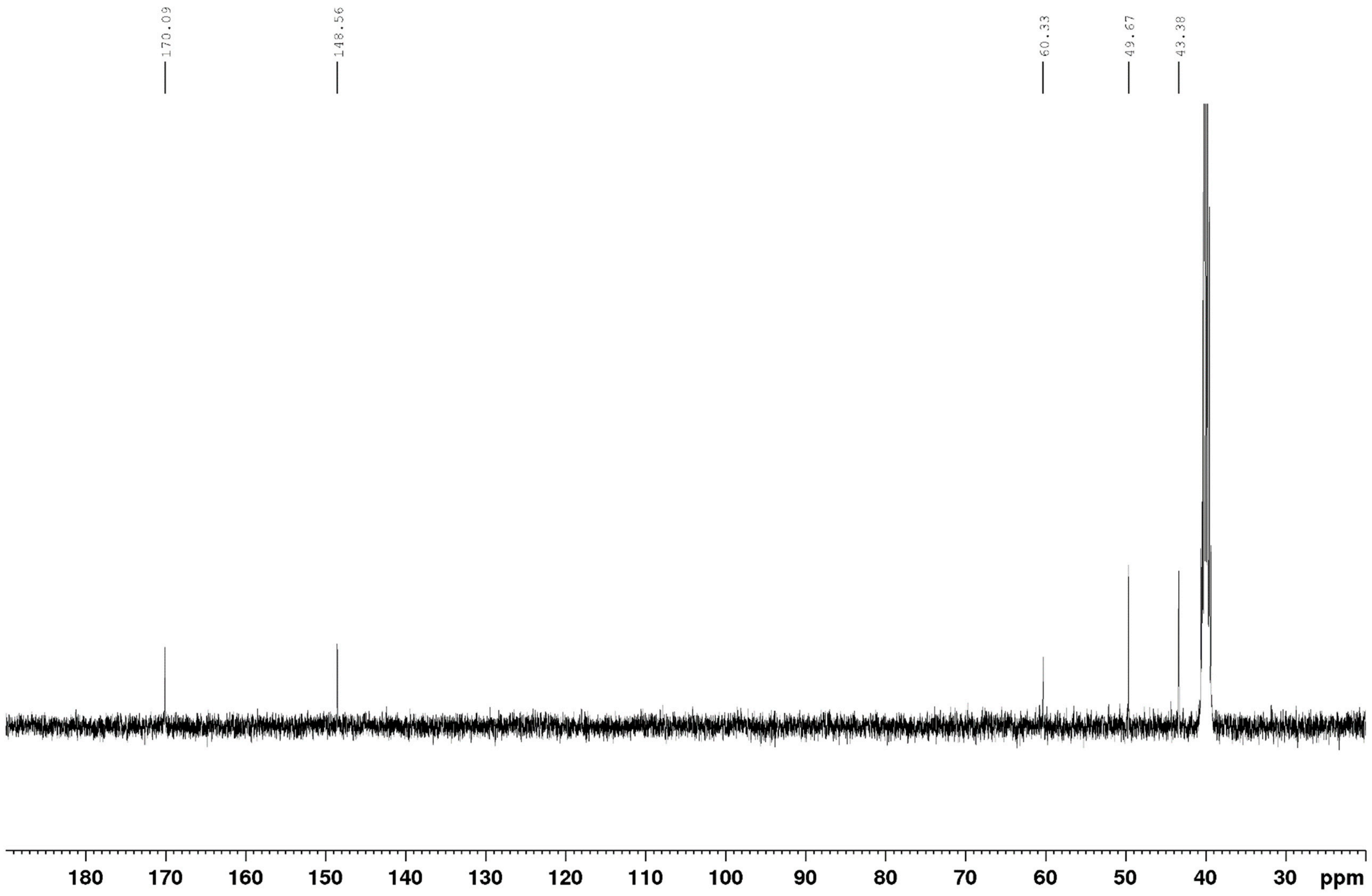
170.09
148.56
60.33
49.67
43.38
180
170
160
150
140
130
120
110
100
90
80
70
60
50
40
30
ppm

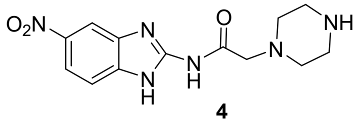
O2N
N
N
H
N
H
O
N
NH

4

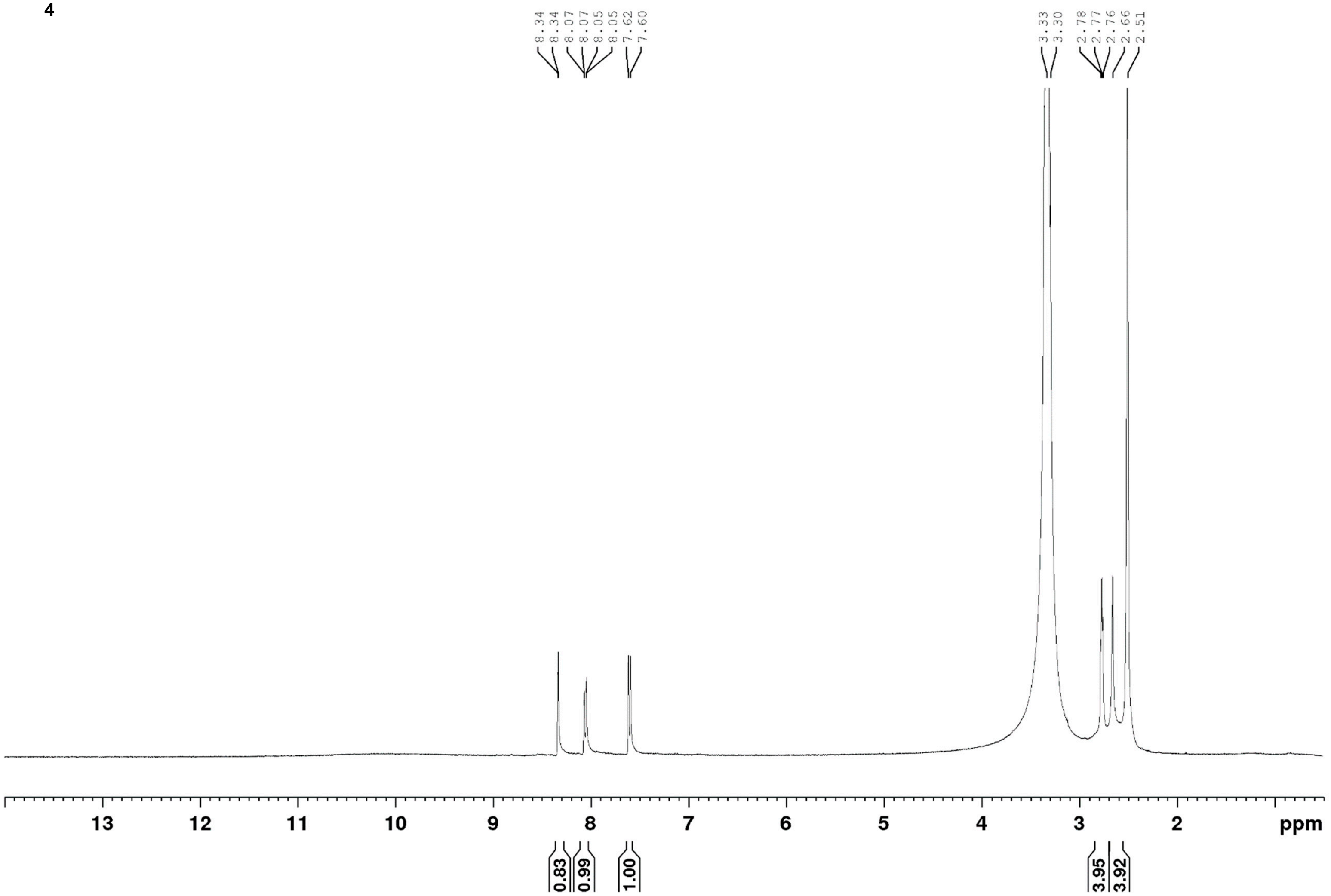
8.34
8.34
8.07
8.07
8.05
8.05
7.62
7.60
3.33
3.30
2.78
2.77
2.76
2.66
2.51
13
12
11
10
9
8
7
6
5
4
3
2
ppm
0.83
0.99
1.00
3.95
3.92

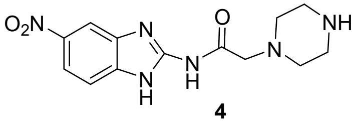

4

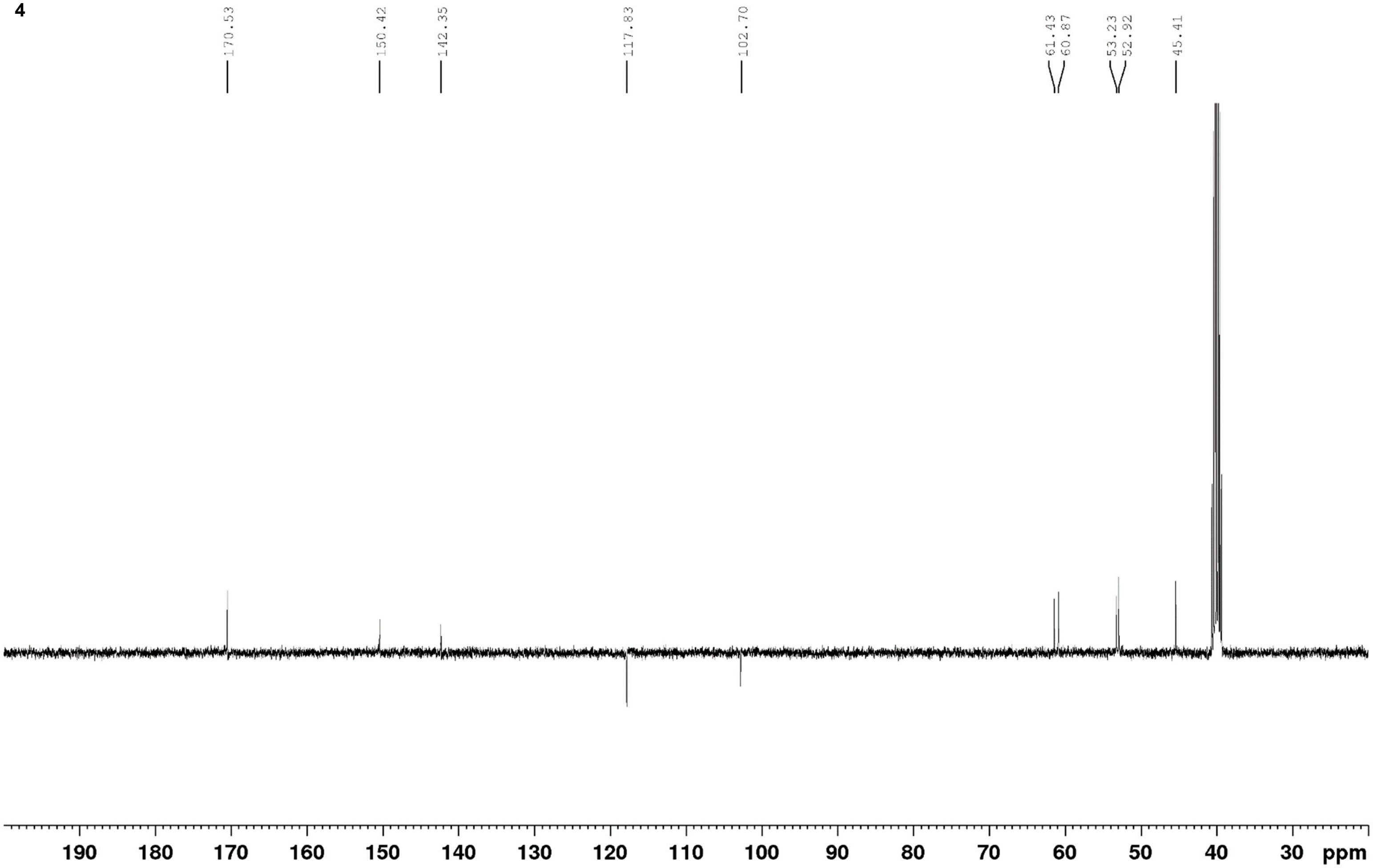
170.53
150.42
142.35
117.83
102.70
61.43
60.87
53.23
52.92
45.41
190
180
170
160
150
140
130
120
110
100
90
80
70
60
50
40
30
ppm

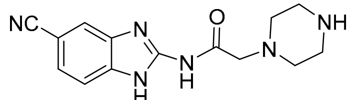
NC
N
N
H
N
H
O
N
NH

5

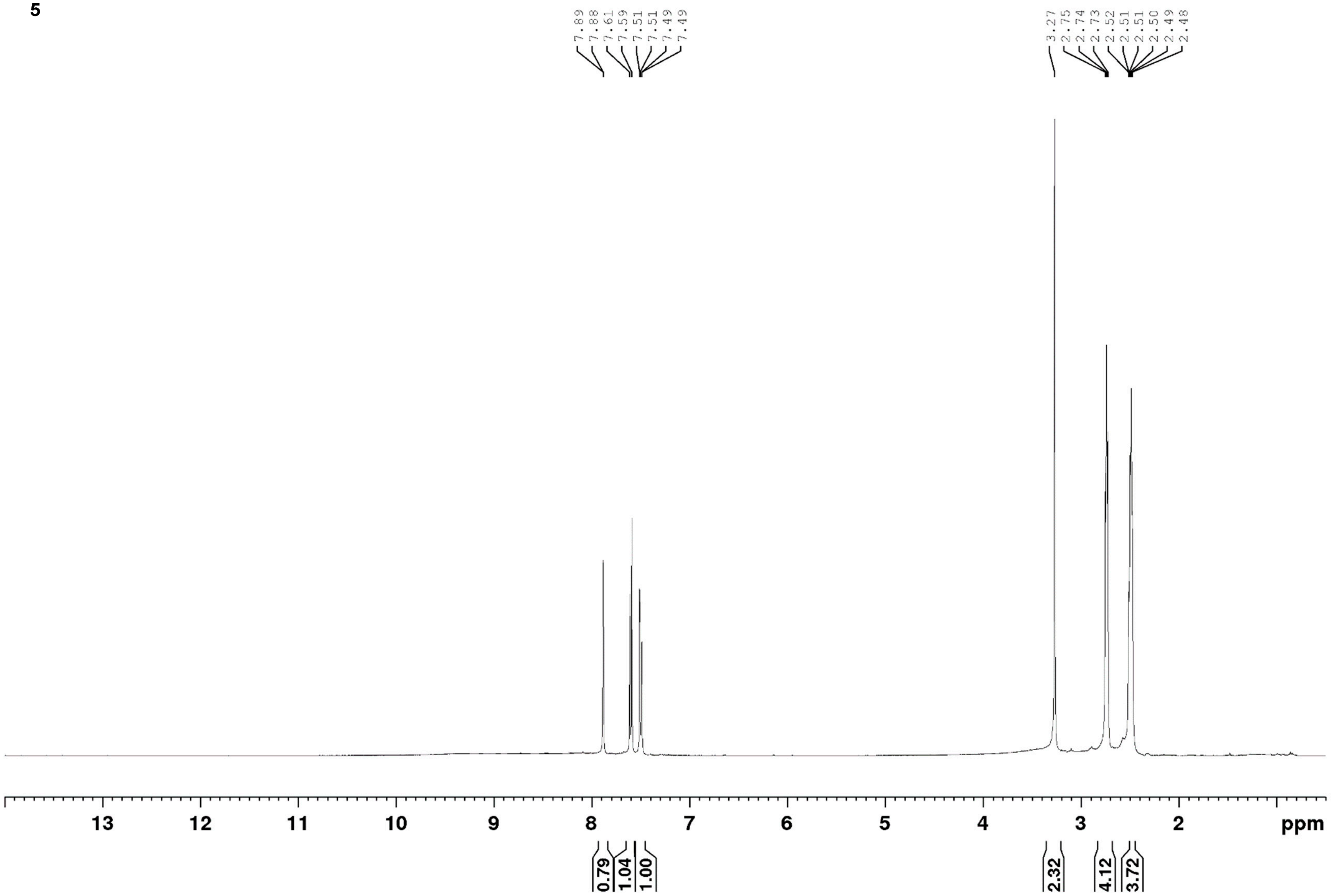
7.89
7.88
7.61
7.59
7.51
7.51
7.49
7.49
3.27
2.75
2.74
2.73
2.52
2.51
2.51
2.50
2.49
2.48
13
12
11
10
9
8
7
6
5
4
3
2
ppm
0.79
1.04
1.00
2.32
4.12
3.72

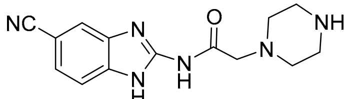
NC
N
N
H
N
H
O
N
NH

5

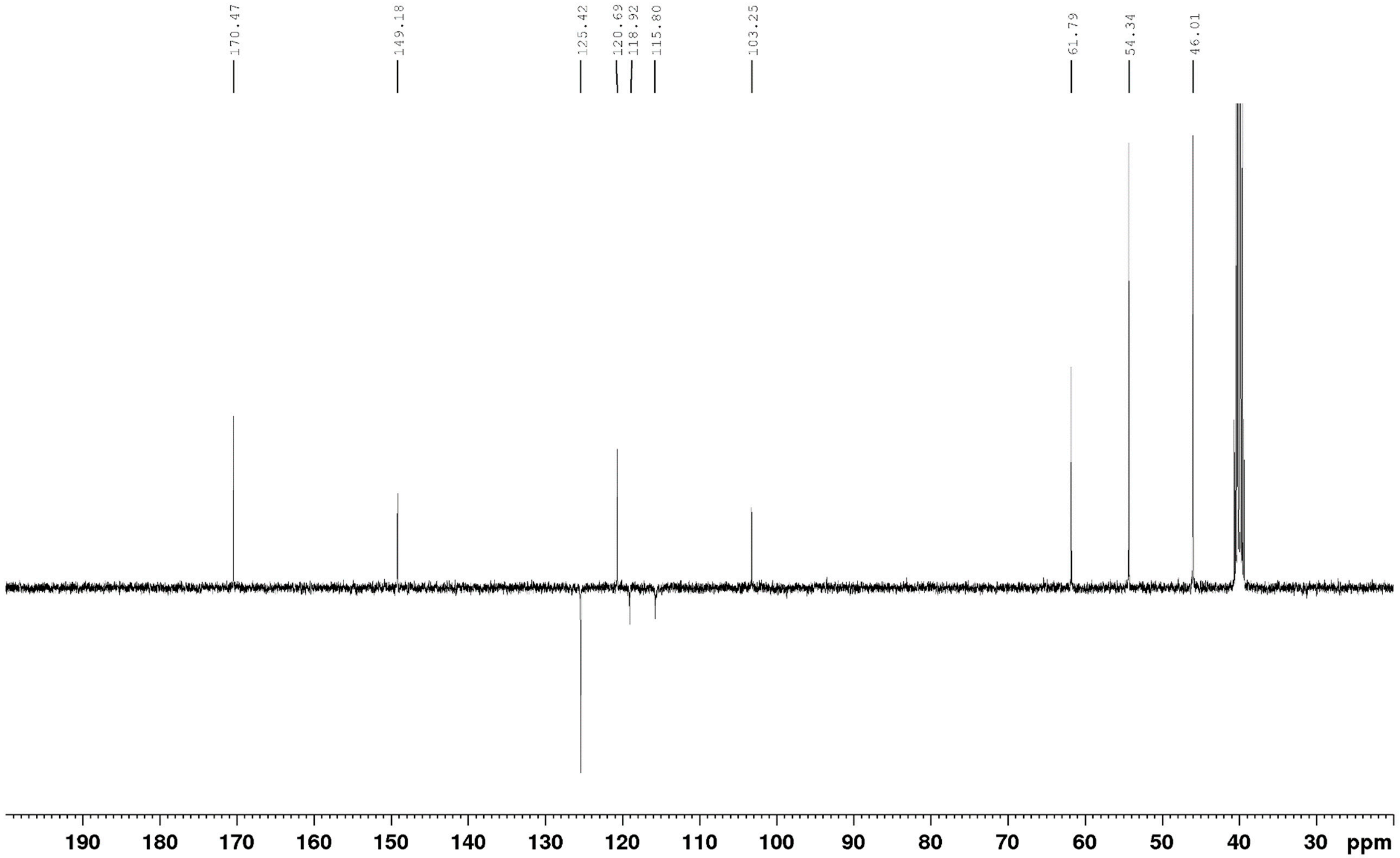
170.47
149.18
125.42
120.69
118.92
115.80
103.25
61.79
54.34
46.01
190
180
170
160
150
140
130
120
110
100
90
80
70
60
50
40
30
ppm

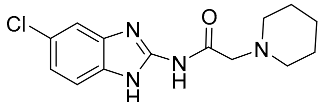
Cl
N
O
N
H
N
N
H

6

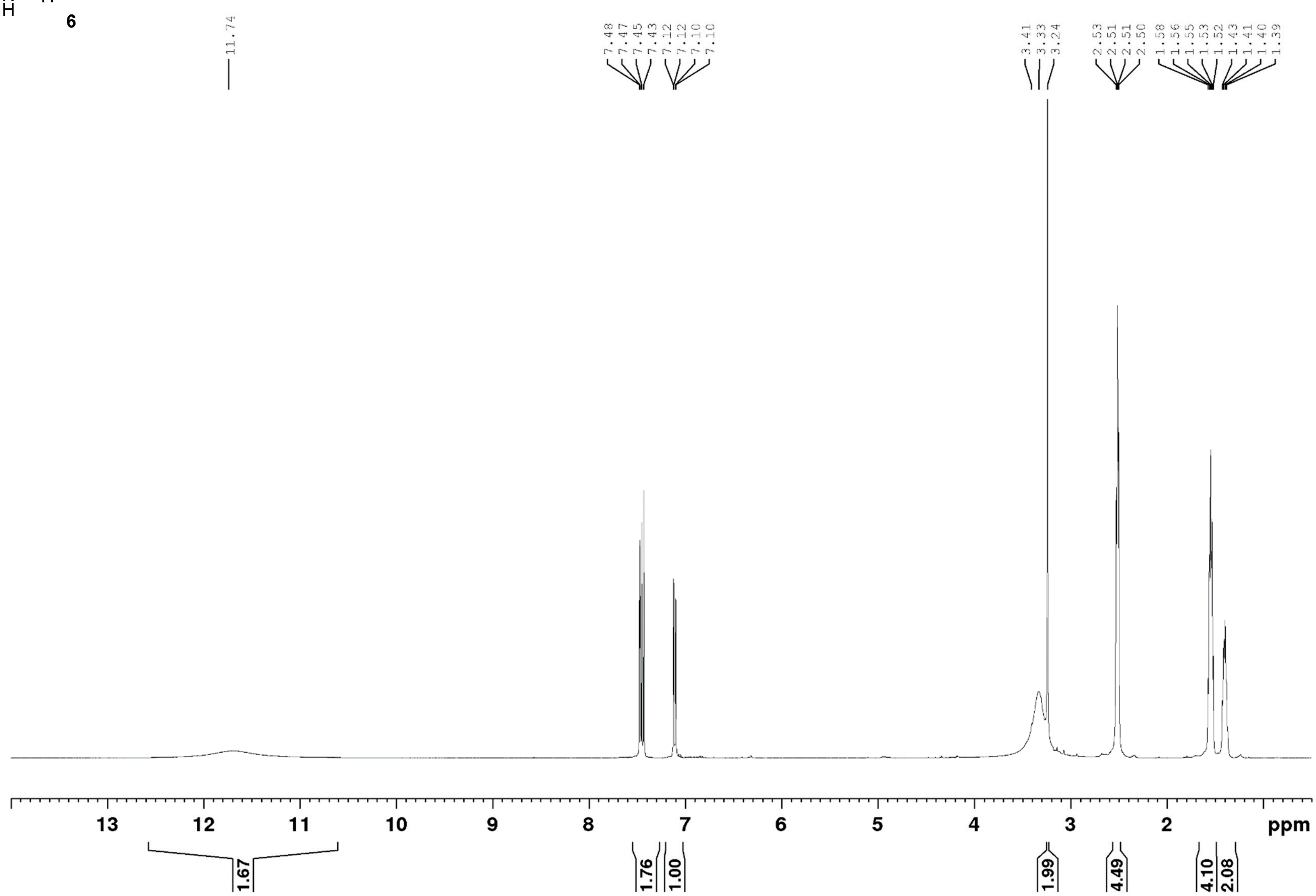
11.74
7.48
7.47
7.45
7.43
7.12
7.12
7.10
7.10
3.41
3.33
3.24
2.53
2.51
2.51
2.50
1.58
1.56
1.55
1.53
1.52
1.43
1.41
1.40
1.39
13
12
11
10
9
8
7
6
5
4
3
2
ppm
1.67
1.76
1.00
1.99
4.49
4.10
2.08

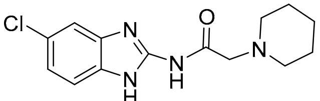

6

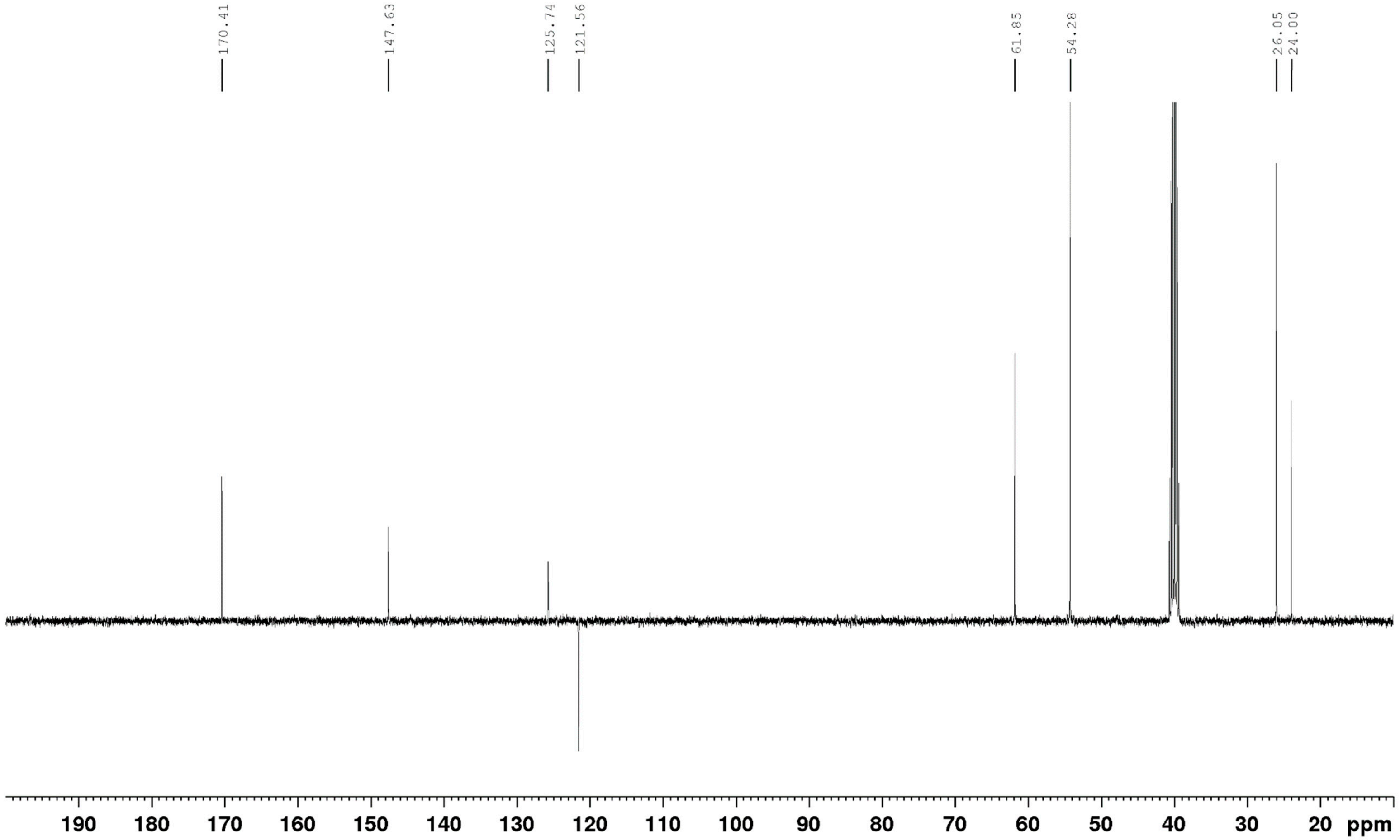
170.41
147.63
125.74
121.56
61.85
54.28
26.05
24.00
190
180
170
160
150
140
130
120
110
100
90
80
70
60
50
40
30
20
ppm

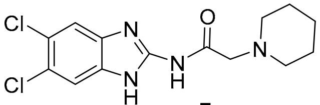
Cl
Cl
N
N
H
O
N
H
N

7

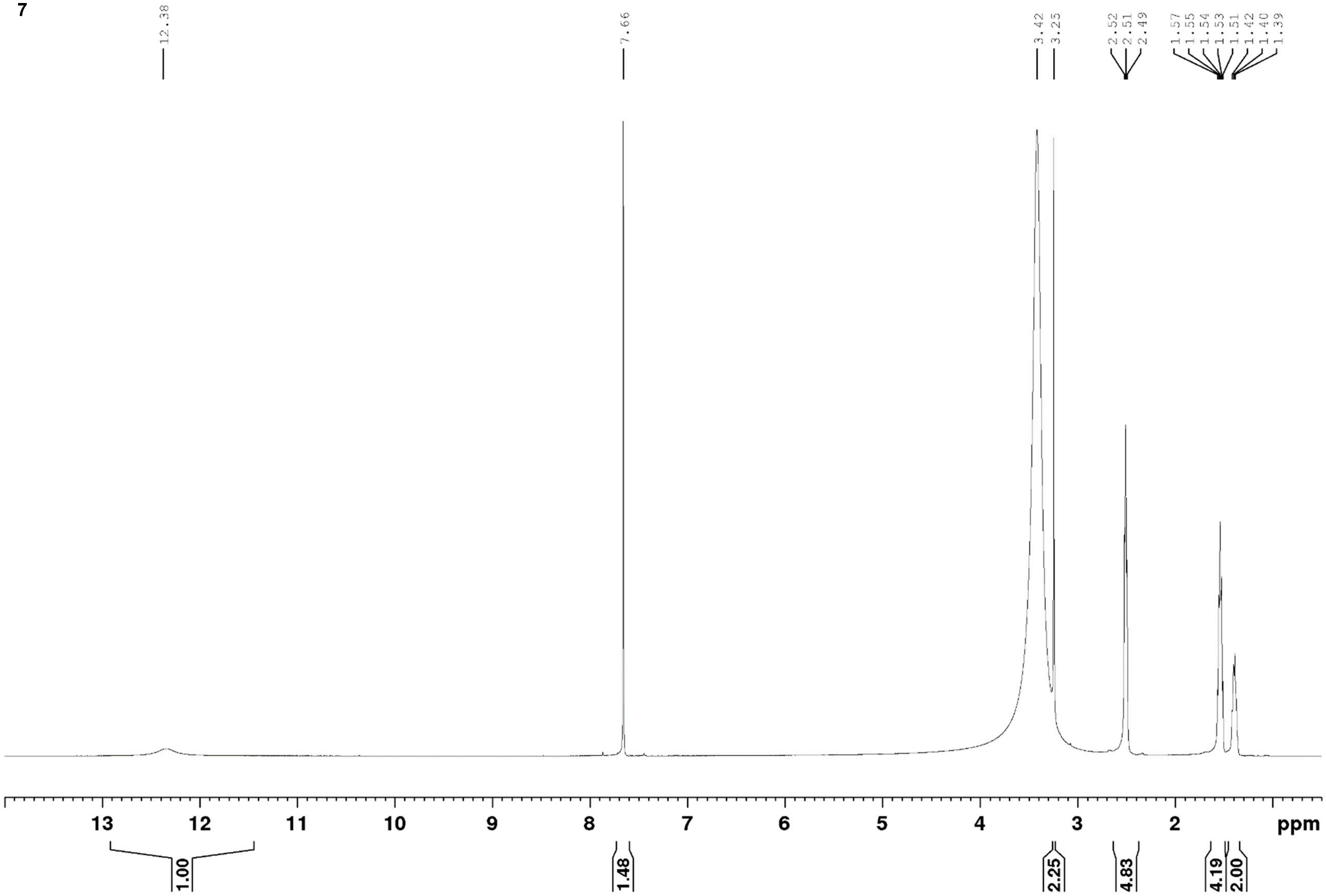
12.38
7.66
3.42
3.25
2.52
2.51
2.49
1.57
1.55
1.54
1.53
1.51
1.42
1.40
1.39
13
12
11
10
9
8
7
6
5
4
3
2
ppm
1.00
1.48
2.25
4.83
4.19
2.00

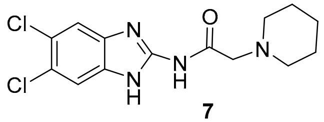

7

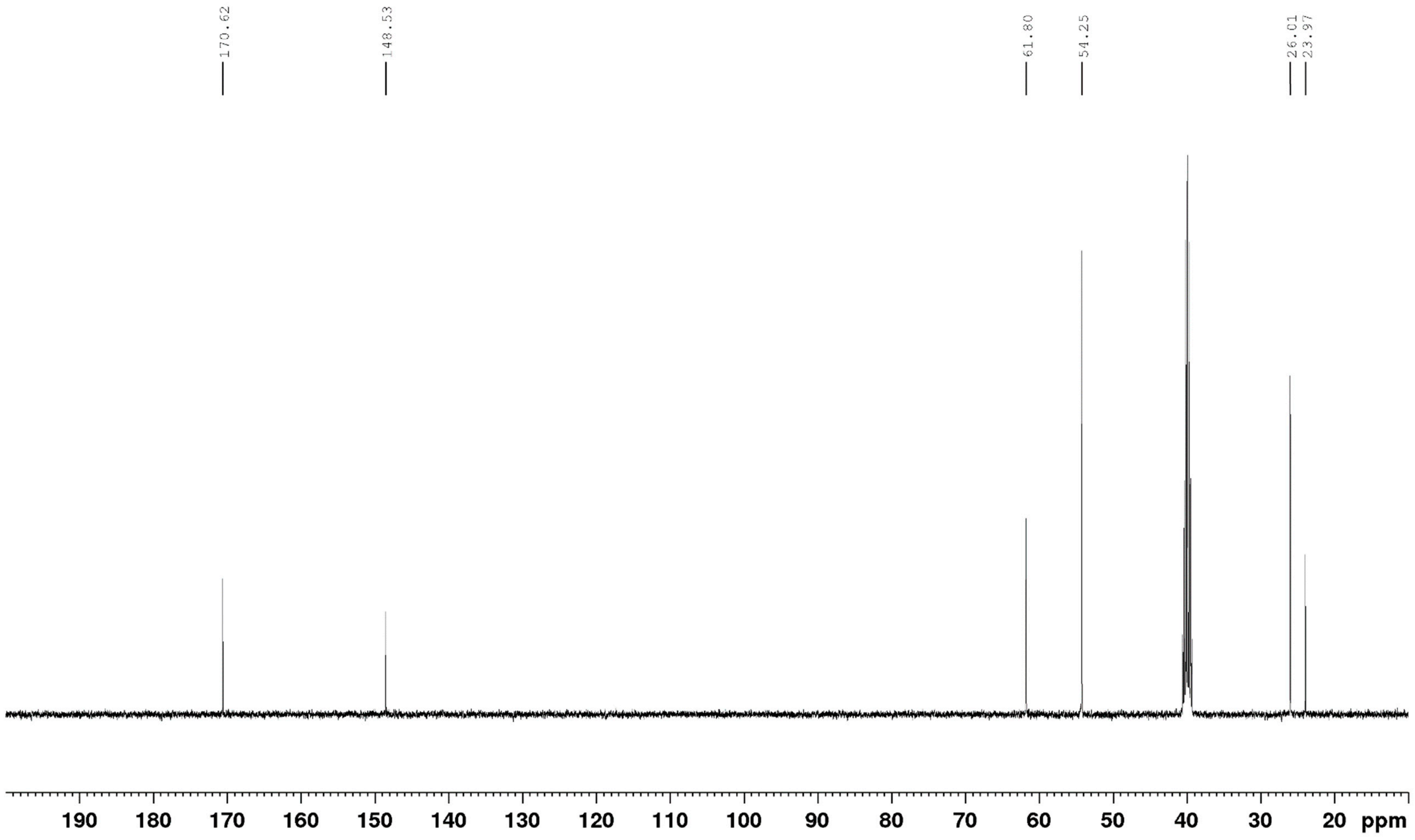

170.62
148.53
61.80
54.25
26.01
23.97
190 180 170 160 150 140 130 120 110 100 90 80 70 60 50 40 30 20 ppm

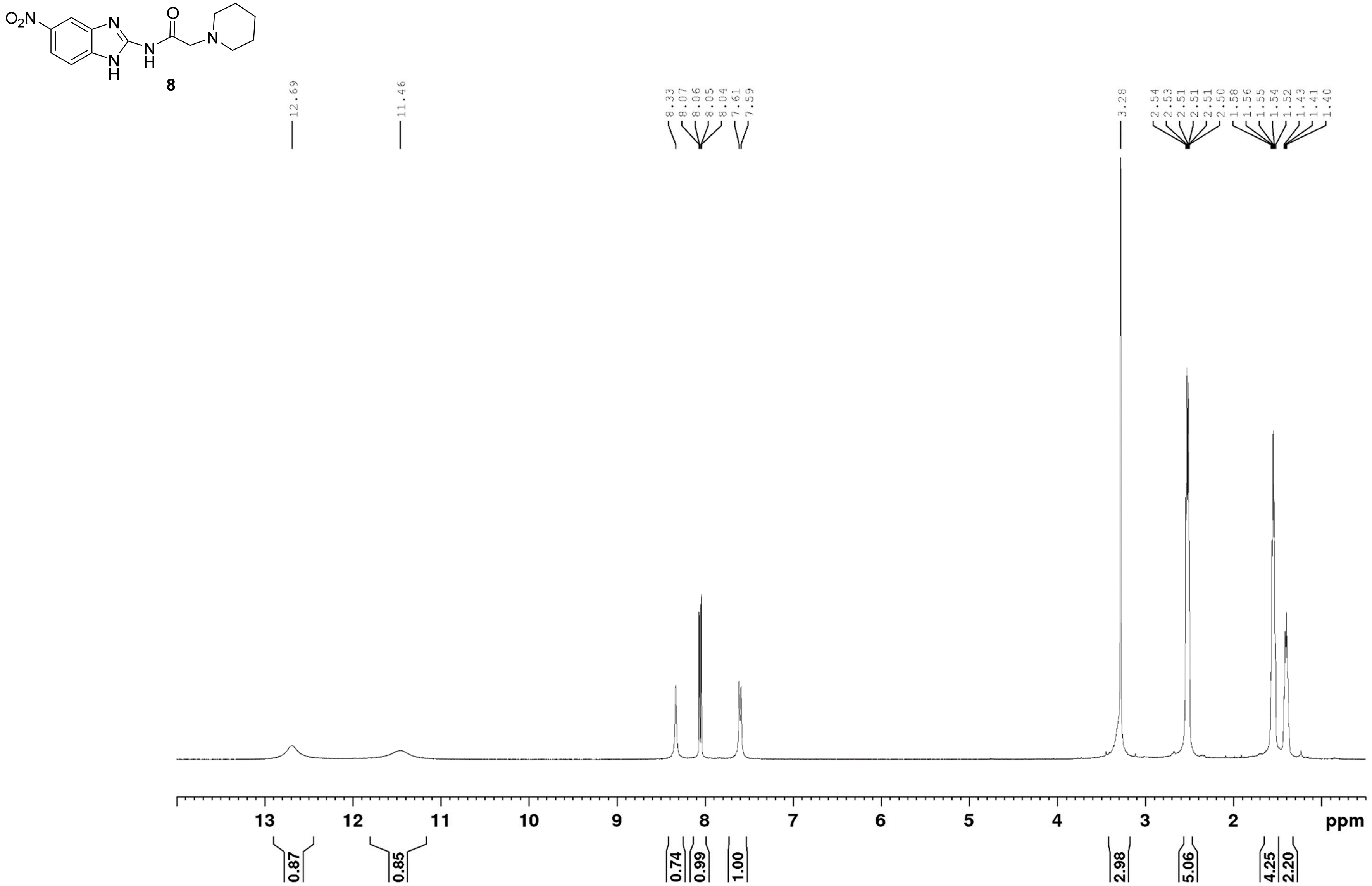

O2N
N
O
N
H
N
N
H
8
12.69
11.46
8.33
8.07
8.06
8.05
8.04
7.61
7.59
3.28
2.54
2.53
2.51
2.51
2.51
2.50
1.58
1.56
1.55
1.54
1.52
1.43
1.41
1.40
13
12
11
10
9
8
7
6
5
4
3
2
ppm
0.87
0.85
0.74
0.99
1.00
2.98
5.06
4.25
2.20

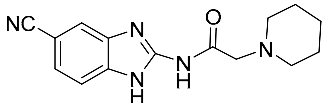
NC
N
N
H
O
N
H
N

9

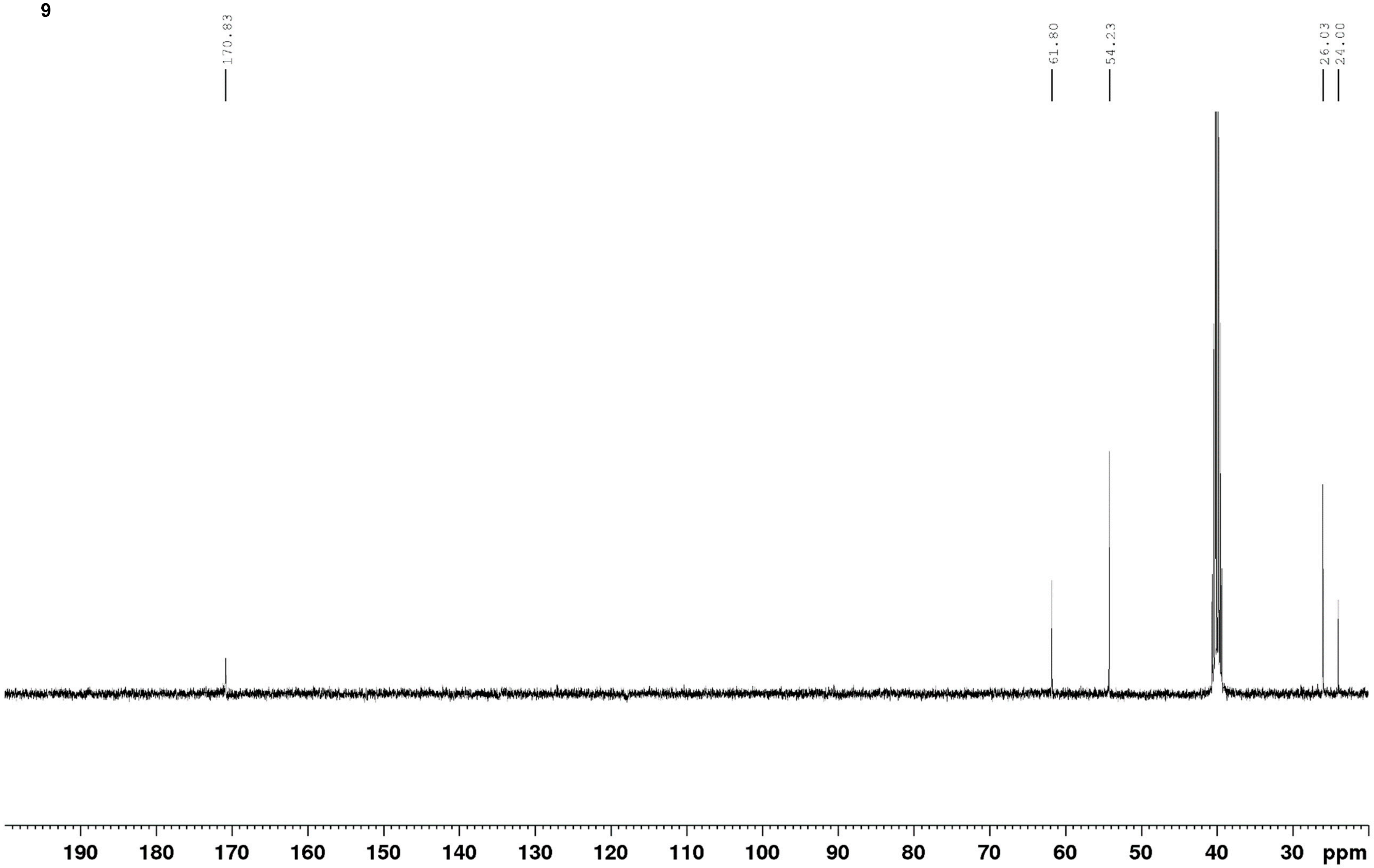
170.83
61.80
54.23
26.03
24.00
190
180
170
160
150
140
130
120
110
100
90
80
70
60
50
40
30
ppm

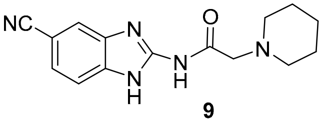
NC
N
N
H
N
H
O
N

9

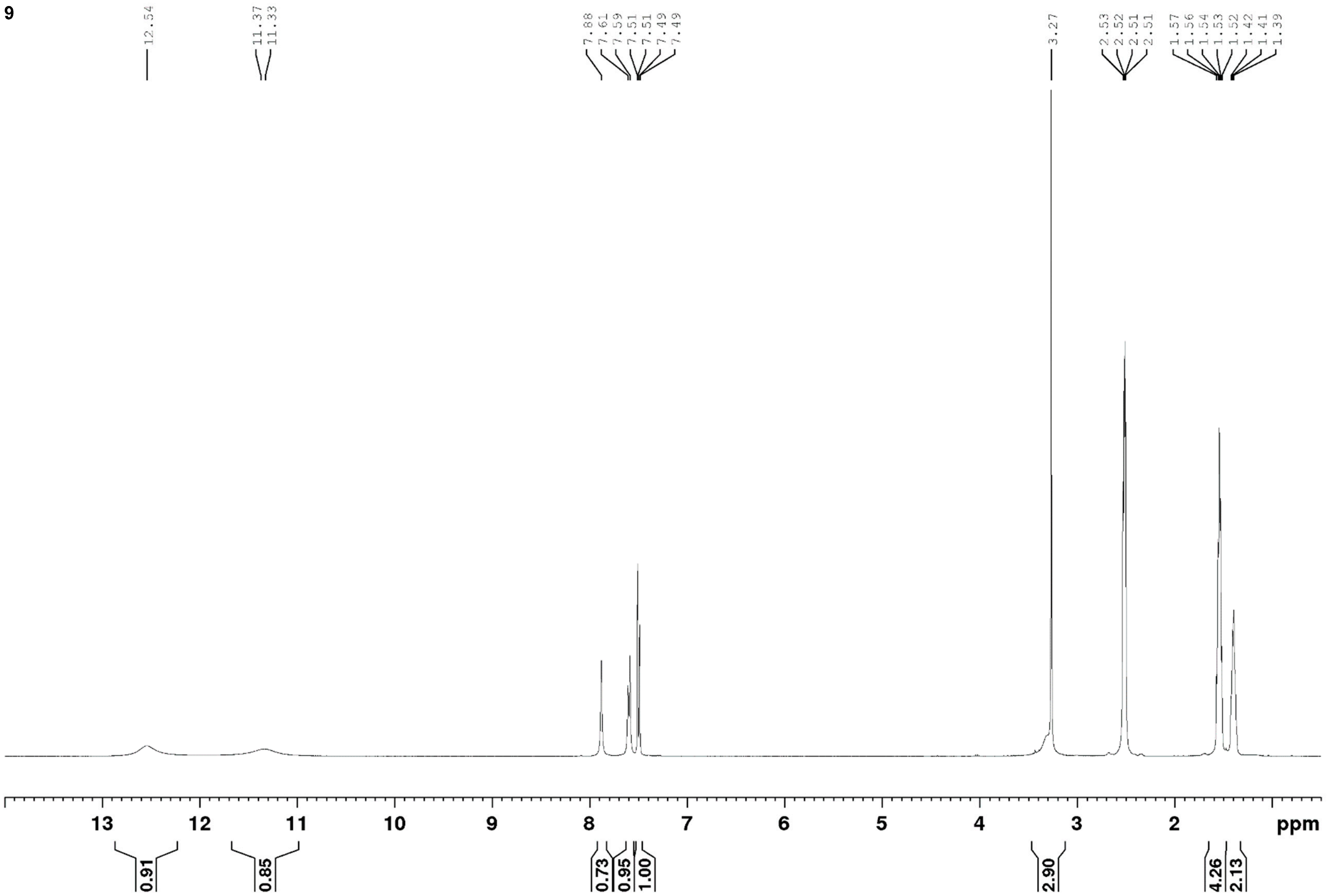
12.54
11.37
11.33
7.88
7.61
7.59
7.51
7.51
7.49
7.49
3.27
2.53
2.52
2.51
2.51
1.57
1.56
1.54
1.53
1.52
1.42
1.41
1.39
13 12 11 10 9 8 7 6 5 4 3 2 ppm
0.91
0.85
0.73
0.95
1.00
2.90
4.26
2.13

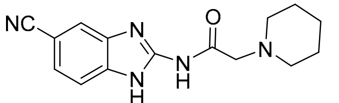
NC
N
N
H
N
H
O
N

9

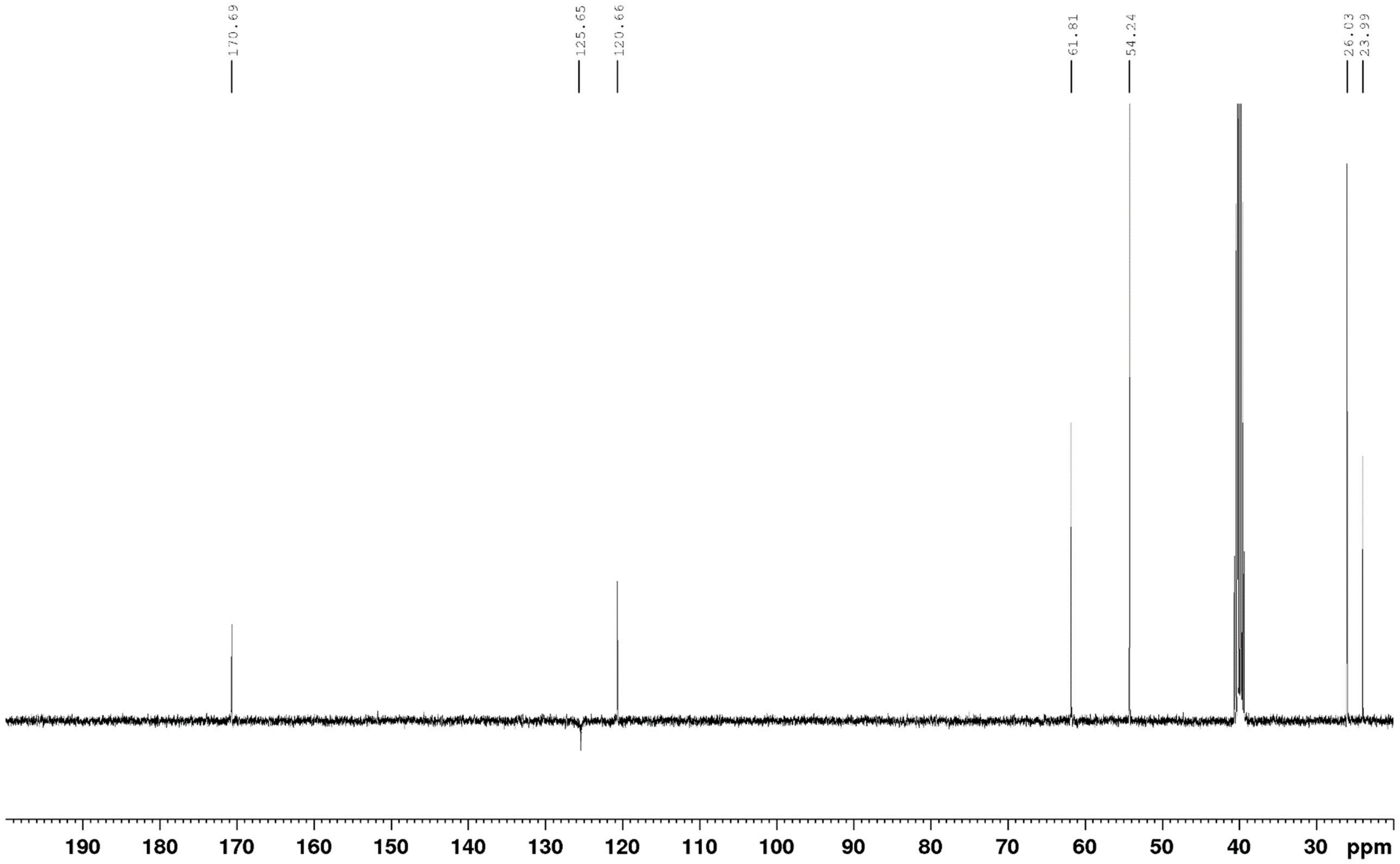
170.69
125.65
120.66
61.81
54.24
26.03
23.99
190
180
170
160
150
140
130
120
110
100
90
80
70
60
50
40
30
ppm

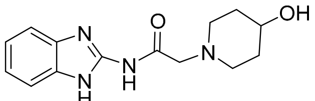

10

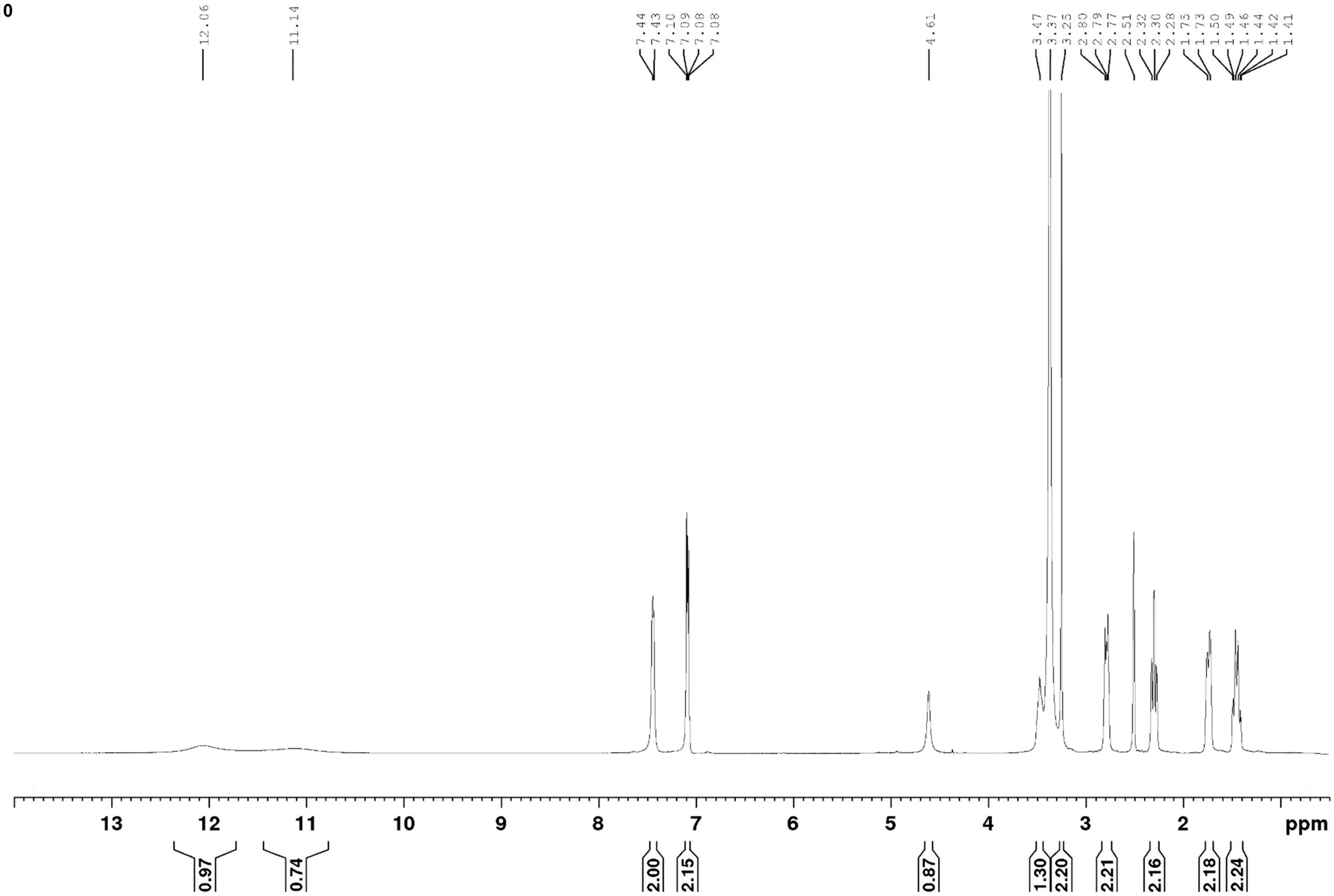

12.06
11.14
7.44
7.43
7.10
7.09
7.08
7.08
4.61
3.47
3.37
3.25
2.80
2.79
2.77
2.51
2.32
2.30
2.28
1.75
1.73
1.50
1.49
1.46
1.44
1.42
1.41
13
12
11
10
9
8
7
6
5
4
3
2
ppm
0.97
0.74
2.00
2.15
0.87
1.30
2.20
2.21
2.16
2.18
2.24

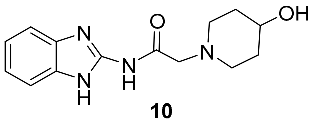

10

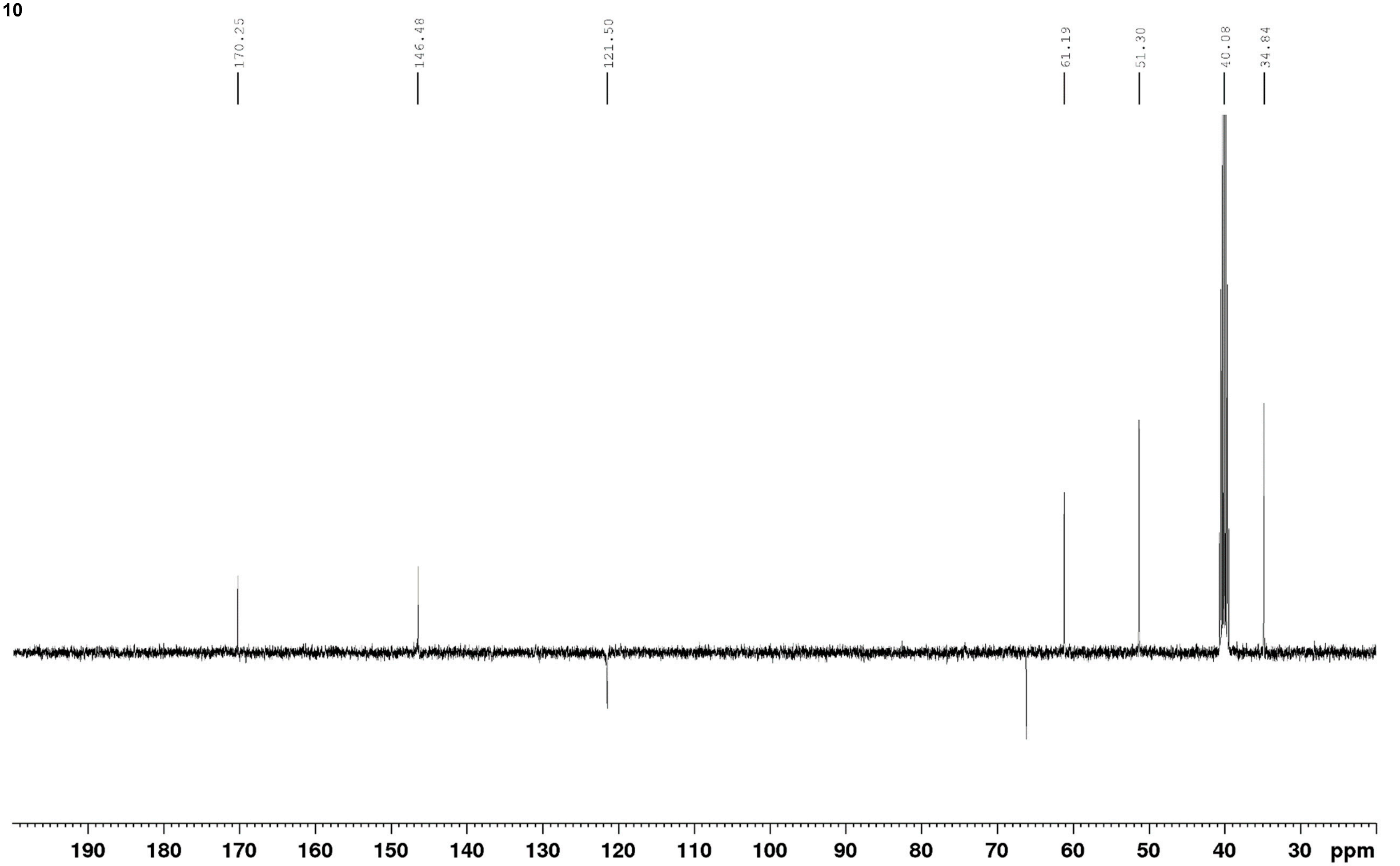
170.25
146.48
121.50
61.19
51.30
40.08
34.84
190
180
170
160
150
140
130
120
110
100
90
80
70
60
50
40
30
ppm

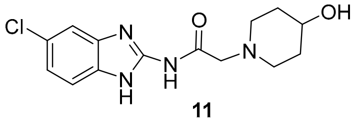
Cl
N
O
OH
N
H
N
N
H

11

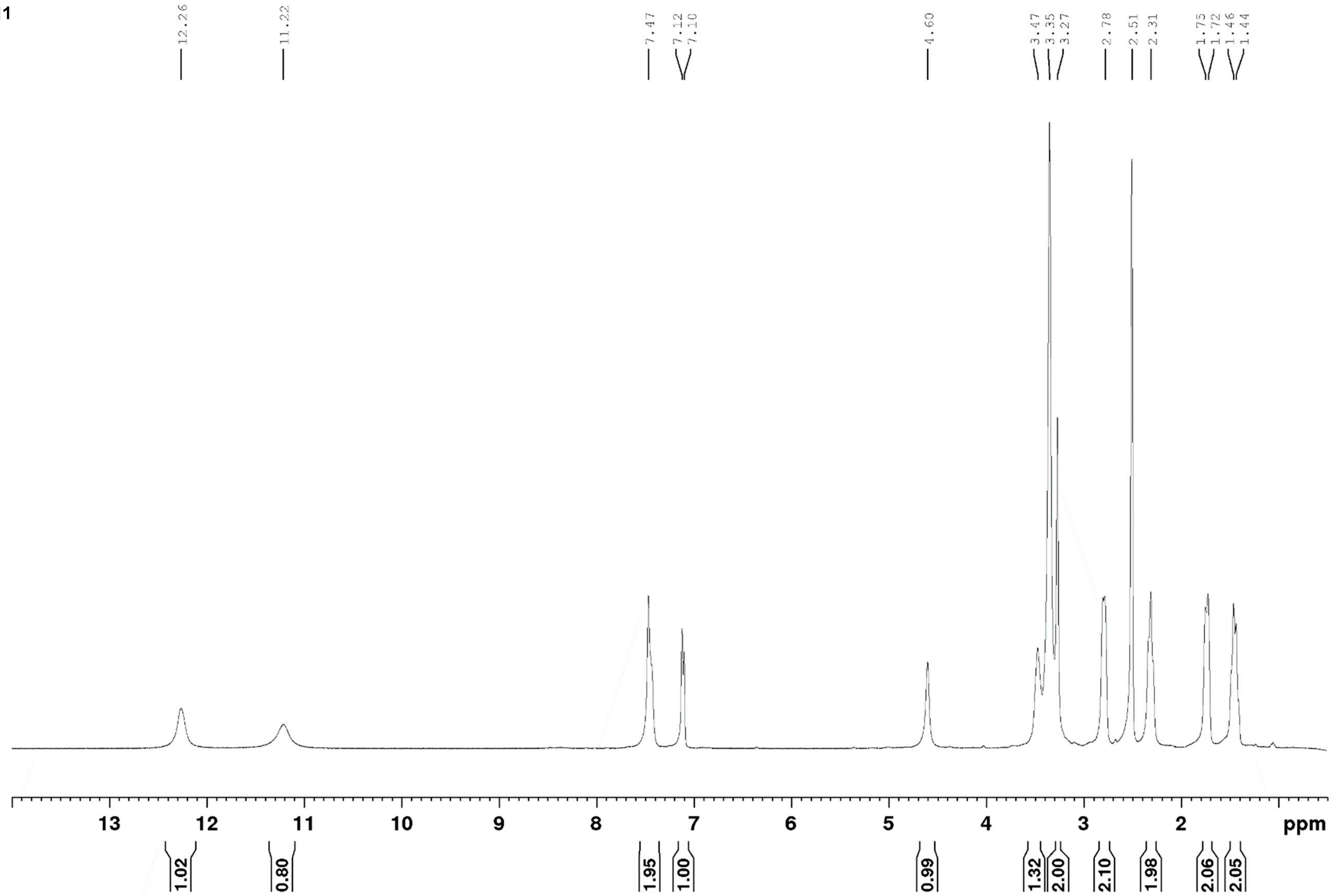
12.26
11.22
7.47
7.12
7.10
4.60
3.47
3.35
3.27
2.78
2.51
2.31
1.75
1.72
1.46
1.44
13
12
11
10
9
8
7
6
5
4
3
2
ppm
1.02
0.80
1.95
1.00
0.99
1.32
2.00
2.10
1.98
2.06
2.05

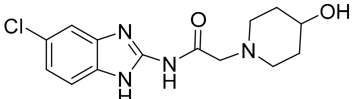

11

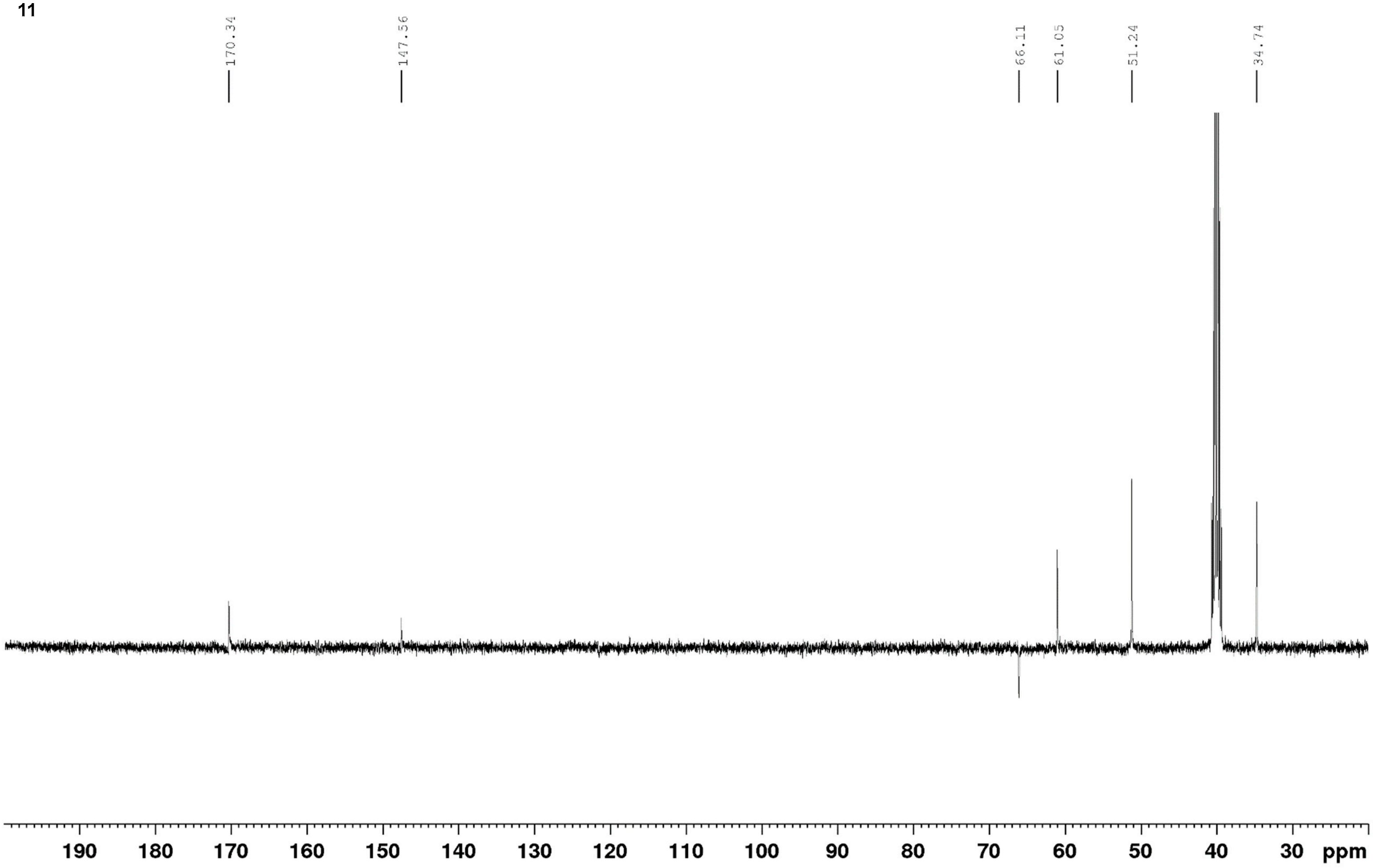
170.34
147.56
66.11
61.05
51.24
34.74
190 180 170 160 150 140 130 120 110 100 90 80 70 60 50 40 30 ppm

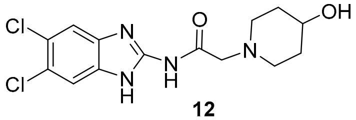

12

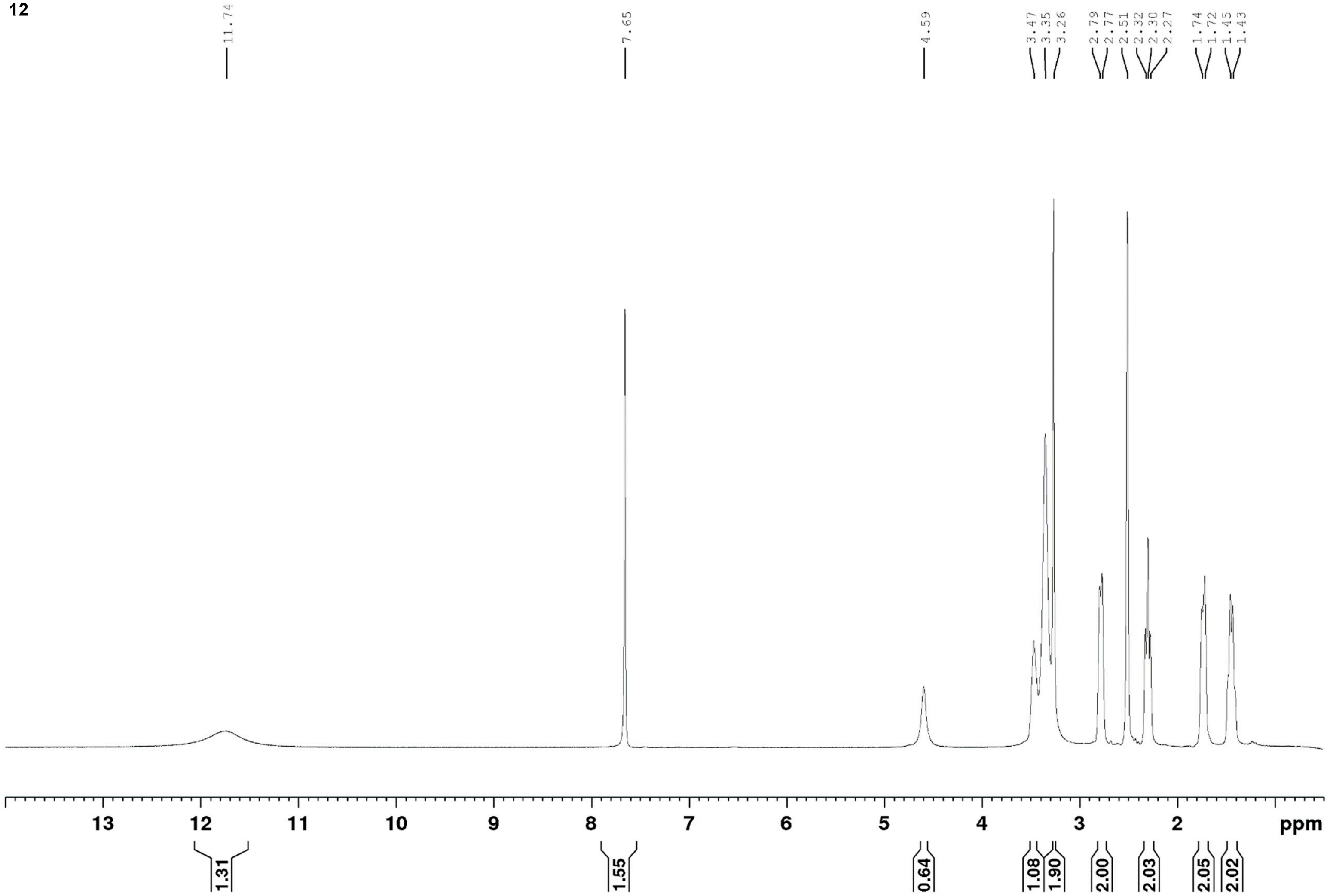

11.74
7.65
4.59
3.47
3.35
3.26
2.79
2.77
2.51
2.32
2.30
2.27
1.74
1.72
1.45
1.43
13
12
11
10
9
8
7
6
5
4
3
2
ppm
1.31
1.55
0.64
1.08
1.90
2.00
2.03
2.05
2.02

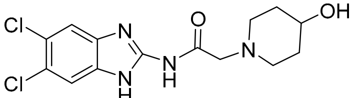
Cl
Cl
N
H
N
H
O
N
OH

12

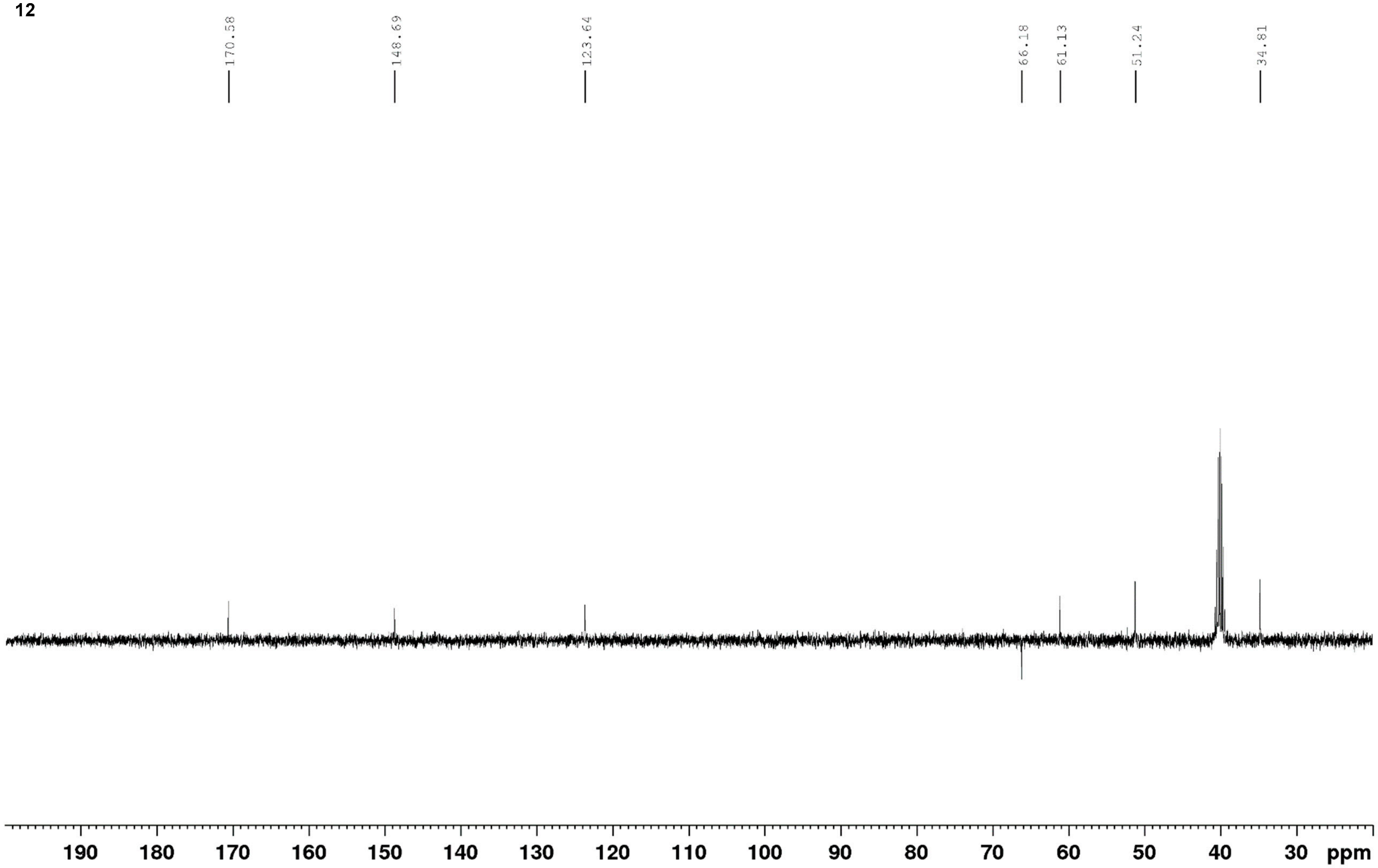
170.58
148.69
123.64
66.18
61.13
51.24
34.81
190
180
170
160
150
140
130
120
110
100
90
80
70
60
50
40
30
ppm

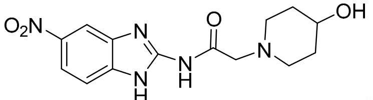
O2N
N
N
H
O
N
H
N
OH

13

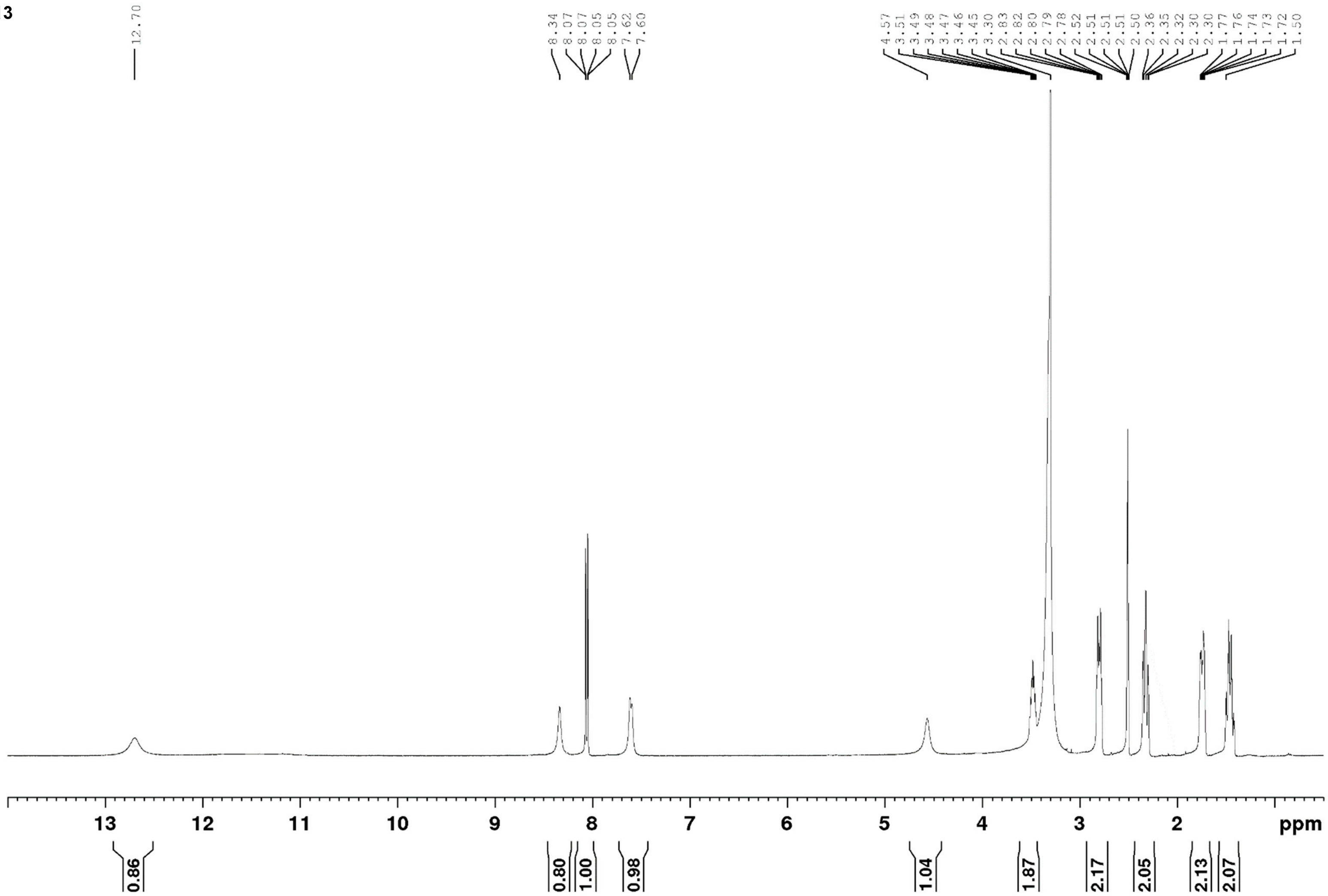
12.70
8.34
8.07
8.07
8.05
8.05
7.62
7.60
4.57
3.51
3.49
3.48
3.47
3.46
3.45
3.30
2.83
2.82
2.80
2.79
2.78
2.52
2.51
2.51
2.51
2.50
2.36
2.35
2.32
2.30
2.30
1.77
1.76
1.74
1.73
1.72
1.50
13
12
11
10
9
8
7
6
5
4
3
2
ppm
0.86
0.80
1.00
0.98
1.04
1.87
2.17
2.05
2.13
2.07

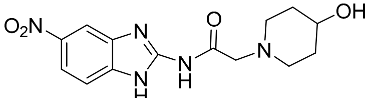

13

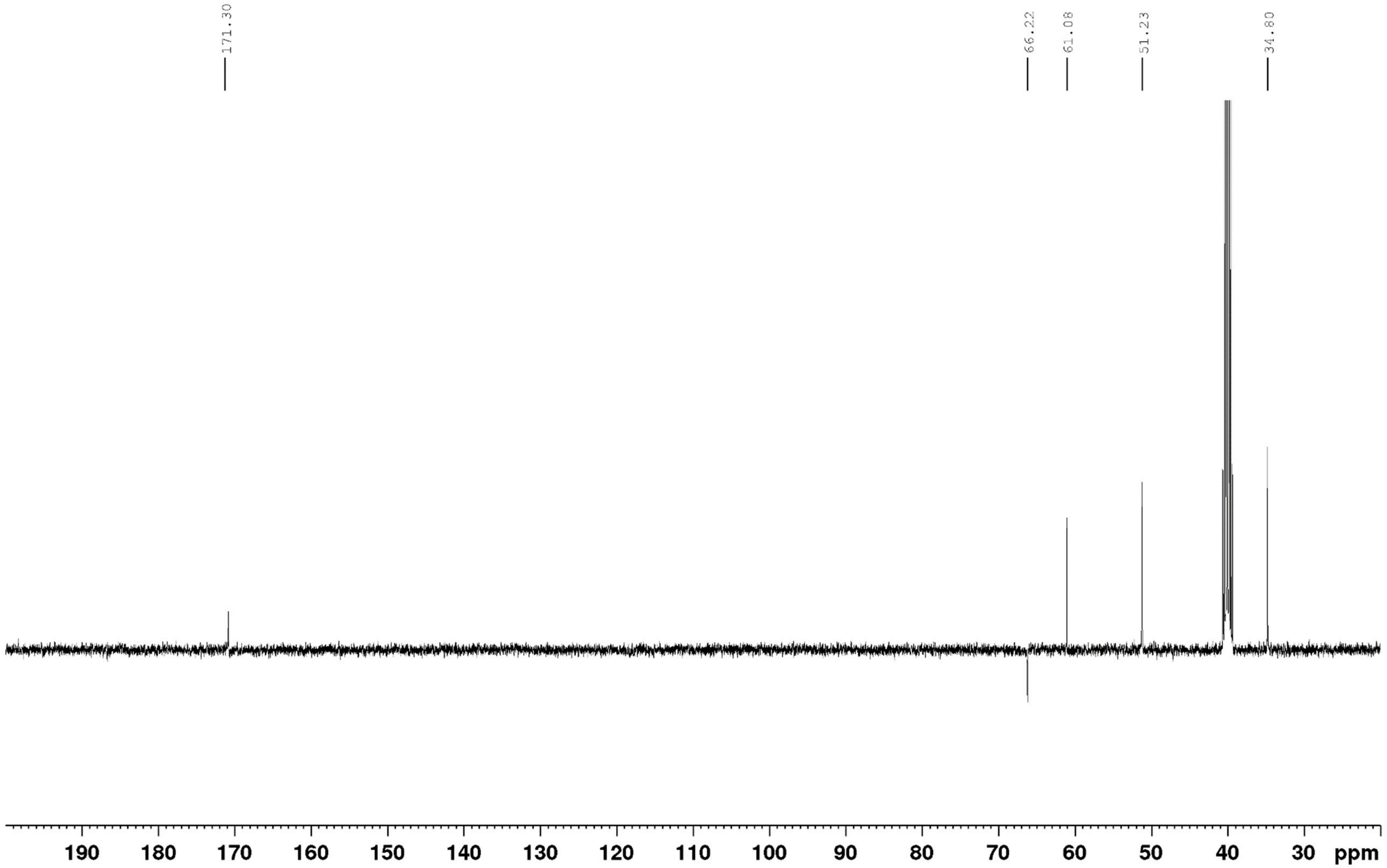
171.30
66.22
61.08
51.23
34.80
190 180 170 160 150 140 130 120 110 100 90 80 70 60 50 40 30 ppm

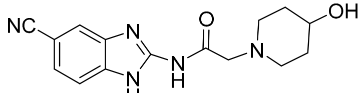

14

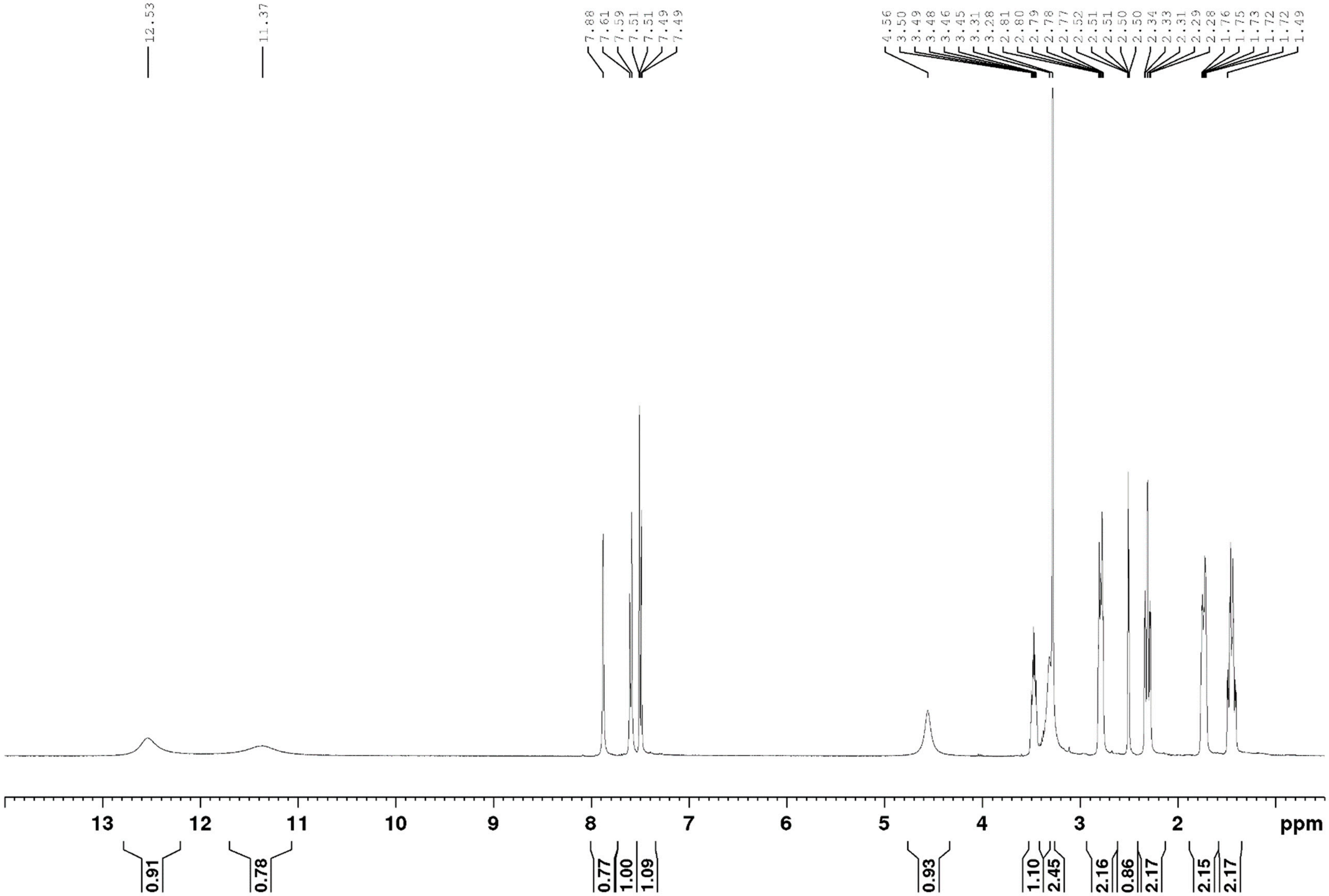

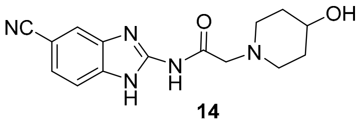

14

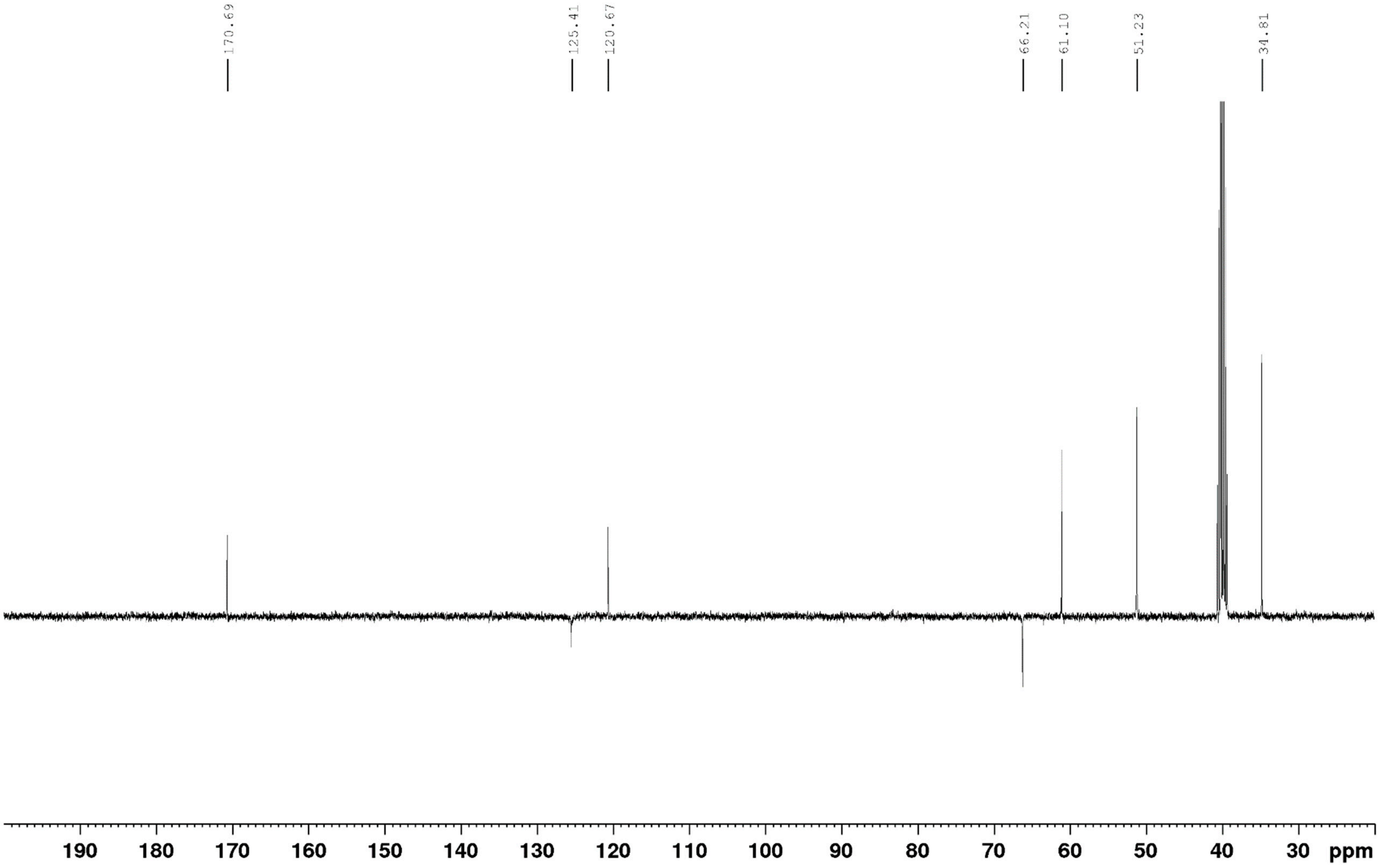
170.69
125.41
120.67
66.21
61.10
51.23
34.81
190
180
170
160
150
140
130
120
110
100
90
80
70
60
50
40
30
ppm

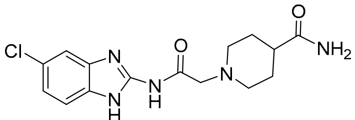

15

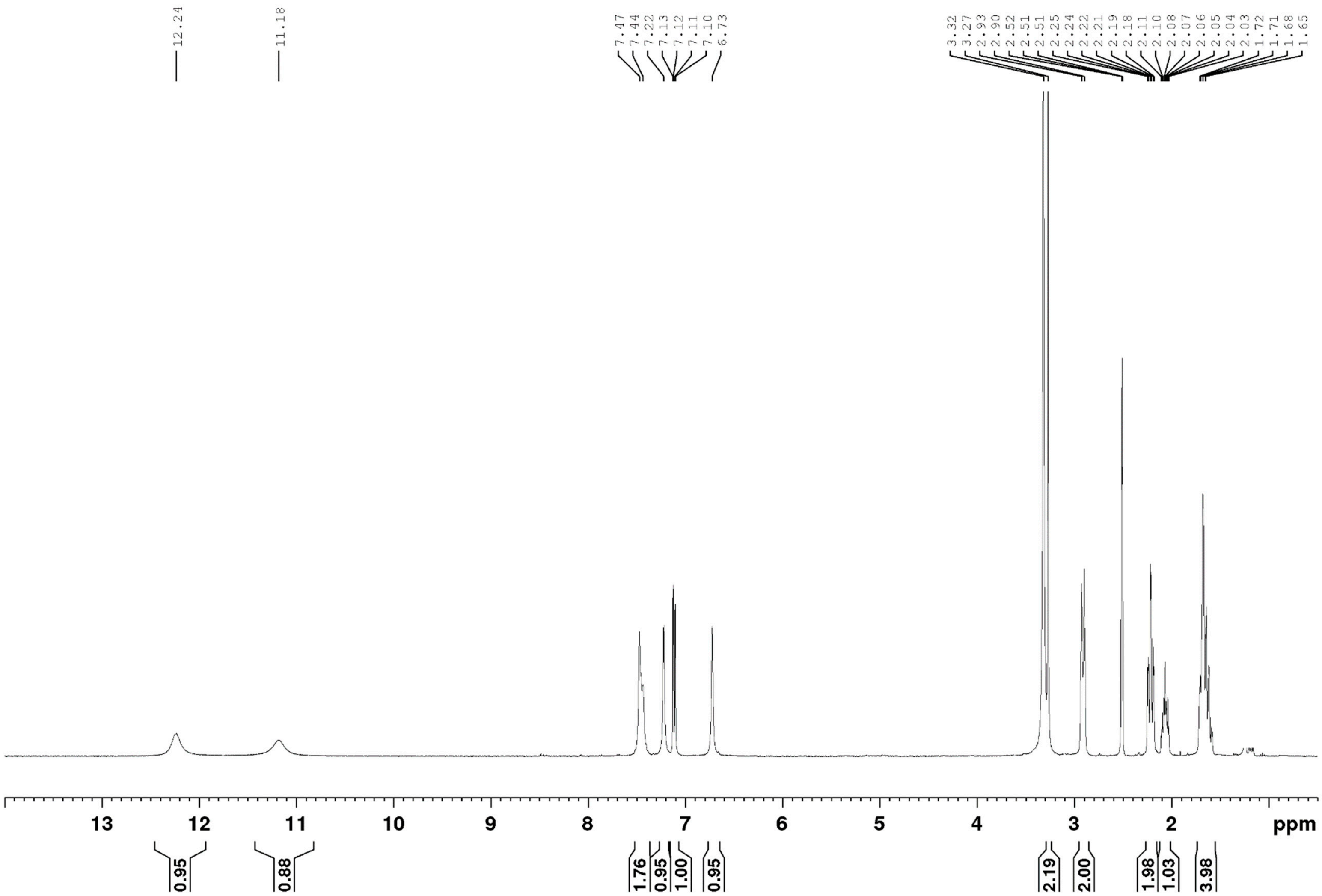
12.24
11.18
7.47
7.44
7.22
7.13
7.12
7.11
7.10
6.73
3.32
3.27
2.93
2.90
2.52
2.51
2.51
2.25
2.24
2.22
2.21
2.19
2.18
2.11
2.10
2.08
2.07
2.06
2.05
2.04
2.03
1.72
1.71
1.68
1.65
13
12
11
10
9
8
7
6
5
4
3
2
ppm
0.95
0.88
1.76
0.95
1.00
0.95
2.19
2.00
1.98
1.03
3.98

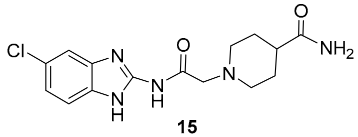

15

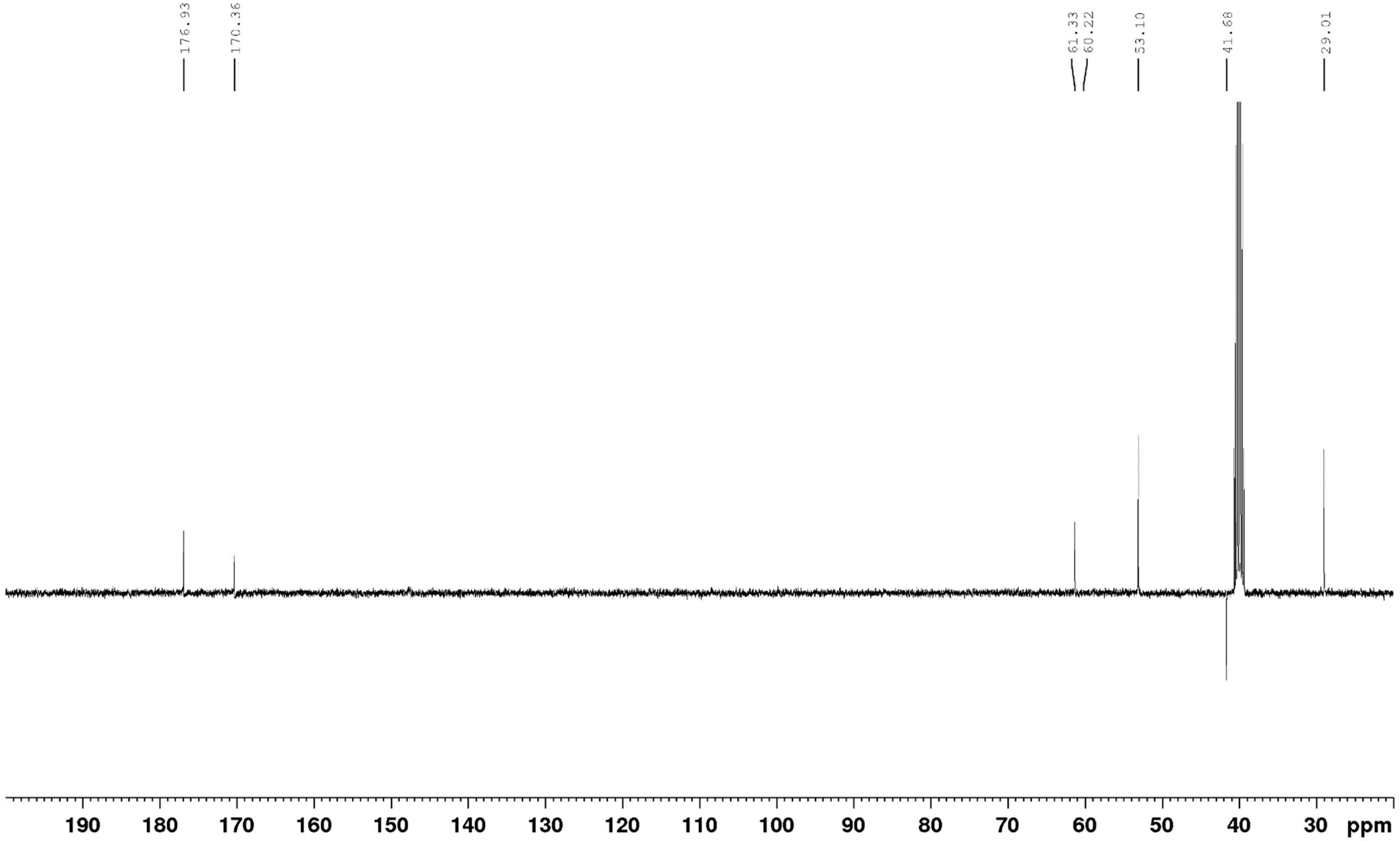

176.93
170.36
61.33
60.22
53.10
41.68
29.01
190
180
170
160
150
140
130
120
110
100
90
80
70
60
50
40
30
ppm

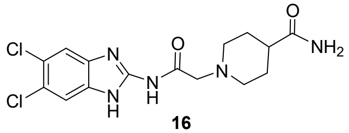
Cl
Cl
N
H
O
NH2

16

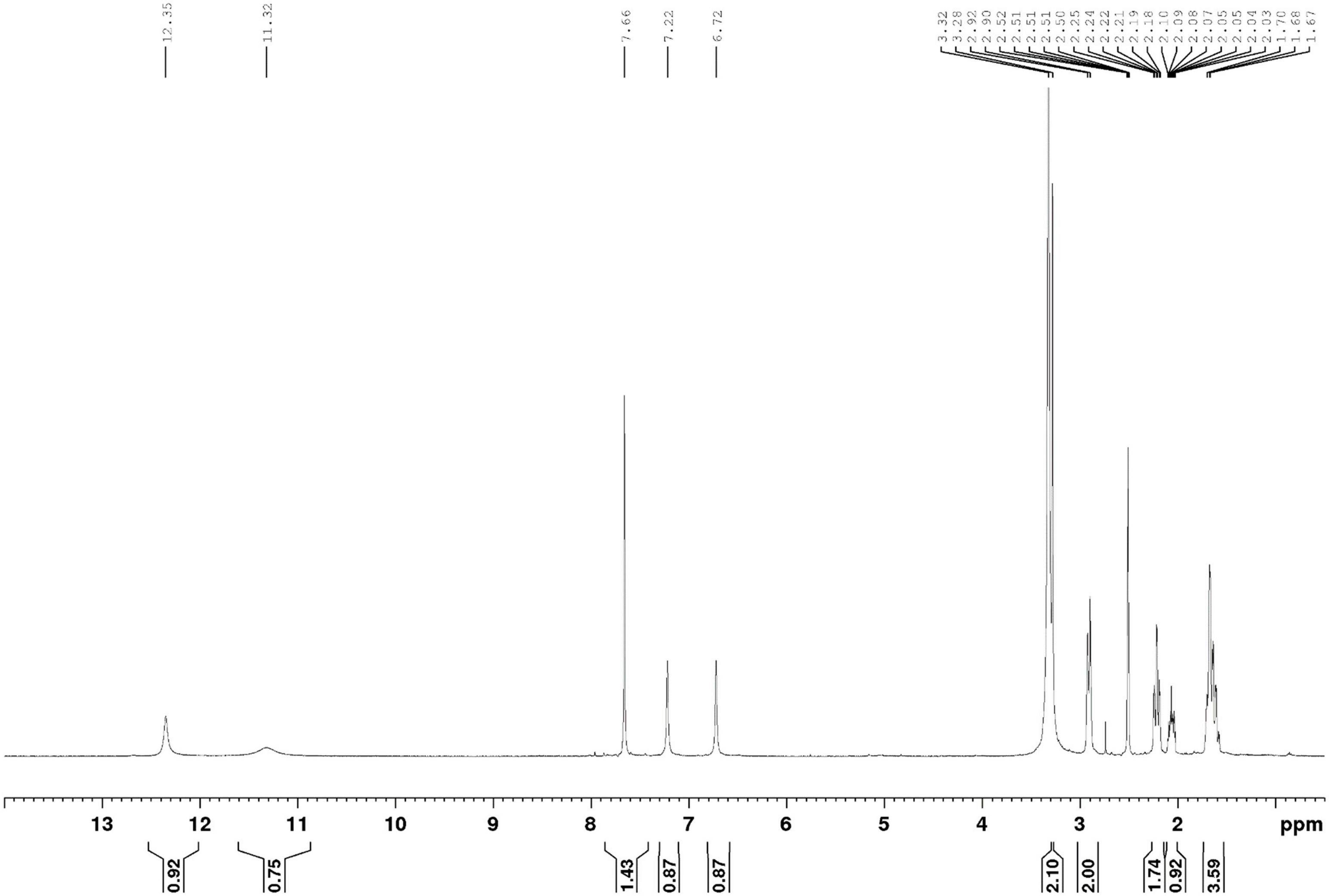
12.35
11.32
7.66
7.22
6.72
3.32
3.28
2.92
2.90
2.52
2.51
2.51
2.50
2.25
2.24
2.22
2.21
2.19
2.18
2.10
2.09
2.08
2.07
2.05
2.04
2.03
1.70
1.68
1.67
13
12
11
10
9
8
7
6
5
4
3
2
ppm
0.92
0.75
1.43
0.87
0.87
2.10
2.00
1.74
0.92
3.59

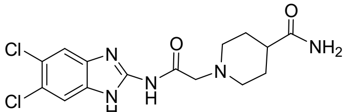
Cl
Cl
N
H
N
H
O
N
O
NH2

16

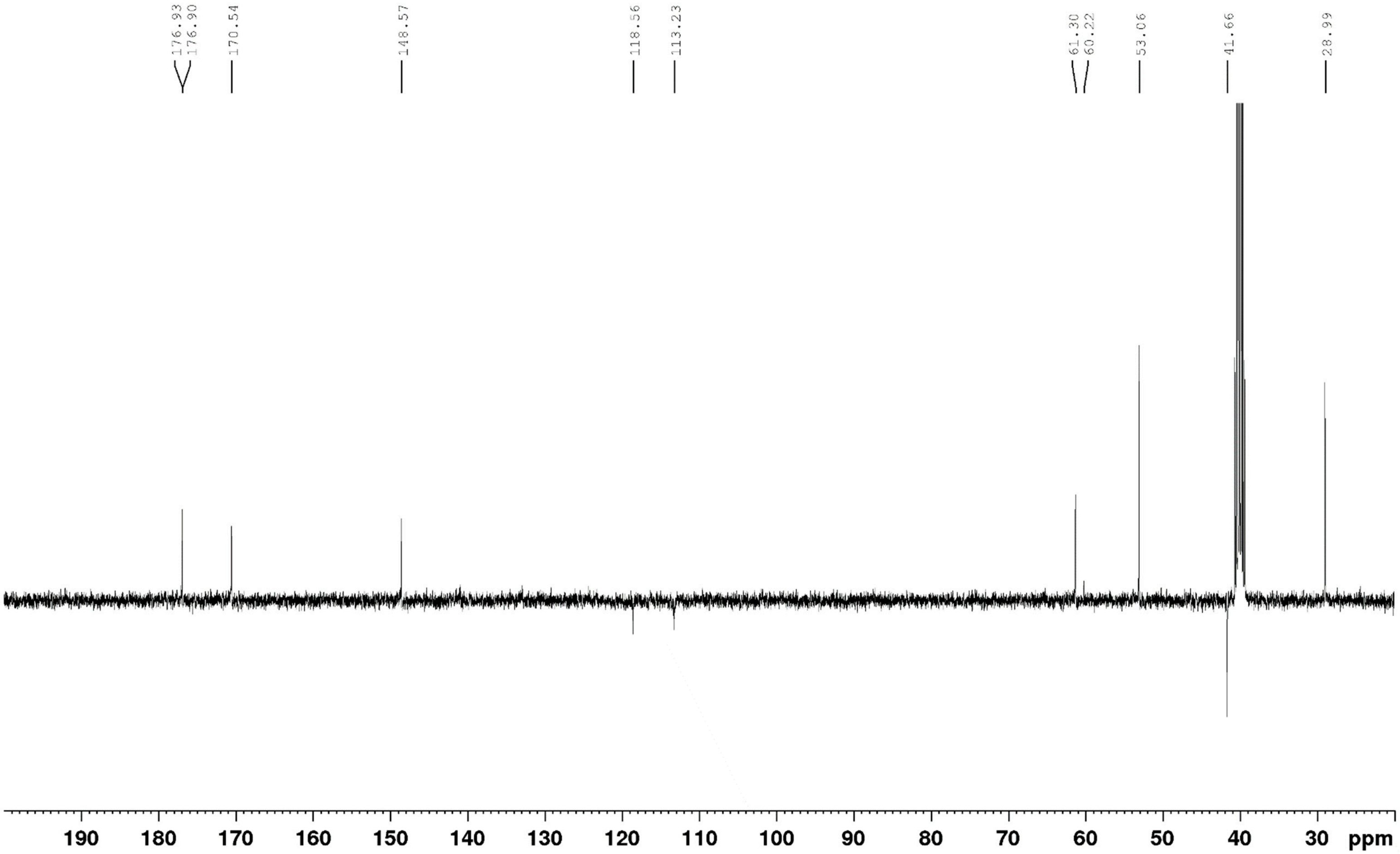
176.93
176.90
170.54
148.57
118.56
113.23
61.30
60.22
53.06
41.66
28.99
190
180
170
160
150
140
130
120
110
100
90
80
70
60
50
40
30
ppm

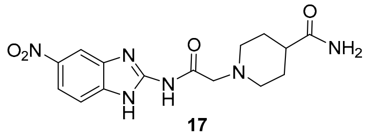

17

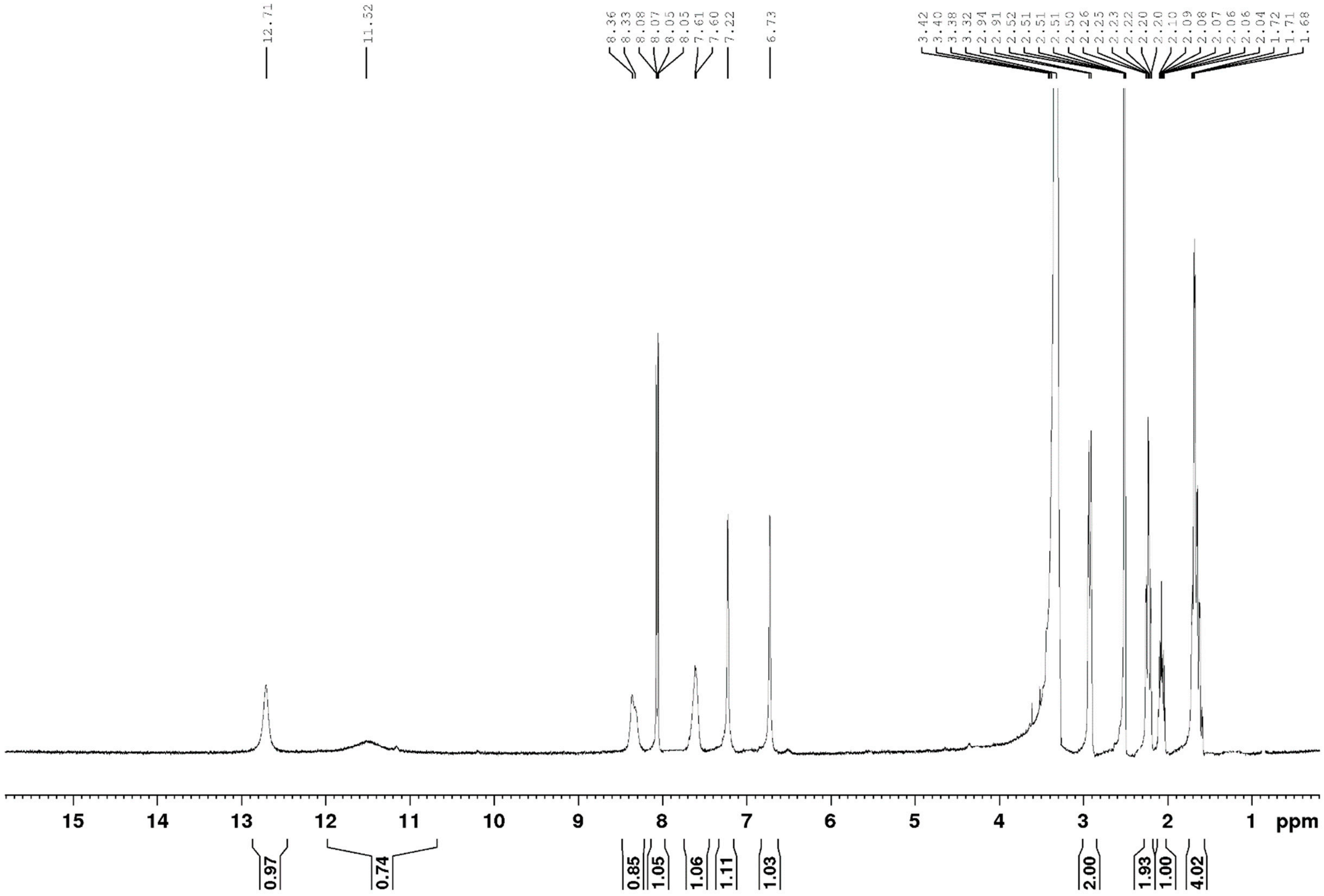

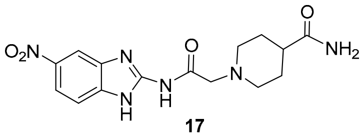
O2N
N
N
H
N
H
O
N
O
NH2

17

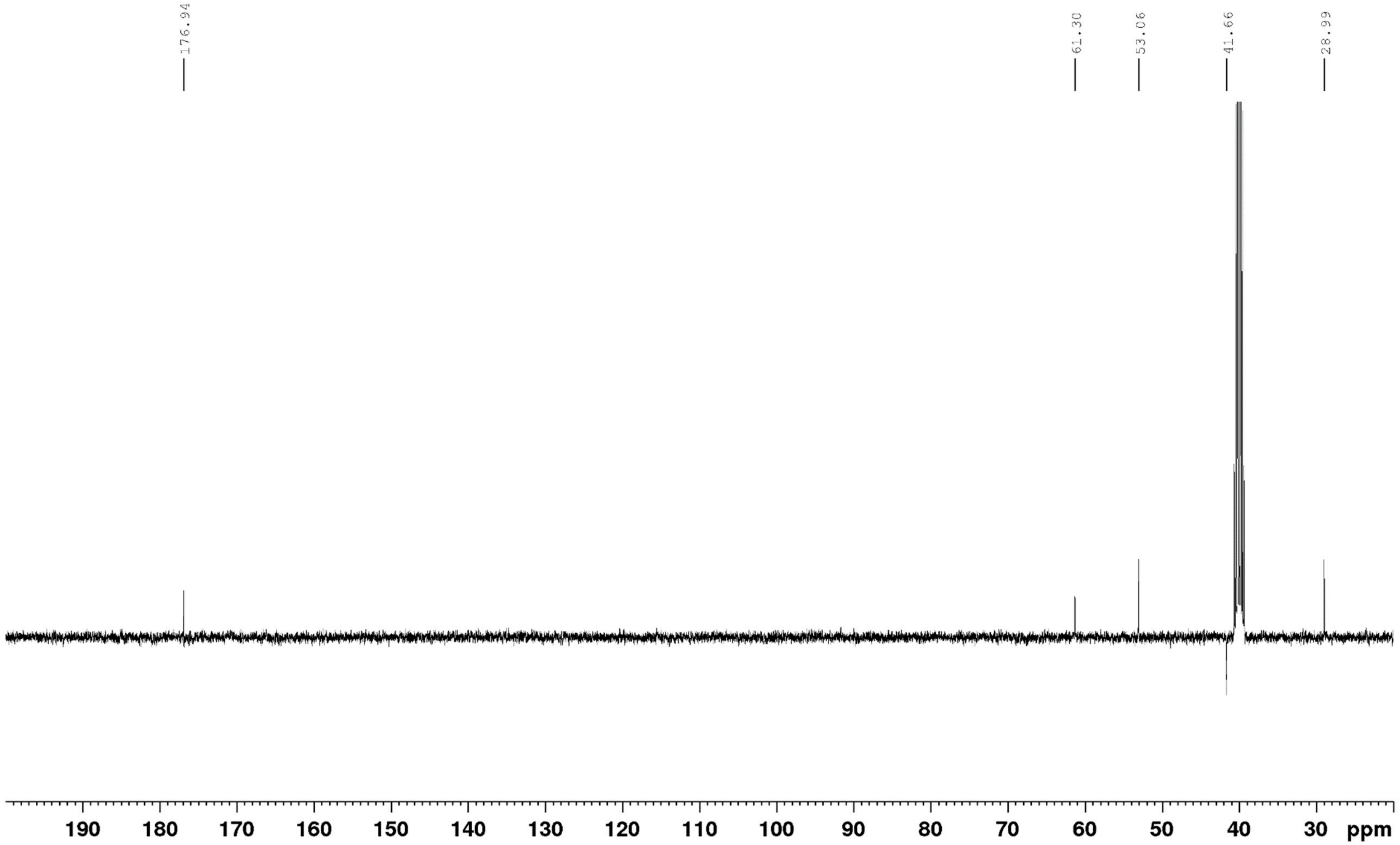
176.94
61.30
53.06
41.66
28.99
190
180
170
160
150
140
130
120
110
100
90
80
70
60
50
40
30
ppm

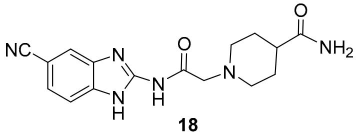
NC
N
N
H
O
N
H
N
O
NH2

18

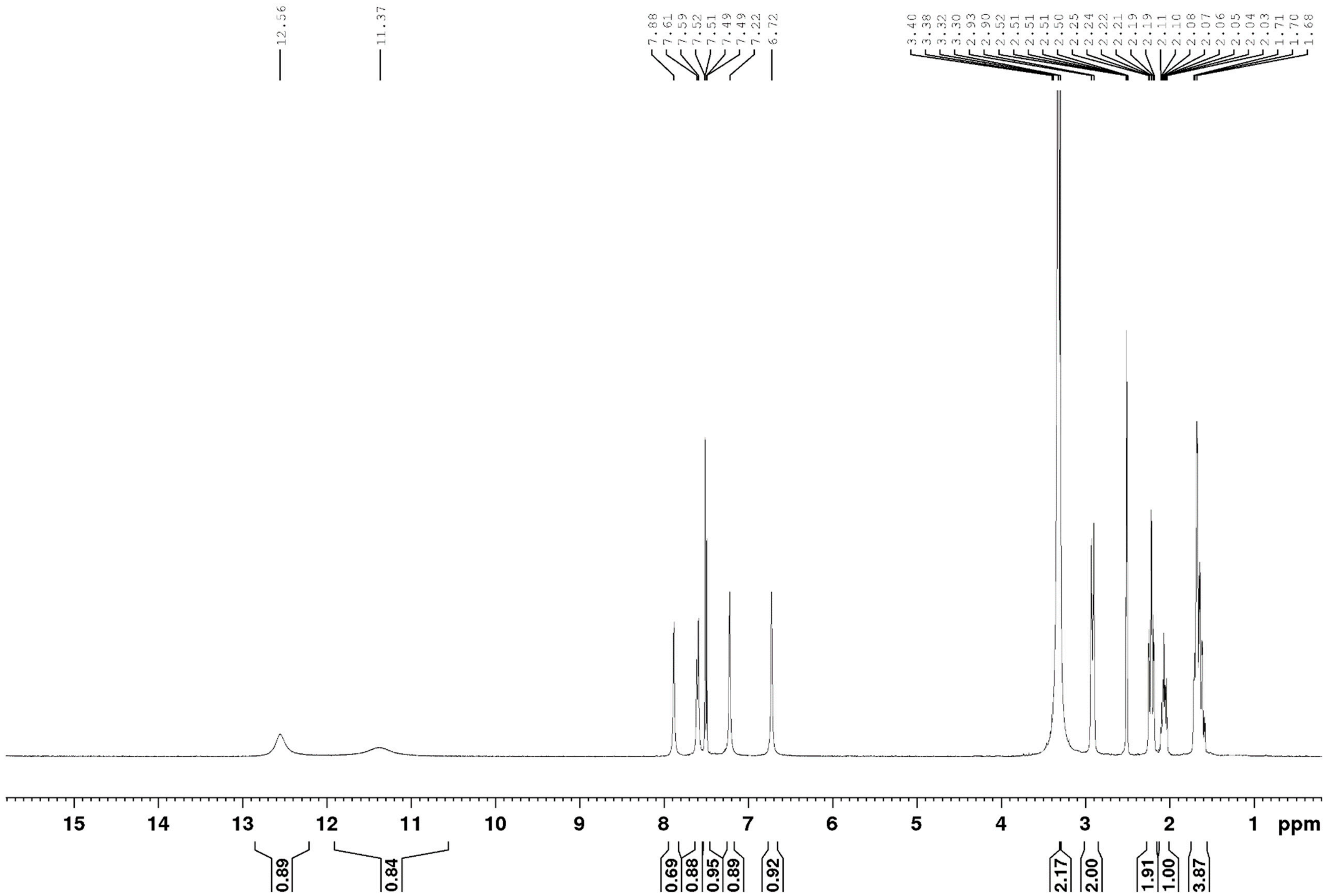
12.56
11.37
7.88
7.61
7.59
7.52
7.51
7.49
7.49
7.22
6.72
3.40
3.38
3.32
3.30
2.93
2.90
2.52
2.51
2.51
2.51
2.50
2.25
2.24
2.22
2.21
2.19
2.19
2.11
2.10
2.08
2.07
2.06
2.05
2.04
2.03
1.71
1.70
1.68
15 14 13 12 11 10 9 8 7 6 5 4 3 2 1 ppm
0.89
0.84
0.69
0.88
0.95
0.89
0.92
2.17
2.00
1.91
1.00
3.87

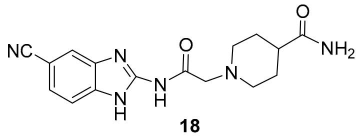

18

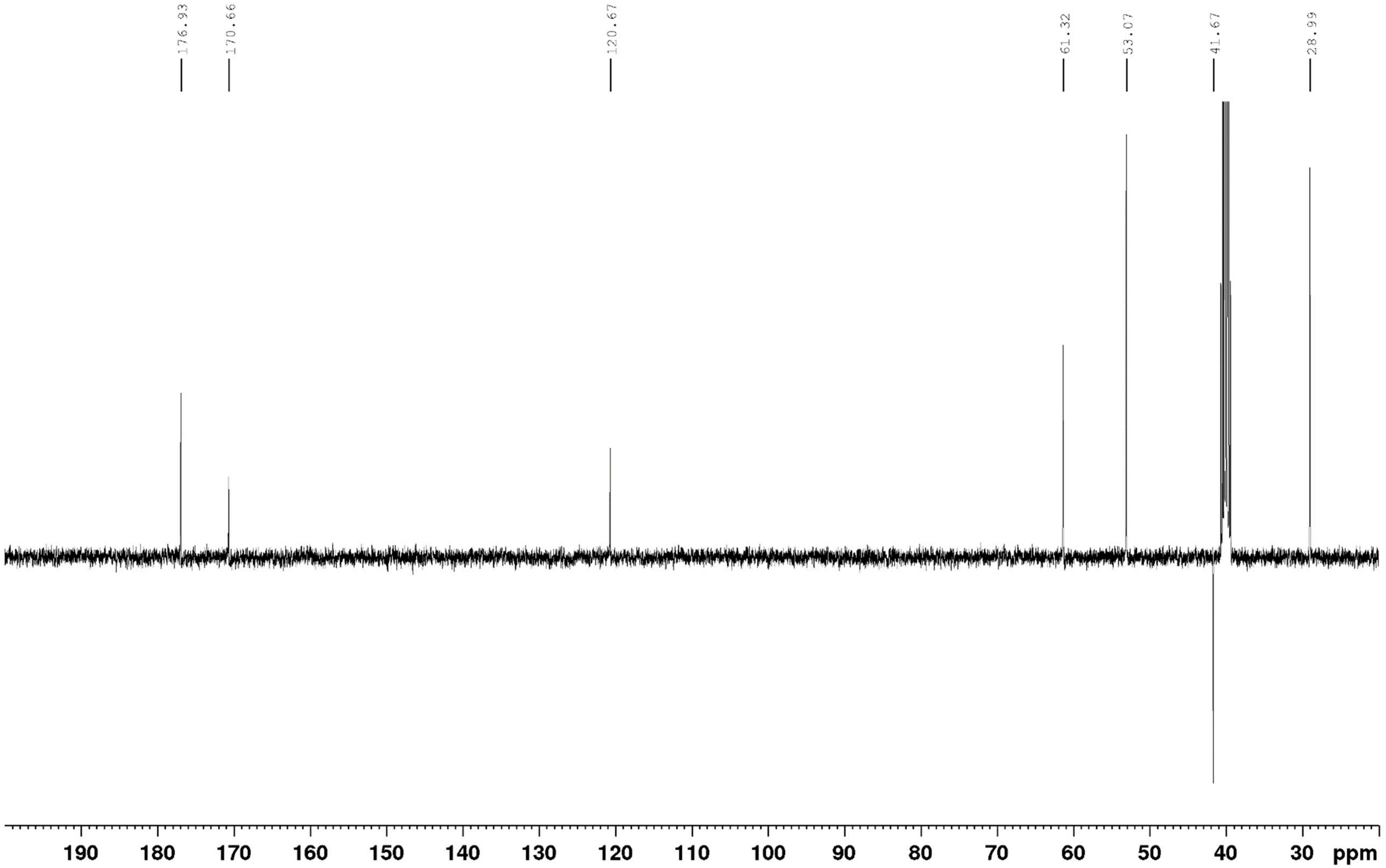
176.93
170.66
120.67
61.32
53.07
41.67
28.99
190
180
170
160
150
140
130
120
110
100
90
80
70
60
50
40
30
ppm

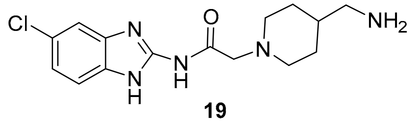

19

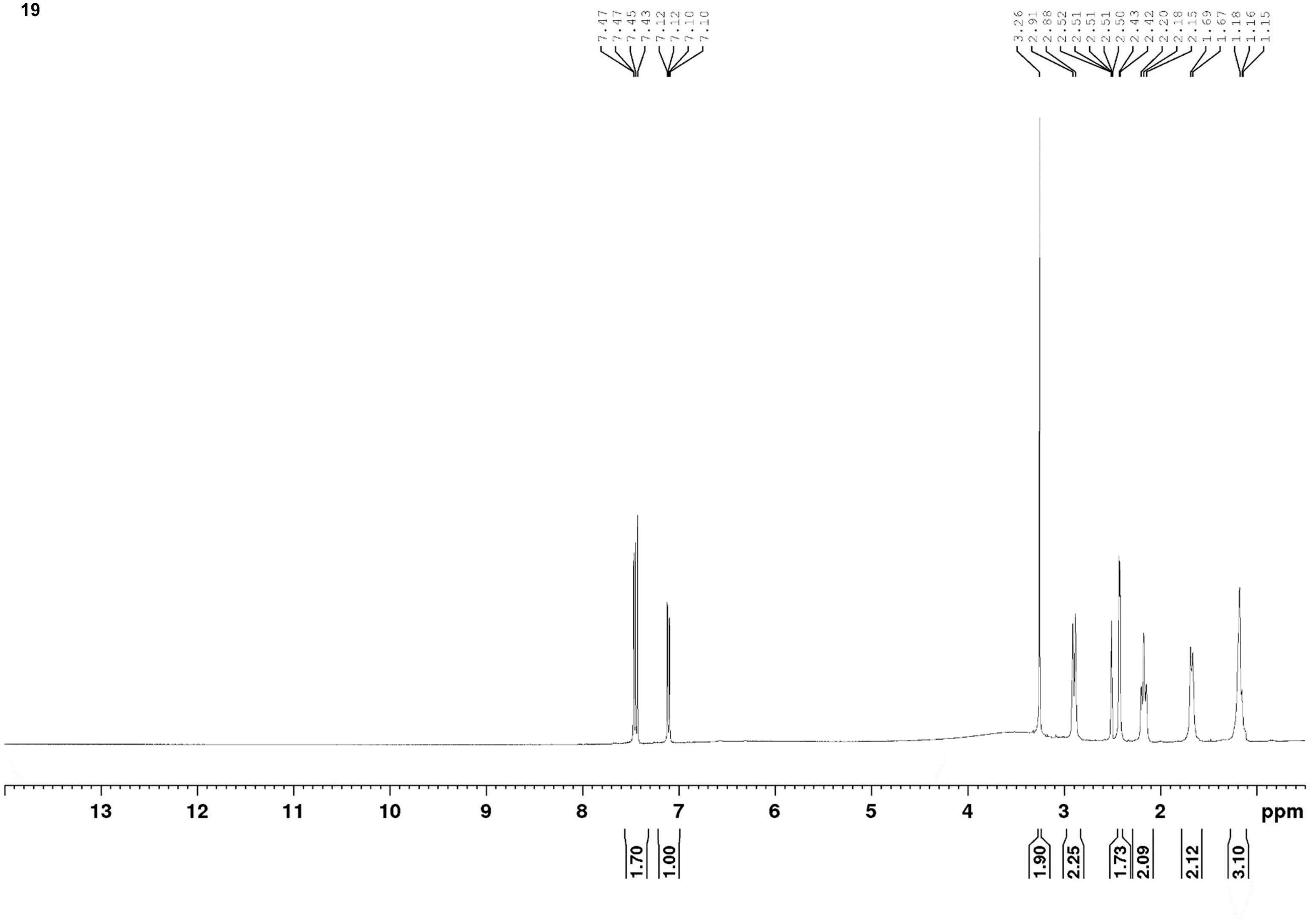
7.47
7.47
7.45
7.43
7.12
7.12
7.10
7.10
3.26
2.91
2.88
2.52
2.51
2.51
2.51
2.50
2.43
2.42
2.20
2.18
2.15
1.69
1.67
1.18
1.16
1.15
13
12
11
10
9
8
7
6
5
4
3
2
ppm
1.70
1.00
1.90
2.25
1.73
2.09
2.12
3.10

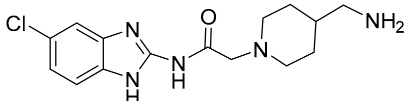

19

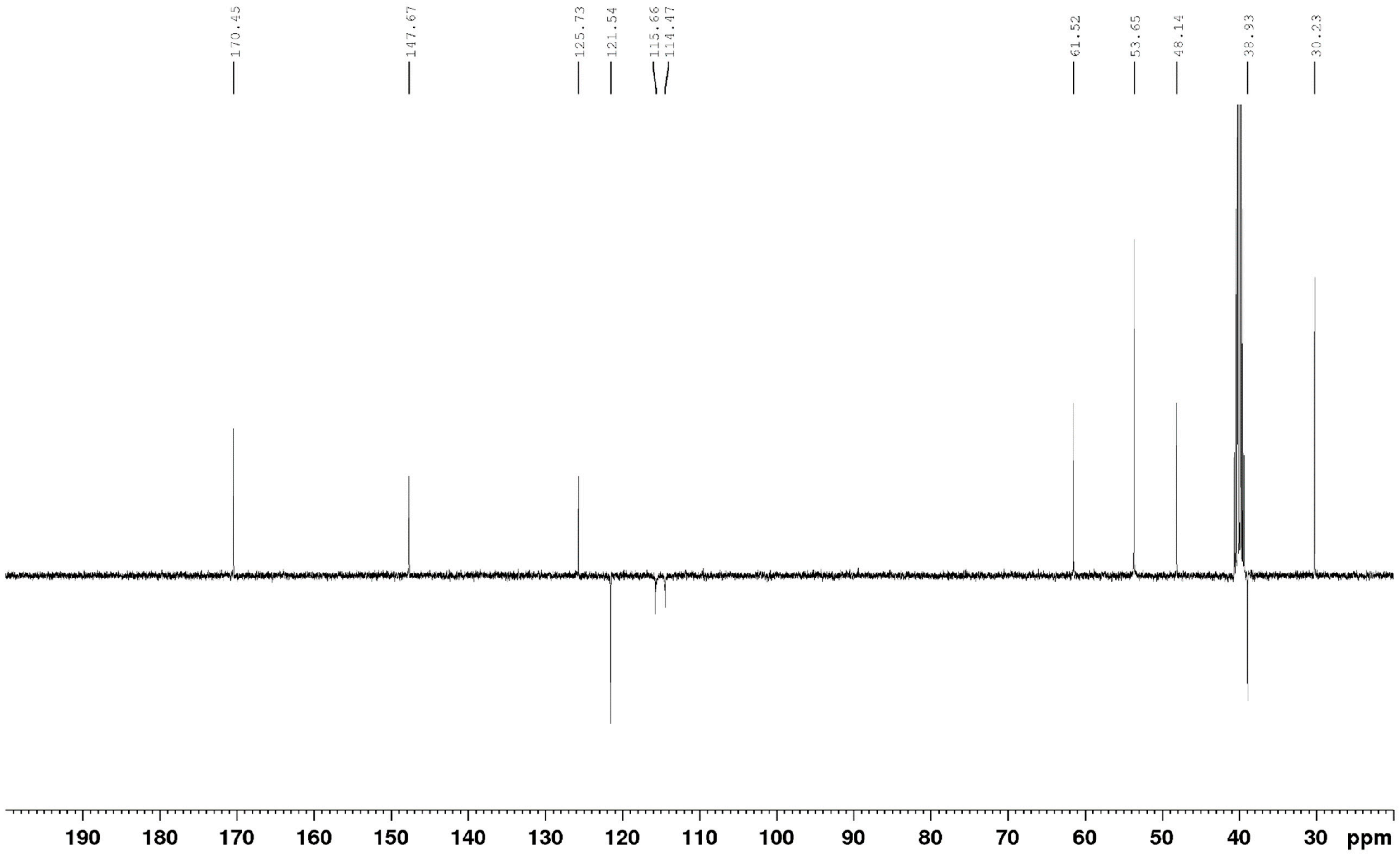
170.45
147.67
125.73
121.54
115.66
114.47
61.52
53.65
48.14
38.93
30.23
190
180
170
160
150
140
130
120
110
100
90
80
70
60
50
40
30
ppm

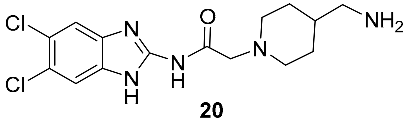
Cl
Cl
N
N
H
N
H
O
N
NH2

20

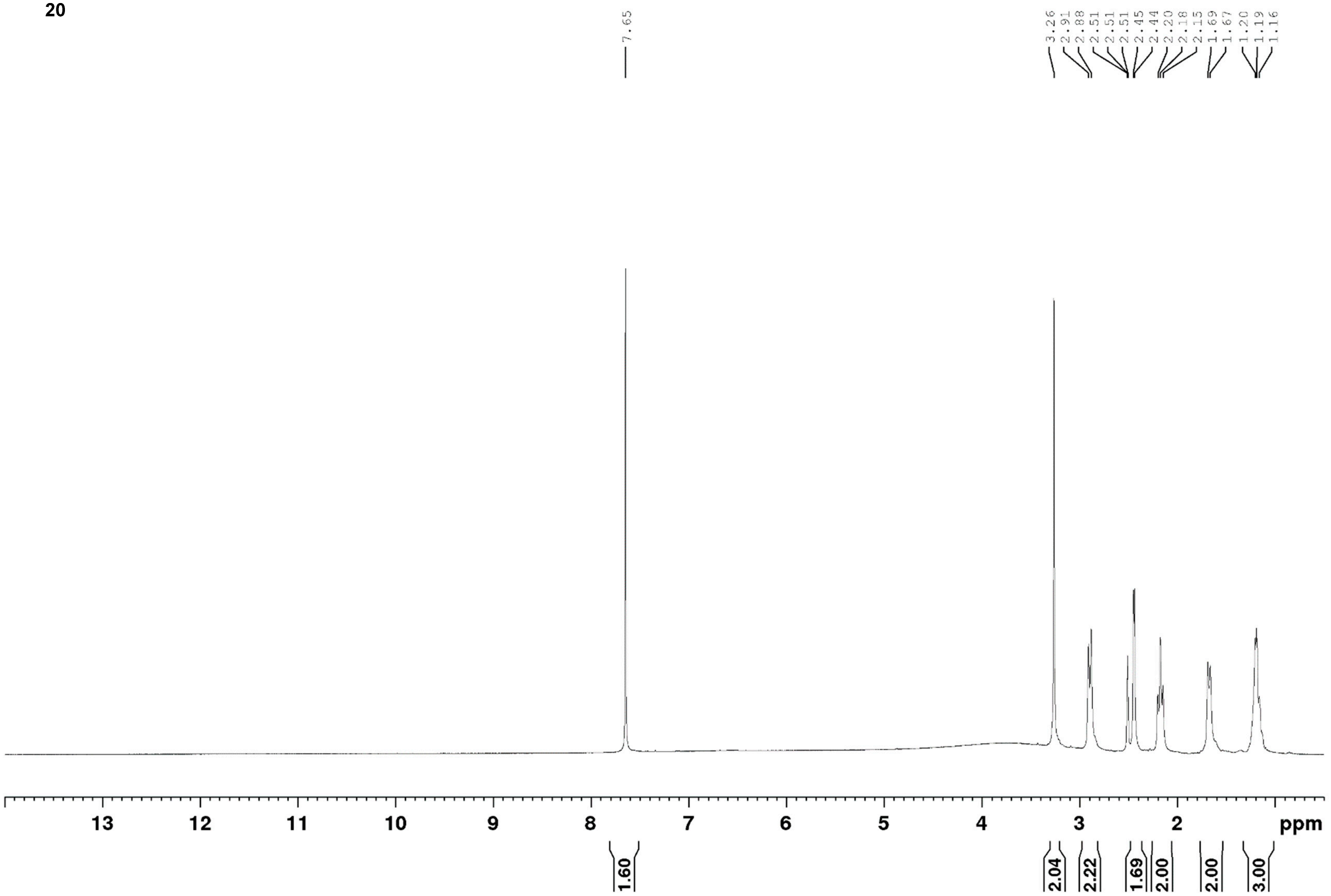
7.65
3.26
2.91
2.88
2.51
2.51
2.51
2.45
2.44
2.20
2.18
2.15
1.69
1.67
1.20
1.19
1.16
13
12
11
10
9
8
7
6
5
4
3
2
ppm
1.60
2.04
2.22
1.69
2.00
2.00
3.00

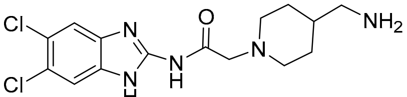
Cl
Cl
N
H
N
H
O
N
NH2

20

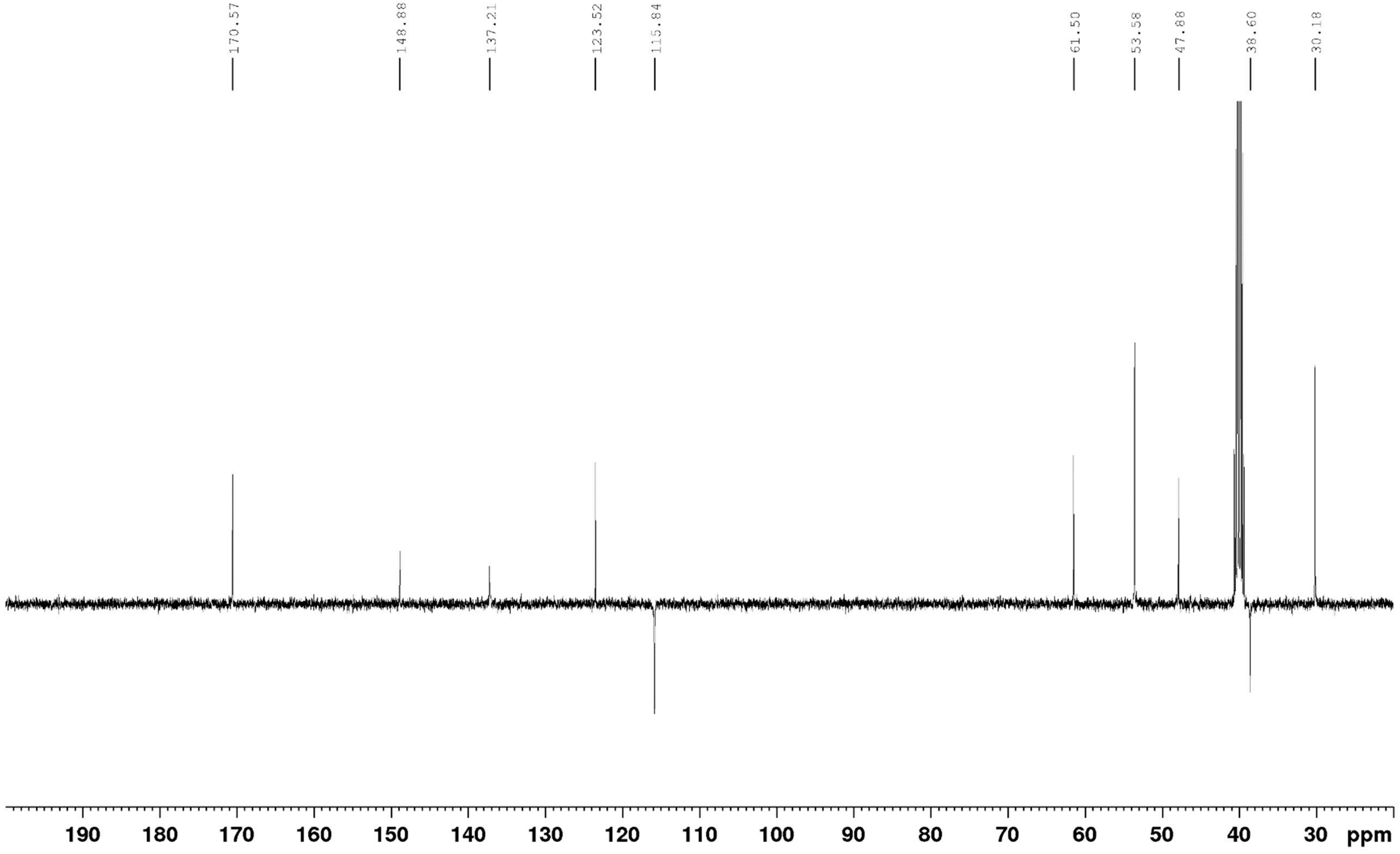
170.57
148.88
137.21
123.52
115.84
61.50
53.58
47.88
38.60
30.18
190
180
170
160
150
140
130
120
110
100
90
80
70
60
50
40
30
ppm

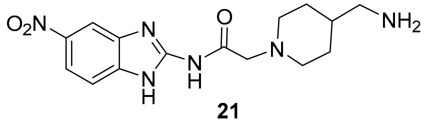

O2N
N
N
H
N
H
O
N
NH2

21

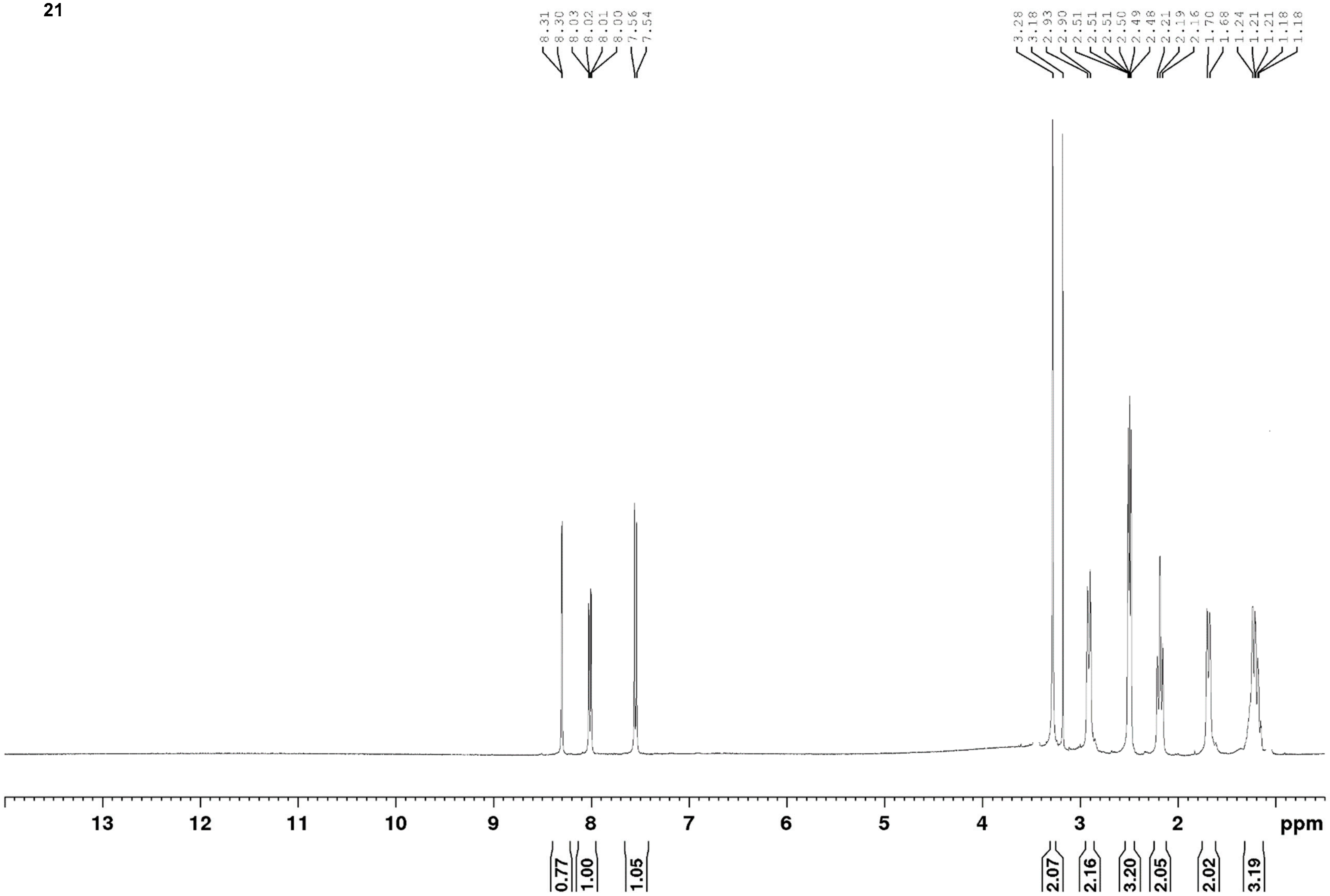

8.31
8.30
8.03
8.02
8.01
8.00
7.56
7.54
3.28
3.18
2.93
2.90
2.51
2.51
2.51
2.50
2.49
2.48
2.21
2.19
2.16
1.70
1.68
1.24
1.21
1.21
1.18
1.18
13
12
11
10
9
8
7
6
5
4
3
2
ppm
0.77
1.00
1.05
2.07
2.16
3.20
2.05
2.02
3.19

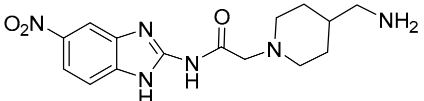
O2N
N
N
H
H
O
N
NH2

21

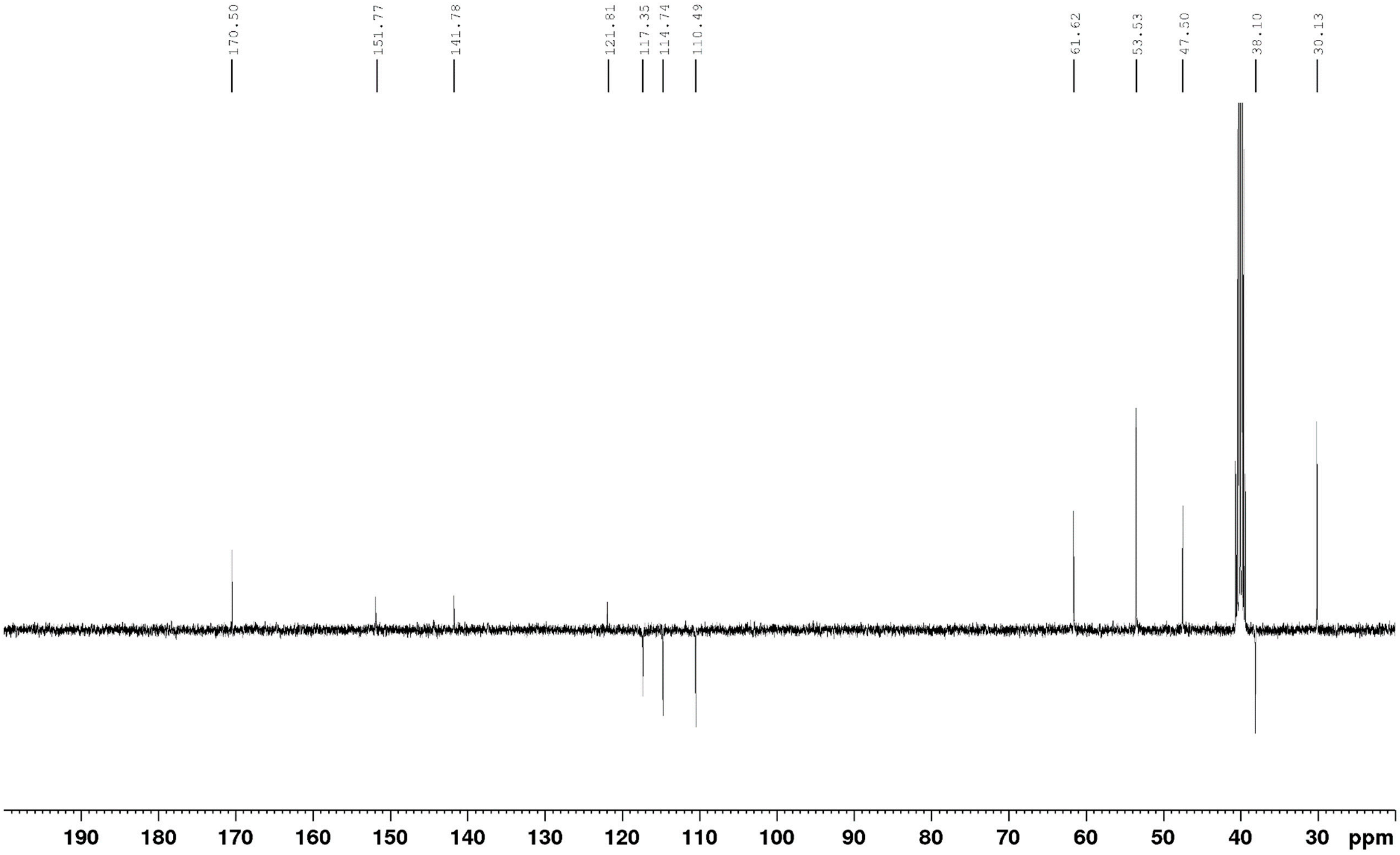
170.50
151.77
141.78
121.81
117.35
114.74
110.49
61.62
53.53
47.50
38.10
30.13
190 180 170 160 150 140 130 120 110 100 90 80 70 60 50 40 30 ppm

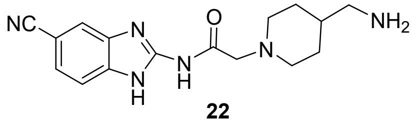
NC
N
O
N
NH2
H
H

22

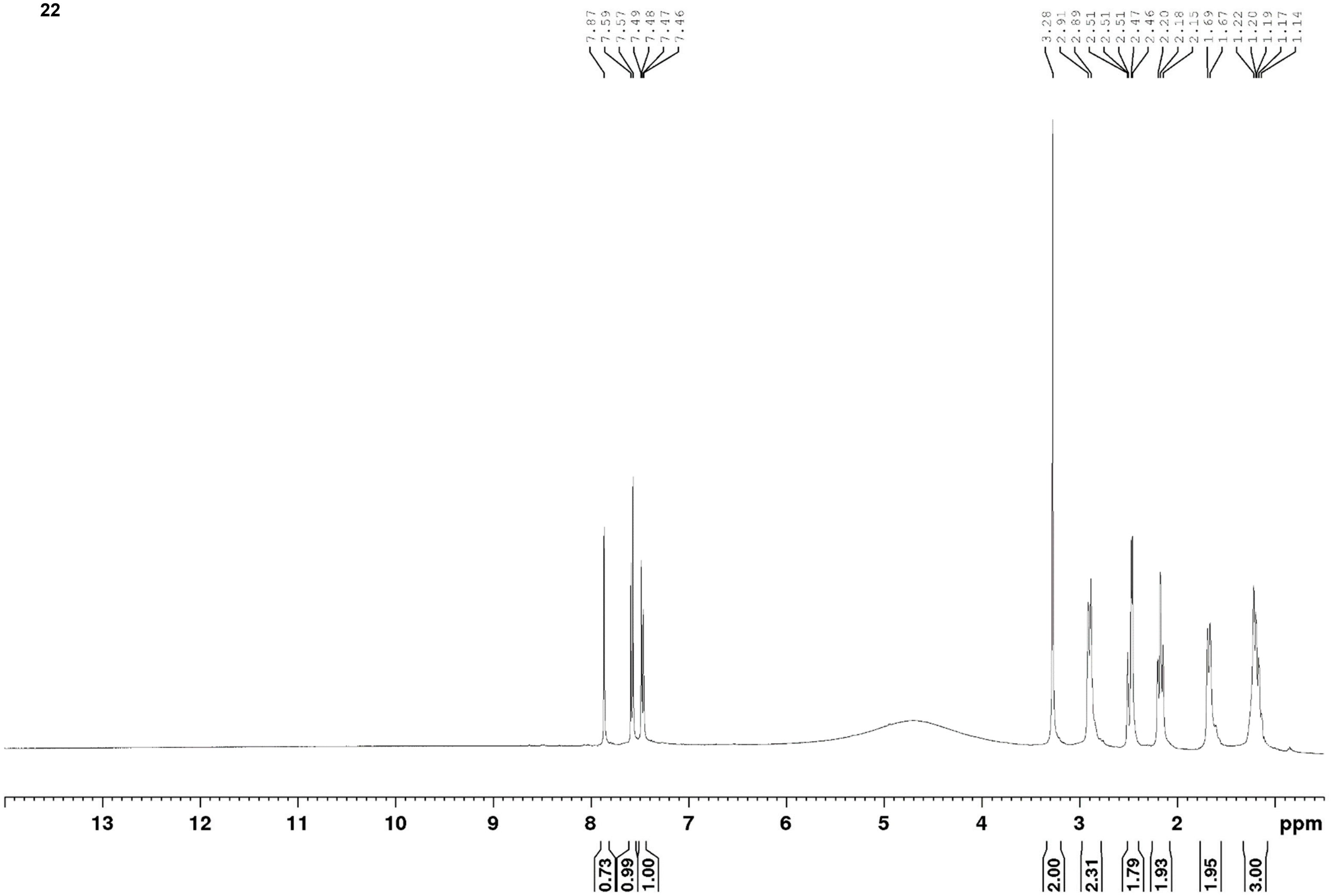
7.87
7.59
7.57
7.49
7.48
7.47
7.46
3.28
2.91
2.89
2.51
2.51
2.51
2.47
2.46
2.20
2.18
2.15
1.69
1.67
1.22
1.20
1.19
1.17
1.14
13
12
11
10
9
8
7
6
5
4
3
2
ppm
0.73
0.99
1.00
2.00
2.31
1.79
1.93
1.95
3.00

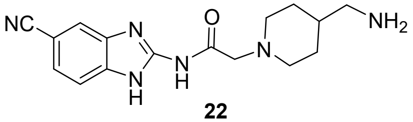

22

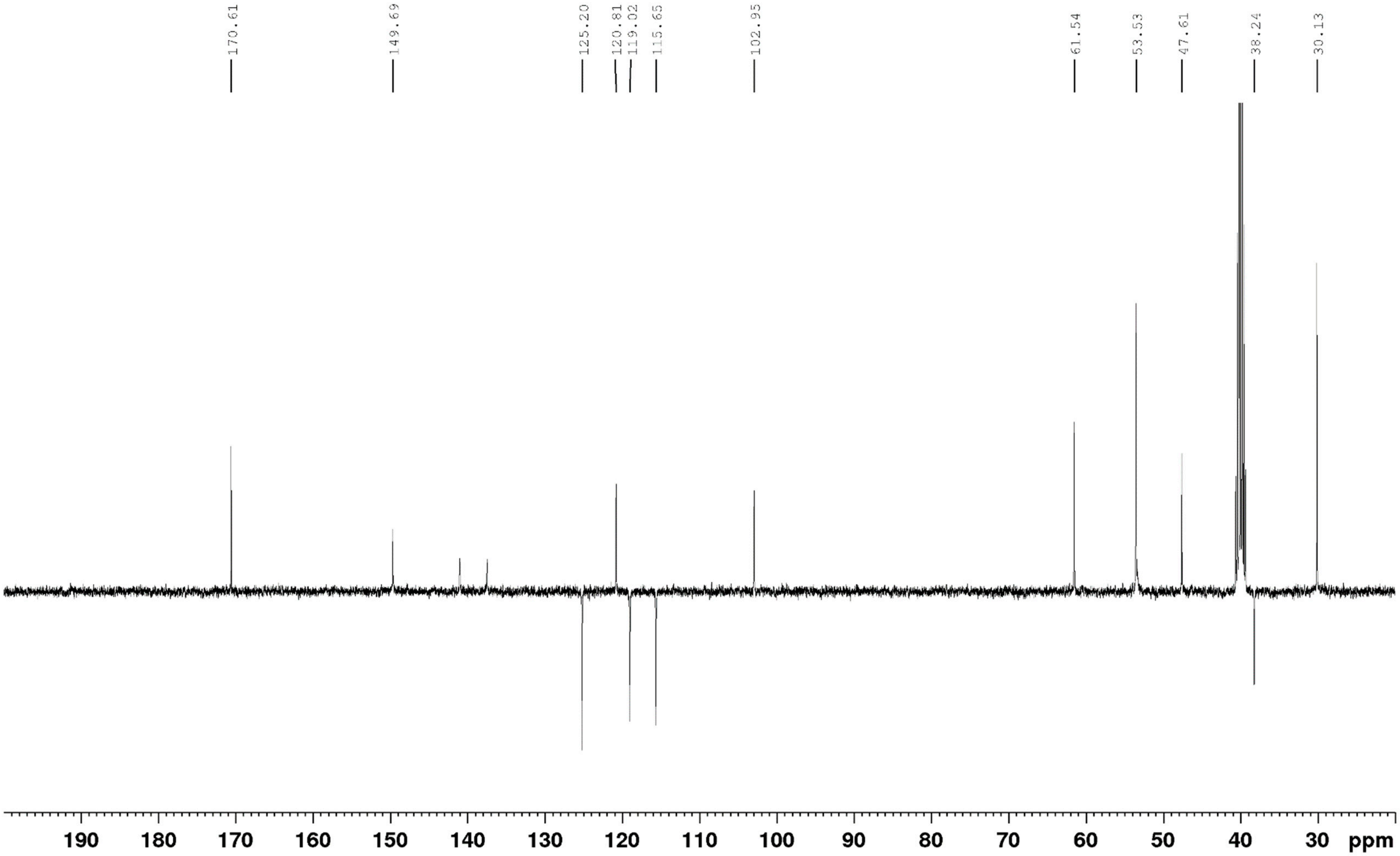
170.61
149.69
125.20
120.81
119.02
115.65
102.95
61.54
53.53
47.61
38.24
30.13
190 180 170 160 150 140 130 120 110 100 90 80 70 60 50 40 30 ppm

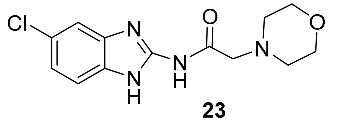

23

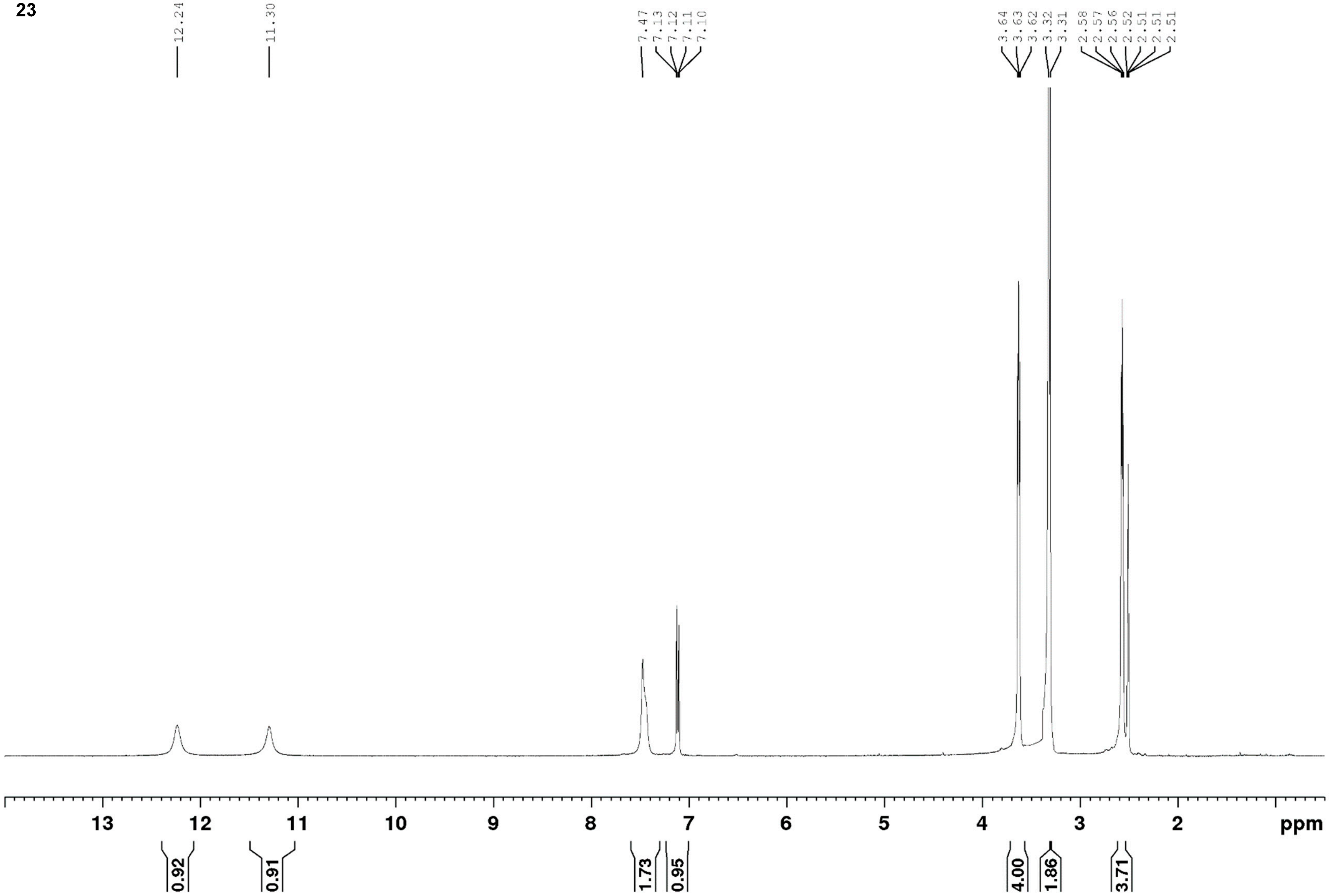
12.24
11.30
7.47
7.13
7.12
7.11
7.10
3.64
3.63
3.62
3.32
3.31
2.58
2.57
2.56
2.52
2.51
2.51
2.51
13
12
11
10
9
8
7
6
5
4
3
2
ppm
0.92
0.91
1.73
0.95
4.00
1.86
3.71

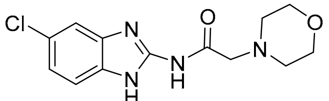

23

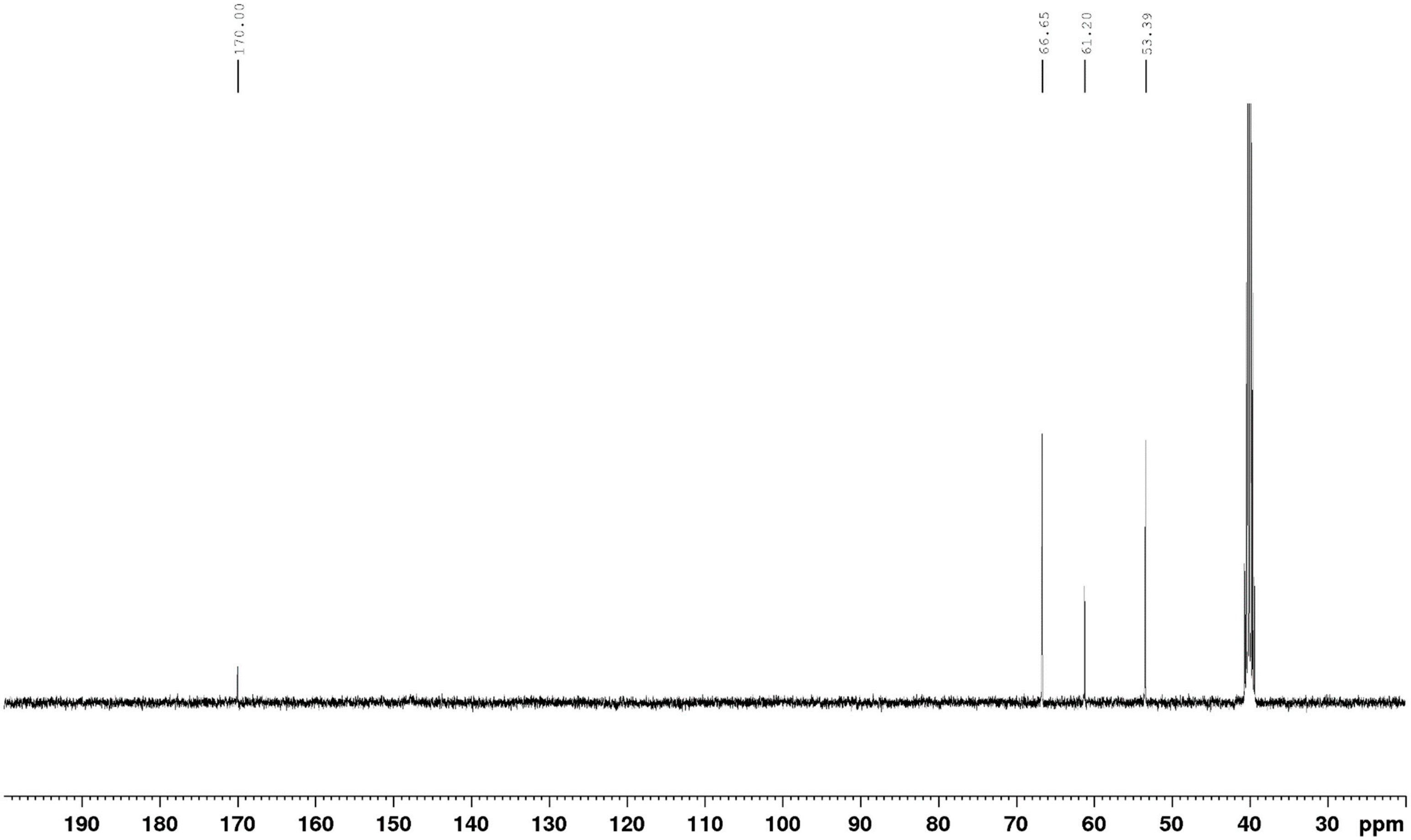

170.00
66.65
61.20
53.39
190
180
170
160
150
140
130
120
110
100
90
80
70
60
50
40
30
ppm

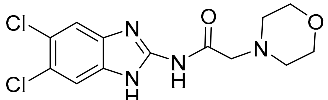

24

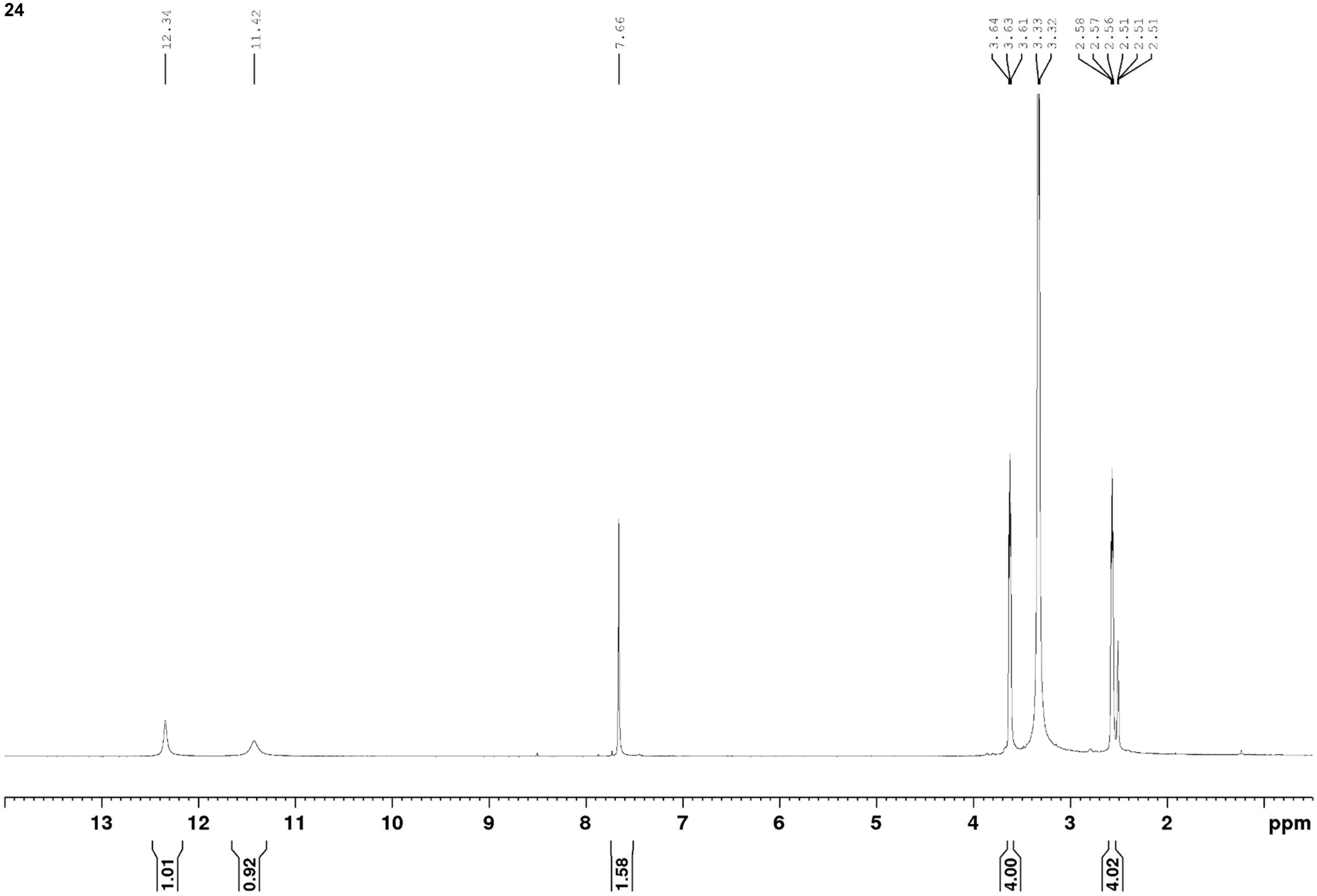
12.34
11.42
7.66
3.64
3.63
3.61
3.33
3.32
2.58
2.57
2.56
2.51
2.51
2.51
13
12
11
10
9
8
7
6
5
4
3
2
ppm
1.01
0.92
1.58
4.00
4.02

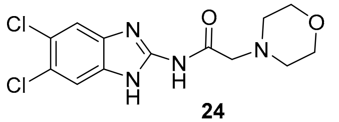

24

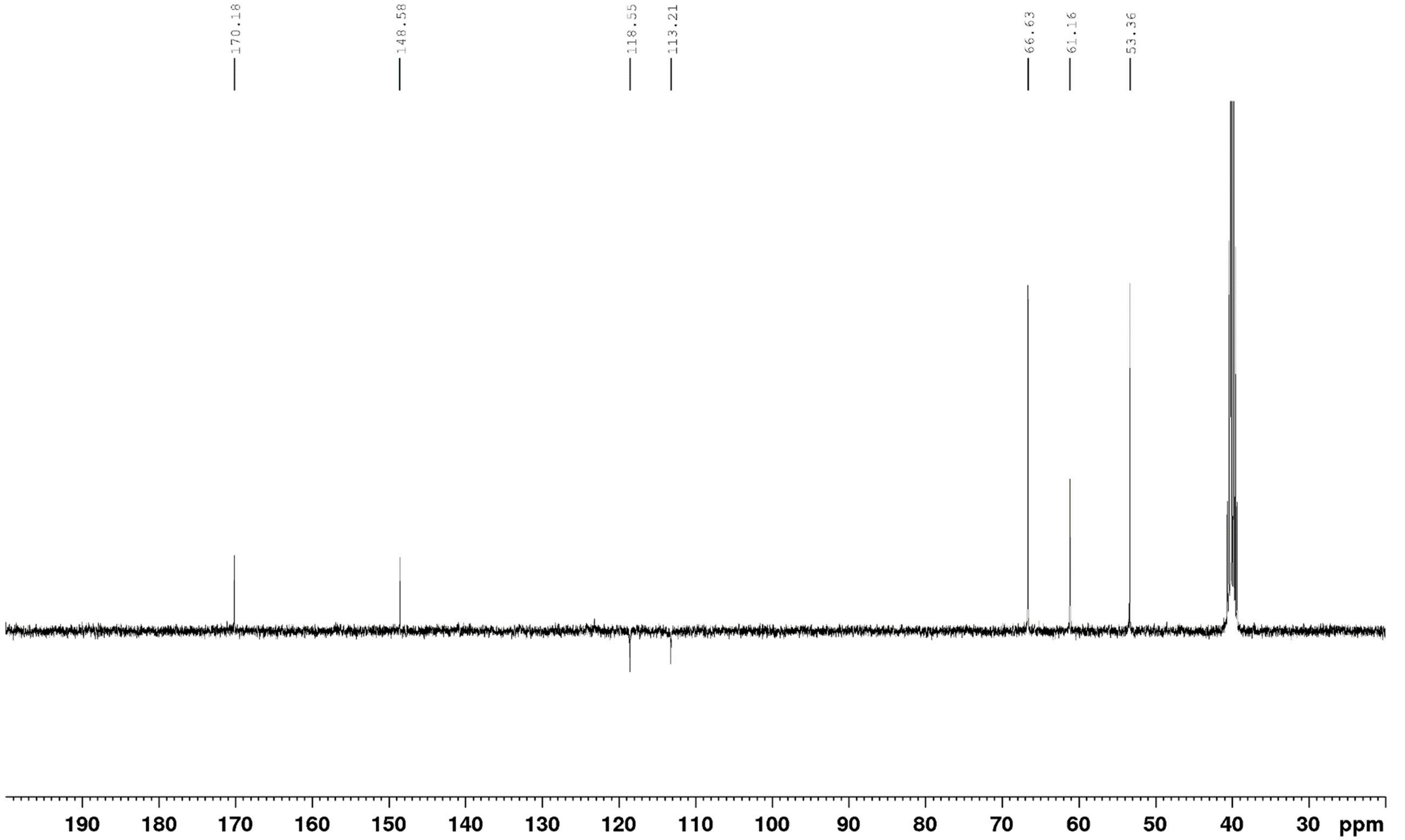

170.18
148.58
118.55
113.21
66.63
61.16
53.36
190
180
170
160
150
140
130
120
110
100
90
80
70
60
50
40
30
ppm

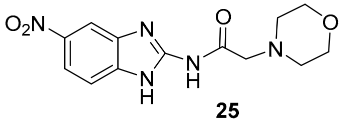

25

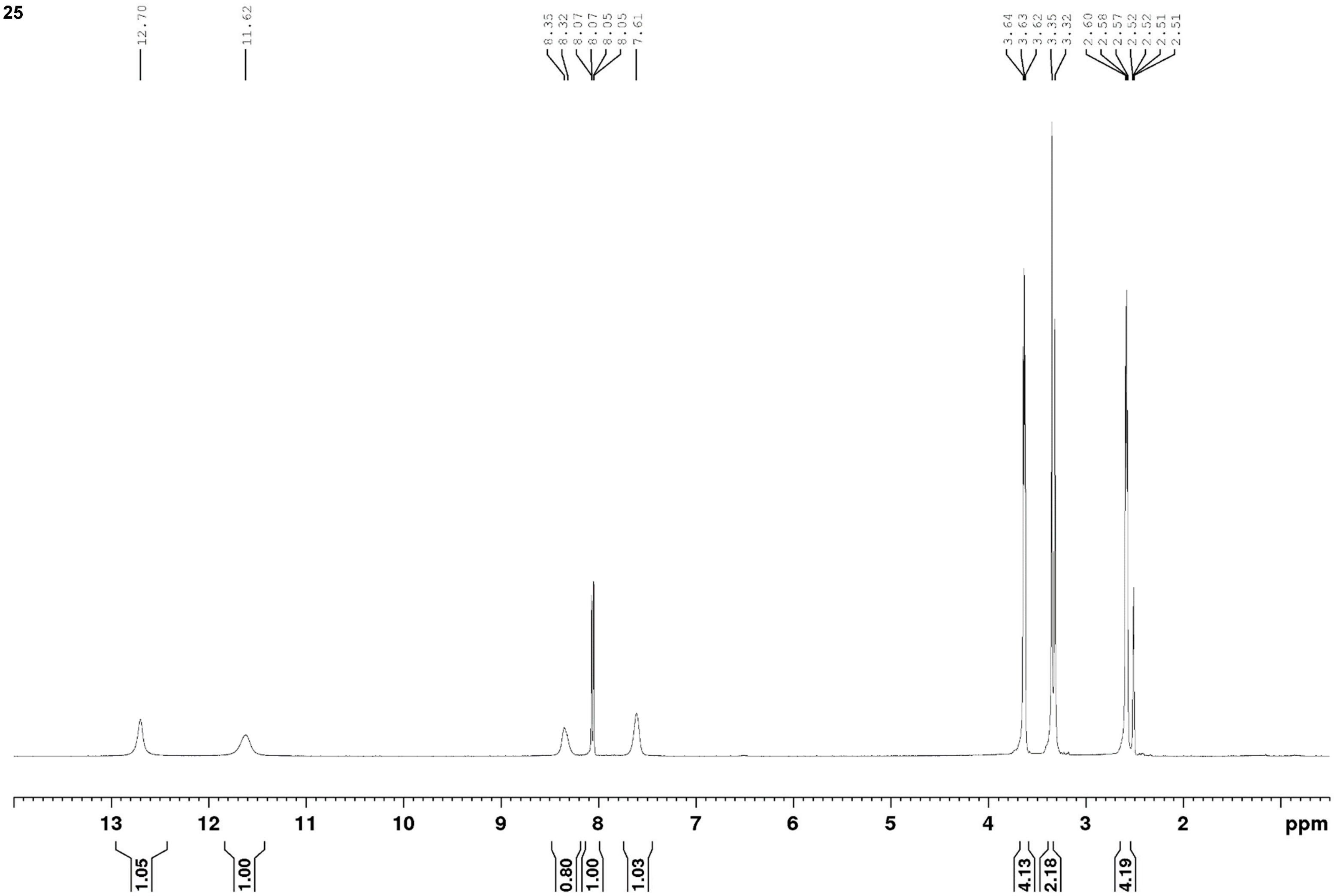

12.70
11.62
8.35
8.32
8.07
8.07
8.05
8.05
7.61
3.64
3.63
3.62
3.35
3.32
2.60
2.58
2.57
2.52
2.52
2.51
2.51
13
12
11
10
9
8
7
6
5
4
3
2
ppm
1.05
1.00
0.80
1.00
1.03
4.13
2.18
4.19

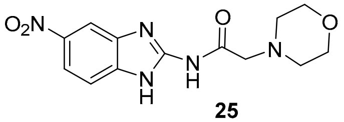
O2N
N
N
H
N
H
O
N
O

25

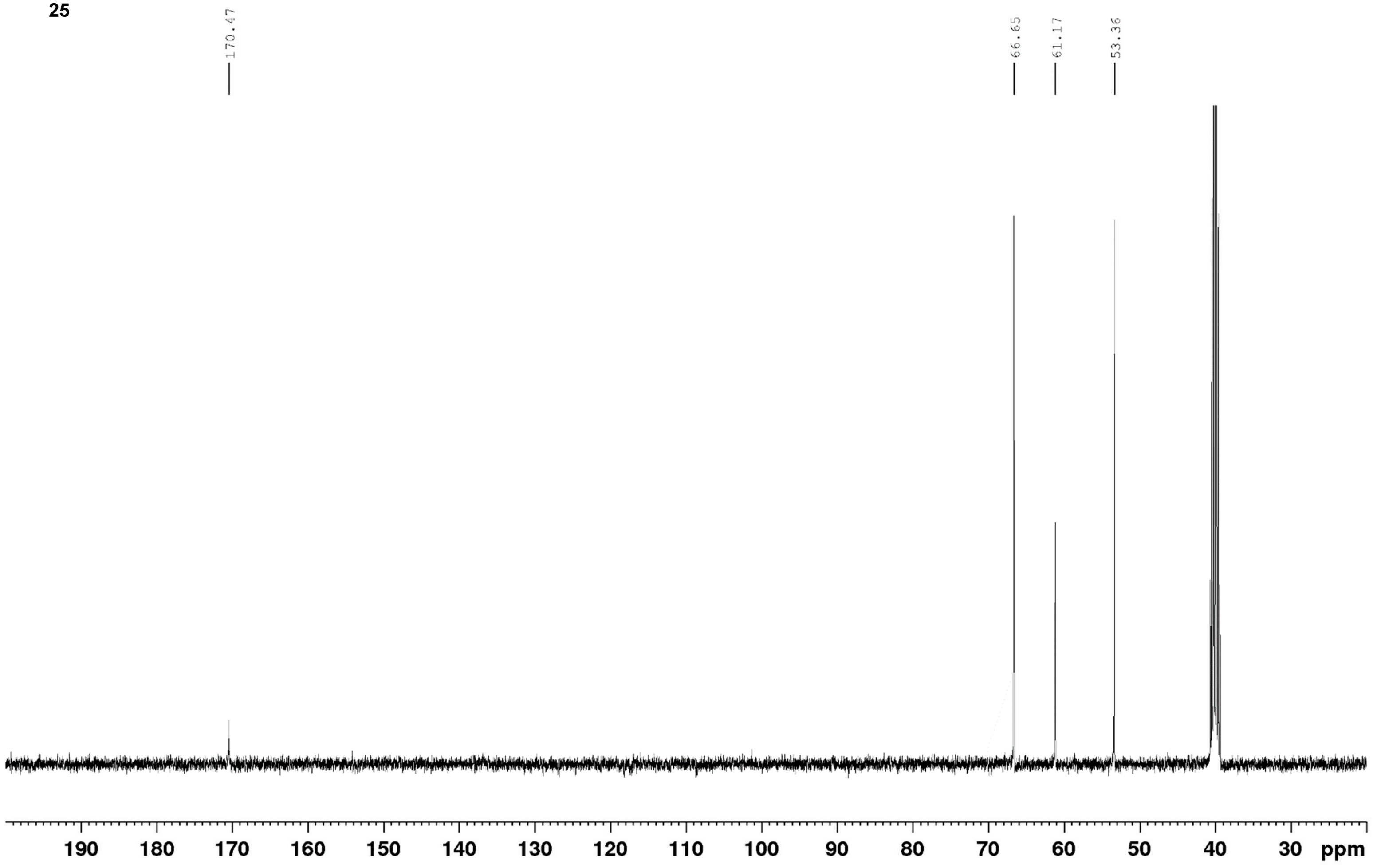
170.47
66.65
61.17
53.36
190
180
170
160
150
140
130
120
110
100
90
80
70
60
50
40
30
ppm

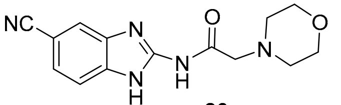
NC
N
N
H
O
N
H
N
O

26

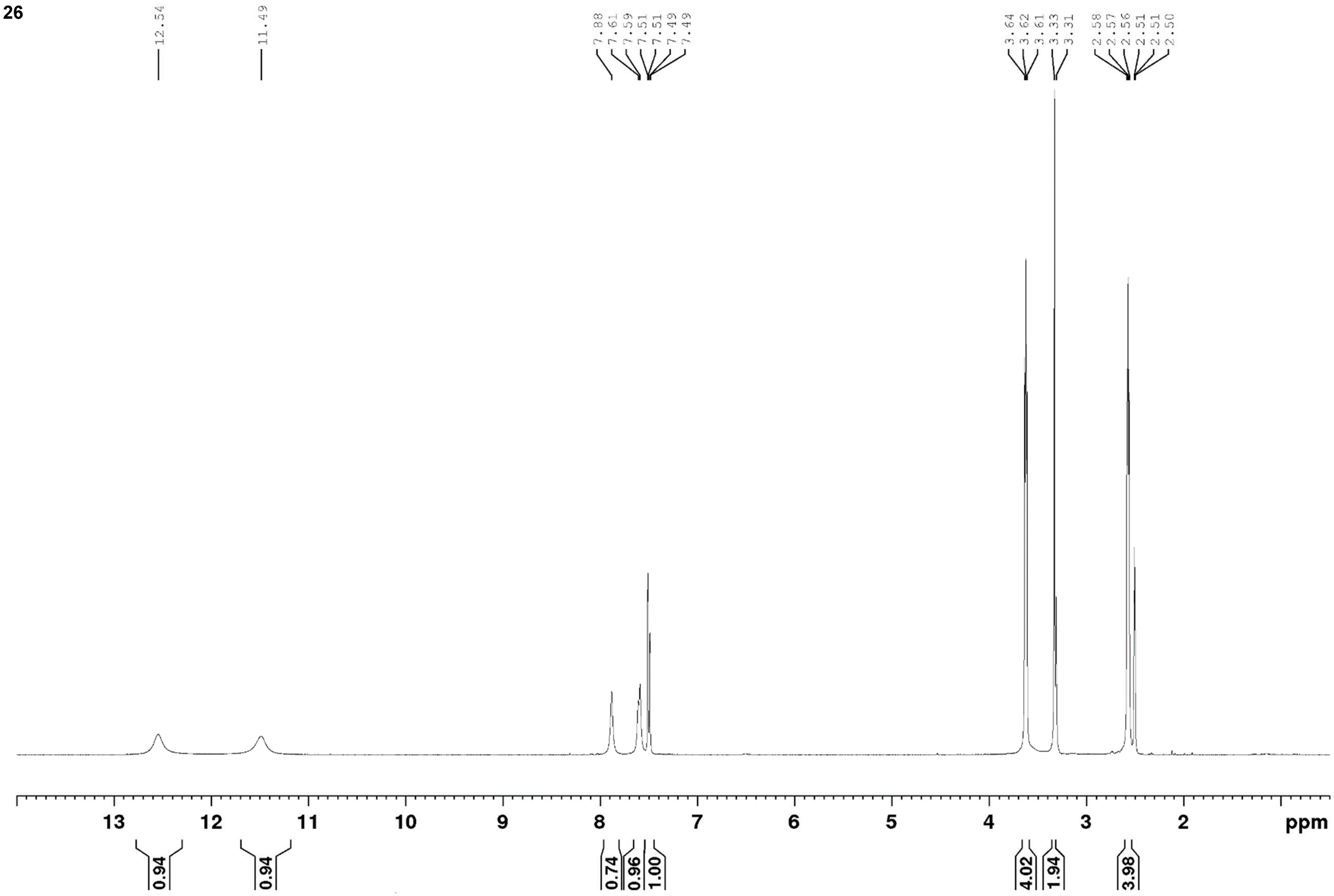
12.54
11.49
7.88
7.61
7.59
7.51
7.51
7.49
7.49
3.64
3.62
3.61
3.33
3.31
2.58
2.57
2.56
2.51
2.51
2.50
13
12
11
10
9
8
7
6
5
4
3
2
ppm
0.94
0.94
0.74
0.96
1.00
4.02
1.94
3.98

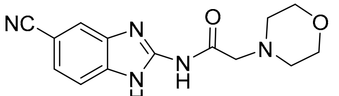

26

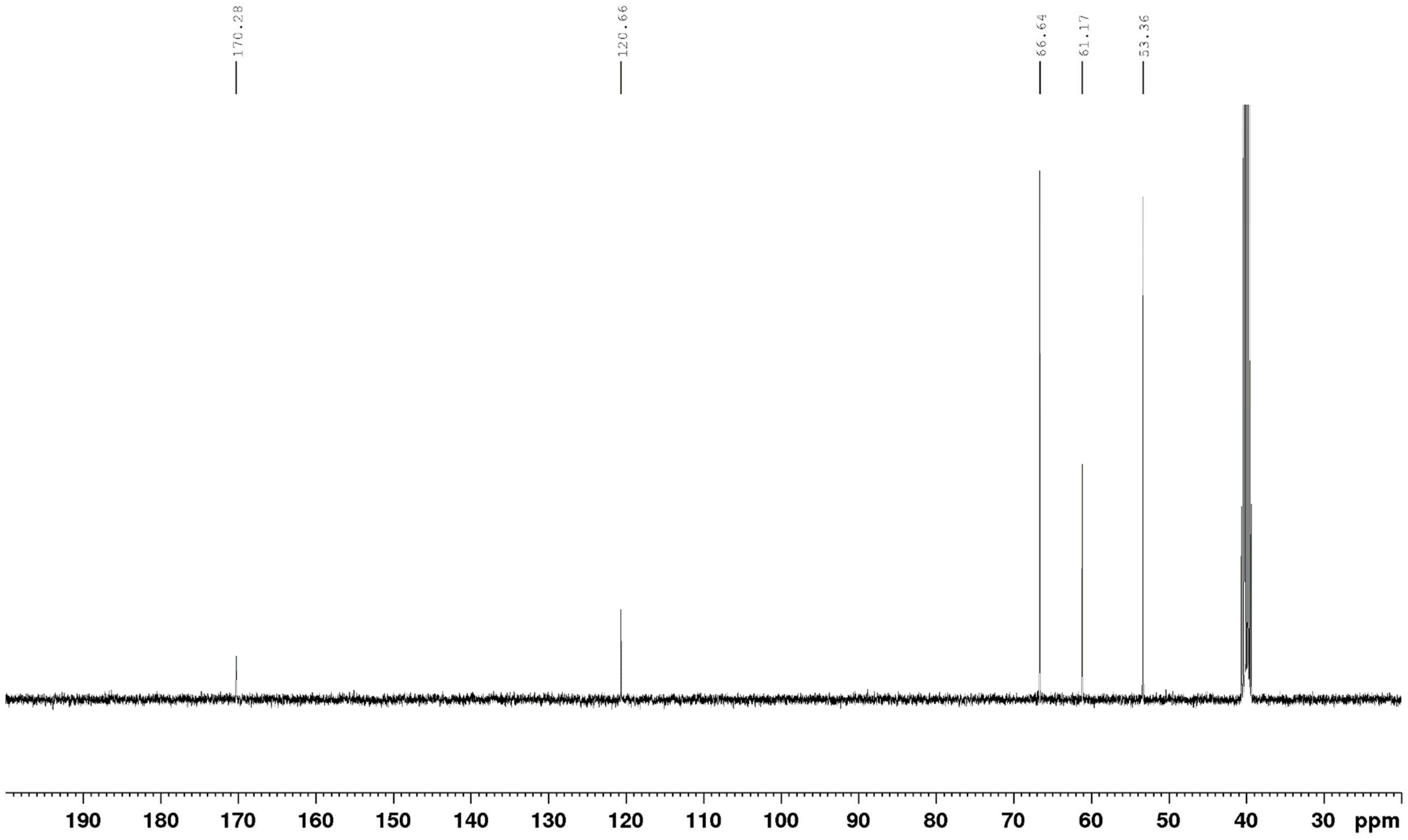

170.28
120.66
66.64
61.17
53.36
190
180
170
160
150
140
130
120
110
100
90
80
70
60
50
40
30
ppm

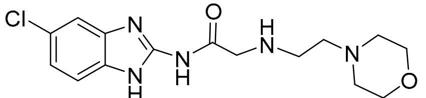

27

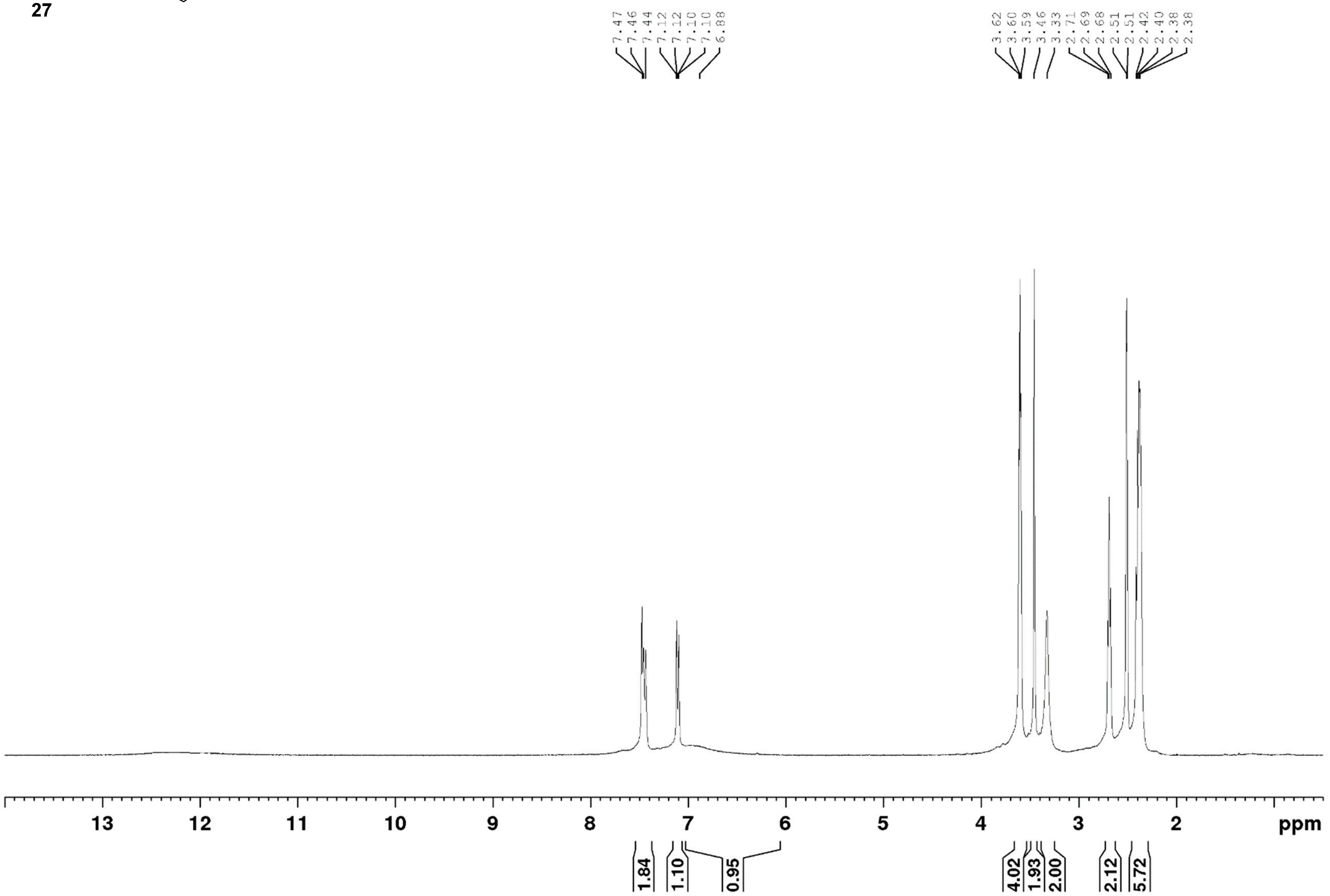
7.47
7.46
7.44
7.12
7.12
7.10
7.10
6.88
3.62
3.60
3.59
3.46
3.33
2.71
2.69
2.68
2.51
2.51
2.42
2.40
2.38
2.38
13
12
11
10
9
8
7
6
5
4
3
2
ppm
1.84
1.10
0.95
4.02
1.93
2.00
2.12
5.72

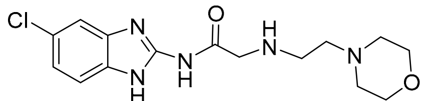

27

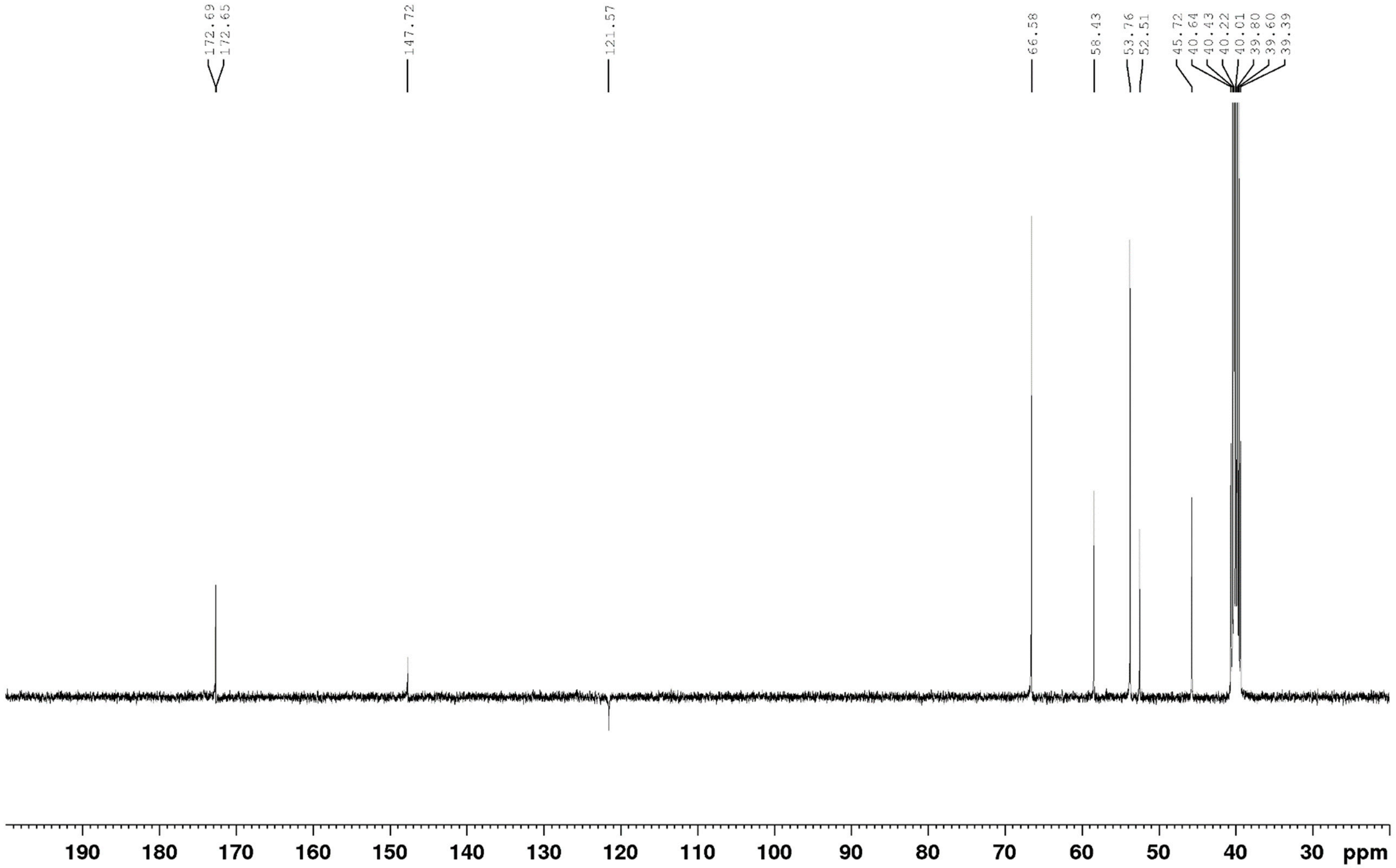

172.69
172.65
147.72
121.57
66.58
58.43
53.76
52.51
45.72
40.64
40.43
40.22
40.01
39.80
39.60
39.39
190
180
170
160
150
140
130
120
110
100
90
80
70
60
50
40
30
ppm

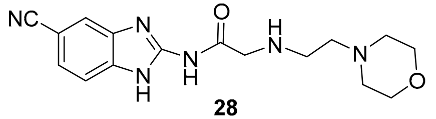
NC
N
O
H
N
N
N
H
N
H
O

28

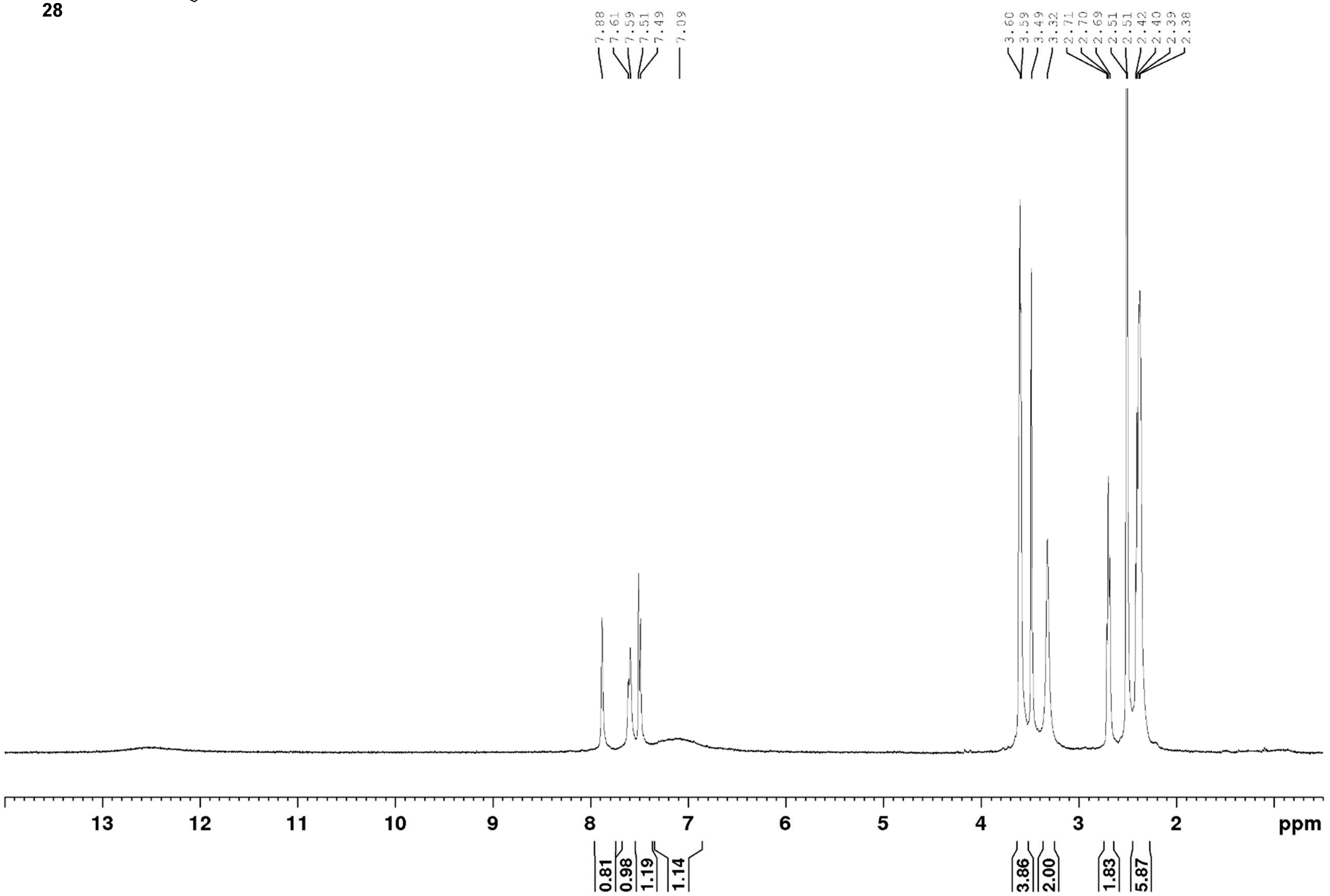
7.88
7.61
7.59
7.51
7.49
7.09
3.60
3.59
3.49
3.32
2.71
2.70
2.69
2.51
2.51
2.42
2.40
2.39
2.38
13 12 11 10 9 8 7 6 5 4 3 2 ppm
0.81
0.98
1.19
1.14
3.86
2.00
1.83
5.87

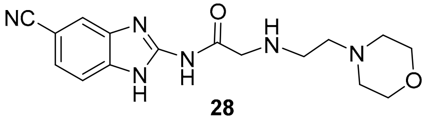
NC
N
N
H
N
H
O
H
N
N
O

28

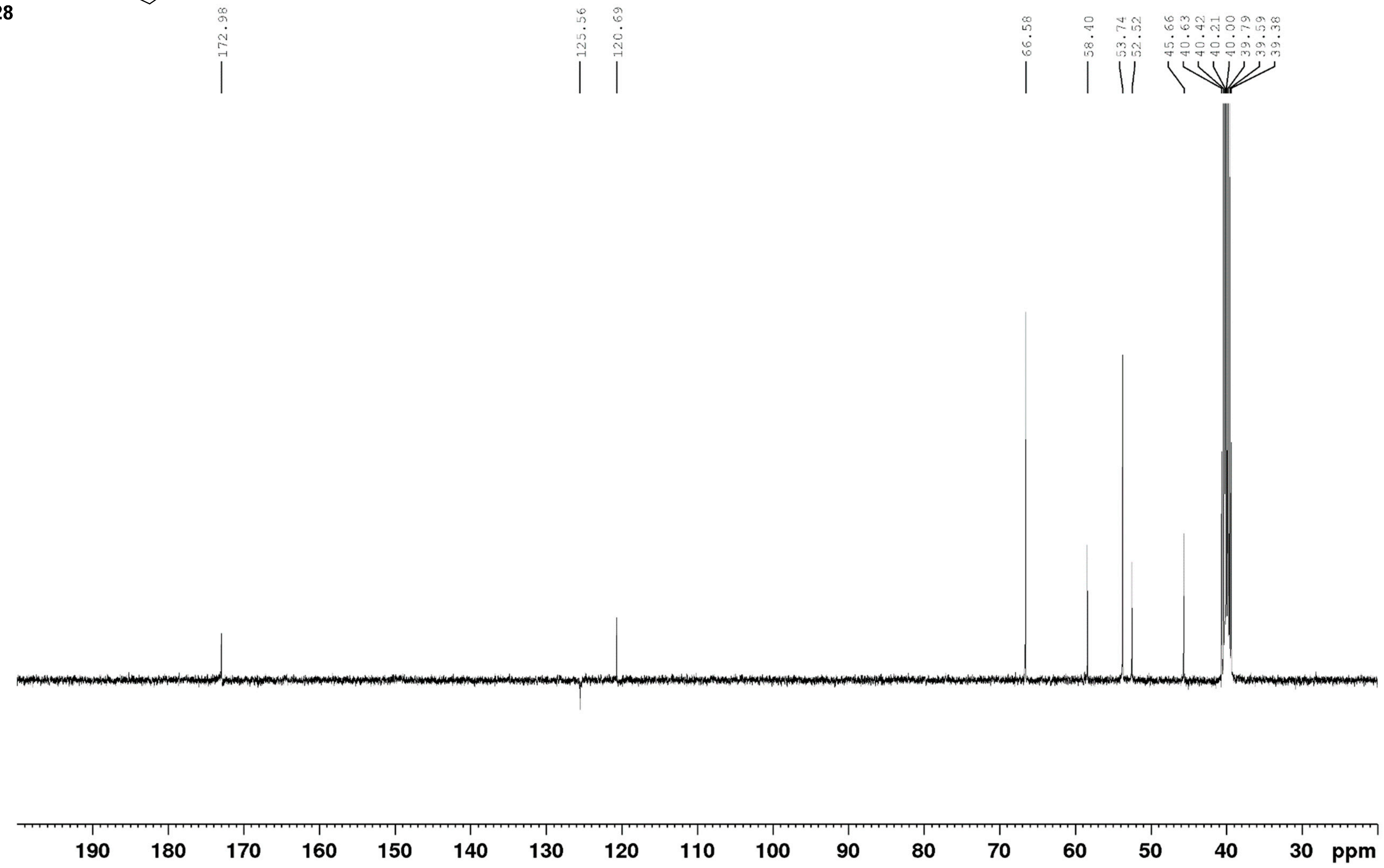
172.98
125.56
120.69
66.58
58.40
53.74
52.52
45.66
40.63
40.42
40.21
40.00
39.79
39.59
39.38
190 180 170 160 150 140 130 120 110 100 90 80 70 60 50 40 30 ppm

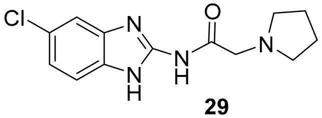

29

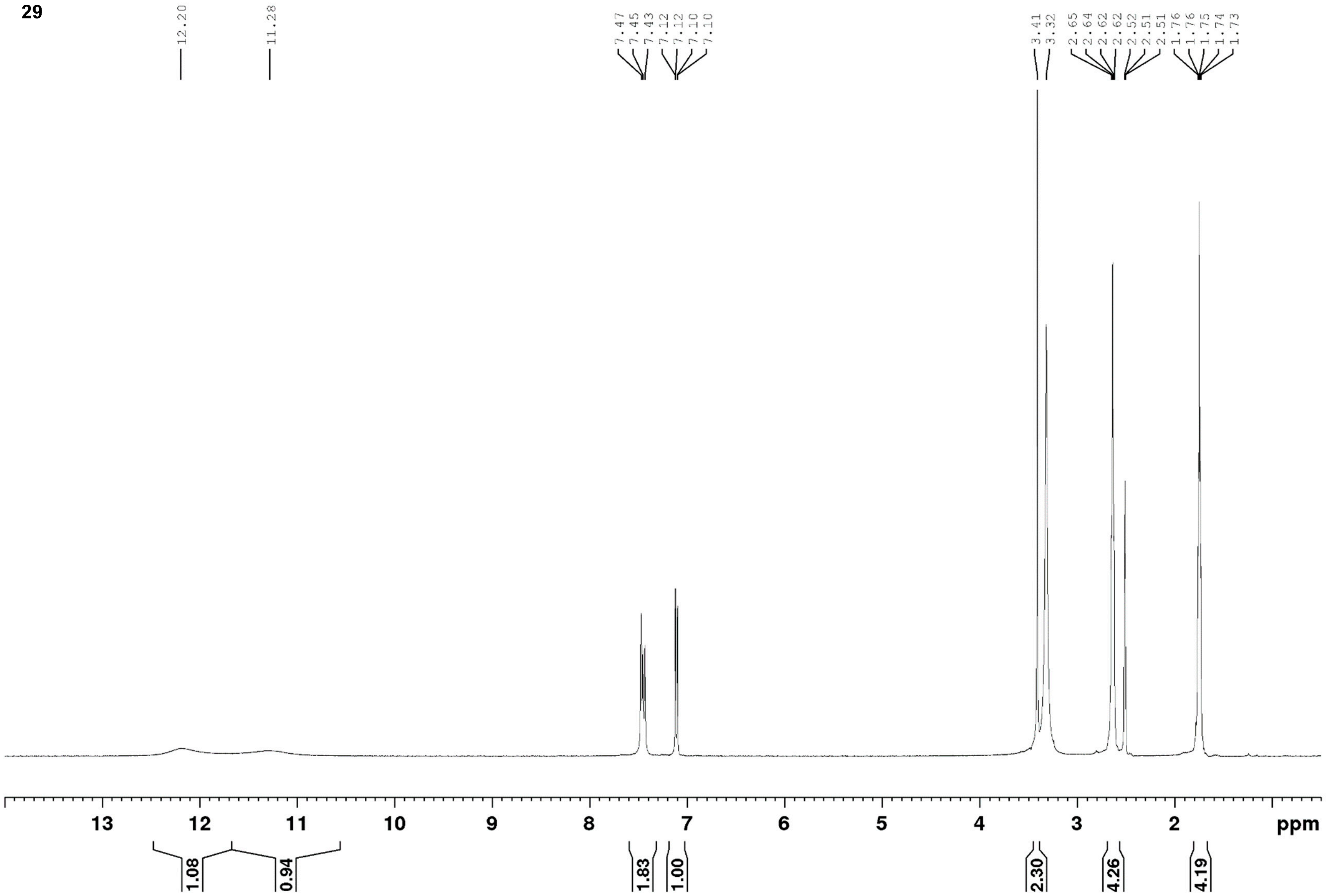

12.20
11.28
7.47
7.45
7.43
7.12
7.12
7.10
7.10
3.41
3.32
2.65
2.64
2.62
2.62
2.52
2.51
2.51
1.76
1.76
1.75
1.74
1.73
13
12
11
10
9
8
7
6
5
4
3
2
ppm
1.08
0.94
1.83
1.00
2.30
4.26
4.19

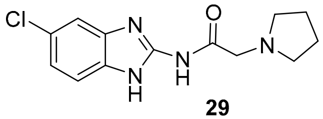

29

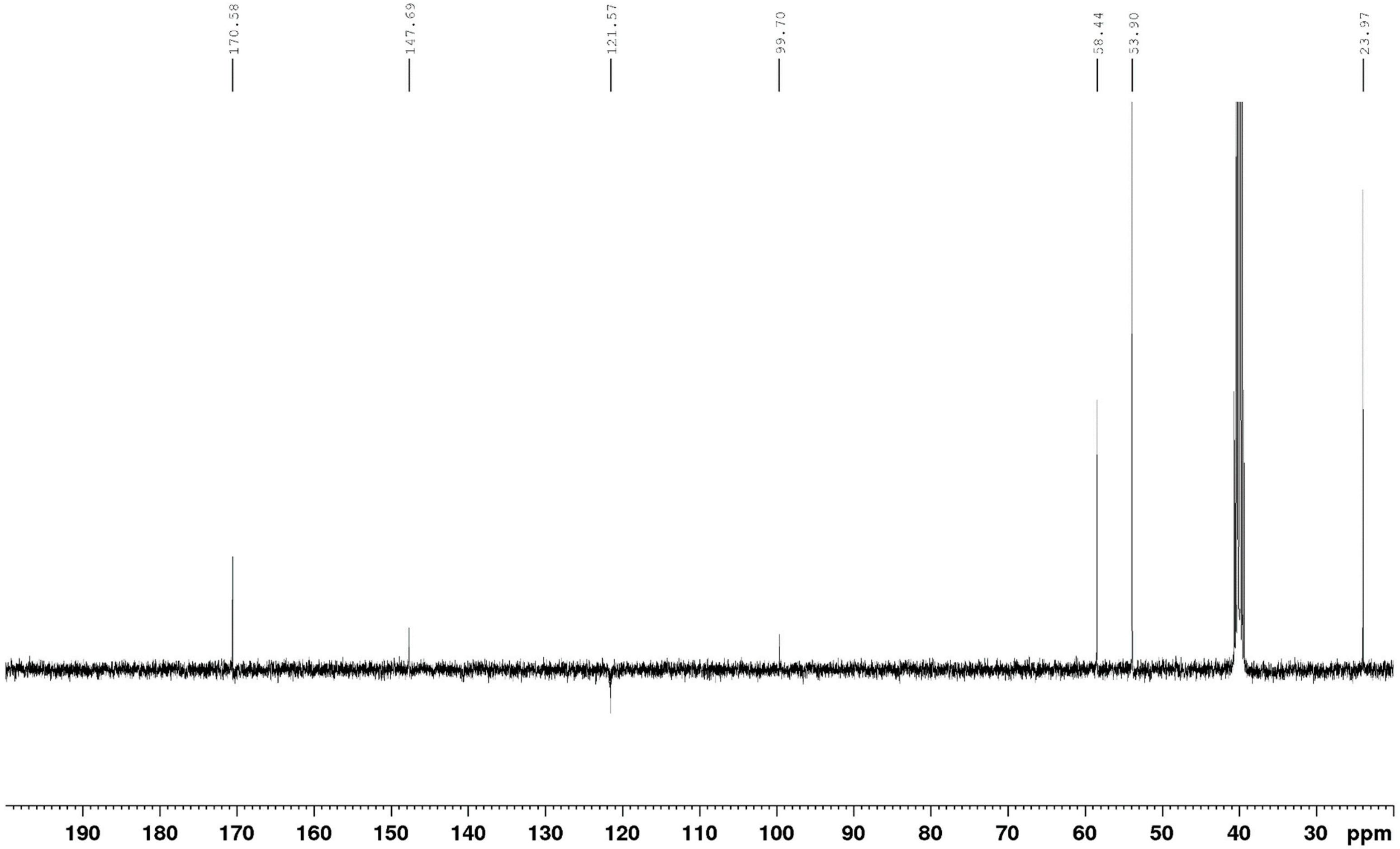

170.58
147.69
121.57
99.70
58.44
53.90
23.97
190
180
170
160
150
140
130
120
110
100
90
80
70
60
50
40
30
ppm

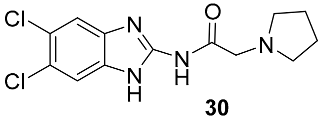

30

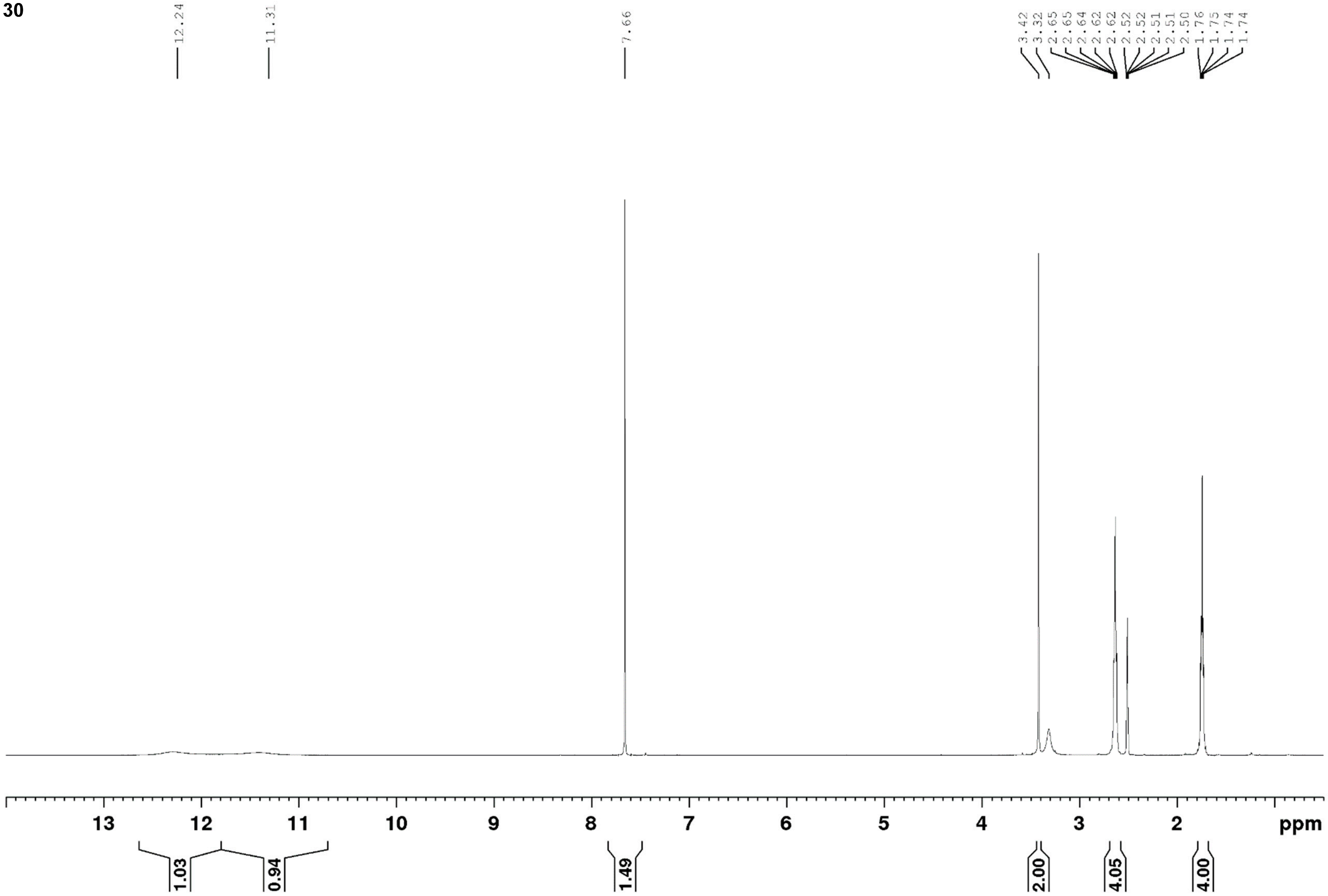
12.24
11.31
7.66
3.42
3.32
2.65
2.65
2.64
2.62
2.62
2.52
2.52
2.51
2.51
2.50
1.76
1.75
1.74
1.74
13
12
11
10
9
8
7
6
5
4
3
2
ppm
1.03
0.94
1.49
2.00
4.05
4.00

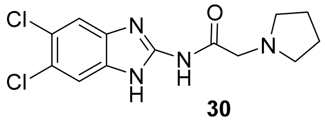
Cl
Cl
N
H
N
H
O
N

30

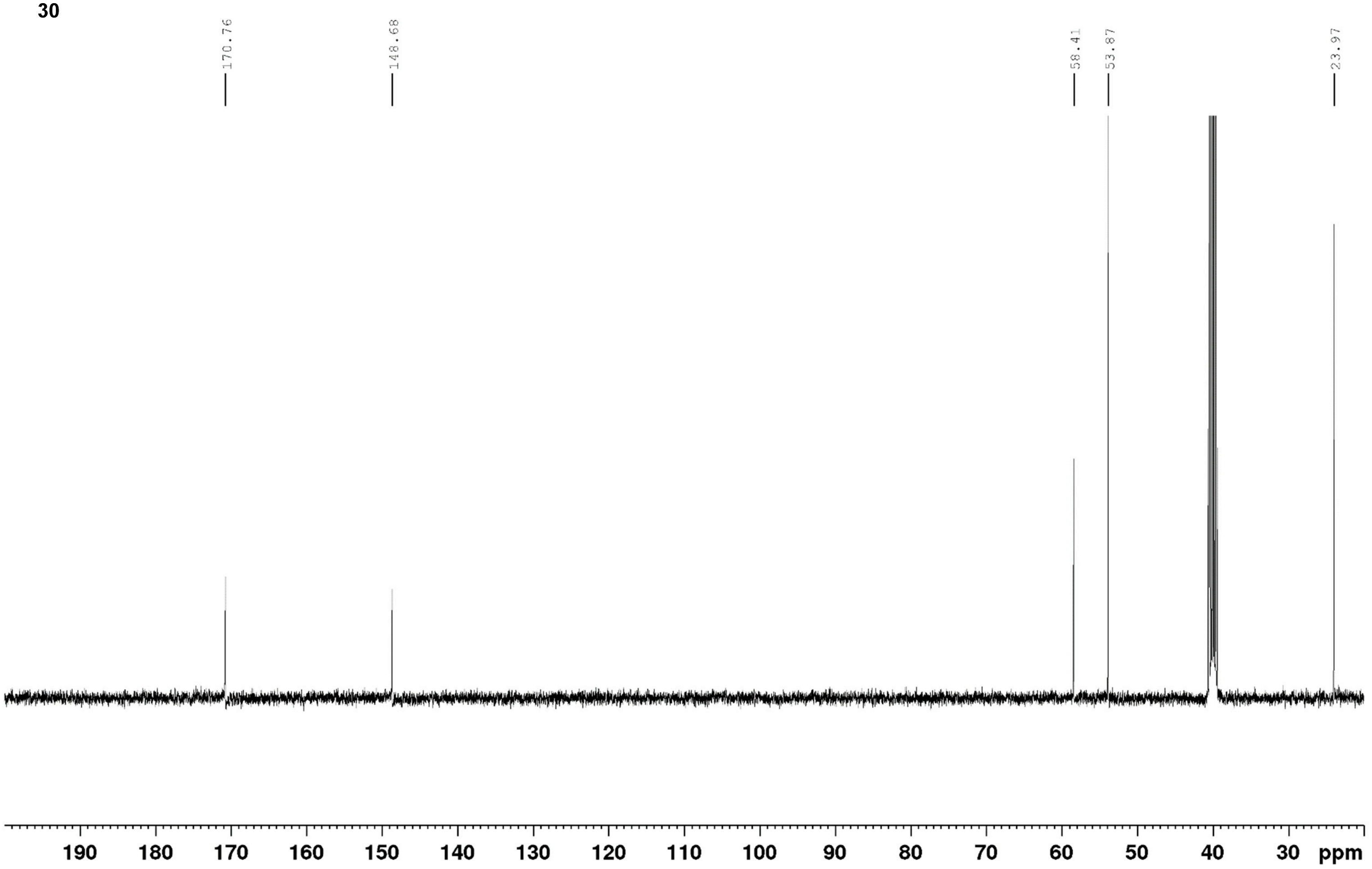
170.76
148.68
58.41
53.87
23.97
190
180
170
160
150
140
130
120
110
100
90
80
70
60
50
40
30
ppm

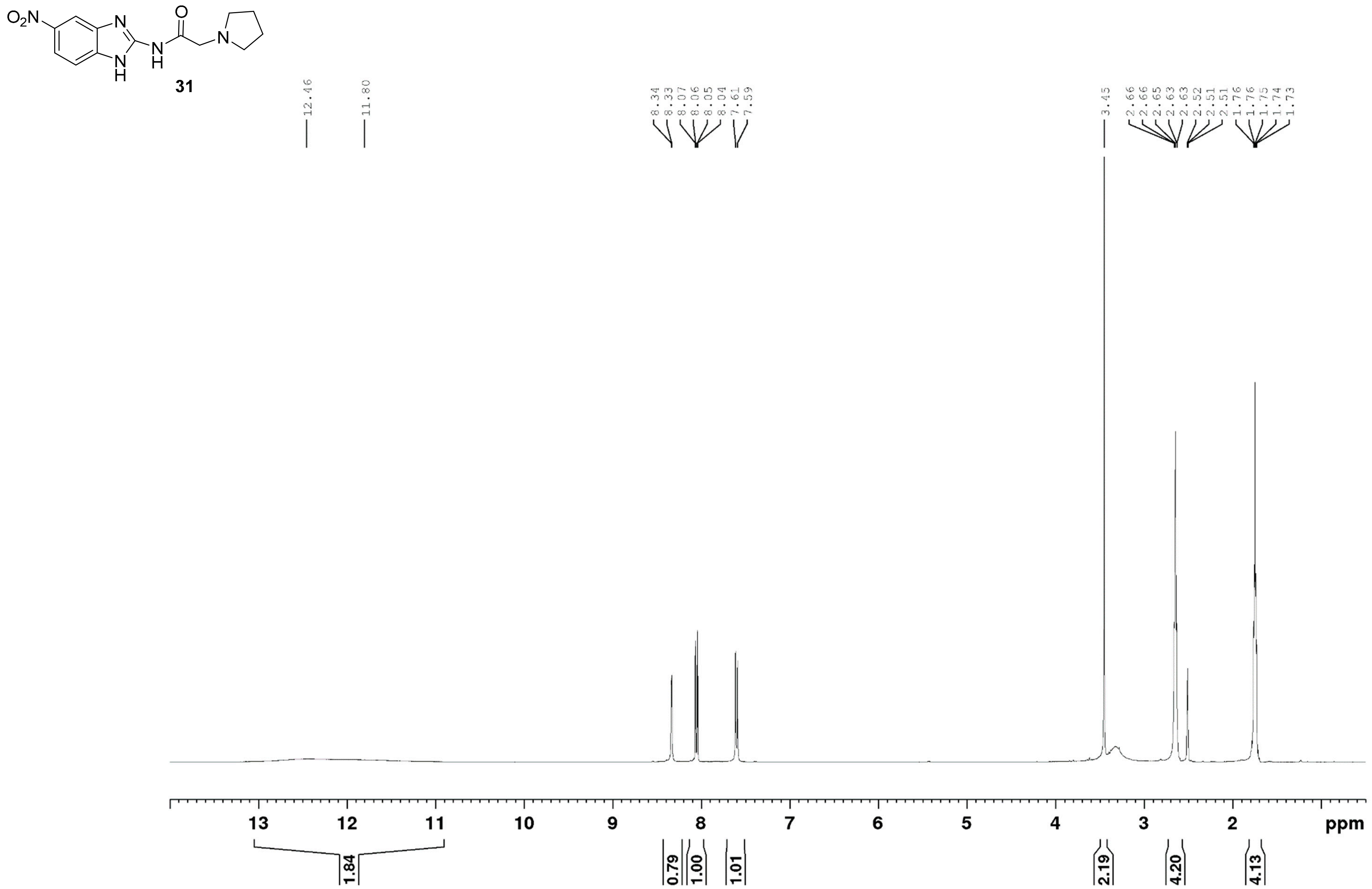
12.46
11.80
8.34
8.33
8.07
8.06
8.05
8.04
7.61
7.59
3.45
2.66
2.66
2.65
2.63
2.63
2.52
2.51
2.51
1.76
1.76
1.75
1.74
1.73
13
12
11
10
9
8
7
6
5
4
3
2
ppm
1.84
0.79
1.00
1.01
2.19
4.20
4.13

31

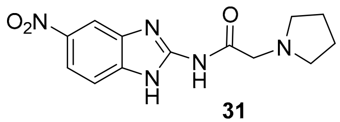

31

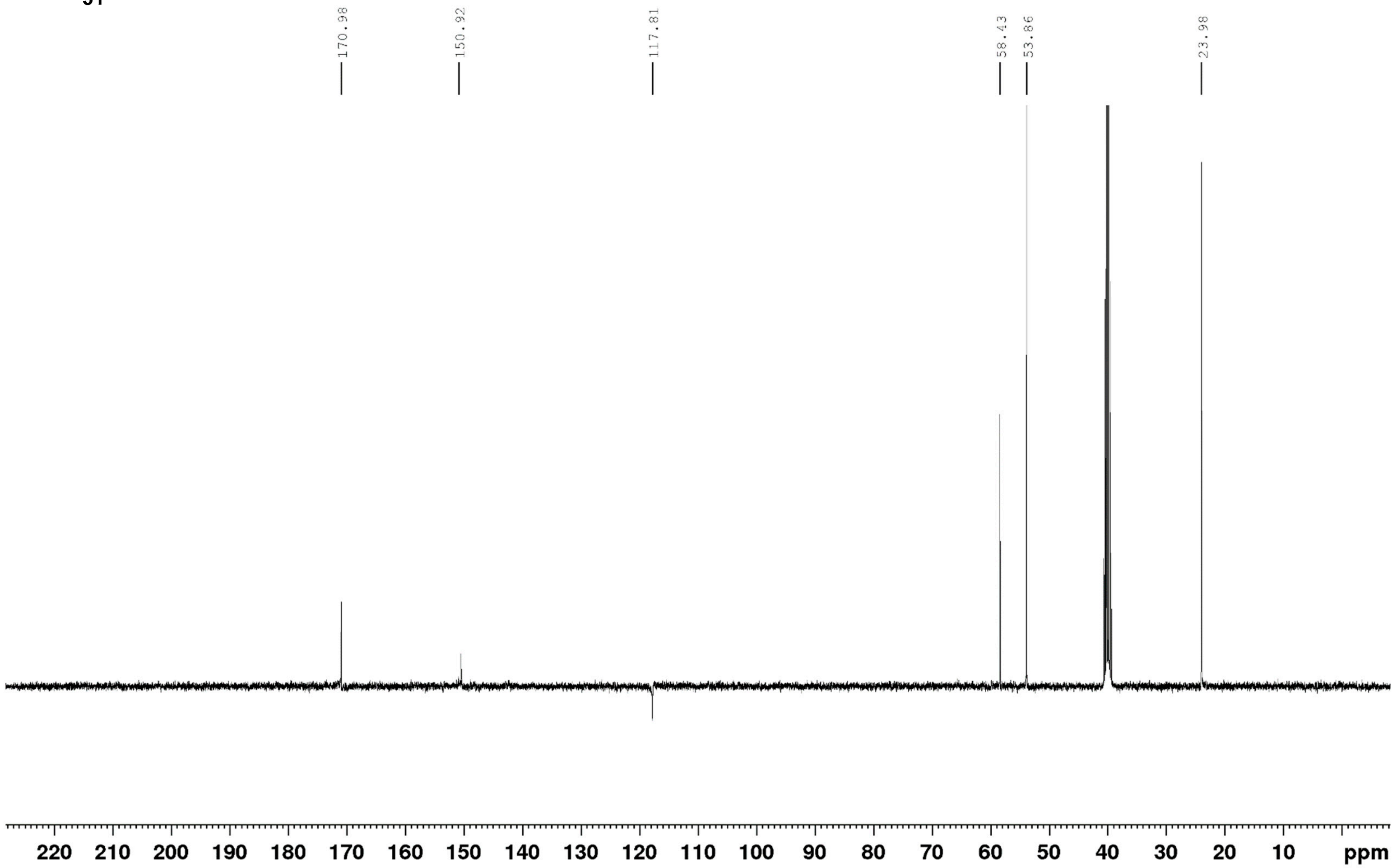

170.98
150.92
117.81
58.43
53.86
23.98
220 210 200 190 180 170 160 150 140 130 120 110 100 90 80 70 60 50 40 30 20 10 ppm

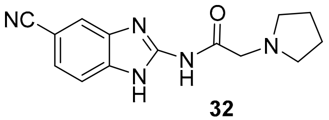
NC
N
O
N
N
H
H

32

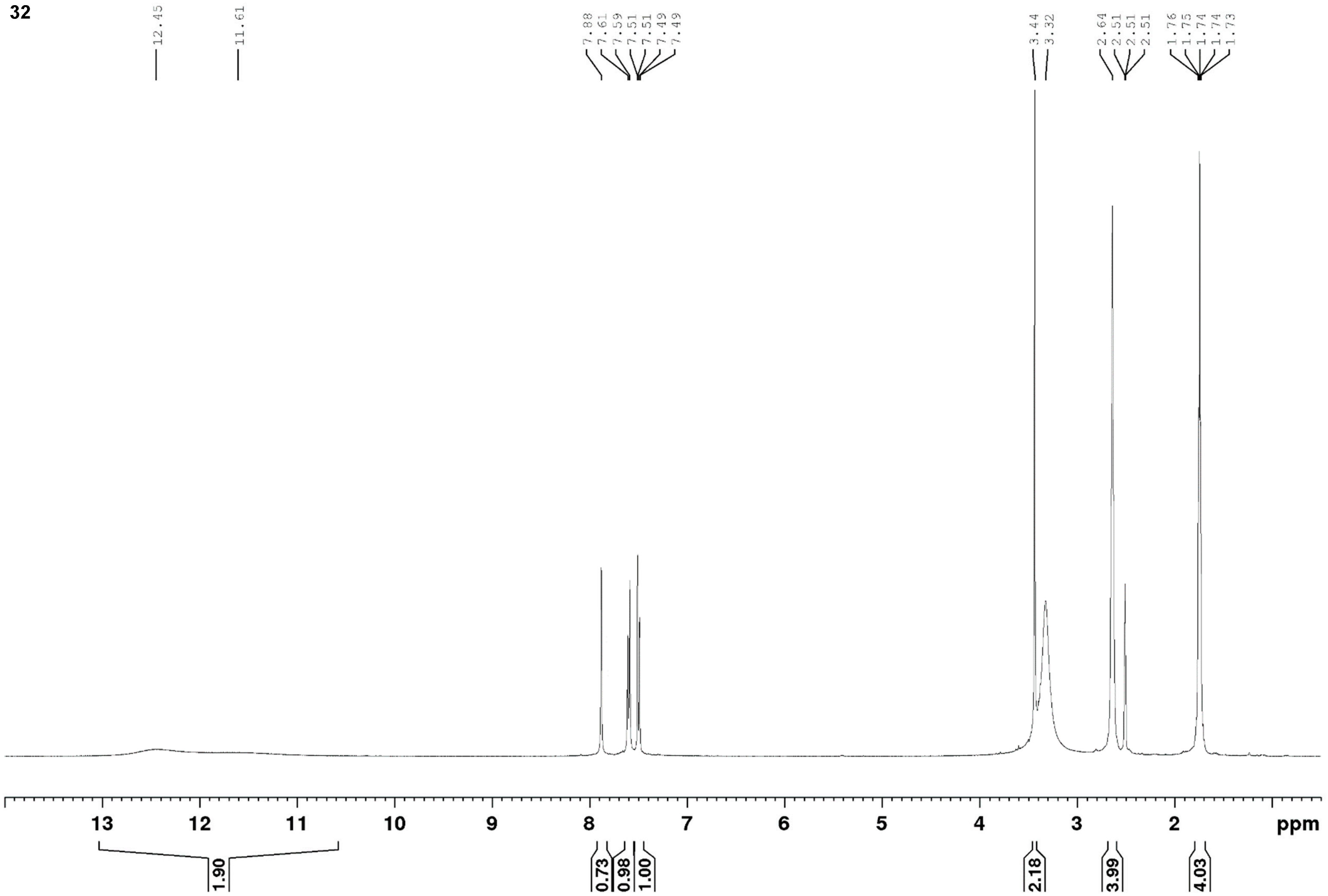
12.45
11.61
7.88
7.61
7.59
7.51
7.51
7.51
7.49
7.49
3.44
3.32
2.64
2.51
2.51
2.51
1.76
1.75
1.74
1.74
1.73
13 12 11 10 9 8 7 6 5 4 3 2 ppm
1.90
0.73
0.98
1.00
2.18
3.99
4.03

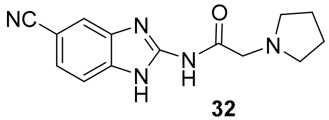
NC
N
N
H
N
H
O
N

32

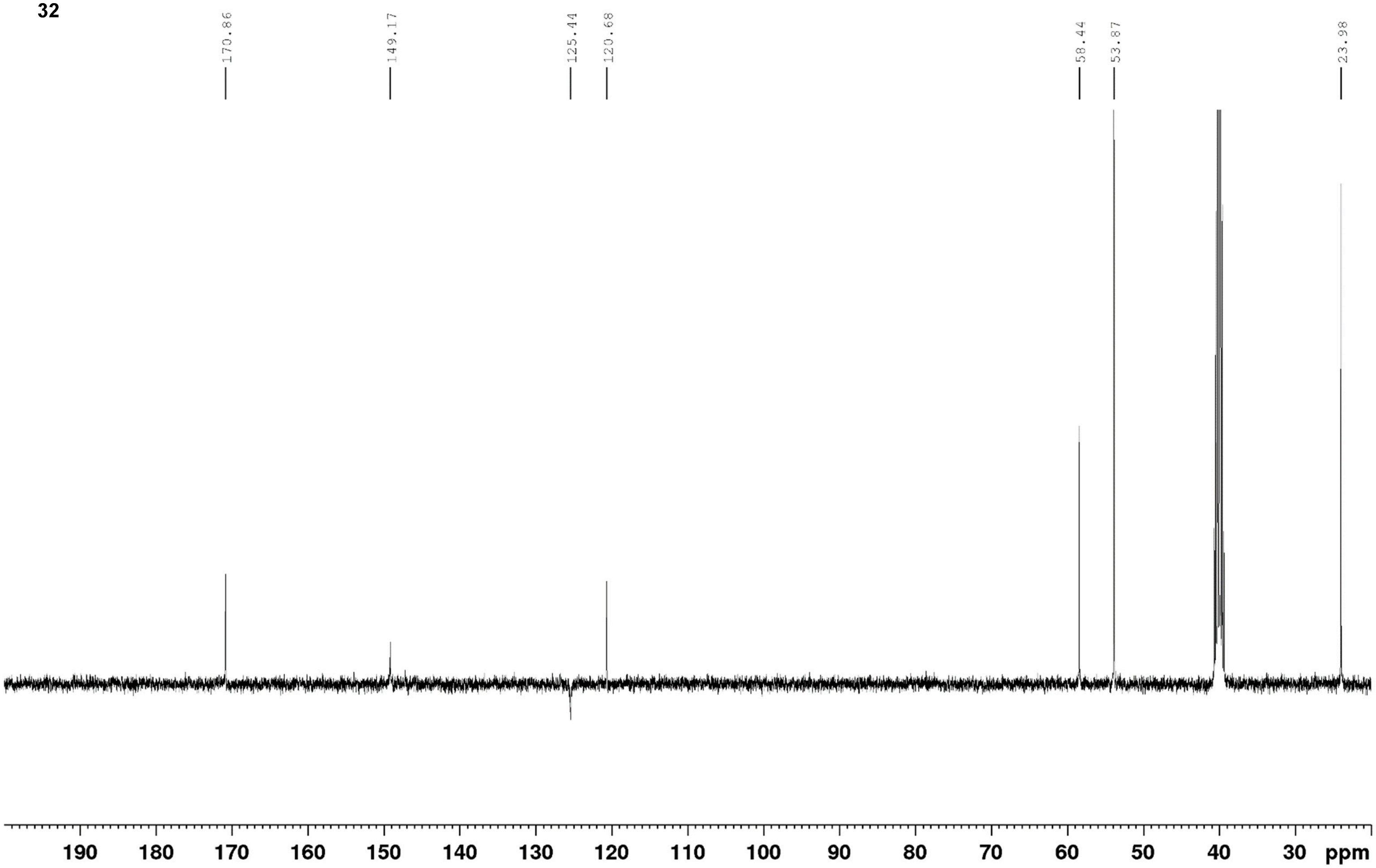
170.86
149.17
125.44
120.68
58.44
53.87
23.98
190
180
170
160
150
140
130
120
110
100
90
80
70
60
50
40
30
ppm

Table S1. Combustion Analysis data of the newly synthesized compounds.

compd	formula	C calcd-found	H calcd-found	N calcd-found
**1**	$C_{13}H_{17}N_5O$	60.21-60.37	6.61-6.63	27.01-27.08
**2**	$C_{13}H_{16}ClN_5O$	53.15-53.27	5.49-5.51	23.84-23.91
**3**	$C_{13}H_{15}Cl_2N_5O$	47.58-47.66	4.61-4.62	21.34-21.41
**4**	$C_{13}H_{16}N_6O_3$	51.31-51.47	5.30-5.32	27.62-27.68
**5**	$C_{14}H_{16}N_6O$	59.14-59.25	5.67-5.68	29.56-29.62
**6**	$C_{14}H_{17}ClN_4O$	57.44-57.57	5.85-5.87	19.14-19.20
**7**	$C_{14}H_{16}Cl_2N_4O$	51.39-51.46	4.93-4.94	17.12-17.16
**8**	$C_{14}H_{17}N_5O_3$	55.44-55.57	5.65-5.67	23.09-23.15
**9**	$C_{15}H_{17}N_5O$	63.59-63.68	6.05-6.06	24.72-24.77
**10**	$C_{14}H_{18}N_4O_2$	61.30-61.44	6.61-6.63	20.42-20.49
**11**	$C_{14}H_{17}ClN_4O_2$	54.46-54.56	5.55-5.56	18.15-18.19
**12**	$C_{14}H_{16}Cl_2N_4O_2$	48.99-49.12	4.70-4.71	16.32-16.36
**13**	$C_{14}H_{17}N_5O_4$	52.66-52.81	5.37-5.38	21.93-21.99
**14**	$C_{15}H_{17}N_5O_2$	60.19-60.33	5.72-5.74	23.40-23.45
**15**	$C_{15}H_{18}ClN_5O_2$	53.65-53.78	5.40-5.41	20.86-20.90
**16**	$C_{15}H_{17}Cl_2N_5O_2$	48.66-48.82	4.63-4.64	18.92-18.97
**17**	$C_{15}H_{18}N_6O_4$	52.02-52.12	5.24-5.26	24.27-24.34
**18**	$C_{16}H_{18}N_6O_2$	58.88-59.03	5.56-5.57	25.75-25.81
**19**	$C_{15}H_{20}ClN_5O$	55.99-56.17	6.26-6.27	21.76-21.82
**20**	$C_{15}H_{19}Cl_2N_5O$	50.57-50.69	5.38-5.40	19.66-19.72
**21**	$C_{15}H_{20}N_6O_3$	54.21-54.38	6.07-6.09	25.29-25.33

**22**	$C_{16}H_{20}N_6O$	61.52-61.72	6.45-6.46	26.90-27.0
**23**	$C_{13}H_{15}ClN_4O_2$	52.98-53.09	5.13-5.15	19.01-19.06
**24**	$C_{13}H_{14}Cl_2N_4O_2$	47.43-47.57	4.29-4.30	17.02-17.07
**25**	$C_{13}H_{15}N_5O_4$	51.15-51.28	4.95-4.96	22.94-23.01
**26**	$C_{14}H_{15}N_5O_2$	58.94-59.13	5.30-5.31	24.55-24.61
**27**	$C_{15}H_{20}ClN_5O_2$	53.03-53.68	5.59-6.25	20.44-21.12
**28**	$C_{16}H_{20}N_6O_2$	58.23-58.81	5.84-6.44	25.25-25.94
**29**	$C_{13}H_{15}ClN_4O$	55.73-56.31	5.11-5.73	19.91-20.29
**30**	$C_{13}H_{14}Cl_2N_4O$	49.63-50.09	4.36-4.68	17.65-18.09
**31**	$C_{13}H_{15}N_5O_3$	53.8-54.11	5.05-5.34	23.96-24.39
**32**	$C_{14}H_{15}N_5O$	62.13-62.71	5.45-5.95	25.65-26.14
